# LMAS: evaluating metagenomic short *de novo* assembly methods through defined communities

**DOI:** 10.1093/gigascience/giac122

**Published:** 2022-12-28

**Authors:** Catarina Inês Mendes, Pedro Vila-Cerqueira, Yair Motro, Jacob Moran-Gilad, João André Carriço, Mário Ramirez

**Affiliations:** Instituto de Microbiologia, Instituto de Medicina Molecular, Faculdade de Medicina, Universidade de Lisboa, 1649-028 Lisboa, Portugal; Instituto de Microbiologia, Instituto de Medicina Molecular, Faculdade de Medicina, Universidade de Lisboa, 1649-028 Lisboa, Portugal; Faculty of Health Sciences, Ben-Gurion University of the Negev, 8410501 Beer-Sheva, Israel; Faculty of Health Sciences, Ben-Gurion University of the Negev, 8410501 Beer-Sheva, Israel; Instituto de Microbiologia, Instituto de Medicina Molecular, Faculdade de Medicina, Universidade de Lisboa, 1649-028 Lisboa, Portugal; Instituto de Microbiologia, Instituto de Medicina Molecular, Faculdade de Medicina, Universidade de Lisboa, 1649-028 Lisboa, Portugal

**Keywords:** shotgun metagenomics, *de novo* assembly, benchmark, draft genome quality, simulation

## Abstract

**Background:**

The *de novo* assembly of raw sequence data is key in metagenomic analysis. It allows recovering draft genomes from a pool of mixed raw reads, yielding longer sequences that offer contextual information and provide a more complete picture of the microbial community.

**Findings:**

To better compare *de novo* assemblers for metagenomic analysis, LMAS (Last Metagenomic Assembler Standing) was developed as a flexible platform allowing users to evaluate assembler performance given known standard communities. Overall, in our test datasets, *k*-mer De Bruijn graph assemblers outperformed the alternative approaches but came with a greater computational cost. Furthermore, assemblers branded as metagenomic specific did not consistently outperform other genomic assemblers in metagenomic samples. Some assemblers still in use, such as ABySS, MetaHipmer2, minia, and VelvetOptimiser, perform relatively poorly and should be used with caution when assembling complex samples. Meaningful strain resolution at the single-nucleotide polymorphism level was not achieved, even by the best assemblers tested.

**Conclusions:**

The choice of a *de novo* assembler depends on the computational resources available, the replicon of interest, and the major goals of the analysis. No single assembler appeared an ideal choice for short-read metagenomic prokaryote replicon assembly, each showing specific strengths. The choice of metagenomic assembler should be guided by user requirements and characteristics of the sample of interest, and LMAS provides an interactive evaluation platform for this purpose. LMAS is open source, and the workflow and its documentation are available at https://github.com/B-UMMI/LMAS and https://lmas.readthedocs.io/, respectively.

## Background

Short-read shotgun metagenomics has the potential to offer comprehensive microbial detection and characterization of complex clinical or environmental samples. Despite becoming an increasingly used approach, it comes at the cost of producing massive amounts of data that require expert handling and processing, as well as adequate computational resources. The *de novo* assembly process is key when analyzing metagenomic data since it allows recovering contigs representing the replicons present in the sample, be it prokaryotic chromosomes, plasmids, or viruses, from a pool of mixed raw reads. These contigs are longer sequences that offer better contextual information than reads alone and provide a more complete picture of the microbial community than the species composition. Despite efforts for the development, standardization, and assessment of software for metagenomic analysis, both commercial and open source [1–5], the *de novo* assembly process still represents a critical point in these analyses.

The assembly of draft genomes has become a central step when analyzing pure bacterial cultures, for instance, allowing genomic comparisons through single-nucleotide polymorphisms (SNPs) or gene-by-gene methods, such as core-genome multilocus sequence typing (cgMLST). de Bruijn graph (dBg) algorithms are currently the most widely used approaches in modern assembly software. dBg handles unresolvable repeats by essentially fragmenting the sequence, that is, forming multiple contigs for each of the possibly contiguous sequences present in the sample. Additionally, the inherent heterogeneity of complex samples, potentially containing a multitude of replicons, could make traditional genome assemblers, implementing optimizations based on the assumption of having a single genome in the sample, not suitable for metagenomics [6].

Several dedicated metagenomic assembly tools for short-read data are available [6]. These tools are generally assumed to perform better when dealing with complex samples having a combination of intragenomic and intergenomic repeats and uneven and low-coverage sequencing depths of some of the replicons [7]. Not using dedicated metagenomic assemblers was suggested to come with the cost of generating artificial variation and chimeric contigs, especially in samples that contain closely related species [8]. However, no formal comparison has looked at increased accuracy or gains in contiguity of assemblies obtained with metagenomic assemblers versus traditional assemblers.

With an ever-increasing range of both traditional and metagenomic assemblers becoming available, choosing the best-performing tool can be an arduous and time-consuming task since the choice may vary depending on the purpose of the analysis, organism of interest, complexity of the sample, and computational infrastructure available. Additionally, the evaluation of the resulting contigs is not straightforward since one metric is not sufficient to classify an assembly, particularly with complex samples [7, 9]. Despite several *de novo* assembly validation methods relying on features of the created contigs themselves, such as QUAST [10], being useful in identifying inconsistencies indicative of potential assembly errors, the use of reference-based validation methods offers the possibility of a more complete evaluation of accuracy and is particularly important to benchmark attempts to reconstruct communities. MetaQUAST [11], a modification of QUAST, extends the original software by performing assembly evaluation based on aligning contigs to a reference, which can be provided or inferred by the software, and reports, in addition to the standard metrics for single genomes reported by QUAST, the number of interspecies translocations and the number of possibly misassembled contigs.

The use of mock communities, with known composition, abundance, and genomic information, provides a ground truth against which the success of the assembly of a complex sample can be evaluated. Furthermore, this can be done in circumstances in which the errors introduced by sample processing and the sequencing and associated methods used approximate, as much as possible, their effects in real samples. Such mock communities facilitate the identification of misassemblies, such as chimeric sequences generated from the improper combination of 2 distinct replicons, indels, or single-nucleotide variants improperly created by the assembler. On the other hand, the comparison of the performance of 2 assemblers is only possible if the input data are the same and if the same evaluation metrics are applied [3].

To tackle these challenges, we developed LMAS (Last Metagenomic Assembler Standing), an automated workflow to enable the benchmarking of traditional and metagenomic prokaryotic *de novo* assembly software using defined mock communities. The results of LMAS are presented in an interactive HTML report where selected global and reference replicon-specific performance metrics can be explored. The mock communities can be provided by the user to better reflect the samples of interest. New assemblers can be added with minimal changes to the pipeline so that LMAS can be expanded to include novel algorithms as they are developed. The portability and ease of use of LMAS are intended to provide users with a continuous benchmarking platform to easily evaluate the performance of assemblers, in mock communities mimicking as closely as possible their samples of interest.

## The LMAS Workflow

### Workflow overview

**Figure 1:**
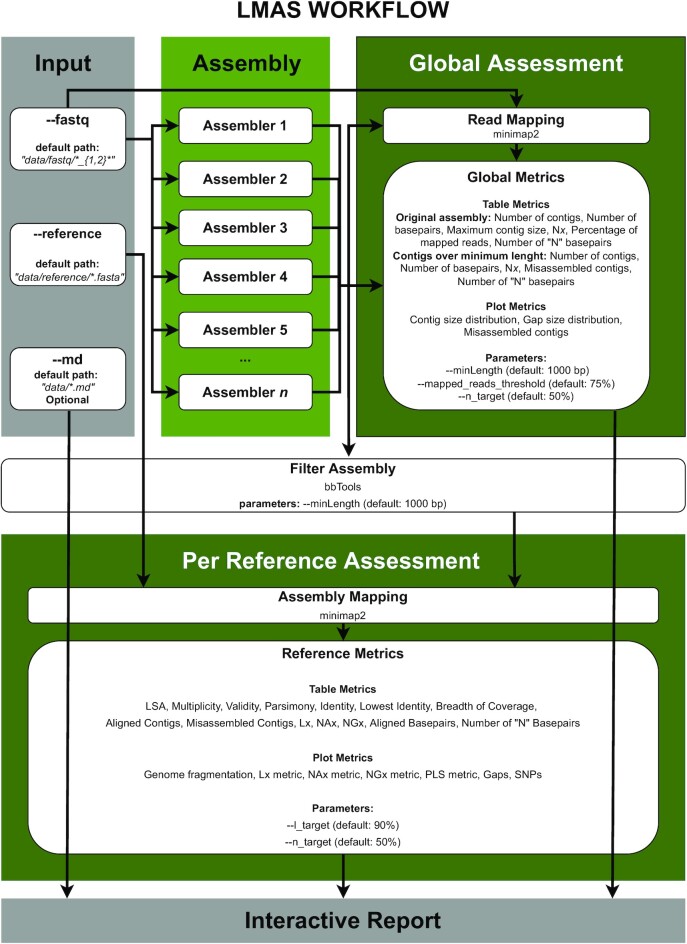
The LMAS workflow. The input sequencing data are assembled in parallel, resources permitting, by the set of assemblers included in LMAS. The resulting contigs are processed and the global quality assessment is performed. After filtering for the user-defined minimum contig size, the remaining sequences are mapped against the provided reference and the resulting information is processed to evaluate assembly quality by replicon in the reference file. All results, as well as optional text information describing the samples, are grouped in the LMAS report.

LMAS is a user-friendly automated workflow enabling the benchmarking of traditional and metagenomic prokaryotic *de novo* assembly software using defined mock communities (Fig. 1). LMAS was implemented in Nextflow [12] to provide flexibility and ensure the transparency and reproducibility of the results. LMAS relies on the use of Docker [13] containers for each assembler, allowing versions to be tracked and changed easily.

### Installation and usage

LMAS can be installed through Bioconda [14] or GitHub [15], with detailed instructions available in the documentation [16]. LMAS requires as inputs the complete reference replicons (genomes, plasmids, or any other replicons present) and short-read paired-end raw data. All complete references (linear replicons) should be provided in a single file. These raw data can be obtained *in silico* by creating simulated reads from the reference replicons or sequencing mock communities of known composition. Optionally, information on the input samples in a markdown file can be provided to be presented in the report.

A step-by-step execution tutorial is available at [17]. Users can customize the workflow execution either by using command-line options or by modifying the simple plain-text configuration files. To make the execution of the workflow as simple as possible, a set of default parameters and directives is provided. A complete description of each parameter is available in Supplementary Material (see Supplementary Material, Workflow parameters), as well as in the documentation [18]. The results are presented in an interactive HTML report, stored in the “*report*” folder in the directory of LMAS’s execution. The output files of all assemblers and quality assessment processing scripts in the workflow are stored in the “*results*” folder, in the same location.

### Supported assemblers and selection criteria

A collection of *de novo* assembly tools was compiled, including OLC and dBg assembly algorithms, the latter including both single *k*-mer and multiple *k*-mer value approaches, and hybrid assemblers implementing both algorithms, including both genomic and metagenomic assemblers (Supplementary Table S1). Of these, 11 assemblers were selected based on the date of last update (at least 2015) and are implemented in LMAS: ABySS [19] (version 2.3.1), GATB Minia Pipeline [20] (commit hash 9d56f42), IDBA-UD [21] (version 1.1.3), MEGAHIT [22] (version 1.2.9), MetaHipMer2 [23] (version 2.0.0.65-gaad446d-dirty-AddGtest), metaSPAdes [24] (version 3.15.3), minia [25] (version 3.2.6), SKESA [26] (version 2.5.0), SPAdes [27] (version 3.15.3), Unicycler [28] (version 0.4.9), and VelvetOptimiser [29] (commit hash 092bdee) (Table 1). The execution commands for each assembler are available as Supplementary Material (see Supplementary Material, Short-read de novo assemblers) and in the documentation [30].

**Table 1:** Prokaryotic *de novo* assemblers integrated into LMAS

Assembler	Type	Algorithm
**GATBMiniaPipeline**	Metagenomic	Multiple *k*-mer de Bruijn graph
**IDBA-UD**	Metagenomic	Multiple *k*-mer de Bruijn graph
**MEGAHIT**	Metagenomic	Multiple *k*-mer de Bruijn graph
**MetaHipMer2**	Metagenomic	Multiple *k*-mer de Bruijn graph
**metaSPAdes**	Metagenomic	Multiple *k*-mer de Bruijn graph
**ABySS**	Genomic	Single *k*-mer de Bruijn graph
**MINIA**	Genomic	Single *k*-mer de Bruijn graph
**SKESA**	Genomic	Multiple *k*-mer de Bruijn graph
**SPAdes**	Genomic	Multiple *k*-mer de Bruijn graph
**Unicycler**	Genomic	Multiple *k*-mer de Bruijn graph
**VelvetOptimizer**	Genomic	Single *k*-mer de Bruijn graph

New assemblers can be added with minimal changes to the pipeline so that LMAS can be expanded as novel algorithms are developed. A template is available to facilitate their integration, and a step-by-step guide is included in the documentation [31]. The only 2 requirements for the addition of a new assembler are the execution command for the assembler for paired-end short-read data and a Nextflow-compatible container with the assembler and any dependencies.

### Assembly quality metrics

The success of an assembly is evaluated in 2 steps: globally (see Global metrics) and relative to each of the replicons present in the sample (see Per reference metrics). In both, the tabular presentation in the reports allows the comparison of exact values between assemblers, and the interactive plots allow a more intuitive overview and easy exploration of results. In addition to the assembly success metrics, computational resource statistics are registered for each assembler (see Supplementary Material, LMAS metrics, Computational performance metrics).

#### Global metrics

The computation of the global metrics is performed through statistics inherent to the complete set of contigs assembled per sample, independent of the species/sample of origin. The metrics are presented, in tabular form, for the complete set of contigs and those filtered for a minimum length, and also graphically for the contigs filtered for a minimum length. The statistics include information on contig number, size, and ambiguous bases and the proportion of reads mapping to the created contigs. Two statistics are a consolidation of per reference metrics: misassemblies (i.e., contigs that do not reflect the structural organization in the reference replicons) and the overall size of gaps in all reference replicons not covered by any contig. A more detailed description of all global metrics is available in Supplementary Material (see Supplementary Material, LMAS metrics, Global metrics).

#### Per reference metrics

For the computation of the reference-based metrics, only the filtered set (FS) contigs are considered, for each reference replicon in the sample. These contigs are the ones exceeding the user-defined minimum sequence length, filtered using BBTools (version 38.44). After this initial step, the contigs are mapped to the reference replicons with minimap2 [32] (version 2.22). The metrics are computed through custom Python code (see Supplementary Material, Assembly filtering and mapping) for each replicon in the file provided as input. A detailed description of all reference-based metrics is available in Supplementary Material (see Supplementary Material, LMAS metrics, Per reference metrics).

In addition to the statistics shared with the global metrics, LMAS also calculates the number of mismatches relative to each reference, the COMPASS [9] metrics, and 2 new metrics we propose: LSA and Pls.

LSA represents the fraction of the longest single alignment between a contig and the reference, relative to the reference length. The Pls, or Phred-like score, is a scoring function based on the identity of each aligned contig to the reference replicon. Similarly to the Phred quality score [33], a measure of the quality of the identification of the bases by sequencing, the Pls measures the quality of the assembly of a contig. The formula of Pls is similar to the Phred score formula but uses as the error function the identity of the base in the contig to that of the reference replicon. The formula to obtain the Pls metric per contig is Equation 1.

(1) \begin{eqnarray*}
Phred\,\,(E) = \left\{ \begin{array}{@{}*{1}{c}@{}} {- \log (E) \times 10\,\,\,\,\,\,\,\,\,\,\,\,\,\,\,\, {\rm if}\,\, 0\,\, < E \le 1}\\ 60\,\,\,\,\,\,\,\,\,\,\,\,\,\,\,\,\,\,\,\,\,\,\,\,\,\,\,\,\,\,\,\,\ {\rm if\,\, E = 0} \end{array}\right. \end{eqnarray*}

where $E\ = \ 1 - Identity$.

### The LMAS report

**Figure 2:**
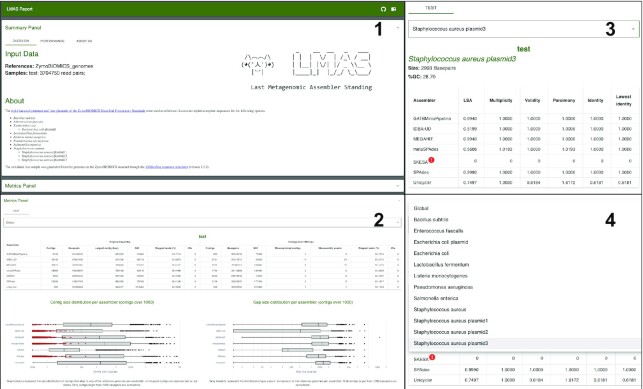
The LMAS report. All results, as well as optional text information describing the samples, are grouped in the LMAS report, an interactive and responsive HTML file, for exploration in any browser. Links for LMAS source code and documentation are available in the top-right corner of the report. (1) The summary panel of the LMAS report contains information on the input reference sequences and raw sequencing data samples (provided by the user) and the overall computational performance of the assemblers in LMAS. (2) The LMAS metric panel contains the explorable global and reference-specific performance metrics per input raw sequencing data sample. The tabular presentation allows direct comparison of exact values between assemblies, and the interactive plots allow for an intuitive overview and easy exploration of results. (3) If an assembler fails to produce an assembly or fails to assemble sequences that map to the reference replicon, it is marked in the table with a red warning sign. (4) The global or reference replicon-specific metrics can be accessed for each sample in the drop-down menu.

The LMAS results are presented in an interactive HTML (Fig. 2). The LMAS report is composed of 2 main panels: a top summary panel with information on input samples (provided by the user) and the resources used during LMAS’s execution and a bottom panel where selected global and reference-specific assembly metrics can be explored for each sample. LMAS constructs the HTML file after workflow completion, storing it in the “reports” folder. The report data can be easily shared between users and requires only a browser for visualization.

#### Summary panel

The top panel of the report contains information on the input samples and overall performance of the assemblers in LMAS, divided into 3 tabs: Overview, Performance, and About us. On the top-right corner of the report, direct links to LMAS’s source repository and documentation are provided.

- *Overview*: This tab contains information on the input data, including the name and number of reads of the raw sequencing data, as well as the name of the reference file. Additional information provided by the user about the community used as input is also presented here.
- *Performance*: This tab contains a table with information on the version, the containers used, and computational performance metrics for each assembler in LMAS.
- *About us*: This tab contains information on the LMAS GitHub repositories and the LMAS development team.

#### Metrics panel

The bottom portion of the report contains the explorable global and reference-specific performance metrics per input raw sequencing data sample. Each sample has its own tab, and the global or reference replicon-specific metrics can be accessed in the drop-down menu.

##### Global metrics

A table displays the global assembly metrics computed for the complete and FS contigs. If an assembler fails to produce an assembly, it is marked on the table with a red warning sign. The global metric plots are interactive, allow zooming in on particular areas, and provide extra information as hover text boxes. The plots can be saved as PNG in whatever view the user selects.

##### Per reference metrics

Similarly to the global assembly metrics, a table displays the computed set of reference-restricted metrics for the FS contigs. If an assembler fails to produce sequences that align to the reference, these are marked in the table with a red warning sign. Information on the expected reference replicon length and the GC content is calculated from the input files and reported above the table. The per reference metric plots are also interactive, allowing the same type of operations as the global metric plots.

### Comparison with other assembly evaluation software programs

The assessment and evaluation of genome assemblies has been a relevant field ever since the emergence of the assembly process itself, and therefore many solutions have been proposed [3, 7, 9–11, 34–36]. The Critical Assessment of Metagenome Interpretation (CAMI) proposed a set of recommendations and best practices for benchmarking in microbiome research [37]. These recommendations include the reporting of computational performance, which may condition the choice of software by the users, such as runtime, disk space, and memory consumption, also reported by LMAS (see Supplementary Material, LMAS metrics). As also suggested by CAMI, LMAS tracks the exact program version and command-line calls through its implementation in Nextflow. Moreover, using containerized assemblers and being easily installable through Bioconda, LMAS facilitates deployment in diverse user machines. Unlike the CAMI tutorial, in which users are asked to download and install the necessary tools, in LMAS, everything is provided in a one-stop reproducible workflow that effortlessly handles all preprocessing, assembly, postprocessing, traceability, and report production steps, freeing users to focus on providing relevant samples for analysis and interpreting the results in view of the intended applications.

Concerning software for assembly quality assessment currently available, the most widely adopted is QUAST [10] or, when dealing with metagenomic data, its extension metaQUAST [11], which was also adopted by the CAMI challenges [3, 5] and suggested in the CAMI Tutorial [37]. Although several features of these tools overlap with LMAS’s quality assessment components, these differ from LMAS in the sense that they are not a single-step workflow allowing a traceable and reproducible assembly of mock communities. Unlike QUAST and metaQUAST, whose purpose is to evaluate assemblies, the purpose of LMAS is to allow users to evaluate assembler performance for a given sample of interest. Supplementary Table S2 shows the comparison of the output and computed assembly quality metrics generated by LMAS, QUAST, and metaQUAST.

## Results and Discussion

To illustrate the use of LMAS and evaluate the performance of the chosen assemblers, we initially used the 8 bacterial genomes and 4 plasmids of the ZymoBIOMICS Microbial Community Standards as reference. As input, we used the raw sequence reads of mock communities with an even and logarithmic distribution of species, from real sequencing runs [38] and simulated read datasets, with and without error, matching the distribution of species in each sample [39]. Our dataset is composed of samples ENN (*in silico* generated evenly distributed without error), EMS (*in silico* generated evenly distributed with Illumina MiSeq error model), ERR2984773 (evenly distributed real Illumina MiSeq sample), LNN (*in silico* generated logarithmically distributed without error), LHS (*in silico* generated logarithmically distributed with Illumina HiSeq error model), and ERR2935805 (logarithmically distributed real Illumina HiSeq sample) (see Supplementary Table S3). Detailed information about the generation of the input samples is available as Supplementary Material (see Supplementary Materials, ZymoBIOMICS Microbial Community Standards, Supplementary Table S4). To evaluate the reproducibility of an assembler performance, the LMAS workflow was run 3 times for all samples using default parameters, and the resulting data were processed for each sample (see Supplementary Materials, Assessment of assembly success). Supplementary Tables S5 to S10 present an overview of the average global performance per assembler for each sample in LMAS.

To test assembler performance with an even more complex dataset, we used the 12 strain BMock community standard (accession SRX4901583, real Illumina HiSeq 2500 sample) [40]. This sample includes a noneven distribution of species, with the most abundant replicon having 3,093× coverage (*Muricauda* sp. ES.050) and the lowest only 0.1× coverage (*Micromonospora coxensis*) (Supplementary Table S24). For the sake of a less resource-intense evaluation, we downsampled to have 20% of the reads available in the original sample and further processed only these. The main challenges of this dataset are possibly to assemble the genomes of the 3 *Micromonospora*spp. and the 2 *Halomonas*spp. strains, which have an Average Nucleotide Identity through BLAST (ANIb) >0.84 and 0.98, respectively (Supplementary Table S25). The 2 Marinobacter spp. have an ANIb of 0.78.

To represent more realistic samples of a human microbiome study, the Gut-Mix-RR and Gut-Mix-HiLo standards, including 20 species known to be present in the human gut, were used as reference (accessions SRR11487941 and SRR11487935, respectively, both real Illumina MiSeq samples) [41]. For the Gut-Mix-RR, the abundance of each bacterial genome is relatively even (maximum of 66×, an average of 22.32×, and a minimum of 5.99×; Supplementary Table S28). The Gut-Mix-HiLo has an uneven abundance of species (maximum of 115×, an average of 20.45×, and a minimum of 0.34×; Supplementary Table S28). The genomes in these mock communities are fairly diverse (average ANIb = 0.67, Supplementary Table S29), with the 2 subspecies of *Bifidobacterium longum* (ANIb = 0.95) possibly being the most challenging. It is worth noting that only draft genomes are available for 8 of the strains, including one of the *B. longum* subspecies (Supplementary Table S28). The *Roseburia hominis* and *Roseburia intestinalis* are the closest related closed replicons (ANIb >0.77) in this sample.

### Some assemblers perform poorly

**Figure 3:**
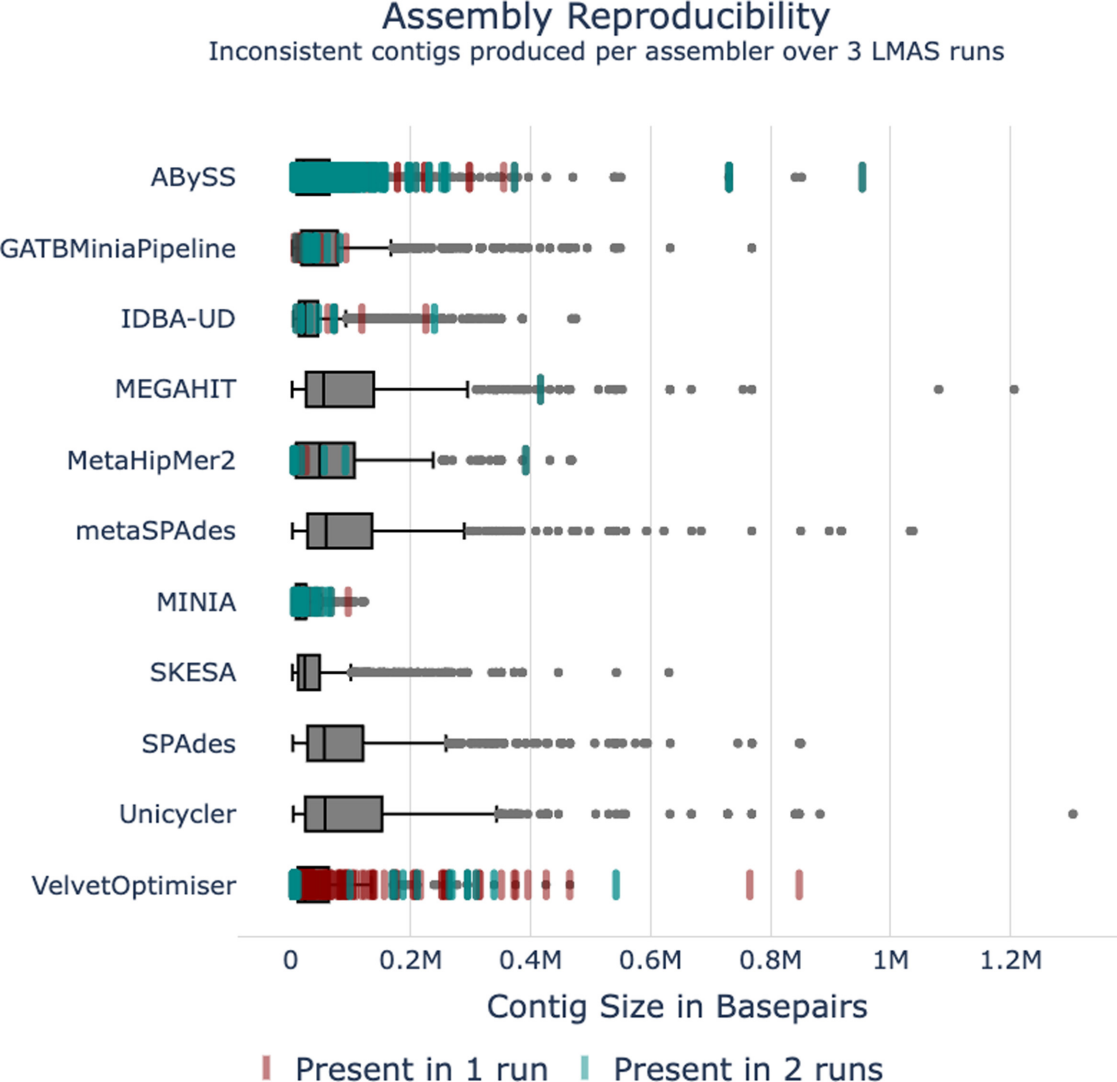
Assembly reproducibility. Inconsistent contigs produced per assembler over 3 LMAS runs. The distribution of contig sizes, in base pairs, consistently present in all 3 LMAS runs are indicated in the gray boxplots for each assembler. If an assembler produced a contig only present in 2 of the runs (as determined by its size), its size is indicated in teal. If a contig is present in a single run, it is represented in red.

Of the 12 *de novo* prokaryotic assemblers included in LMAS, 4 stand out as having an overall poor performance in the ZymoBIOMICS Microbial Community Standards dataset: ABySS, MetaHipmer2, minia, and VelvetOptimiser. Both ABySS and MetaHipmer2 performed inconsistently with differing resource requirements for the same sample in different runs, namely, runtime and memory allocation (see Supplementary Materials, Resource requirements differ greatly, Supplementary Fig. S2). Moreover, ABySS failed to produce an assembly for sample ERR2984773 for 1 of the runs (see Supplementary Table S7) and for sample LHS in any of the 3 runs in the time limit of 3 days (see Supplementary Table S9), and MetaHipmer2 failed to produce an assembly for samples LNN and LHS in all 3 runs (see Supplementary Tables S8–S9). VelvetOptimiser generated the highest number of inconsistent contigs across the 3 LMAS runs (Fig. 3, Supplementary Table S11), with 1.69% of the total contigs created present in only 1 or 2 runs. Although not as extreme as VelvetOptimiser, ABySS (0.52%), minia (0.14%), GATBMiniaPipeline (0.32%), MetaHipMer2 (0.11%), and IDBA-UD (0.08%) also showed inconsistencies in contig size.

**Figure 4:**
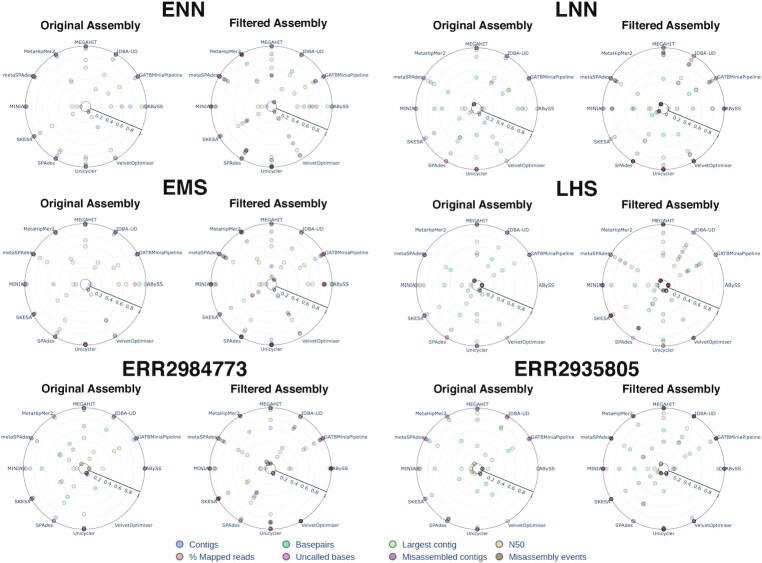
Assembler performance for the ZymoBIOMICS Microbial Community Standards dataset. For each sample in the dataset, the best score of each assembler in the 3 LMAS runs was selected. The results for each global assembly metric were normalized, with 1 representing the best result and 0 the worst. For the original assembly, the following metrics are presented: number of contigs produced (in blue), number of base pairs produced (in teal), the size of the largest contig assembled (in green), N50 (in yellow), percentage of mapped reads to the assembly (in orange), and uncalled bases (in red). For the filtered assembly, the additional metrics are presented: number of misassembled contigs (in purple) and number of misassembly events (in brown).

Regarding the quality assessment of the assemblies produced (Fig. 4, Supplementary Table S12), ABySS and minia are the only single *k*-mer dBg assemblers in the collection and were found to mostly underperform relative to their multiple *k*-mer dBg counterparts as reported previously [3, 37, 42, 43], generally resulting in more fragmented assemblies, although there were significant differences in performance across samples. Among multiple *k*-mer assemblers, VelvetOptimiser frequently produced a very high number of contigs of very small size (over 99% of the contigs not surpassing the minimum length of 1,000 bp) and therefore a low N50 (an average of 29,768 bp versus a global average of 84,114 bp) (Supplementary Tables S5–S10). Additionally, ABySS and VelvetOptimizer produced contigs with a very large number of *N*s, with an average of 1,019 and 3,035 uncalled bases per assembly, respectively. MetaHipMer2, although having overall average metrics in the 2 evenly distributed mock samples (ENN and EMS, Supplementary Tables S5–S6) where it was able to run successfully, severely underperformed in the real samples (ERR2984773 and ERR2935805, Supplementary Tables S7 and S10). Generally, the performance scores of the assemblers decreased considerably for the real samples in comparison with the simulated ones, either with or without error, underscoring the importance of using mock samples instead of simulated reads to evaluate assembler performance. High utilization of the reads in the dataset is observed for most assemblers, with on average at least 90% of the reads mapping back to the assembly, except for ABySS, MetaHipMer2, and VelvetOptimiser, whose values are in the range of 46% to 79%. Despite an overall good performance, SPAdes produced the highest number of misassembled contigs in the logarithmically distributed sample, with an average of 98 and a maximum of 572 (sample ERR2935805, Supplementary Table S10), in comparison to the global average of 11 misassembled contigs for all assemblers across all samples. However, this behavior was not consistent across samples, with the evenly distributed sample showing similar misassembled contigs between SPAdes and other assemblers, similarly to the other mock samples tested (see below).

Due to their poor performance discussed above, the following assemblers have not been included in subsequent analyses: ABySS, MetaHipmer2, minia, and VelvetOptimiser.

### Metagenomic dedicated assemblers do not outperform genomic assemblers

**Figure 5:**
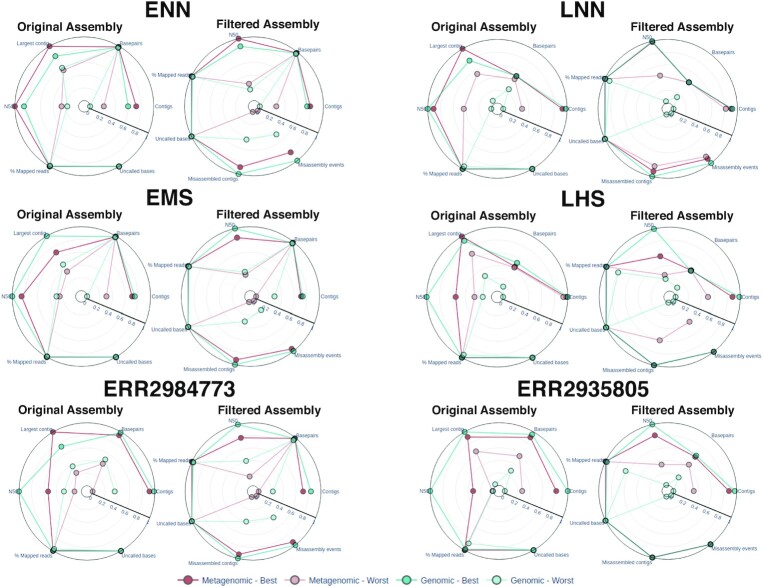
Performance of genomic and metagenomic assemblers for the ZymoBIOMICS Microbial Community Standards dataset. For each sample in the dataset and for the 3 runs, the best and worst scores for each assembler category were selected: genomic (in blue) and metagenomic (in red). The results for each global assembly metric were normalized, with 1 representing the best result and 0 the worst. For the original assembly, the following metrics are presented: number of contigs produced, number of base pairs produced, the size of the largest contig assembled, N50, percentage of mapped reads to the assembly, and uncalled bases. For the filtered assembly, the additional metrics are presented: number of misassembled contigs and number of misassembly events.

After excluding the poorly performing assemblers, LMAS includes 3 genomic (SKESA, SPAdes, and Unicycler) and 4 labeled as metagenomic-specific (GATBMiniaPipeline, IDBA-UD, MEGAHIT, and metaSPAdes) *de novo* prokaryotic assemblers, all implementing multiple *k*-mer dBg algorithms. As observed in Fig. 5, Supplementary Table S13, and Supplementary Fig. S3, there were very significant differences between the best- and the worst-performing assemblers of each type for the ZymoBIOMICS Microbial Community Standards dataset, with this difference being more pronounced for metagenomic assemblers. The best-performing assemblers of each type behaved frequently quite similarly, and the differences between them tended to be attenuated after filtering for contigs <1 kbp. Still, for the linearly distributed samples (ENN, EMS, and ERR2984773), the overall worst performers tended to be metagenomic assemblers. In contrast, for the logarithmically distributed samples (LNN, LHS, and ERR2935805), the opposite was observed, with genomic assemblers tending to be the worst performing (Fig. 5). For the logarithmically distributed samples, the number of base pairs recovered is significantly lower than expected from their composition for both genomic and metagenomic assemblers, particularly after filtering (Supplementary Table S13), as contigs representing the less abundant species are not recovered by either type of assemblers (see assembler performance is influenced by replicon abundance in the sample). For this dataset, the fact that an assembler is branded as genomic or metagenomic does not translate into better or worse performance in dealing with these complex samples, but rather characteristics of the individual assemblers themselves determine their performance.

In the BMock standard, similarly to the ZymoBIOMICS standard, no significant difference was observed between the genomic and metagenomic assemblers, particularly after filtering for contigs <1 kbp (Supplementary Table S26). Both *Marinobacter* replicons (2,615,840,697 and 2,616,644,829; 448× and 135× coverage, respectively) were successfully recovered by all genomic and metagenomic assemblers with >0.87 breadth of coverage (Supplementary Table S27). The 2 most abundant *Micromonospora* replicons (2,623,620,557 and 2,623,620,567; 15× and 18× coverage, respectively) were also recovered to a breadth of coverage >0.95 by most assemblers, except the genomic assemblers SKESA and Unicycler (Supplementary Table S27). All assemblers, with the exception of SKESA and GATBMiniaPipeline, recovered both *Halomonas* replicons (2,623,620,617 and 2,623,620,618) with a breadth of coverage of >0.84 (Supplementary Table S27). When considering this set of closely related replicons, metagenomic assemblers also did not perform consistently better than genomic assemblers in the number of misassembled contigs or SNPs relative to the reference genome. Among the metagenomic assemblers, GATBMiniaPipeline and IDBA-UD performed particularly well but with values close to those of the 2 best genomic assemblers (SPAdes and Unicycler). IDBA-UB performed significantly worse with the *Micromonospora* replicons, possibly because of their lower coverage. As could have been expected, the number of SNPs in the lower-coverage *Halomonas* replicon was consistently higher than in the one with higher coverage (1.6-fold to 85.0-fold depending on the assembler), despite their relatively modest (12.6%) difference in estimated coverage in the sample and very significant depth of coverage (>500×) (Supplementary Tables S24 and S27). However, when comparing the number of SNPs in the *Marinobacter* replicons, this relationship is reversed, with the replicon with higher coverage (448×) having more SNPs relative to the reference than the one with lower coverage (135×) (Supplementary Table S27). This indicates that other factors, such as characteristics of the replicon, the actual representation in the sample, and the closeness to other replicons in the sample, may influence the performance of assemblers. It is also interesting to see that the contigs generated by themselves do not allow the distinction of closely related strains, since the number of SNPs relative to the reference genomes (if the assembler is able to cover >0.79 of the genome) is in the ranges of 315 to 9,915 and 9,773 to 70,196 for the higher and lower depth of coverage *Halomonas* replicons, respectively.

For the Gut-Mix-RR and Gut-HiLo-RR mock communities, the same pattern was observed as with the other mock communities, with the differences between the metagenomic and genomic assemblers being attenuated after filtering for contigs <1 kbp (Supplementary Table S30 and S31). Particularly for the evenly distributed Gut-Mix-RR sample, when considering the subset of *Roseburia*spp., the replicon with the lowest coverage (12× for *R. intestinalis* versus 18× for *R. hominis*) had a consistently higher number of SNPs, with the exception of the SKESA assemblies, where the opposite was observed. This is similar to what was observed for the *Halomonas* replicons in the BMock12 community standard.

### Success is not straightforward

Several factors contribute to suboptimal performance of the assembly process, including DNA isolation and library preparation protocol; sequencing technology, depth, and read length; and possible contamination and inherent characteristics of the sample composition.

#### Assembler performance is influenced by species

**Figure 6:**
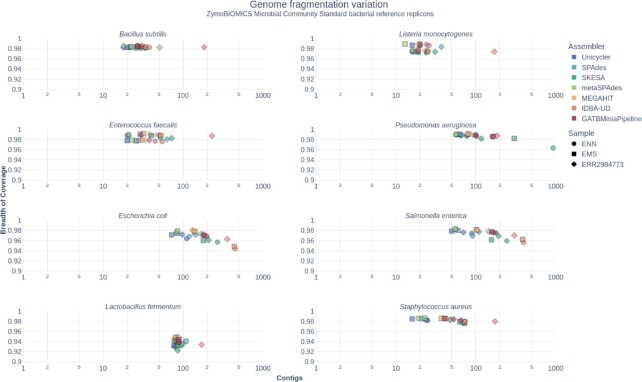
Genome fragmentation for each reference replicon of the ZimoBIOMICS community standards dataset for the evenly distributed samples. Genome fragmentation for the 3 LMAS runs is represented by the number of contigs and breadth of coverage of the reference per assembler for the evenly distributed samples: ENN (evenly distributed without error model, identified by a circle), EMS (evenly distributed with Illumina MiSeq error model, identified by a square), and ERR2984773 (real Illumina MiSeq sample, identified by a diamond). Each assembler is identified with the following color scheme—dark blue, Unicycler; light blue, SPAdes; dark green, SKESA; light green, metaSPAdes; yellow, MEGAHIT; orange, IDBA-UD; red, GATBMiniaPipeline.

For the 8 bacterial genomes present in the ZymoBIOMICS Microbial Community Standards dataset samples, even in those with an even distribution of the genomes (ENN, EMS, and ERR2984773), variations in the assembly metrics were observed (Fig. 6, Supplementary Figures S4–S6, Supplementary Tables S14–S16). For all samples in the dataset, the genomes are recovered almost completely, with all replicons being >90% represented in the resulting assemblies. *Lactobacillus fermentum* is the least represented genome (92.2%–94.9%). Most replicon sequences are recovered in <100 contigs, except for *Pseudomonas aeruginosa, Escherichia coli*, and *Salmonella enterica*, and not considering IDBA-UB, which frequently produces a larger number of contigs when compared to other assemblers. However, in other mock samples, this worse performance of IDBA-UD in terms of number of contigs is not so clear. The absolute values of other metrics of assembly quality, such as LSA, misassembly events, or uncalled bases, are also different between bacterial genomes (Supplementary Tables S14–S16). The fact that *S. enterica* is a closely related species to *E. coli*, with high level of genetic similarity (ANIb >0.8, Supplementary Table S23), could have created difficulties for resolving the assemblies in a mixed sample and led to the lower coverage observed, the higher number of contigs, and the increased number of misassembled contigs identified in these species in some samples. However, in the case of the larger number of contigs of *P. aeruginosa*, no related species are present in the sample, and these possibly reflect intrinsic properties of the replicon such as a high number of prophages integrated in the bacterial genome [44]. Similarly, replicon characteristics could be behind the lower breadth of coverage consistently observed in *L. fermentum* assemblies.

In the BMock12 and the Gut-Mix samples, which have pairs of much more similar replicons, it is true that the closely related replicons do have higher numbers of contigs. However, it is possible that the properties of the individual replicons also have an impact on the number of contigs generated by the assemblers (Supplementary Fig. S8). Another potential confounder that was not explored is the length of the reads, with Miseq samples having 300-bp reads and HiSeq samples having 150-bp reads.

##### Longer contigs have higher confidence

**Figure 7:**
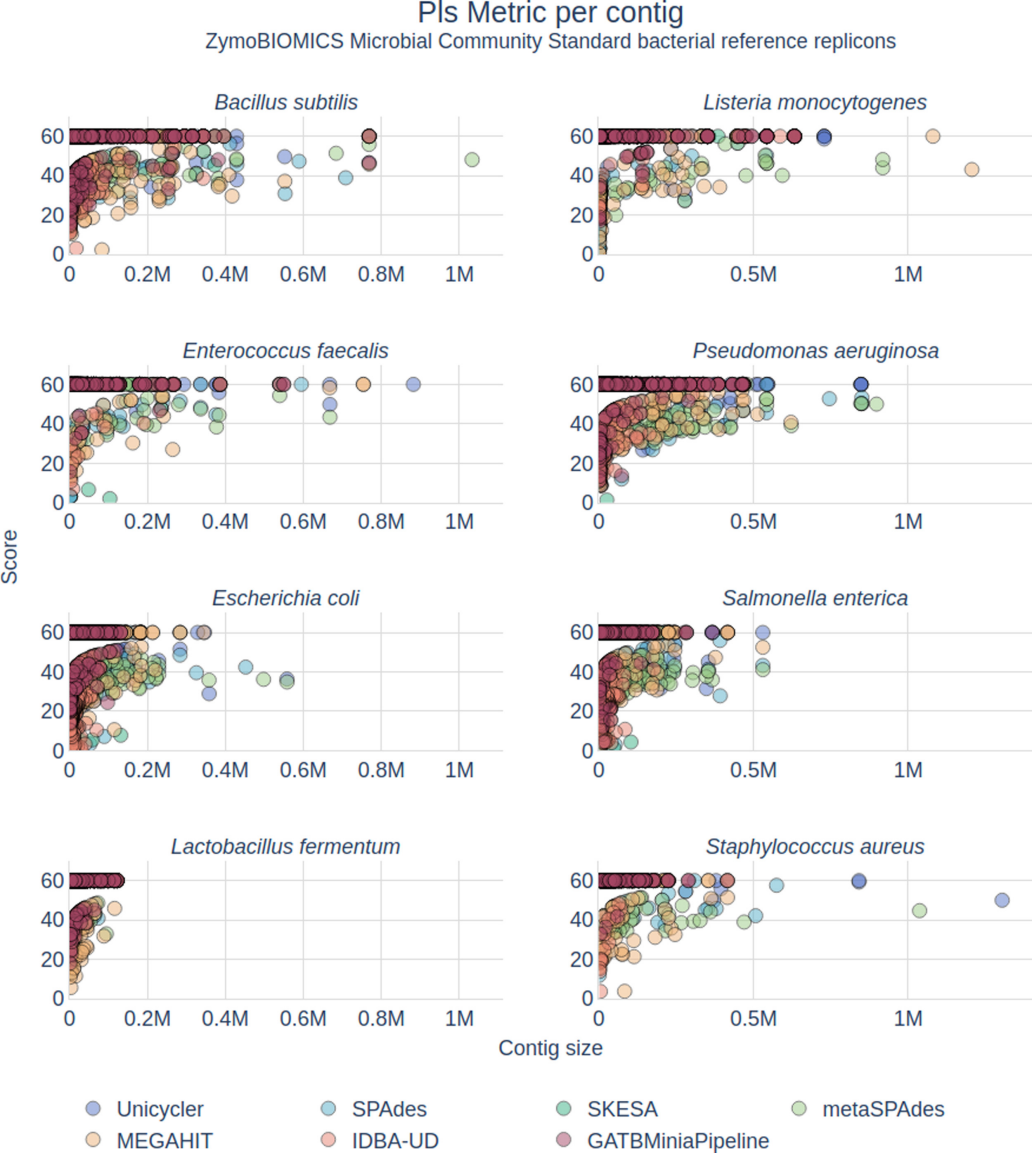
Phred-like score (Pls) per contig for each reference replicon of the ZimoBIOMICS community standards datasets. The Pls score was calculated for each unique contig produced by each assembler in 3 LMAS runs and is represented in relation to its contig size. Each contig is colored according to the assembler with the following color scheme—dark blue, Unicycler; light blue, SPAdes; dark green, SKESA; light green, metaSPAdes; yellow, MEGAHIT; orange, IDBA-UD; red, GATBMiniaPipeline.

The Pls metric, which measures the error rate of a contig relative to the reference, shows that for every replicon, longer contigs have higher Pls (Fig. 7). This could justify the option of filtering an assembly by length, even beyond the 1,000-bp minimum contig size implemented by default in LMAS. Not only are we eliminating shorter, less informative contigs in terms of genetic context, but these are also the ones most likely to contain errors relative to the reference sequence.

##### Certain genomic regions are problematic for all assemblers

**Figure 8:**
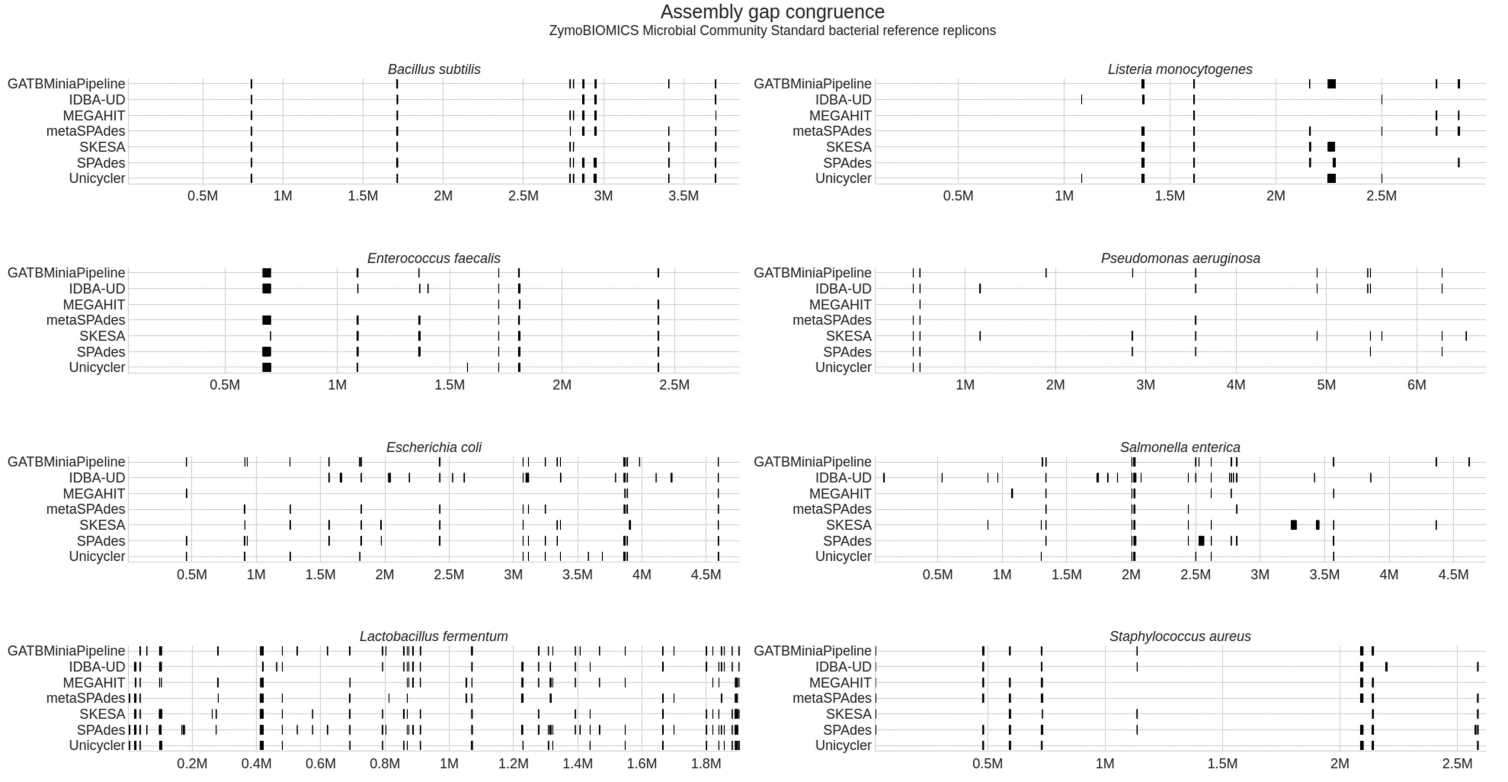
Location of gaps in comparison to the reference sequence, per assembler, for each reference replicon of the ZimoBIOMICS community standards datasets. The resulting plot contains the consistent gaps obtained from a 3-LMAS run for the evenly distributed dataset (ENN, EMS, and ERR2984773) for GATBMiniaPipeline, IDBA-UD, MEGAHIT, metaSPAdes, SKESA, SPAdes, and Unicycler assemblers.

Some genomic regions in several replicons are consistently a challenge for all assemblers. As observed in Fig. 8, all genomes of the ZymoBIOMICS Microbial Community Standards dataset present certain regions that fail to assemble for all tools in all runs, even those generating high-quality draft assemblies. Of all 7 assemblers considered, only GATBMiniaPipeline, MEGAHIT, and IDBA-UD showed inconsistency in the gaps produced over the 3 LMAS runs (Supplementary Table S17), as expected from producing variable sets of contigs. The regions consistently missing for all assemblers in all runs are rich in repetitive elements, such as ribsomal RNA and transfer RNA coding sequences and mobile genetic elements (Supplementary Table S18), with larger gaps corresponding to tandem sets of these elements. This reflects an intrinsic limitation of short-read sequencing since the length of a read pair is not enough to bridge across the repetitive element, preventing the generation of contigs representing these regions. This is something that could be addressed by the use of long-read sequencing technologies. Despite this, some assemblers are able to produce contigs that represent some of these large tandem regions, such as MEGAHIT and SKESA for *E. faecalis* and IDBA-UD, MEGAHIT, and metaSPADES for *L. monocytogenes*, but such performance is not consistent for all reference replicons. For instance, SKESA fails to assemble 2 large regions of the *S. enterica* genome that all other assemblers successfully cover.

For the BMock12 community standard, the same pattern of consistency of difficult regions across replicons can be observed for all replicons, with the exception of the lowest abundance replicons (see Supplementary Fig. S9). Interestingly, the 2 closely related *Halomonas* replicons present a very dissimilar gap pattern, with a high number of gaps (*n* = 2,789 and *n* = 2,702) distributed throughout the replicon sequence, which possibly reflects the difficulty of assembling closely related replicons in the same sample.

#### Assembler performance is influenced by replicon abundance in the sample

The logarithmically distributed samples (LNN, LHS, and ERR2935805) of the ZymoBIOMICS community standard dataset showed greater variation in the assembly success metrics than the evenly distributed samples (Supplementary Tables S8–S10), reflecting the difficulty of recovering sequences of the lowest abundant replicons. For the 3 replicons with an estimated depth of coverage >15×, a similar pattern is observed in logarithmically distributed samples as in evenly distributed samples, albeit with greater dispersion in the number of contigs generated and with a markedly decreased breath of coverage for some assemblers and samples in the logarithmically distributed samples (Fig. 6 and Supplementary Fig. S7). Almost no contigs >1,000 bp were retrieved for replicons with an estimated depth of coverage of <2×, resulting in a very low breadth of coverage (<1%) (Supplementary Table S4, Supplementary Table S22). This leads to a severe underrepresentation of the diversity of the community in the generated contigs, particularly of plasmid sequences due to their smaller length and abundance as was described previously [3, 45, 46]. This happens despite the greater sequencing depth of these samples versus those with an even distribution (>5-fold difference in the number of reads).

For the BMock12 community standard, the very low replicon abundance (*Micromonospora coxensis*, 2,623,620,609, 0.02× coverage) fails to assemble in all tools (Supplementary Fig. S9, Supplementary Table S24). The *Micromonospora echinaurantiaca* (2,623,620,557, 14.9× coverage) and *Micromonospora echinofusca* (2,623,620,567, 18.2× coverage) fail to assemble with SKESA and Unicycler, and the *Propionibacteriaceae* replicon (2,615,840,646, 31.9× coverage) fails to be assembled with SKESA. In the Gut-Mix-RR standards, there are several replicons with <1× to 20× depth of coverage. Significant breath of coverage (>0.7) was obtained with the contigs created by most assemblers, including successful assemblies of some of the replicons by SKESA and Unicycler that had failed with higher-coverage replicons in the BMock12 standard. Taking together these data and that of the ZymoBIOMICS standard, these suggest that it is hard to establish a universal breakpoint at which each of the assemblers is able to generate high breadth of coverage contigs, with the actual genome of interest and the composition of the sample possibly playing a role. Nevertheless, coverages >15× result in high breadth of coverage contigs, albeit in the lower range with many contigs and a significant number of SNPs.

## Conclusions

The purpose of LMAS is to empower users to test assembler performance in meaningful conditions for their experimental setup and objectives. Suitable mock communities, reproducing the users’ samples of interest, can be used as a gold standard to evaluate assembler performance. To illustrate LMAS’s functionalities, we analyzed 3 well-known samples used in several studies. Although the 8-species ZymoBIOMICS Microbial Community Standards might not be representative of the metagenomic complexity of the samples of interest of most researchers, we hoped that its relative simplicity meant that the results shown would represent a best-case scenario, since as sample complexity increases, so do the challenges to assembler performance. However, the results of the BMock12 and Gut-Mix community standards suggest that the actual genome of interest and community composition play an important part in the results of individual assemblers.

Our results showed significant differences in both global and reference-dependent assembly quality metrics generated by each *de novo* assembler. The performance of each assembler varied depending on the species of interest and its abundance in the sample, with less abundant species presenting a significant challenge for all assemblers. The fact that an assembler is branded as specific for metagenomics does not guarantee a better performance in metagenomic samples, with assemblers used for genomic assembly outperforming the worst metagenomic assembler tested. Even when considering communities with very similar replicons, the overall performance of metagenomic assemblers was not consistently better than that of genomic assemblers. The results also indicate that the recovery of assemblies allowing strain-level discrimination at the SNP level is highly unlikely based solely on the assembler-generated contigs. The following assemblers showed significant performance problems, and their usability may be limited, at least with the default parameters we used: ABySS, MetaHipmer2, minia, and VelvetOptimiser.

The choice of *de novo* assembler depends greatly on the computational resources available, the species of interest, its representation in the sample, and, possibly, the composition of the community in the sample. In our testing with any of the mock communities, no assembler stood out as an undisputed all-purpose choice for short-read metagenomic prokaryotic genome assembly, with different assemblers showing specific strengths. Users would thus benefit from analyzing the results of sequencing mock communities (ideally) or of artificially generated reads simulating their samples of interest to guide their choice of assembler. LMAS was developed to be an easy-to-use and flexible tool for this purpose. From the results that we obtained with various mock communities, the following assemblers performed consistently well (presented in alphabetical order): MEGAHIT, metaSPAdes, SKESA, SPAdes, and Unicycler. From our assessment, we conclude that these assemblers are the most likely candidates to perform well in other complex samples.

LMAS was built with modularity and containerization as keystones, leveraging the parallelization of processes and guaranteeing reproducibility across platforms. The modular design allows for new assemblers to be easily added and existing assemblers to be easily updated, allowing LMAS to function as a continuous benchmarking platform and ensuring its future relevance as improvements in assembly software are proposed. LMAS will also support evaluating the gains of any cumulative improvements to existing assemblers using the same benchmark set adapted to a specific project or goal. Such reproducibility, capacity to easily add assemblers of interest not included in the current version, and flexibility for future extensions are important principles in computational method benchmarking. Moreover, in addition, by lowering the barriers to perform comparisons between assemblers, LMAS will encourage users to compare software performance against mock communities of special interest, depending on their operational focus.

The interactive report provides an intuitive platform for data exploration, allowing the user to easily sift through global and reference-specific performance metrics for each sample, as well as providing information on the assemblers executed to allow traceability of the results. Producing an extensive, metric-rich report allows users interested in different aspects of assembler performance to make informed decisions, particularly when choosing among the top-performing assemblers, which show only minor differences.

LMAS applies several well-known assembly metrics and proposes 2 more: LSA, which represents the fraction of the longest single alignment between a contig and the reference, and Pls, a scoring function based on the identity of each aligned contig to the reference replicon. The entire set of assembly quality metrics used in LMAS allows not only the assessment of quality based on statistics inherent to a set of assembled contigs but also a comparison to a ground truth provided using samples of known composition and reference sequences. The LMAS report provides an interactive and intuitive platform for the exploration of these results, allowing users to easily test assemblers in mock samples with species composition and distribution relevant for their own studies.

Although computationally intensive due to the complex nature of the *de novo* assembly process, LMAS is the only software-integrating assembly and its evaluation into a single pipeline, guaranteeing the same conditions are met for all tools. With LMAS, it is now possible to continuously evaluate which *de novo* assembler produces the most relevant results for a given community of interest. The LMAS workflow is open source, and its code and documentation are available at https://github.com/B-UMMI/LMAS and https://lmas.readthedocs.io/, respectively.

## Availability of Supporting Source Code and Requirements

**Project name:** LMAS

**Project home page:**  https://github.com/B-UMMI/LMAS

**Operating system(s):** UNIX-like systems.

**Programming languages:** Nextflow, Python, Bash, Javascript

**Other requirements:** Java version 8 or highest. Docker/Singularity/Shifter

**License:** GNU GPL v3

**RRID:** SCR_022251

## Additional Files

**Supplementary Fig. S1**. LMAS misassembly classification. Misassembled contigs are classified into 6 main categories: chimera, insertion, deletion, inversion, rearrangement, translocation, and duplication, according to the mapping orientation, the distance between blocks in the contig, and the mapping coordinates in the reference replicon. If a contig is classified as being chimeric, no further classification is performed. The other categories are classified independently of each other, with combinations being possible, to better reflect the differences in comparison to the reference. If a contig is broken into multiple sequence blocks but fails to be classified in any of the previous categories, it is reported as being inconsistent.

**Supplementary Fig. S2**. Computational resources used by each assembler for the evenly and logarithmically distributed samples. Each plot describes the distribution of resource consumption for 3 LMAS runs for the ZymoBIOMICS Microbial Community Standards dataset for the following metrics: (A) CPU/hour, (B) maximum memory in GB, (C) data written to disk in GB, (D) data read from dist in GB, and (E) runtime in hours. The mean for all samples and all assemblers is indicated in red. The samples are indicated as follows: ENN: dark blue, EMS: teal, ERR2984773: green, LNN: light green, LHS: yellow, ERR2935805: light orange.

**Supplementary Fig. S3**. Performance per reference of genomic and metagenomic assemblers for the evenly distributed samples in the ZymoBIOMICS Microbial Community Standards dataset. For each sample in the dataset and for the 3 runs, the best and worst scores for each assembler category were selected: genomic (in blue) and metagenomic (in red). The results for each global assembly metric were normalized, with 1 representing the best result and 0 the worst.

**Supplementary Fig. S4**. Assembler performance per reference for the ZymoBIOMICS Microbial Community Standards dataset for sample ENN. The best score for each assembler was selected for 3 LMAS runs. The results for each global assembly metric were normalized, with 1 representing the best result and 0 the worst. The following assemblers are represented: GATBMiniaPipeline: dark blue, IDBA-UD: light blue, MEGAHIT: dark green, metaSPAdes: light green, SKESA: yellow, SPAdes: orange, Unicycler: red.

**Supplementary Fig. S5**. Assembler performance per reference for the ZymoBIOMICS Microbial Community Standards dataset for sample EMS. The best score for each assembler was selected for 3 LMAS runs. The results for each global assembly metric were normalized, with 1 representing the best result and 0 the worst. The following assemblers are represented: GATBMiniaPipeline: dark blue, IDBA-UD: light blue, MEGAHIT: dark green, metaSPAdes: light green, SKESA: yellow, SPAdes: orange, Unicycler: red.

**Supplementary Fig. S6**. Assembler performance per reference for the ZymoBIOMICS Microbial Community Standards dataset for sample ERR2984773. The best score for each assembler was selected for 3 LMAS runs. The results for each global assembly metric were normalized, with 1 representing the best result and 0 the worst. The following assemblers are represented: GATBMiniaPipeline: dark blue, IDBA-UD: light blue, MEGAHIT: dark green, metaSPAdes: light green, SKESA: yellow, SPAdes: orange, Unicycler: red.

**Supplementary Fig. S7**. Genome fragmentation for each reference replicon of the ZimoBIOMICS community standards dataset for the logarithmically distributed samples. Genome fragmentation for the 3 LMAS runs is represented by the number of contigs and breadth of coverage of the reference per assembler for the logarithmically distributed samples: LNN (logarithmically distributed without error model, identified by a circle), LHS (logarithmically distributed with Illumina HiSeq error model, identified by a square), and ERR2935805 (real Illumina HiSeq sample, identified by a diamond). Each assembler is identified with the following color scheme—dark blue: Unicycler, light blue: SPAdes, dark green: SKESA, light green: metaSPAdes, yellow: MEGAHIT, orange: IDBA-UD, red: GATBMiniaPipeline.

**Supplementary Fig. S8**. Genome fragmentation for each reference replicon of the BMock12 community standards dataset sample. Genome fragmentation is represented by the number of contigs and breadth of coverage of the reference per assembler. Each assembler is identified with the following color scheme—dark blue: Unicycler, light blue: SPAdes, dark green: SKESA, light green: metaSPAdes, yellow: MEGAHIT, orange: IDBA-UD, red: GATBMiniaPipeline. Each reference replicon is identified by its IMG Taxon ID: 2615840527, *Muricauda* sp.; 2615840533, *Thioclava* sp.; 2615840601, *Cohaesibacter* sp.; 2615840646,*Propionibacteriaceae* bacterium; 2615840697, *Marinobacter* sp. LV10R510-8; 2616644829, *Marinobacter*. sp LV10MA510-1; 2617270709, *Psychrobacter* sp.; 2623620557, *Micromonospora echinaurantiaca*; 2623620567, *Micromonospora echinofusca*; 2623620609, *Micromonospora coxensis*; 2623620617, *Halomonas* sp. HL-4; and 2623620618, *Halomonas* sp. HL-93.

**Supplementary Fig. S9**. Location of gaps in comparison to the reference sequence, per assembler, for each reference replicon of the BMock12 community standards datasets. The resulting plot contains the gaps obtained for GATBMiniaPipeline, IDBA-UD, MEGAHIT, metaSPAdes, SKESA, SPAdes, and Unicycler assemblers.

**Supplementary Table S1**. Tools available for the *de novo* assembly of prokaryotic genomes. For each tool, its publication is indicated, if available, as well as the assembly algorithm implemented if it was developed explicitly to handle metagenomic datasets. The tools are ordered by the date of the last update, with the source code indicated when available. The tools incorporated in LMAS are indicated as such.

**Supplementary Table S2**. Comparison of metrics and features of LMAS with QUAST and MetaQUAST.

**Supplementary Table S3**. The ZymoBIOMICS Microbial Community Standards datasets. Set of raw sequence reads used as input in LMAS of mock communities with an even and logarithmic distribution of species, from real sequencing runs and simulated read datasets, with and without error, matching the intended distribution of species in ZymoBIOMICS Microbial Community Standards.

**Supplementary Table S4**. Microbial composition of the ZymoBIOMICS Microbial Community Standards dataset with even and logarithmic distribution of species. Theoretical microbial composition of the standards and the corresponding number of reads generated for each replicon.

**Supplementary Table S5**. Global quality metrics variation in 3 LMAS runs for sample ENN per assembler. The average calculated for all samples in the dataset for the 3 independent LMAS runs, followed by the minimum and maximum values obtained, is presented for each metric for each assembler.

**Supplementary Table S6**. Global quality metrics variation in 3 LMAS runs for sample EMS per assembler. The average calculated for all samples in the dataset for the 3 independent LMAS runs, followed by the minimum and maximum values obtained, is presented for each metric for each assembler.

**Supplementary Table S7**. Global quality metrics variation in 3 LMAS runs for sample ERR2984773 per assembler. The average calculated for all samples in the dataset for the 3 independent LMAS runs, followed by the minimum and maximum values obtained, is presented for each metric for each assembler.

**Supplementary Table S8**. Global quality metrics variation in 3 LMAS runs for sample LNN per assembler. The average calculated for all samples in the dataset for the 3 independent LMAS runs, followed by the minimum and maximum values obtained, is presented for each metric for each assembler.

**Supplementary Table S9**. Global quality metrics variation in 3 LMAS runs for sample LHS per assembler. The average calculated for all samples in the dataset for the 3 independent LMAS runs, followed by the minimum and maximum values obtained, is presented for each metric for each assembler.

**Supplementary Table S10**. Global quality metrics variation in 3 LMAS runs for sample ERR2935805 per assembler. The average calculated for all samples in the dataset for the 3 independent LMAS runs, followed by the minimum and maximum values obtained, is presented for each metric for each assembler.

**Supplementary Table S11**. Inconsistent contigs produced by the assemblers in 3 LMAS runs. For each assembler, the total number of contigs produced over the 3 runs of the LMAS workflow is indicated, as well as the contigs present in only 2 runs and a single run.

**Supplementary Table S12**. Global assembly metrics for single and multiple *k*-mer dBg assemblers. The median and the minimum and maximum values obtained are presented for each metric for all samples in 3 runs of LMAS. Single *k*-mer bBg assemblers: ABySS and minia. Multiple *k*-mer bBg assembler: GATBMiniaPipeline, IDBA-UD, MEGAHIT, MetaHipMer2, metaSPAdes, SKESA, SPAdes, Unicycler, and VelverOptimiser.

**Supplementary Table S13**. Global assembly metrics for genomic and metagenomic multiple *k*-mer dBg assemblers. The median and the minimum and maximum values obtained are presented for each metric for all samples in 3 runs of LMAS. Genomic assemblers: SKESA, SPAdes, and Unicycler. Metagenomic assemblers: GATBMiniaPipeline, IDBA-UD, MEGAHIT, and metaSPAdes.

**Supplementary Table S14**. Per reference quality metrics variation in 3 LMAS runs for sample ENN per assembler of the ZymoBIOMICS Microbial Community Standards dataset. The average calculated for all samples in the dataset for the 3 independent LMAS runs, followed by the minimum and maximum values obtained, is presented for each metric for each assembler.

**Supplementary Table S15**. Per reference quality metrics variation in 3 LMAS runs for sample EMS per assembler of the ZymoBIOMICS Microbial Community Standards dataset. The average calculated for all samples in the dataset for the 3 independent LMAS runs, followed by the minimum and maximum values obtained, is presented for each metric for each assembler.

**Supplementary Table S16**. Per reference quality metrics variation in 3 LMAS runs for sample ERR2984773 per assembler of the ZymoBIOMICS Microbial Community Standards dataset. The average calculated for all samples in the dataset for the 3 independent LMAS runs, followed by the minimum and maximum values obtained, is presented for each metric for each assembler.

**Supplementary Table S17**. Inconsistent gaps produced by the assemblers in 3 LMAS runs. For each assembler, the total number of gaps consistently produced in relation to the reference replicons over the 3 runs of the LMAS workflow is indicated, as well as gaps present in only 2 runs and a single run.

**Supplementary Table S18**. Annotation of consistent gaps produced by the assemblers in 3 LMAS runs.

**Supplementary Table S19**. Number of transfer RNA and ribosomal RNA coding sequencing, as well as mobile elements in ZymoBIOMICS Microbial Community Standards reference replicons. The average calculated for all samples in the dataset for the 3 independent LMAS runs, followed by the minimum and maximum values obtained, is presented for each metric for each assembler.

**Supplementary Table S20**. Taxonomic classification of the ZymoBIOMICS Microbial Community Standards dataset. The classification was performed with Kraken2, using the Standard Database. The results are presented as the percentage of classified reads for the 8 bacterial species in the community, as well as unclassified reads and the group of reads that are classified as species not contained in the community standard.

**Supplementary Table S21**. Global assembly metrics for dBg assemblers with single and multiple *k*-mer algorithms.

**Supplementary Table S22**. Reference-based quality metrics in 3 LMAS runs for the ZimoBIOMICS community standards dataset.

**Supplementary Table S23**. Pairwise comparisons of the ZymoBIOMICS Microbial Community Standards reference replicons. All pairwise comparisons among the set of genomes were conducted using Average Nucleotide Identity through BLAST as a proxy for DNA–DNA hybridization.

**Supplementary Table S24**. Microbial composition of the BMock12 microbial community standard dataset.

**Supplementary Table S25**. Pairwise comparisons of the BMock12 microbial community standard reference replicons.

**Supplementary Table S26**. Global quality metrics for the BMock12 sample SRX4901583 per assembler.

**Supplementary Table S27**. Per reference quality metrics for the BMock12 sample SRX4901583 per assembler.

**Supplementary Table S28**. Microbial composition of the Gut-Mix-RR microbial community standard dataset.

giac122_GIGA-D-22-00108_Original_Submission

giac122_GIGA-D-22-00108_Revision_1

giac122_Response_to_Reviewer_Comments_Original_Submission

giac122_Reviewer_1_Report_Original_Submission Adrian Fritz -- 6/7/2022 Reviewed

giac122_Reviewer_1_Report_Revision_1 Adrian Fritz -- 10/6/2022 Reviewed

giac122_Reviewer_2_Report_Original_Submission Rayan Chikhi -- 6/10/2022 Reviewed

giac122_Reviewer_2_Report_Revision_1 Rayan Chikhi -- 10/25/2022 Reviewed

giac122_Supplemental_Files

## Abbreviations

bp: base pair; cgMLST: core-genome multilocus sequence typing; dBg: de Bruijn graphs; FS: filtered set; GB: gigabytes; HPC: high-performance computing cluster; LMAS: Last Metagenomic Assembler Standing; LSA: longest single alignment; OLC: overlap-layout-consensus; Pls: Phred-like score; SNP: single-nucleotide polymorphism.

## Data Availability

The datasets analyzed during the current study are available in the Zenodo repository, under [48]. All supplemental material is available in the Zenodo repository, under [49]. Likewise, all figures in the current manuscript are available in their original format in the Zenodo repository, under [50]. Real sequencing data of the ZymoBIOMICS Microbial Community Standards are available under accessions ERR2984773 and ERR2935805 [38]. All data generated or analyzed during this study are included in this published article, its supplementary information files, and the data analysis repository located at [47]. Additionally, the reports for the ZymoBIOMICS Microbial Community Standards, BMock12 Community Standard, and NIBSC Gut DNA Reference are available at [51], [52], and [53], respectively.

An archival copy of the GitHub repository (https://github.com/B-UMMI/LMAS) is also available via the *GigaScience* database, GigaDB [54].

## Competing Interests

M.R. received honoraria for serving on the speakers' bureau of Pfizer and Merck Sharp and Dohme and for participating in expert panels of GlaxoSmithKline and Merck Sharp and Dohme. The other authors declare that they have no competing interests.

## Funding

Catarina Inês Mendes was supported by the Fundação para a Ciência e Tecnologia (grant SFRH/BD/129483/2017).

## Authors' Contributions

C.I.M. and M.R. designed the workflow. C.I.M. implemented and optimized the workflow, created the Docker containers, generated mock shotgun metagenomics data used to test and validate the workflow, contributed to the development of the HTML report, and analyzed the data. C.I.M. and M.R. wrote the manuscript. P.V.C. contributed to the development of the HTML report. M.R., J.A.C., Y.M., and J.M.G critically revised the manuscript. All authors read, commented on, and approved the final manuscript.
