# Supplementary material for: LMAS: evaluating metagenomic short de novo assembly methods through defined communities
Source: Gigascience. 2022 Dec 28;12:giac122. doi: 10.1093/gigascience/giac122 (PMC9795473; doi:10.1093/gigascience/giac122)
Supplement: giac122_GIGA-D-22-00108_Original_Submission [file giac122_giga-d-22-00108_original_submission.pdf]

GigaScience

LMAS: Last Metagenomic Assembler Standing

--Manuscript Draft--

**Manuscript Number:**	GIGA-D-22-00108
**Full Title:**	LMAS: Last Metagenomic Assembler Standing
**Article Type:**	Technical Note
**Funding Information:**	Fundação para a Ciência e Tecnologia (SFRH/BD/129483/2017) — Miss Catarina Inês Mendes
**Abstract:**	Background The de novo assembly of raw sequence data is key in metagenomic analysis. It allows recovering draft genomes from a pool of mixed raw reads, yielding longer sequences that offer contextual information and provide a more complete picture of the microbial community.<br>Findings To better compare de novo assemblers for metagenomic analysis, LMAS was developed as a flexible platform allowing users to evaluate assembler performance given known standard communities. Overall, in our test datasets, k-mer De Bruijn graph assemblers outperformed the alternative approaches but came with a greater computational cost. Furthermore, assemblers branded as metagenomic specific did not consistently outperform other genomic assemblers in metagenomic samples. Some assemblers still in use, such as ABySS, BCALM2, MetaHipmer2, minia and VelvetOptimiser, perform relatively poorly and should be used with caution when assembling complex samples.<br>Conclusions The choice of a de novo assembler depends on the computational resources available, the replicon of interest, and the major goals of the analysis. No single assembler appeared an ideal choice for short-read metagenomic prokaryote replicon assembly, each showing specific strengths. The choice of metagenomic assembler should be guided by user requirements and characteristics of the sample of interest, and LMAS provides an interactive evaluation platform for this purpose. LMAS is open source and the workflow and its documentation are available at https://github.com/B-UMMI/LMAS and https://lmas.readthedocs.io/ respectively.
**Corresponding Author:**	Catarina Inês Mendes, MSc<br>Universidade de Lisboa Faculdade de Medicina<br>Lisboa, Portugal PORTUGAL
**Corresponding Author Secondary Information:**	
**Corresponding Author's Institution:**	Universidade de Lisboa Faculdade de Medicina
**Corresponding Author's Secondary Institution:**	
**First Author:**	Catarina Inês Mendes, MSc
**First Author Secondary Information:**	
**Order of Authors:**	Catarina Inês Mendes, MSc
	Pedro Vila-Cerqueira
	Yair Motro
	Jacob Moran-Gilad
	João André Carriço
	Mário Ramirez
**Order of Authors Secondary Information:**	
**Additional Information:**	
**Question**	**Response**
Are you submitting this manuscript to a	No

Powered by Editorial Manager® and ProduXion Manager® from Aries Systems Corporation

special series or article collection?	
**Experimental design and statistics**<br><br>Full details of the experimental design and statistical methods used should be given in the Methods section, as detailed in our Minimum Standards Reporting Checklist. Information essential to interpreting the data presented should be made available in the figure legends.<br><br>Have you included all the information requested in your manuscript?	Yes
**Resources**<br><br>A description of all resources used, including antibodies, cell lines, animals and software tools, with enough information to allow them to be uniquely identified, should be included in the Methods section. Authors are strongly encouraged to cite Research Resource Identifiers (RRIDs) for antibodies, model organisms and tools, where possible.<br><br>Have you included the information requested as detailed in our Minimum Standards Reporting Checklist?	Yes
**Availability of data and materials**<br><br>All datasets and code on which the conclusions of the paper rely must be either included in your submission or deposited in publicly available repositories (where available and ethically appropriate), referencing such data using a unique identifier in the references and in the "Availability of Data and Materials" section of your manuscript.<br><br>Have you have met the above requirement as detailed in our Minimum Standards Reporting Checklist?	Yes

Powered by Editorial Manager® and ProduXion Manager® from Aries Systems Corporation

Powered by Editorial Manager® and ProduXion Manager® from Aries Systems Corporation

Manuscript Click here to access/download;Manuscript;LMAS Manuscript - GigaScience.docx

LMAS: Last Metagenomic Assembler Standing

C I Mendes*, Instituto de Microbiologia, Instituto de Medicina Molecular, Faculdade de Medicina, Universidade de Lisboa, Lisboa, Portugal, cimendes@medicina.ulisboa.pt, https://orcid.org/0000-0002-3090-7426

P Vila-Cerqueira, Instituto de Microbiologia, Instituto de Medicina Molecular, Faculdade de Medicina, Universidade de Lisboa, Lisboa, Portugal, pedro.cerqueira@medicina.ulisboa.pt, https://orcid.org/0000-0002-6121-8906

Y Motro, Faculty of Health Sciences, Ben-Gurion University of the Negev, Beer-Sheva, Israel, motroy@post.bgu.ac.il, https://orcid.org/0000-0003-1289-6919

J Moran-Gilad, Faculty of Health Sciences, Ben-Gurion University of the Negev, Beer-Sheva, Israel, giladko@post.bgu.ac.il, https://orcid.org/0000-0001-9134-050X

J A Carriço, Instituto de Microbiologia, Instituto de Medicina Molecular, Faculdade de Medicina, Universidade de Lisboa, Lisboa, Portugal, jcarrico@medicina.ulisboa.pt, https://orcid.org/0000-0002-5274-2722

M Ramirez, Instituto de Microbiologia, Instituto de Medicina Molecular, Faculdade de Medicina, Universidade de Lisboa, Lisboa, Portugal, ramirez@medicina.ulisboa.pt, https://orcid.org/0000-0002-4084-6233

*** corresponding author**

Abstract

Background The *de novo* assembly of raw sequence data is key in metagenomic analysis. It allows recovering draft genomes from a pool of mixed raw reads, yielding longer sequences

that offer contextual information and provide a more complete picture of the microbial community.

Findings To better compare *de novo* assemblers for metagenomic analysis, LMAS was developed as a flexible platform allowing users to evaluate assembler performance given known standard communities. Overall, in our test datasets, k-mer De Bruijn graph assemblers outperformed the alternative approaches but came with a greater computational cost. Furthermore, assemblers branded as metagenomic specific did not consistently outperform other genomic assemblers in metagenomic samples. Some assemblers still in use, such as ABySS, BCALM2, MetaHipmer2, minia and VelvetOptimiser, perform relatively poorly and should be used with caution when assembling complex samples.

Conclusions The choice of a *de novo* assembler depends on the computational resources available, the replicon of interest, and the major goals of the analysis. No single assembler appeared an ideal choice for short-read metagenomic prokaryote replicon assembly, each showing specific strengths. The choice of metagenomic assembler should be guided by user requirements and characteristics of the sample of interest, and LMAS provides an interactive evaluation platform for this purpose. LMAS is open source and the workflow and its documentation are available at https://github.com/B-UMMI/LMAS and https://lmas.readthedocs.io/ respectively.

Keywords

Shotgun Metagenomics, *de novo* assembly, benchmark, draft genome quality, simulation

Background

Short-read shotgun metagenomics has the potential to offer comprehensive microbial detection and characterisation of complex clinical or environmental samples. Despite becoming an increasingly used approach, it comes at the cost of producing massive amounts of data that require expert handling and processing, as well as adequate computational resources. The *de novo* assembly process is key when analysing metagenomic data since it allows recovering contigs representing the replicons present in the sample, be it genomes, plasmids or viruses, from a pool of mixed raw reads. These contigs are longer sequences that offer better contextual information than reads alone and provide a more complete picture of the microbial community than the species composition. Despite efforts for the development, standardization and assessment of software for metagenomic analysis, both commercial and open-source [1–5], the *de novo* assembly process still represents a critical point in these analyses.

The assembly of draft genomes has become a central step when analysing pure bacterial cultures, for instance allowing genomic comparisons through single nucleotide polymorphisms (SNPs) or gene-by-gene methods, such as core-genome multilocus sequence typing (cgMLST). The first assemblers implemented overlap-layout-consensus (OLC) approaches, comparing all reads in a sample, computing overlaps and generating consensus sequences by picking the most likely nucleotide for each position in the contigs. As the throughput of sequencing methods increased exponentially, so did the number of pairwise comparisons, limiting the efficiency of these algorithms and making them computationally too expensive. To circumvent this, De Bruijn graphs (dBg) algorithms were increasingly adopted and are currently the most widely used approaches in modern assembly software. Both OLC and dBg handle unresolvable repeats by essentially fragmenting the sequence, that is, forming multiple contigs

for each of the possibly contiguous sequences present in the sample. Additionally, the inherent heterogeneity of complex samples, potentially containing a multitude of replicons, could make traditional genome assemblers, implementing optimizations based on the assumption of having a single genome in the sample, not suitable for metagenomics [6].

Several dedicated metagenomic assembly tools for short-read data are available [6]. These tools are generally assumed to perform better when dealing with complex samples having a combination of intragenomic and intergenomic repeats and uneven and low coverage sequencing depths of some of the replicons [7]. Not using dedicated metagenomic assemblers was suggested to come with the cost of generating artificial variation and chimeric contigs, especially in samples that contain closely related species [8]. However, no formal comparison has been done looking at increased accuracy or gains in contiguity of assemblies obtained with metagenomic assemblers versus traditional assemblers.

With an ever-increasing range of both traditional and metagenomic assemblers becoming available, choosing the best performing tool can be an arduous and time-consuming task since the choice may vary depending on the purpose of the analysis, organism of interest, complexity of the sample and computational infrastructure available. Additionally, the evaluation of the resulting contigs is not straightforward since one metric is not sufficient to classify an assembly, particularly with complex samples [7,9]. Despite several *de novo* assembly validation methods relying on features of the created contigs themselves, such as QUAST [10], being useful in identifying inconsistencies indicative of potential assembly errors, the use of reference-based validation methods offer the possibility of a more complete evaluation of accuracy and are particularly important to benchmark attempts to reconstruct communities. MetaQUAST [9], a modification of QUAST, extends the original software by performing assembly evaluation based on aligning contigs to a reference, which can be provided or inferred by the software, and reports, in addition to the standard metrics for single genomes reported by QUAST, the number of interspecies translocations and the number of possibly misassembled contigs.

The use of mock communities, with known composition, abundance and genomic information, provides a ground truth against which the success of the assembly of a complex sample can be evaluated. Furthermore, this can be done in circumstances in which the errors introduced by sample processing and the sequencing and associated methods used approximate, as much as possible, their effects in real samples. Such mock communities facilitate the identification of misassemblies, such as chimeric sequences generated from the improper combination of two distinct replicons, indels or single nucleotide variants improperly created by the assembler. On the other hand, the comparison of the performance of two assemblers is only possible if the input data is the same and if the same evaluation metrics are applied [3].

To tackle these challenges, we developed LMAS (Last Metagenomic Assembler Standing), an automated workflow to enable the benchmarking of traditional and metagenomic prokaryotic *de novo* assembly software using defined mock communities. The results of LMAS are presented in an interactive HTML report where selected global and reference replicon specific performance metrics can be explored. The mock communities can be provided by the user to better reflect the samples of interest. New assemblers can be added with minimal changes to the pipeline so that LMAS can be expanded to include novel algorithms as they are developed. The portability and ease of use of LMAS are intended to allow users to easily evaluate the performance of assemblers in mock communities, mimicking as closely as possible their samples of interest.

The LMAS Workflow

Workflow overview

LMAS is a user-friendly automated workflow enabling the benchmarking of traditional and metagenomic prokaryotic *de novo* assembly software using defined mock communities. LMAS

was implemented in Nextflow [12] to provide flexibility and ensure the transparency and reproducibility of the results. LMAS relies on the use of Docker [13] containers for each assembler, allowing versions to be tracked and changed easily.

Figure 1: **The LMAS workflow.** The input sequencing data is assembled in parallel, resources permitting, by the set of assemblers included in LMAS. The resulting contigs are processed and the global quality assessment is performed. After filtering for the user-defined minimum contig size, the remaining sequences are mapped against the provided reference and the resulting information is processed to evaluate assembly quality by replicon in the reference file. All results, and optional text information describing the samples, are grouped in the LMAS report.

Installation and Usage

LMAS can be installed through Bioconda [14] or Github [15], with detailed instructions available in the documentation [16]. LMAS requires as inputs the complete reference replicons (genomes, plasmids or any other replicons present) and short-read paired-end raw data. All complete references (linear replicons) should be provided in a single file. This raw data can be either obtained *in silico* by creating simulated reads from the reference replicons or sequencing mock communities of known composition. Optionally, information on the input samples in a markdown file can be provided to be presented in the report.

A step-by-step execution tutorial is available at [17]. Users can customize the workflow execution either by using command-line options or by modifying the simple plain-text configuration files. To make the execution of the workflow as simple as possible, a set of default parameters and directives is provided. A complete description of each parameter is available in Supplemental Material (see Supplemental Material, Workflow parameters), as well as in the documentation [18]. The results are presented in an interactive HTML report, stored in the "*report*" folder in the directory of LMAS' execution. The output files of all assemblers and quality assessment processing scripts in the workflow are stored in the "*results*" folder, in the same location.

Supported Assemblers and selection criteria

A collection of *de novo* assembly tools was compiled, including OLC and dBg assembly algorithms, the latter including both single k-mer and multiple k-mer value approaches, and hybrid assemblers implementing both algorithms, including both genomic and metagenomic assemblers (Supplemental Table S1). Of these, 12 assemblers were selected based on the date of last update and are implemented in LMAS: ABySS [19] (version 2.3.1), BCALM2 [20] (version 2.2.3), GATB Minia Pipeline [21] (commit hash 9d56f42) , IDBA-UD [22] (version 1.1.3), MEGAHIT [23] (version 1.2.9), MetaHipMer2 [24] (version 2.0.0.65-gaad446d-dirty-AddGtest), metaSPAdes [25] (version 3.15.3), minia [26] (version 3.2.6), SKESA [27] (version 2.5.0), SPAdes [28] (version 3.15.3), Unicycler [29] (version 0.4.9) and VelvetOptimiser [30] (commit hash 092bdee) (Table 1). The execution commands for each assembler are available as Supplemental Material (see Supplemental Material, Short-read *de novo* assemblers) and in the documentation [31].

New assemblers can be added with minimal changes to the pipeline so that LMAS can be expanded as novel algorithms are developed. A template is available to facilitate their integration and a step-by-step guide is included in the documentation [32]. The only two requirements for the addition of a new assembler are the execution command for the assembler for paired-end short-read data and a Nextflow-compatible container with the assembler and any dependencies.

Table 1: Prokaryotic *de novo* assemblers integrated into LMAS.

Assembler	Type	Algorithm
**GATBMiniaPipeline**	Metagenomic	Multiple k-mer De Bruijn graph
**IDBA-UD**	Metagenomic	Multiple k-mer De Bruijn graph
**MEGAHIT**	Metagenomic	Multiple k-mer De Bruijn graph
**MetaHipMer2**	Metagenomic	Multiple k-mer De Bruijn graph

**metaSPAdes**	Metagenomic	Multiple k-mer De Bruijn graph
**ABySS**	Genomic	Single k-mer De Bruijn graph
**BCALM2**	Genomic	Single k-mer De Bruijn graph
**MINIA**	Genomic	Single k-mer De Bruijn graph
**SKESA**	Genomic	Multiple k-mer De Bruijn graph
**SPAdes**	Genomic	Multiple k-mer De Bruijn graph
**Unicycler**	Genomic	Multiple k-mer De Bruijn graph
**VelvetOptimizer**	Genomic	Multiple k-mer De Bruijn graph

Assembly Quality Metrics

The success of an assembly is evaluated in two steps: globally (see Global Metrics) and relative to each of the replicons present in the sample (see Per Reference Metrics). In both, the tabular presentation in the reports allows the comparison of exact values between assemblers, and the interactive plots allow a more intuitive overview and easy exploration of results. In addition to the assembly success metrics, computational resource statistics are registered for each assembler (see Supplemental Material, LMAS Metrics, Computational Performance Metrics).

Global Metrics

The computation of the global metrics is performed through statistics inherent to the complete set of contigs assembled per sample, independent of the species/sample of origin. The metrics are presented, in tabular form, for the complete set of contigs and those filtered for a minimum length, and also graphically for the contigs filtered for a minimum length. The statistics include information on contig number, size and ambiguous bases; and the proportion of reads mapping to the created contigs. Two statistics are a consolidation of per reference metrics: misassemblies (i.e. contigs that do not reflect the structural organization in the reference replicons); and the overall size of gaps in all reference replicons not covered by any contig. A

more detailed description of all global metrics is available in Supplemental Material (see Supplemental Material, LMAS Metrics, Global Metrics).

Per Reference Metrics

For the computation of the reference-based metrics, only the filtered set (FS) contigs are considered, for each reference replicon in the sample. These contigs are the ones exceeding the user-defined minimum sequence length, filtered using BBTools (version 38.44). After this initial step, the contigs are mapped to the reference replicons with minimap2 [33] (version 2.22). The metrics are computed through custom python code (see Supplemental Material, Assembly filtering and mapping) for each replicon in the file provided as input. A detailed description of all reference-based metrics is available in Supplemental Material (see Supplemental Material, LMAS Metrics, Per Reference Metrics).

In addition to the statistics shared with the global metrics, LMAS also calculates the number of mismatches relative to each reference, the COMPASS [21] metrics and two new metrics we propose: LSA and Pls.

LSA represents the fraction of the longest single alignment between a contig and the reference, relative to the reference length. The Pls, or Phred-like score, is a scoring function based on the identity of each aligned contig to the reference replicon. Similarly to the Phred quality score [34], a measure of the quality of the identification of the bases by sequencing, the Pls measures the quality of the assembly of a contig. The formula of Pls is similar to the Phred score formula but uses as the error function the identity of the base in the contig to that of the reference replicon. The formula to obtain the Pls metric per contig is Equation 1.

$$Phred(E) = \begin{cases} -\log(E) \times 10 & if\ E\ < 60 \\ 60 & if\ E = 0 \end{cases}$$

where $E = 1 - Identity$

Equation 1: Formula for the Pls.

The LMAS Report

The LMAS results are presented in an interactive HTML. The LMAS report is composed of two main panels: a top summary panel with information on input samples (provided by the user) and the resources used during LMAS' execution, and a bottom panel where selected global and reference specific assembly metrics can be explored for each sample. LMAS constructs the HTML file after workflow completion, storing it in the "reports" folder. The report data can be easily shared between users and requires only a browser for visualization.

Figure 2: **The LMAS report.** All results, and optional text information describing the samples, are grouped in the LMAS report, an interactive and responsive HTML file, for exploration in any browser. Links for LMAS source code and documentation are available in the top right corner of the report. 1) The summary panel of the LMAS report contains information on the input reference sequences and raw sequencing data samples (provided by the user), and the overall computational performance of the assemblers in LMAS. 2) The LMAS metric panel contains the explorable global and reference specific performance metrics per input raw sequencing data sample. The tabular presentation allows direct comparison of exact values between assemblies, and the interactive plots allow for an intuitive overview and easy exploration of results. 3) If an assembler fails to produce an assembly, or fails to assemble sequences that map to the reference replicon, it is marked in the table with a red warning sign. 4) The global or reference replicon specific metrics can be accessed for each sample in the dropdown menu.

Summary Panel

The top panel of the report contains information on the input samples and overall performance of the assemblers in LMAS, divided into three tabs: Overview, Performance and About us. On the top right corner of the report, direct links to LMAS' source repository and documentation are provided.

- *Overview*: This tab contains information on the input data, including the name and number of reads of the raw sequencing data, and the name of the reference file. Additional information provided by the user about the community used as input is also presented here.
- *Performance*: This tab contains a table with information on the version, the containers used and computational performance metrics for each assembler in LMAS.

- *About us*: This tab contains information on the LMAS GitHub repositories and the LMAS development team

Metrics Panel

The bottom portion of the report contains the explorable global and reference specific performance metrics per input raw sequencing data sample. Each sample has its own tab and the global or reference replicon specific metrics can be accessed in the dropdown menu.

Global Metrics

A table displays the global assembly metrics computed for the complete and FS contigs. If an assembler fails to produce an assembly, it is marked on the table with a red warning sign. The global metric plots are interactive, allow zooming in on particular areas and provide extra information as hover text boxes. The plots can be saved as PNG in whatever view the user selects.

Per Reference Metrics

Similarly to the global assembly metrics, a table displays the computed set of reference restricted metrics for the FS contigs. If an assembler fails to produce sequences that align to the reference, these are marked in the table with a red warning sign. Information on the expected reference replicon length and the GC content is calculated from the input files and reported above the table. The per-reference metric plots are also interactive, allowing the same type of operations as the global metric plots.

Comparison with other assembly evaluation software programs

The assessment and evaluation of genome assemblies has been a relevant field ever since the emergence of the assembly process itself, and therefore many solutions have been proposed [3,7,9–11,35–37]. The Critical Assessment of Metagenome Interpretation (CAMI) proposed a set of recommendations and best practices for benchmarking in microbiome

research [38]. These recommendations include the reporting of computational performance, which may condition the choice of software by the users, such as runtime, disk space and memory consumption, also reported by LMAS (see Supplementary Material, LMAS Metrics). As also suggested by CAMI, LMAS tracks the exact program version and command-line calls through its implementation in Nextflow. Moreover, using containerised assemblers and being easily installable through Bioconda, LMAS facilitates deployment in diverse user machines. Unlike the CAMI tutorial, in which users are asked to download and install the necessary tools, in LMAS everything is provided in a one-stop reproducible workflow that effortlessly handles all pre-processing, assembly, post-processing, traceability and report production steps, freeing users to focus on providing relevant samples for analysis and interpreting the results in view of the intended applications.

Concerning software for assembly quality assessment currently available, the most widely adopted is QUAST [10], or when dealing with metagenomic data, its extension metaQUAST [11], which was also adopted by the CAMI challenges [3,5] and suggested in the CAMI Tutorial [38]. Although several features of these tools overlap with LMAS' quality assessment components, these differ from LMAS in the sense that they are not a single step workflow allowing a traceable and reproducible assembly of mock communities. Unlike QUAST and metaQUAST, whose purpose is to evaluate assemblies, the purpose of LMAS is to allow users to evaluate assembler performance for a given sample of interest. Supplementary Table S2 shows the comparison of the output and computed assembly quality metrics generated by LMAS, QUAST and metaQUAST.

Results and Discussion

To illustrate the use of LMAS and evaluate the performance of the chosen assemblers we used the eight bacterial genomes and four plasmids of the ZymoBIOMICS Microbial Community Standards as reference. As input we used the raw sequence reads of mock

communities with an even and logarithmic distribution of species, from real sequencing runs [39] and simulated read datasets, with and without error, matching the distribution of species in each sample [40]. Our dataset is composed of samples ENN (*in silico* generated evenly distributed without error), EMS (*in silico* generated evenly distributed with Illumina MiSeq error model), ERR2984773 (evenly distributed real Illumina MiSeq sample), LNN (*in silico* generated logarithmically distributed without error), LHS (*in silico* generated logarithmically distributed with Illumina HiSeq error model) and ERR2935805 (logarithmically distributed real Illumina HiSeq sample) (see Supplemental Table S3). Detailed information about the generation of the input samples is available as Supplemental Material (see Supplemental Materials, ZymoBIOMICS microbial community standards, Supplemental Table S4). To evaluate the reproducibility of an assembler performance, the LMAS workflow was run three times for all samples using default parameters, and the resulting data was processed for each sample (see Supplemental Materials, Assessment of Assembly Success) Supplementary Table S5 to Table S10 present an overview of the average global performance per assembler for each sample in LMAS.

Some assemblers perform poorly

Of the 12 *de novo* prokaryotic assemblers included in LMAS, five stand out as having an overall poor performance: ABySS, BCALM2, MetaHipmer2, minia and VelvetOptimiser. Both ABySS and MetaHipmer2 performed inconsistently with differing resource requirements for the same sample in different runs, namely run time and memory allocation (see Supplemental Materials, Resource Requirements Differ Greatly, Supplemental Figure S2). Moreover, ABySS failed to produce an assembly for sample ERR2984773 for 1 of the runs (see Supplementary Table S7) and for sample LHS in any of the 3 runs in the time limit of 3 days (see Supplementary Table S9), and MetaHipmer2 failed to produce an assembly for samples LNN and LHS in all 3 runs (see Supplementary Tables S8-S9). VelvetOptimiser generated the highest number of inconsistent contigs across the 3 LMAS runs (Figure 3, Supplementary Table S11), with 1.69%

of the total contigs created present in only 1 or 2 runs. Although not as extreme as VelvetOptimiser, ABySS (0.52%), minia (0.14%), GATBMiniaPipeline (0.32%), MetaHipMer2 (0.11%) and IDBA-UD (0.08%) also showed inconsistencies in contig size.

Figure 3: Assembly robustness. Inconsistent contigs produced per assembler over 3 LMAS runs. The distribution of contig sizes, in basepairs, consistently present in all three LMAS runs are indicated in the grey boxplots for each assembler. If an assembler produced a contig only present in two of the runs (as determined by its size), its size is indicated in teal. If a contig is present in a single run, it is represented in red.

Regarding the quality assessment of the assemblies produced (Figure 4, Supplementary Table S12), ABySS, BCALM2 and minia are the only single k-mer dBg assemblers in the collection and were found to mostly underperform relative to their multiple k-mer dBg counterparts, generally resulting in more fragmented assemblies, although there were significant differences in performance across samples. Among multiple k-mer assemblers, VelvetOptimiser frequently produced a very high number of contigs of very small size (over 99% of the contigs not surpassing the minimum length of 1,000 bp) and therefore a low N50 (an average of 29,768 bp versus a global average of 84,114 bp) (Supplementary tables S5-S10). Additionally, ABySS and VelvetOptimizer produced contigs with a very large number of Ns, with an average of 1,019 and 3,035 uncalled bases per assembly, respectively. MetaHipMer2, although having overall average metrics in the two evenly distributed mock samples (ENN and EMS, Supplementary Tables S5-S6) where it was able to run successfully, it severely underperformed in the real samples (ERR2984773 and ERR2935805, Supplementary Tables S7 and S10). Generally, the performance scores of the assemblers decreased considerably for the real samples in comparison with the simulated ones, either with or without error. High utilization of the reads in the dataset is observed for most assemblers, with on average at least 90% of the reads mapping back to the assembly, except for ABySS, BCALM2, MetaHipMer2 and VelvetOptimiser whose values are in the range of 46-79%. Despite an overall good performance, SPAdes produced the highest number of misassembled contigs, with an average of 98 and a maximum of 572 (sample ERR2935805,

Supplementary Table S10), in comparison to the global average of 11 misassembled contigs for all assemblers across all samples.

Due to their poor performance discussed above, the following assemblers have not been included in subsequent analyses: ABySS, BCALM2, MetaHipmer2, minia and VelvetOptimiser.

Figure 4: Assembler performance for the ZymoBIOMICS Microbial Community Standards dataset. For each sample in the dataset, the best score of each assembler in the 3 LMAS runs was selected. The results for each global assembly metric was normalised, with 1 representing the best result, and 0 the worst. For the original assembly, the following metrics are presented: number of contigs produced (in blue), number of basepairs produced (in teal), the size of the largest contig assembled (in green), N50 (in yellow), percentage of mapped reads to the assembly (in orange) and uncalled bases (in red). For the filtered assembly, the additional metrics are presented: number of misassembled contigs (in purple) and number of misassembly events (in brown).

Metagenomic dedicated assemblers do not outperform genomic assemblers

After excluding the poorly performing assemblers, LMAS includes 3 genomic (SKESA, SPAdes and Unicycler) and 4 labelled as metagenomic specific (GATBMiniaPipeline, IDBA-UD, MEGAHIT and metaSPAdes) *de novo* prokaryotic assemblers, all implementing multiple k-mer dBg algorithms. As observed in Figure 5, Supplementary Table S13 and Supplemental Figure S3, there were very significant differences between the best and the worst performing assemblers of each type, with this difference being more pronounced for metagenomic assemblers. The best performing assemblers of each type behaved frequently quite similarly, and the differences between them tended to be attenuated after filtering for contigs <1 kbp. Still, for the linearly distributed samples (ENN, EMS and ERR2984773), the overall worst performers tended to be metagenomic assemblers. In contrast, for the logarithmically distributed samples (LNN, LHS and ERR2935805) the opposite was observed, with genomic assemblers tending to be the worst-performing (Figure 5). For the logarithmically distributed samples, the number of basepairs recovered is significantly lower than expected from their

composition for both genomic and metagenomic assemblers, particularly after filtering (Supplementary Table S13), as contigs representing the less abundant species are not recovered by either type of assemblers (see Assembler performance is influenced by replicon abundance in the sample). For this dataset, the fact that an assembler is branded as genomic or metagenomic does not translate into better or worse performance in dealing with these complex samples, but rather characteristics of the individual assemblers themselves determine their performance.

Figure 5: Performance of genomic and metagenomic assemblers for the ZymoBIOMICS Microbial Community Standards dataset. For each sample in the dataset and for the 3 runs, the best and worst scores for each assembler category were selected: genomic (in blue) and metagenomic (in red). The results for each global assembly metric were normalised, with 1 representing the best result, and 0 the worst. For the original assembly, the following metrics are presented: number of contigs produced, number of basepairs produced, the size of the largest contig assembled, N50, percentage of mapped reads to the assembly and uncalled bases. For the filtered assembly, the additional metrics are presented: number of misassembled contigs and number of misassembly events.

Success is not straightforward

Several factors contribute to suboptimal performance of the assembly process, from DNA isolation and library preparation protocol; sequencing technology, depth and read length; to possible contamination and inherent characteristics of the sample composition.

Assembler performance is influenced by species

For the eight bacterial genomes present in the samples, even in those with an even distribution of the genomes (ENN, EMS and ERR2984773), variations in the assembly metrics were observed (Figure 6, Supplemental Figures S4-S6, Supplemental Tables S14-S16). For all samples in the dataset, the genomes are recovered almost completely, with all replicons being >90% represented in the resulting assemblies. *Lactobacillus fermentum* is the least represented genome (92.2%-94.9%). Most replicon sequences are recovered in <100 contigs, except for *Pseudomonas aeruginosa*, *Escherichia coli* and *Salmonella enterica*, and not considering IDBA-UB, which frequently produces a larger number of contigs when compared

to other assemblers. The absolute values of other metrics of assembly quality, such as LSA, misassembly events or uncalled bases, are also different between bacterial genomes (Supplemental Tables S14-S16). The fact that *S. enterica* is a closely related species to *E. coli*, with high level of genetic similarity (ANIb >0.8, Supplemental Table S23), could have created difficulties for resolving the assemblies in a mixed sample and lead to the lower coverage observed, the higher number of contigs and the increased number of misassembled contigs identified in these species in some samples. However, in the case of the larger number of contigs of *P. aeruginosa*, no related species are present in the sample and these possibly reflect intrinsic properties of the replicon. Similarly, replicon characteristics could be behind the lower breadth of coverage consistently observed in *L. fermentum* assemblies.

Figure 6: Genome fragmentation for each reference replicon of the ZimoBIOMICS community standards dataset for the evenly distributed samples. Genome fragmentation for the 3 LMAS runs is represented by the number of contigs and breadth of coverage of the reference per assembler for the evenly distributed samples: ENN (evenly distributed without error model, identified by a circle), EMS (evenly distributed with Illumina MiSeq error model, identified by a square) and ERR2984773 (real Illumina MiSeq sample, identified by a diamond). Each assembler is identified with the following colour scheme - dark blue: Unicycler, light blue: SPAdes, dark green: SKESA, light green: metaSPAdes, yellow: MEGAHIT, orange: IDBA-UD, red: GATBMiniaPipeline.

Longer contigs have higher confidence

The Pls metric, which measures the error rate of a contig relative to the reference, shows that for every replicon, longer contigs have higher Pls (Figure 7). This could justify the option of filtering an assembly by length, even beyond the 1000 bp minimum contig size implemented by default in LMAS. Not only are we eliminating shorter, less informative contigs in terms of genetic context, but these are also the ones most likely to contain errors relative to the reference sequence.

Figure 7: Phred-like score (Pls) per contig for each reference replicon of the ZimoBIOMICS community standards datasets. The Pls score was calculated for each unique contig produced by each assembler in 3 LMAS runs and is represented in relation to its contig size. Each contig is coloured according to the assembler with the following colour scheme - dark blue: Unicycler, light blue: SPAdes, dark green: SKESA, light green: metaSPAdes, yellow: MEGAHIT, orange: IDBA-UD, red: GATBMiniaPipeline.

Certain genomic regions are problematic for all assemblers

Some genomic regions in several replicons are consistently a challenge for all assemblers. As observed in Figure 8, all genomes present certain regions that fail to assemble for all tools in all runs, even those generating high-quality draft assemblies. Of all seven assemblers considered, only GATBMiniaPipeline, MEGAHIT and IDBA-UD showed inconsistency in the gaps produced over the 3 LMAS runs (Supplemental Table S17), as expected from producing variable sets of contigs. The regions consistently missing for all assemblers in all runs are rich in repetitive elements, such as rRNA and tRNA coding sequences and mobile genetic elements (Supplemental Table S18), with larger gaps corresponding to tandem sets of these elements. This reflects an intrinsic limitation of short-read sequencing since the length of a read pair is not enough to bridge across the repetitive element, preventing the generation of contigs representing these regions. This is something that could be addressed by the use of long-read sequencing technologies. Despite this, some assemblers are able to produce contigs that represent some of these large tandem regions, such as MEGAHIT and SKESA for *E. faecalis,* and IDBA-UD, MEGAHIT and metaSPADES for *L. monocytogenes*, but such performance is not consistent for all reference replicons. For instance, SKESA fails to assemble two large regions of the *S. enterica* genome that all other assemblers successfully cover.

Figure 8: Location of gaps in comparison to the reference sequence, per assembler, for each reference replicon of the ZimoBIOMICS community standards datasets. The resulting plot contains the consistent gaps obtained from a three LMAS run for the evenly distributed dataset (ENN, EMS and ERR2984773) for GATBMiniaPipeline, IDBA-UD, MEGAHIT, metaSPAdes, SKESA, SPAdes and Unicycler assemblers.

Assembler performance is influenced by replicon abundance in the sample

The logarithmically distributed samples (LNN, LHS and ERR2935805) showed greater variation in the assembly success metrics than the evenly distributed samples (Supplementary Table S8-S10), reflecting the difficulty of recovering sequences of the lowest abundant replicons. For the three replicons with an estimated depth of coverage >15x, a similar pattern is observed in logarithmically distributed samples as in evenly distributed samples, albeit with greater dispersion in the number of contigs generated and with a markedly decreased breath of coverage for some assemblers and samples in the logarithmically distributed samples (Figure 6 and Supplementary Figure S7). Almost no contigs >1000 bp were retrieved for replicons with an estimated depth of coverage of <2x resulting in a very low breadth of coverage (<1%) (Supplementary Table S4, Supplementary Table S22). This leads to a severe underrepresentation of the diversity of the community in the generated contigs, particularly of plasmid sequences due to their smaller length and abundance. This happens despite the greater sequencing depth of these samples versus those with an even distribution (>5-fold difference in the number of reads).

Conclusions

The purpose of LMAS is to empower users to test assembler performance in meaningful conditions for their experimental setup and objectives. Suitable mock communities, reproducing the users' samples of interest, can be used as a gold standard to evaluate assembler performance. To illustrate LMAS' functionalities we analysed a well-known sample used in several studies. Although the eight species ZymoBIOMICS Microbial Community Standards might not be representative of the metagenomic complexity of the samples of interest of most researchers, its relative simplicity means that the results shown probably represent a best-case scenario, since as sample complexity increases so do the challenges

to assembler performance. Our results showed significant differences in both global and reference-dependent assembly quality metrics generated by each *de novo* assembler. The performance of each assembler varied depending on the species of interest and its abundance in the sample, with less abundant species presenting a significant challenge for all assemblers. The fact that an assembler is branded as specific for metagenomics does not guarantee a better performance in metagenomic samples, with assemblers used for genomic assembly outperforming the worst metagenomic assembler tested. The following assemblers showed significant performance problems and their usability may be limited, at least with the default parameters we used: ABySS, BCALM2, MetaHipmer2, minia and VelvetOptimiser.

The choice of de novo assembler depends greatly on the computational resources available, the species of interest, and, possibly, the composition of the community in the sample. In our testing with the ZymoBIOMICS community, no assembler stood out as an undisputed all-purpose choice for short-read metagenomic prokaryotic genome assembly, with different assemblers showing specific strengths. Users would thus benefit from analysing the results of sequencing mock communities or of artificially generated reads simulating their samples of interest to guide their choice of assembler. LMAS was developed to be an easy to use and flexible tool for this purpose. From the results that we obtained with the ZymoBIOMICS dataset, the following assemblers performed consistently well (presented in alphabetical order): MEGAHIT, metaSPAdes, SKESA, SPAdes and Unicycler. From our assessment, we conclude that these assemblers are the most likely candidates to perform well in other complex samples.

LMAS was built with modularity and containerization as keystones, leveraging the parallelization of processes and guaranteeing reproducibility across platforms. The modular design allows for new assemblers to be easily added and existing assemblers to be easily updated, ensuring its future relevance as improvements in assembly software are proposed, and evaluating the gains of such cumulative improvements using the same benchmark set adapted to a specific project or goal. Such reproducibility, capacity to easily add assemblers

of interest not included in the current version and flexibility for future extensions are important principles in computational method benchmarking. Moreover, users may compare software performance against mock communities of special interest, depending on their operational focus.

The interactive report provides an intuitive platform for data exploration, allowing the user to easily sift through global and reference specific performance metrics for each sample, as well as providing information on the assemblers executed to allow traceability of the results. Producing an extensive, metric rich report allows users interested in different aspects of assembler performance to make informed decisions, particularly when choosing among the top-performing assemblers, which show only minor differences.

LMAS applies several well-known assembly metrics and proposes two more: LSA, which represents the fraction of the longest single alignment between a contig and the reference, and Pls, a scoring function based on the identity of each aligned contig to the reference replicon. The entire set of assembly quality metrics used in LMAS allows not only the assessment of quality based on statistics inherent to a set of assembled contigs but also a comparison to a ground truth provided through the use of samples of known composition and reference sequences. The LMAS report provides an interactive and intuitive platform for the exploration of these results, allowing users to easily test assemblers in mock samples with species composition and distribution relevant for their own studies.

Although computationally intensive due to the complex nature of the *de novo* assembly process, LMAS is the only software integrating assembly and its evaluation into a single pipeline, guaranteeing the same conditions are met for all tools. With LMAS, it is now possible to evaluate which *de novo* assembler produces the most relevant results for a given community of interest. The LMAS workflow is open-source and its code and documentation are available at https://github.com/B-UMMI/LMAS and https://lmas.readthedocs.io/ respectively.

Availability of supporting source code and requirements

Project name: LMAS

Project home page: https://github.com/B-UMMI/LMAS

Operating system(s): UNIX-like systems.

Programming languages: Nextflow, Python, Bash, Javascript

Other requirements: Java version 8 or highest. Docker/Singularity/Shifter

License: GNU GPL v3

RRID: SCR_022251

List of abbreviations

Bp - Basepairs

cgMLST - core-genome multilocus sequence typing

dBg - de Bruijn graphs

FS - Filtered set

GB - gigabytes

HPCs - high-performance computing clusters

LSA - Longest single alignment

OLC - overlap-layout-consensus

Pls - Phred-like score

SNPs - single nucleotide polymorphisms

Declarations

Ethics approval and consent to participate

Not applicable.

Consent for publication

Not applicable.

Availability of data and material

The datasets analysed during the current study are available in the Zenodo repository, under https://doi.org/10.5281/zenodo.4588969. Real sequencing data of the ZymoBIOMICS Microbial Community Standards is available under accessions ERR2984773 and ERR2935805 [39]. All data generated or analysed during this study are included in this published article, its supplementary information files and the data analysis repository located at [41].

Competing interests

MR received honoraria for serving on the speakers' bureau of Pfizer and for consulting for GlaxoSmithKline and Merck Sharp and Dohme. The other authors declare that they have no competing interests.

Funding

C.I.M. was supported by the Fundação para a Ciência e Tecnologia (grant SFRH/BD/129483/2017).

Author's contributions

C.I.M., M.R. designed the workflow. C.I.M implemented and optimised the workflow, created the Docker containers, generated mock shotgun metagenomics data used to test and validate the workflow, contributed to the development of the HTML report and analysed the data. C.I.M. and M.R. wrote the manuscript. P.V.C. contributed to the development of the HTML report. M.R., J.A.C. Y.M, and J.M.G critically revised the manuscript. All authors read, commented on, and approved the final manuscript.

Acknowledgements

The authors would like to thank Rafael Mamede for his contribution to the implementation and commentary on the several interactive plots implemented throughout the LMAS report. The authors would also like to thank Nabil Fareed-Alikan for his insightful commentary on the interpretation of the results reported in this manuscript, and Anthony Underwood and Robert A. Petit III for their assistance in building the LMAS Nextflow workflow. The author would also like to thank Samuel Nicholls, Joshua Quick, Shuiquan Tang and Nicholas Loman for publicly providing the sequencing data for the ZymoBIOMICS Microbial Community Standards.

References

1. Angers-Loustau A, Petrillo M, Bengtsson-Palme J, Berendonk T, Blais B, Chan K-G, et al.. The challenges of designing a benchmark strategy for bioinformatics pipelines in the identification of antimicrobial resistance determinants using next generation sequencing technologies. *F1000Research*. 2018; doi: 10.12688/f1000research.14509.2.
2. Gruening B, Sallou O, Moreno P, da Veiga Leprevost F, Ménager H, Søndergaard D, et al.. Recommendations for the packaging and containerizing of bioinformatics software. *F1000Research*. 2019; doi: 10.12688/f1000research.15140.2.
3. Sczyrba A, Hofmann P, Belmann P, Koslicki D, Janssen S, Dröge J, et al.. Critical Assessment of Metagenome Interpretation—a benchmark of metagenomics software. *Nat Methods*. 2017; doi: 10.1038/nmeth.4458.
4. Couto N, Schuele L, Raangs EC, Machado MP, Mendes CI, Jesus TF, et al.. Critical steps in clinical shotgun metagenomics for the concomitant detection and typing of microbial pathogens. *Sci Rep*. 2018; doi: 10.1038/s41598-018-31873-w.
5. Meyer F, Fritz A, Deng Z-L, Koslicki D, Gurevich A, Robertson G, et al.. Critical Assessment of Metagenome Interpretation - the second round of challenges. Bioinformatics; 2021 Jul.
6. Ayling M, Clark MD, Leggett RM. New approaches for metagenome assembly with short reads. *Brief Bioinform*. 2020; doi: 10.1093/bib/bbz020.
7. Olson ND, Treangen TJ, Hill CM, Cepeda-Espinoza V, Ghurye J, Koren S, et al.. Metagenomic assembly through the lens of validation: recent advances in assessing and improving the quality of genomes assembled from metagenomes. *Brief Bioinform*. 2019; doi: 10.1093/bib/bbx098.
8. Teeling H, Glockner FO. Current opportunities and challenges in microbial metagenome analysis--a bioinformatic perspective. *Brief Bioinform*. 2012; doi: 10.1093/bib/bbs039.
9. Bradnam KR, Fass JN, Alexandrov A, Baranay P, Bechner M, Birol I, et al.. Assemblathon 2: evaluating de novo methods of genome assembly in three vertebrate species. *GigaScience*. 2013; doi: 10.1186/2047-217X-2-10.
10. Gurevich A, Saveliev V, Vyahhi N, Tesler G. QUAST: quality assessment tool for genome assemblies. *Bioinformatics*. 2013; doi: 10.1093/bioinformatics/btt086.
11. Mikheenko A, Saveliev V, Gurevich A. MetaQUAST: evaluation of metagenome assemblies. *Bioinformatics*. 2016; doi: 10.1093/bioinformatics/btv697.
12. Di Tommaso P, Chatzou M, Floden EW, Barja PP, Palumbo E, Notredame C. Nextflow enables reproducible computational workflows. *Nat Biotechnol*. 2017; doi: 10.1038/nbt.3820.
13. Merkel D. Docker: Lightweight Linux Containers for Consistent Development and Deployment. *Linux J*. Houston, TX: Belltown Media; 20142014;
14. : Lmas :: Anaconda.org. https://anaconda.org/bioconda/lmas Accessed 2022 Apr 6.
15. Mendes I, Vila-Cerqueira P, Ramirez M: LMAS: Last (Meta)genomic Assembler Standing. https://github.com/B-UMMI/LMAS (2021). Accessed 2022 Apr 4.
16. : Installation — LMAS 0.1 documentation. https://lmas.readthedocs.io/en/latest/getting_started/installation.html Accessed 2022 Apr 4.
17. : Basic Usage — LMAS 0.1 documentation. https://lmas.readthedocs.io/en/latest/user/basic_usage.html Accessed 2022 Apr 4.
18. : Parameters — LMAS 0.1 documentation. https://lmas.readthedocs.io/en/latest/user/parameters.html Accessed 2022 Apr 4.
19. Jackman SD, Vandervalk BP, Mohamadi H, Chu J, Yeo S, Hammond SA, et al.. ABySS 2.0: resource-efficient assembly of large genomes using a Bloom filter. *Genome Res*. 2017; doi: 10.1101/gr.214346.116.
20. Chikhi R, Limasset A, Medvedev P. Compacting de Bruijn graphs from sequencing data quickly and in low memory. *Bioinformatics*. 2016; doi: 10.1093/bioinformatics/btw279.
21. : GATB/gatb-minia-pipeline. https://github.com/GATB/gatb-minia-pipeline (2022). Accessed 2022 Apr 4.

22. Peng Y, Leung HCM, Yiu SM, Chin FYL. IDBA-UD: a de novo assembler for single-cell and metagenomic sequencing data with highly uneven depth. *Bioinformatics*. 2012; doi: 10.1093/bioinformatics/bts174.
23. Li D, Liu C-M, Luo R, Sadakane K, Lam T-W. MEGAHIT: an ultra-fast single-node solution for large and complex metagenomics assembly via succinct de Bruijn graph. *Bioinformatics*. 2015; doi: 10.1093/bioinformatics/btv033.
24. Georganas E, Egan R, Hofmeyr S, Goltsman E, Arndt B, Tritt A, et al.. Extreme Scale De Novo Metagenome Assembly. *SC18 Int Conf High Perform Comput Netw Storage Anal*. Dallas, TX, USA: IEEE;
25. Nurk S, Meleshko D, Korobeynikov A, Pevzner PA. metaSPAdes: a new versatile metagenomic assembler. *Genome Res*. 2017; doi: 10.1101/gr.213959.116.
26. Chikhi R, Rizk G. Space-efficient and exact de Bruijn graph representation based on a Bloom filter. *Algorithms Mol Biol*. 2013; doi: 10.1186/1748-7188-8-22.
27. Souvorov A, Agarwala R, Lipman DJ. SKESA: strategic k-mer extension for scrupulous assemblies. *Genome Biol*. 2018; doi: 10.1186/s13059-018-1540-z.
28. Bankevich A, Nurk S, Antipov D, Gurevich AA, Dvorkin M, Kulikov AS, et al.. SPAdes: A New Genome Assembly Algorithm and Its Applications to Single-Cell Sequencing. *J Comput Biol*. 2012; doi: 10.1089/cmb.2012.0021.
29. Wick RR, Judd LM, Gorrie CL, Holt KE. Unicycler: Resolving bacterial genome assemblies from short and long sequencing reads. Phillippy AM, editor. *PLOS Comput Biol*. 2017; doi: 10.1371/journal.pcbi.1005595.
30. Seemann T: VelvetOptimiser: automate your Velvet assemblies. https://github.com/tseemann/VelvetOptimiser (2021). Accessed 2022 Apr 4.
31. : Short-Read (Meta)Genomic Assemblers — LMAS 0.1 documentation. https://lmas.readthedocs.io/en/latest/user/assemblers.html Accessed 2022 Apr 4.
32. : Add Assembler Process — LMAS 0.1 documentation. https://lmas.readthedocs.io/en/latest/dev/add_process.html Accessed 2022 Apr 4.
33. Li H. Minimap2: pairwise alignment for nucleotide sequences. Birol I, editor. *Bioinformatics*. 2018; doi: 10.1093/bioinformatics/bty191.
34. Ewing B, Hillier L, Wendl MC, Green P. Base-Calling of Automated Sequencer Traces Using *Phred.* I. Accuracy Assessment. *Genome Res*. 1998; doi: 10.1101/gr.8.3.175.
35. Manchanda N, Portwood JL, Woodhouse MR, Seetharam AS, Lawrence-Dill CJ, Andorf CM, et al.. GenomeQC: a quality assessment tool for genome assemblies and gene structure annotations. *BMC Genomics*. 2020; doi: 10.1186/s12864-020-6568-2.
36. Meader S, Hillier LW, Locke D, Ponting CP, Lunter G. Genome assembly quality: assessment and improvement using the neutral indel model. *Genome Res*. 2010; doi: 10.1101/gr.096966.109.
37. Challis R, Richards E, Rajan J, Cochrane G, Blaxter M. BlobToolKit – Interactive Quality Assessment of Genome Assemblies. *G3 GenesGenomesGenetics*. 2020; doi: 10.1534/g3.119.400908.
38. Meyer F, Lesker T-R, Koslicki D, Fritz A, Gurevich A, Darling AE, et al.. Tutorial: assessing metagenomics software with the CAMI benchmarking toolkit. *Nat Protoc*. Nature Publishing Group; 2021; doi: 10.1038/s41596-020-00480-3.
39. Nicholls SM, Quick JC, Tang S, Loman NJ. Ultra-deep, long-read nanopore sequencing of mock microbial community standards. *GigaScience*. 2019; doi: 10.1093/gigascience/giz043.
40. Gourlé H, Karlsson-Lindsjö O, Hayer J, Bongcam-Rudloff E. Simulating Illumina metagenomic data with InSilicoSeq. Hancock J, editor. *Bioinformatics*. 2019; doi: 10.1093/bioinformatics/bty630.
41. : LMAS Manuscript Analysis. https://github.com/B-UMMI/LMAS_Manuscript_Analysis (2022). Accessed 2022 Apr 4.

Figure 1

Click here to access/download;Figure;Figure 1.png

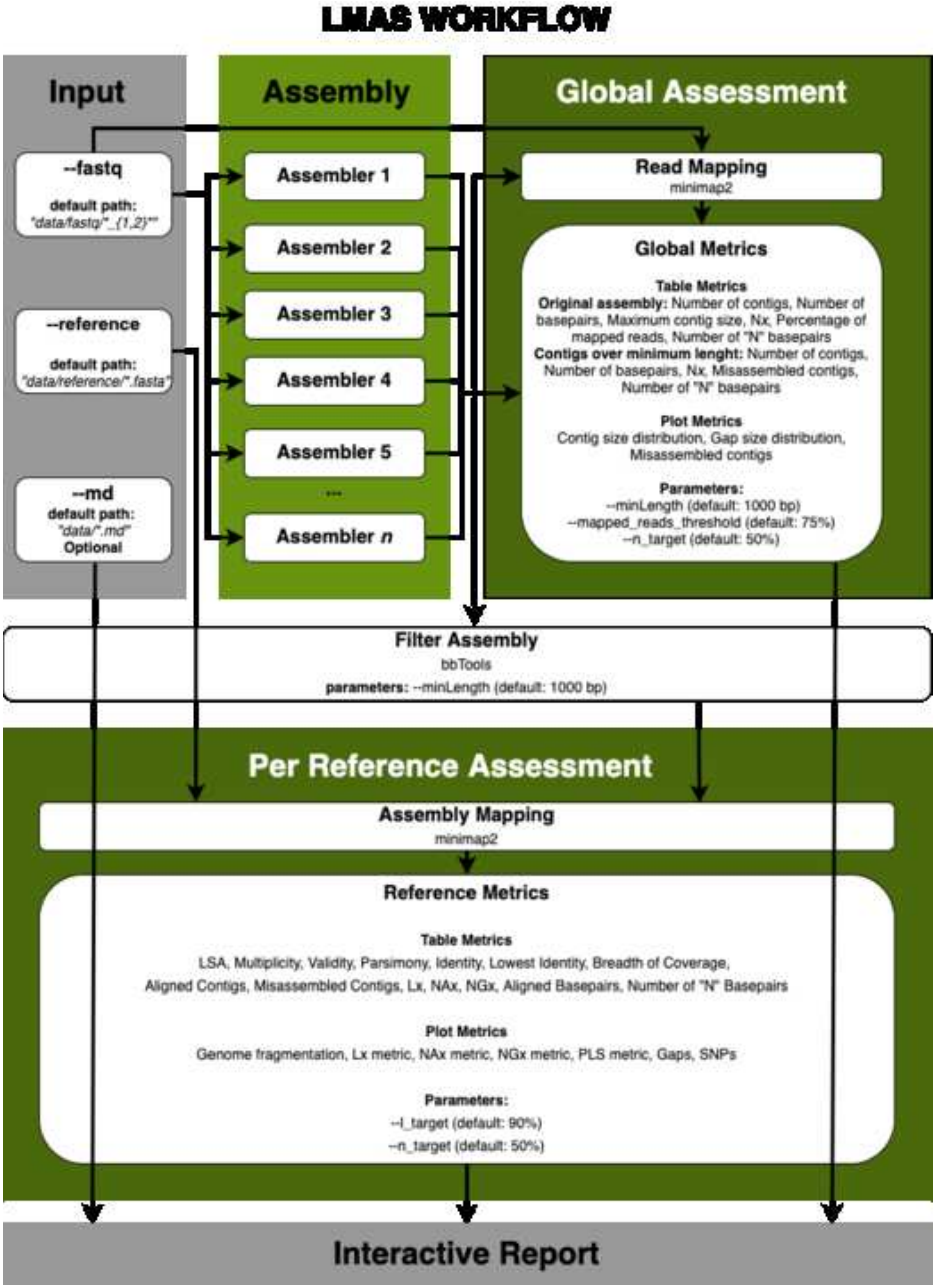

Figure 2

Click here to access/download;Figure;Figure 2.png

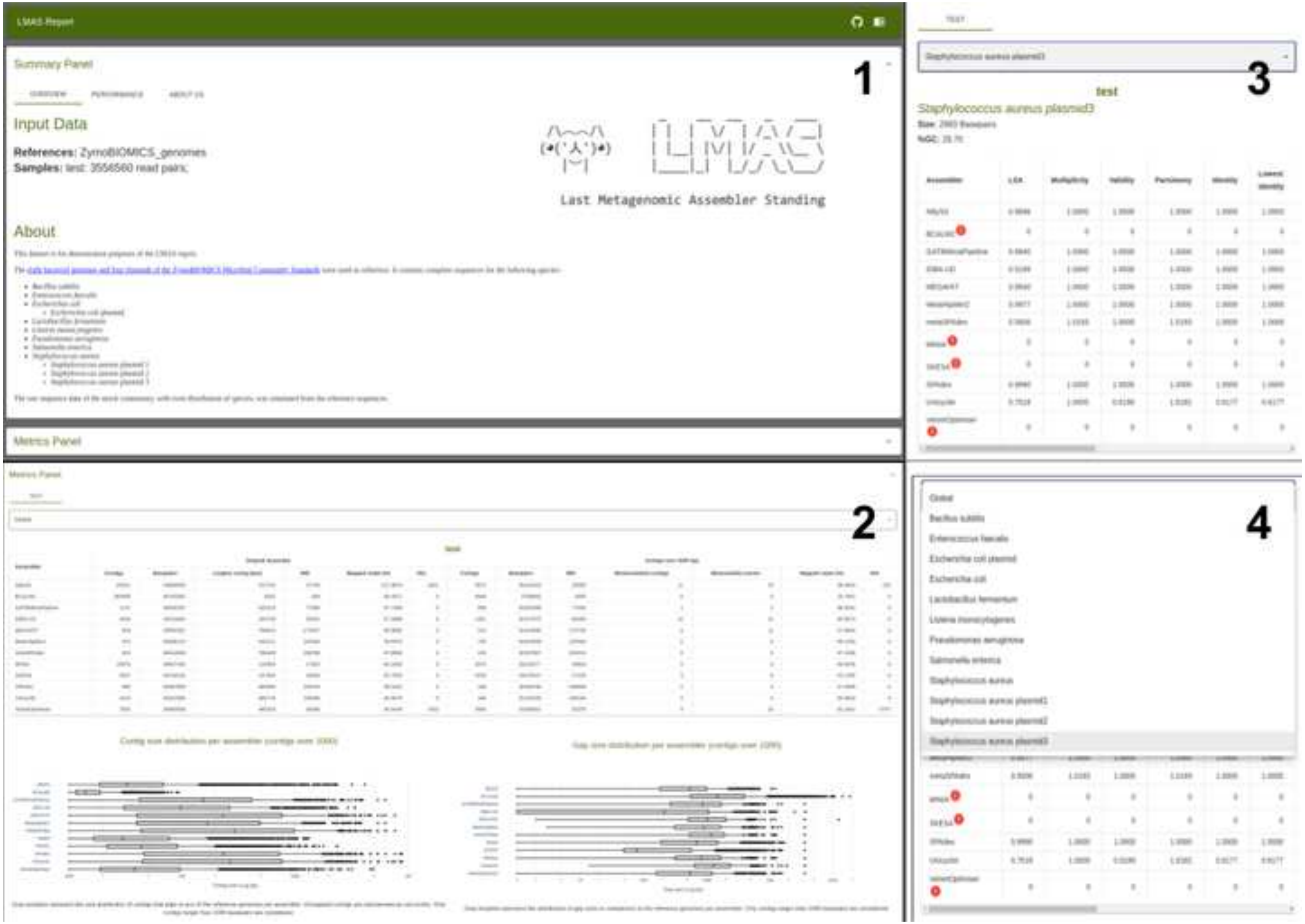

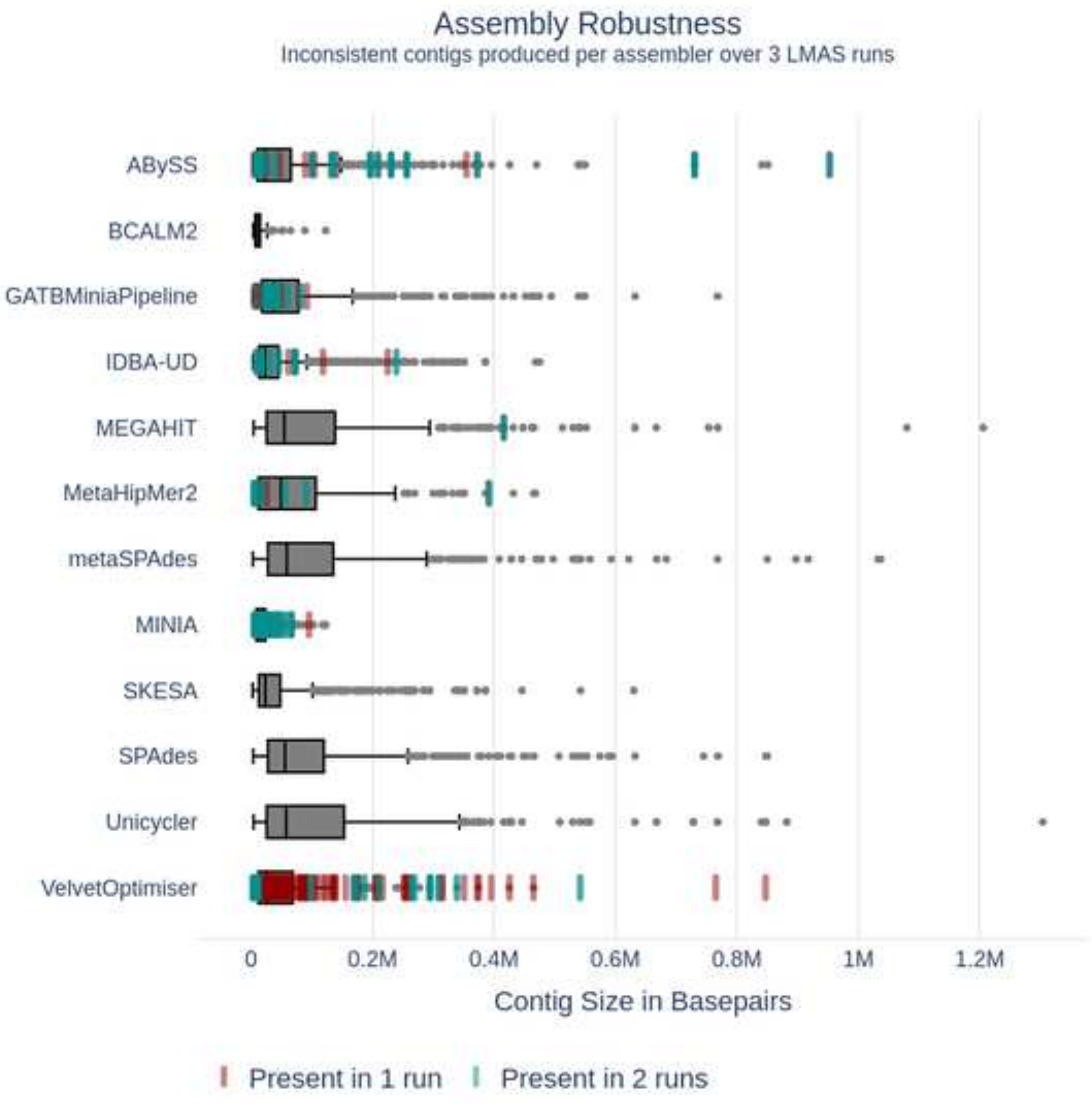
Assembly Robustness
Inconsistent contigs produced per assembler over 3 LMAS runs
ABySS
BCALM2
GATBMiniaPipeline
IDBA-UD
MEGAHIT
MetaHipMer2
metaSPAdes
MINIA
SKESA
SPAdes
Unicycler
VelvetOptimiser
0
0.2M
0.4M
0.6M
0.8M
1M
1.2M
Contig Size in Basepairs
Present in 1 run
Present in 2 runs

Figure 4

Click here to access/download;Figure;Figure 4.png

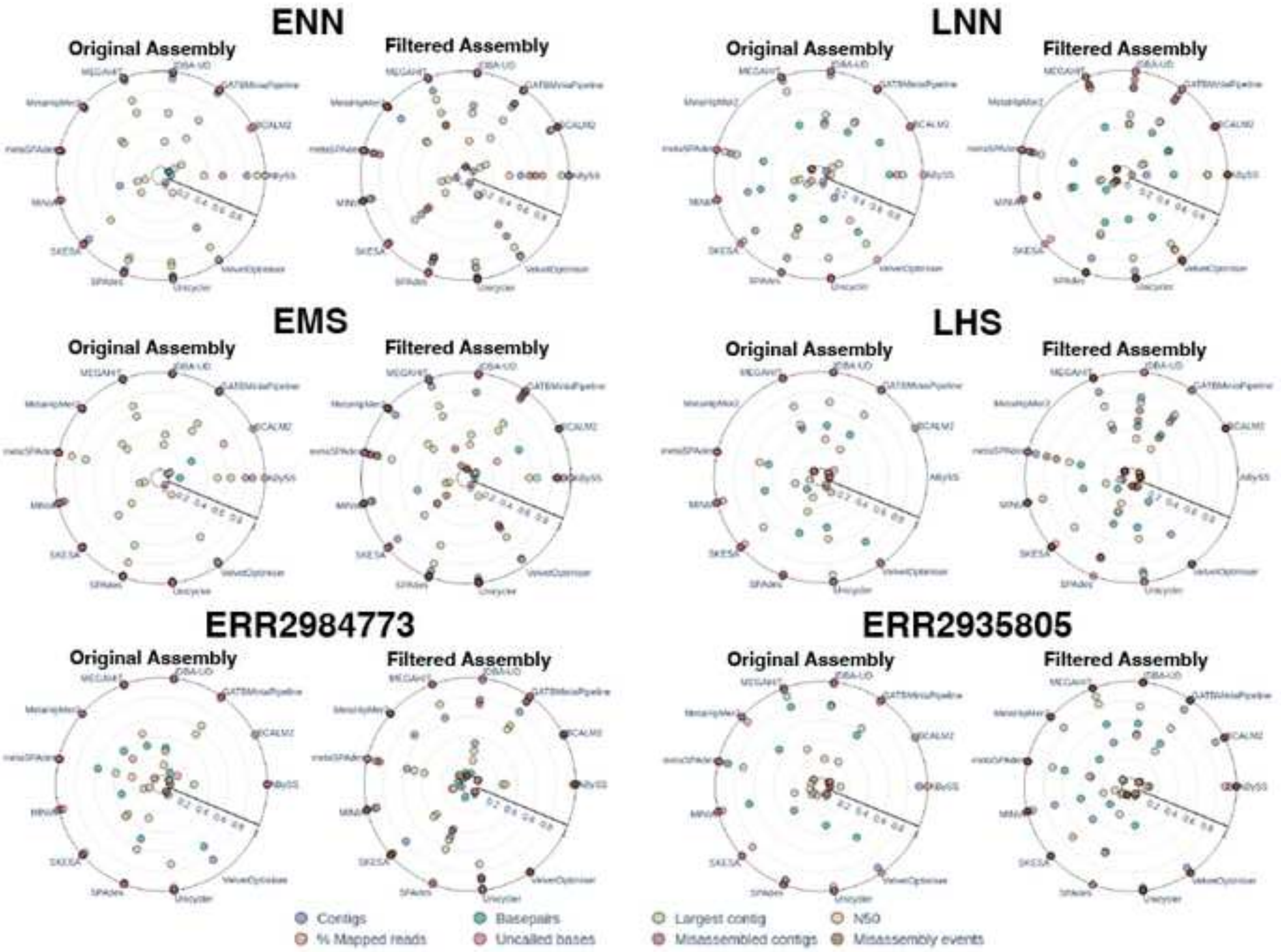

Figure 5

Click here to access/download;Figure;Figure 5.png

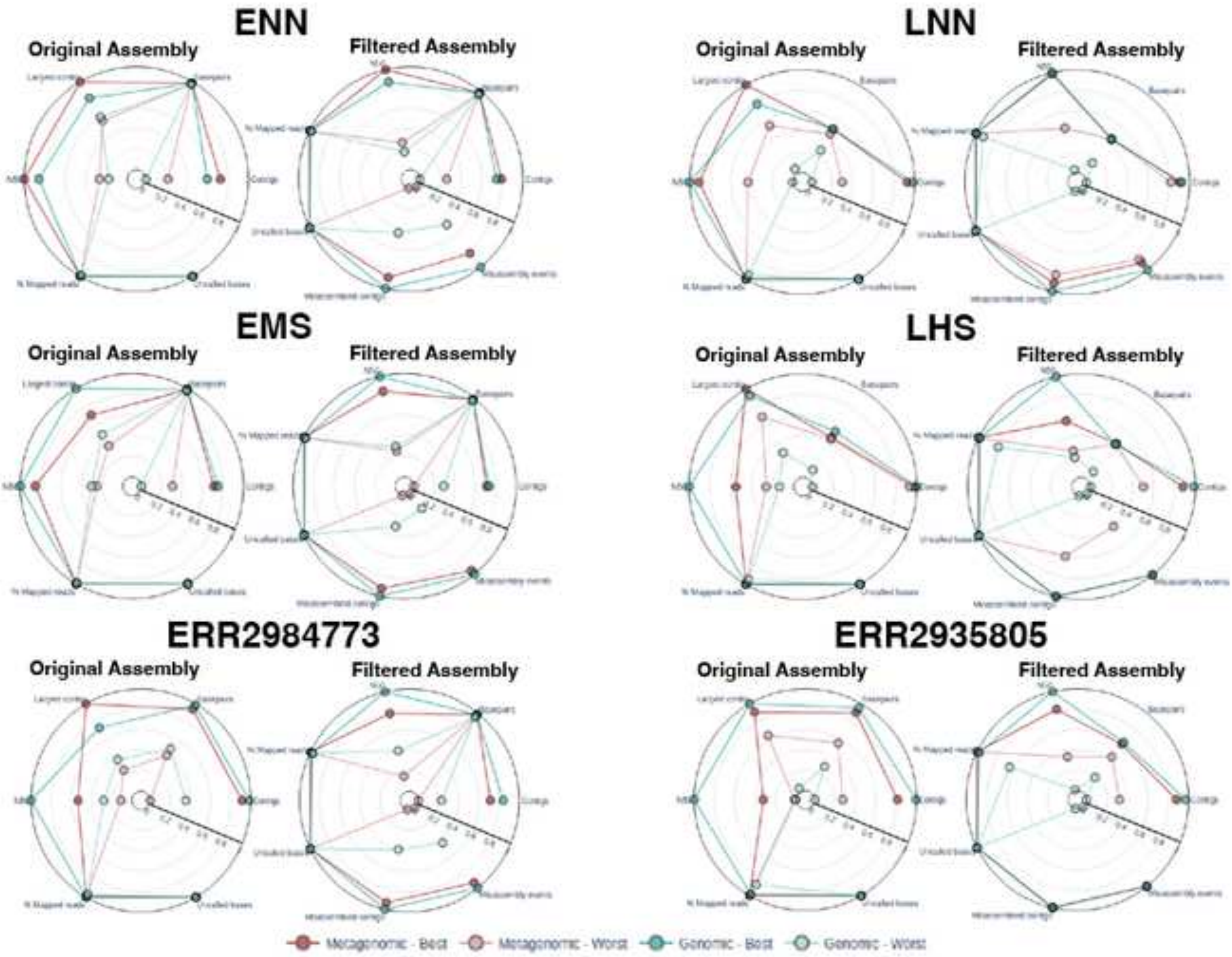

Figure 6

Click here to access/download;Figure;Figure 6.png

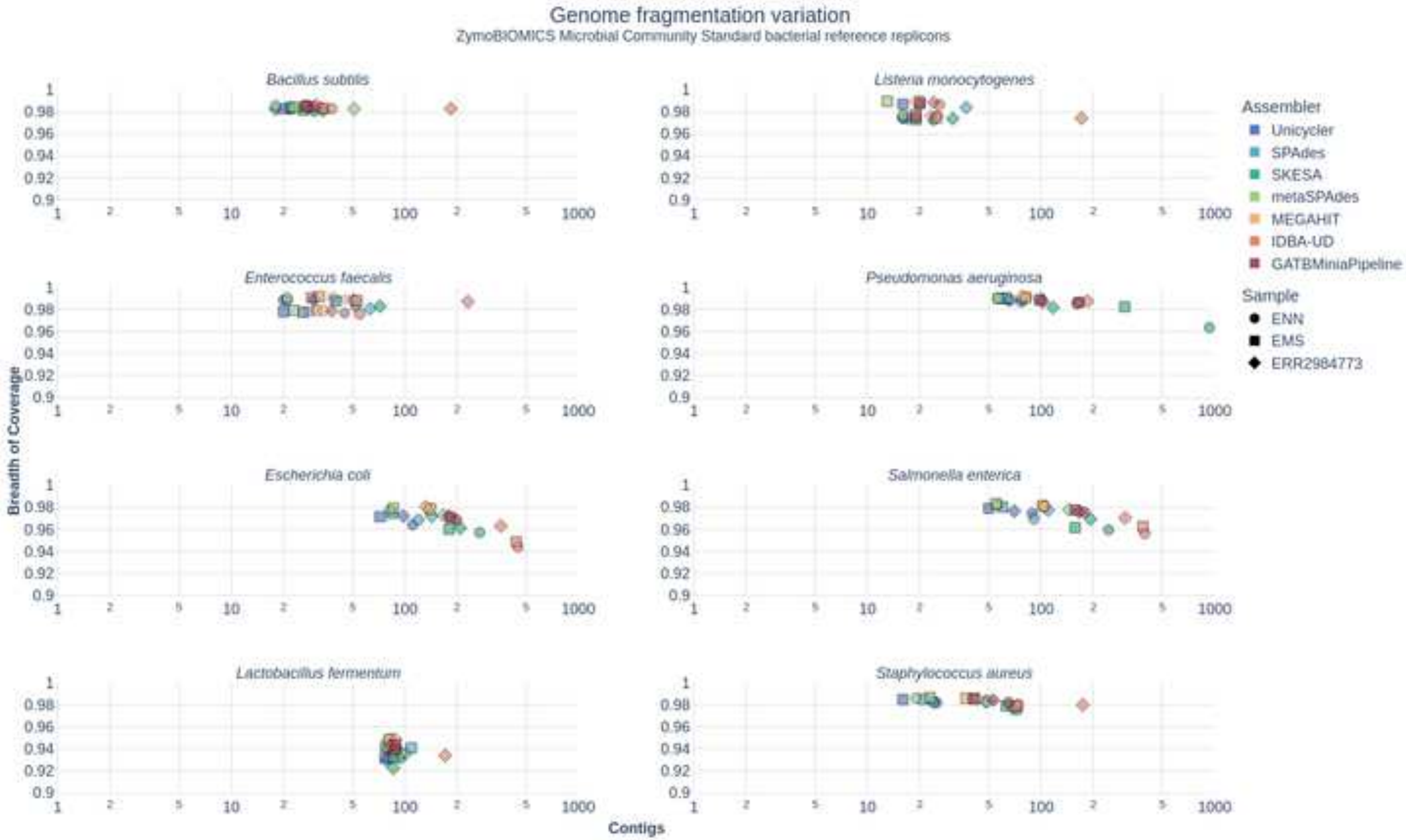

Figure 7

Click here to access/download;Figure;Figure 7.png

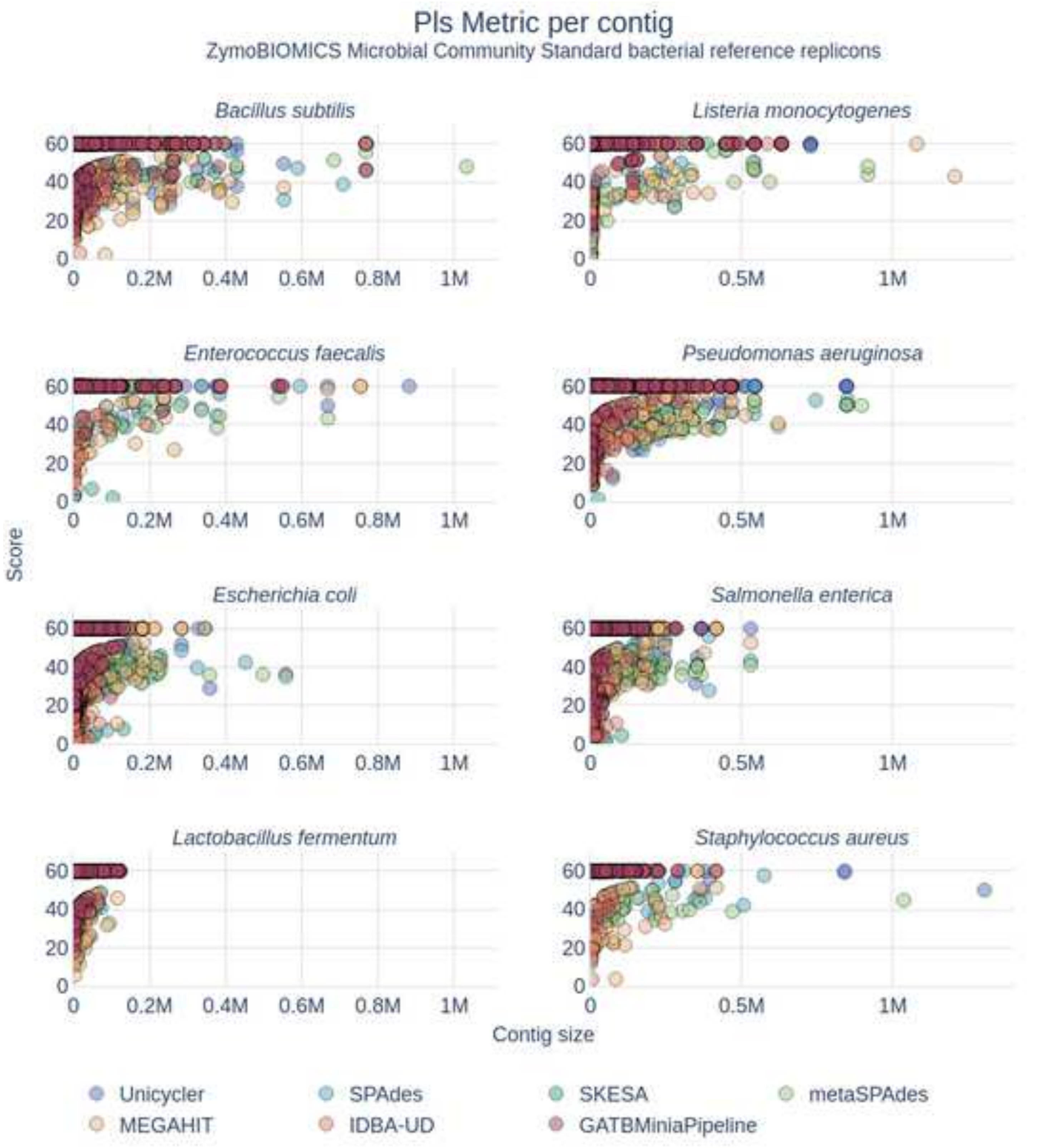

Figure 8

Click here to access/download;Figure;Figure 8.png

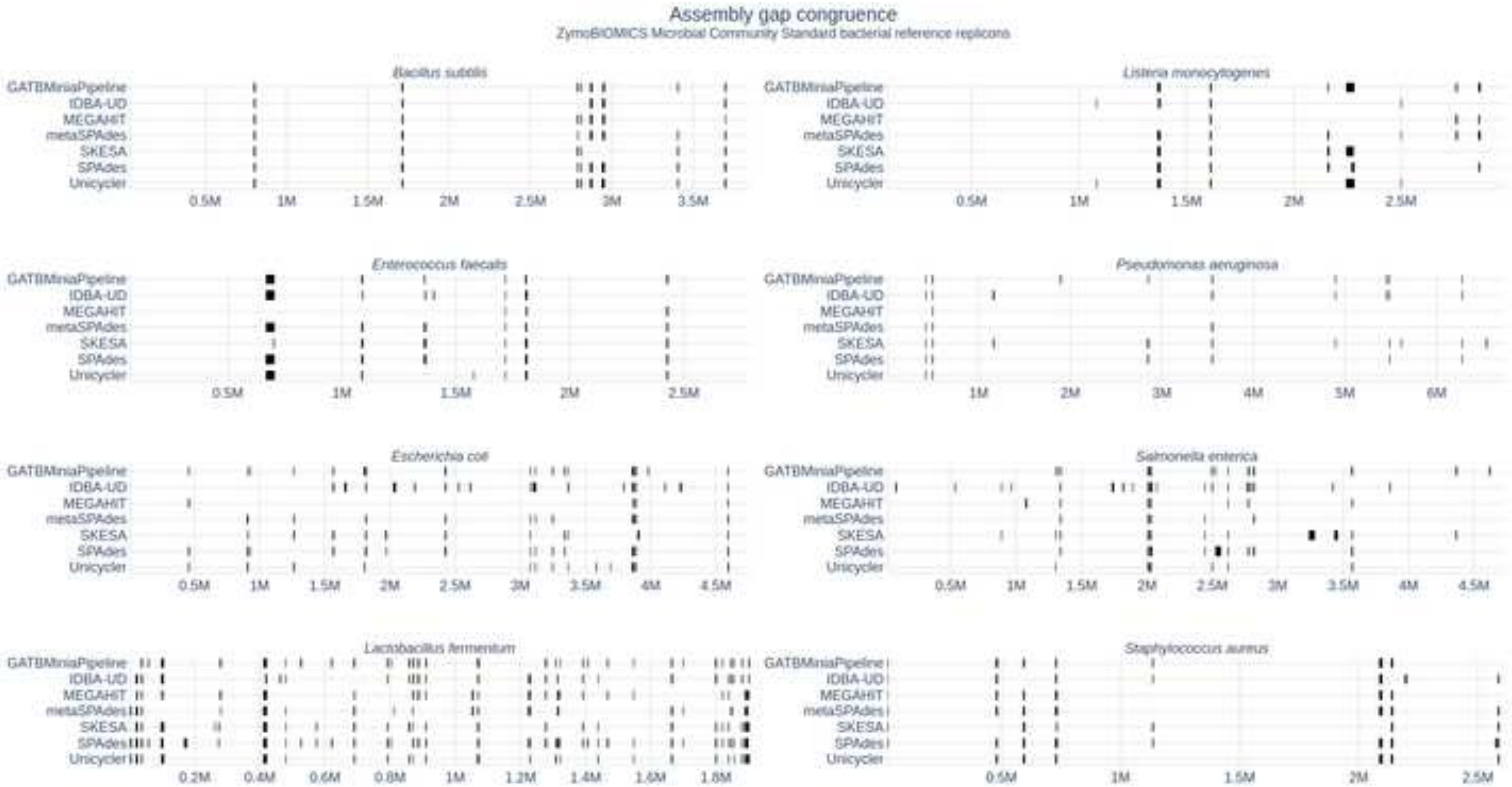

Supplementary Tables

Click here to access/download
Supplementary Material
LMAS Supplemental Material - Tables.xlsx

Click here to access/download
Supplementary Material
LMAS Supplemental Material - GigaScience.docx

Personal Cover

Click here to access/download;Personal Cover;LMAS - Cover letter.docx

Catarina Inês Mendes
Instituto de Medicina Molecular João Lobo Antunes
Lisbon, Portugal
cimendes@medicina.ulisboa.pt

Dear Dr Scott Edmunds, editor-in-chief of *GigaScience*,

I ask that you consider our manuscript entitled "*LMAS: Last Metagenomic Assembler Standing*" for publication in *GigaScience*.

Short-read shotgun metagenomics can offer comprehensive microbial detection and characterisation of complex clinical samples. The *de novo* assembly of raw sequence data is key in metagenomic analysis, yielding longer sequences that offer contextual information and afford a more complete picture of the microbial community. The assembly process is the bedrock and may constitute a major bottleneck in obtaining trustworthy, reproducible results.

In this manuscript, we present LMAS, an automated workflow developed as a flexible platform to allow users to evaluate traditional and metagenomic dedicated prokaryotic *de novo* assembly software performance given known standard communities. Its implementation in Nextflow ensures the transparency and reproducibility of the results obtained and the use of Docker containers provides further flexibility. The results are presented in an interactive HTML report where global and reference specific performance metrics can be explored. Currently, twelve assemblers are still being maintained and were implemented in LMAS, with the possibility of expansion as novel algorithms are developed and new versions released.

LMAS is intended as a tool empowering users to perform their own analysis in conditions meaningful in their context, in contrast to more generic benchmarking efforts such as CAMI. To showcase LMAS we used the test dataset of eight bacterial genomes and four plasmids of the ZymoBIOMICS Microbial Community Standards with linear and logarithmic species distribution, and found that k-mer De Bruijn graph assemblers outperformed the alternative approaches but came with a greater computational cost. Furthermore, assemblers branded as metagenomic specific did not consistently outperform other genomic assemblers in metagenomic samples. Some assemblers still in use, such as ABySS, BCALM2, MetaHipmer2, minia and VelvetOptimiser, showed significant performance problems and their usability may be limited with default parameters, particularly when assembling complex samples.

The performance of each assembler varied depending on the species of interest and its abundance in the sample, with less abundant species presenting a significant challenge for all assemblers. No assembler stood out as an undisputed all-purpose choice for short-read metagenomic prokaryote genome assembly, highlighting that efforts are still needed to further improve metagenomic assembler performance. Our results also suggest that sample complexity and a particular interest in some sample components may affect assembler choice. The great diversity of samples of interest further highlights the usefulness of a one-stop tool assisting users in their decision of which assembler to choose. Using LMAS could help users in their selection of assembler for their specific purpose. As such, we believe that this manuscript is appropriate for publication in *GigaScience* as a Technical note.

This manuscript has not been published and is not under consideration for publication elsewhere. LMAS has been featured in several international conferences such as ABPHM 2021 (doi: 10.5281/zenodo.6025166), ECCMID 2021 (doi: 10.5281/zenodo.6025210) and ICCMg 6 (doi: 10.5281/zenodo.5578327), the latter receiving the best poster award. All authors have approved the manuscript for submission and have no conflicts of interest to disclose.

Thank you for your consideration.
Sincerely, on behalf of all authors,

Catarina Inês Mendes, MSc
Email: cimendes@medicina.ulisboa.pt
Universidade de Lisboa, Instituto de Medicina Molecular, Instituto de Microbiologia
