# Supplementary material for: LMAS: evaluating metagenomic short de novo assembly methods through defined communities
Source: Gigascience. 2022 Dec 28;12:giac122. doi: 10.1093/gigascience/giac122 (PMC9795473; doi:10.1093/gigascience/giac122)
Supplement: giac122_GIGA-D-22-00108_Revision_1 [file giac122_giga-d-22-00108_revision_1.pdf]

GigaScience

# LMAS: Evaluating metagenomic short de novo assembly methods through defined communities

--Manuscript Draft--

| | |
|---|---|
| **Manuscript Number:** | GIGA-D-22-00108R1 |
| **Full Title:** | LMAS: Evaluating metagenomic short de novo assembly methods through defined communities |
| **Article Type:** | Technical Note |
| **Funding Information:** | Fundação para a Ciência e Tecnologia (SFRH/BD/129483/2017) — Miss Catarina Inês Mendes |
| **Abstract:** | Background The de novo assembly of raw sequence data is key in metagenomic analysis. It allows recovering draft genomes from a pool of mixed raw reads, yielding longer sequences that offer contextual information and provide a more complete picture of the microbial community.<br>Findings To better compare de novo assemblers for metagenomic analysis, LMAS was developed as a flexible platform allowing users to evaluate assembler performance given known standard communities. Overall, in our test datasets, k-mer De Bruijn graph assemblers outperformed the alternative approaches but came with a greater computational cost. Furthermore, assemblers branded as metagenomic specific did not consistently outperform other genomic assemblers in metagenomic samples. Some assemblers still in use, such as ABySS, BCALM2, MetaHipmer2, minia and VelvetOptimiser, perform relatively poorly and should be used with caution when assembling complex samples.<br>Conclusions The choice of a de novo assembler depends on the computational resources available, the replicon of interest, and the major goals of the analysis. No single assembler appeared an ideal choice for short-read metagenomic prokaryote replicon assembly, each showing specific strengths. The choice of metagenomic assembler should be guided by user requirements and characteristics of the sample of interest, and LMAS provides an interactive evaluation platform for this purpose. LMAS is open source and the workflow and its documentation are available at https://github.com/B-UMMI/LMAS and https://lmas.readthedocs.io/ respectively. |
| **Corresponding Author:** | Catarina Inês Mendes, MSc<br>Universidade de Lisboa Faculdade de Medicina<br>Lisboa, Portugal PORTUGAL |
| **Corresponding Author Secondary Information:** | |
| **Corresponding Author's Institution:** | Universidade de Lisboa Faculdade de Medicina |
| **Corresponding Author's Secondary Institution:** | |
| **First Author:** | Catarina Inês Mendes, MSc |
| **First Author Secondary Information:** | |
| **Order of Authors:** | Catarina Inês Mendes, MSc |
| | Pedro Vila-Cerqueira |
| | Yair Motro |
| | Jacob Moran-Gilad |
| | João André Carriço |
| | Mário Ramirez |
| **Order of Authors Secondary Information:** | |
| **Response to Reviewers:** | September 26, 2022<br>Catarina Mendes |

Powered by Editorial Manager® and ProduXion Manager® from Aries Systems Corporation

Instituto de Microbiologia, Instituto de Medicina Molecular
Faculdade de Medicina, Universidade de Lisboa
Av. Prof. Egas Moniz
PT 1649-028 Lisboa
Portugal
Email: cimendes@medicina.ulisboa.pt

Dr Nicole Nogoy
Executive Editor, GigaScience

Dear Dr. Nogoy,

We are grateful for the editorial and referral critique of our paper entitled "LMAS: Last Metagenomic Assembler Standing" (GIGA-D-22-00108) that we submitted for publication in GigaScience. The reviewers’ comments led to further analyses on our part which we believe enriched the paper. Moreover, it led us to highlight findings which we believe will be useful to the community, which were not immediately apparent previously, broadening the scope of the original paper. We have also carefully reviewed and modified the manuscript to further clarify in the text the points raised by the reviewers.

The CAMI datasets have remained popular after their publication (Sczyrba et al. 2017), Despite their popularity, no information is provided in the manuscript or in the supplemental material about the source of the genomes in each dataset. It is only mentioned that the community composition was designed according to specified criteria and the metagenome datasets simulated using https://github.com/CAMIchallenge/MetagenomeSimulationPipeline/releases/tag/0.1, which is no longer available. As such, despite the datasets being described as publicly available at https://data.cami-challenge.org/participate, only the “Gold Standard” assemblies are made available, and not the source references for the generation of the mock datasets. As the reviewer suggested, we tried to download the datasets and had difficulties obtaining the dataset following the information provided in the paper. We then contacted the authors and were informed that there were problems with the server where the data was hosted and that the data was unavailable (see attachment). Unfortunately there was no subsequent contact from the CAMI authors indicating the data had again become available.

As an alternative, and keeping in mind the requirement of the inclusion of closely related species in the dataset, we’ve introduced a new analysis in the manuscript of the BMock12 community (Sevim et al. 2019), composed of 12 species including several closely related sets: two replicons of Halomonas sp. (ANIb=0.98), three replicons of the Micromonospora genus (average ANIb=0.85) and two replicons of Marinobacter sp (ANIb=0.78). Furthermore, and to represent mock community trying to reproduce and existing microbiome, the NIBSC Gut DNA Reference Gut-Mix-RR and Gut-Mix-HiLo community standard (Amos et al. 2020) was analysed, consisting of 20 common gut microbiome strains in an even and staggered composition respectively. These strains have an average ANIb of 0.67, with a maximum of 0.95 between the two Bifidobacterium longum subspecies. It’s worth noting that no complete genome is available for eight of the strains in the sample, including one of the Bifidobacterium longum subspecies, therefore taking full advantage of the potential information in this sample is challenging. With the addition of these two datasets, we aim to provide both a high similarity case study as well as the usefulness of this tool using a perhaps more realistic mock community.

LMAS requires defined mock communities as a ground truth. To emphasise this requirement, we’ve changed the title of the manuscript to “LMAS: Evaluating metagenomic short de novo assembly methods through defined communities”. Below we will respond to all comments and corrections suggested by the reviewers.

We have submitted a new manuscript, with all changes highlighted in the text. The low quality figures have also been submitted separately to the submission platform in PDF format, as well as Zenodo (https://doi.org/10.5281/zenodo.6783042), including the original dynamic HTML files. Additionally, the reports for the ZymoBIOMICS Microbial Community Standard, BMock12 Community Standard and NIBSC Gut DNA Reference

Powered by Editorial Manager® and ProduXion Manager® from Aries Systems Corporation

are available at https://doi.org/10.5281/zenodo.7088960, https://doi.org/10.5281/zenodo.7092431 and https://doi.org/10.5281/zenodo.7092693, respectively. We hope to have satisfactorily addressed all the issues raised by the reviewers and hope that you find the revised version of the manuscript acceptable for publication.

With our best regards,
On behalf of all authors,

Catarina Mendes

Reviewer 1 - Major Comments
The authors describe "LMAS: Last Metagenome Assembler Standing", as a platform for evaluating different metagenome assemblers given known communities. Since the field of metagenome assemblers is a rapidly developing one, having good resources for evaluating and selecting methods available is an important topic. The paper itself is well-written and installation of the software via bioconda works out of the box. There are however some problems with the current form of the software and article which need to be addressed.

Regarding the software:

While starting the pipeline is easy enough, LMAS still requires a lot of resources since the assemblers themselves are run. Running the pipeline on the server available to me was not possible though (crashed with the error message "local avail memory attribute cannot zero. expression (avail memory 0). values avail memory = 0"). Using a lower max_cpus and max_memory option and running on a laptop (using the ENN dataset with even errors) was possible, but only a few assemblers were successful and the final report was not created.

We thank the reviewer for the comment. The default CPU and memory values in LMAS are the ones we found were required to successfully assemble the ZymoBIOMICS dataset. Assembly is a resource-intensive process, as is the mapping done in the evaluation of the assemblies by LMAS. Unfortunately, this is a limitation that LMAS cannot circumvent. Although The CPU and memory values can be easily changed, and maximum CPU and memory values defined, we cannot guarantee the success of the assembly process for such data. LMAS is robust to assembler failures, with the possibility of assemblers being skipped from the workflow, as well as an indication in the final report that an assembly process has failed. The cause for a lack of a final report would warrant further investigation, but it could be due to a lack of resources for the mapping process required by LMAS. To address the reviewer's concerns continuous integration of the LMAS workflow was implemented, for both docker and singularity systems, to ensure that the system is working as intended (https://github.com/B-UMMI/LMAS/actions). Test data is available in LMAS' repository (https://github.com/B-UMMI/LMAS/tree/main/test/data) consisting of a small in silico sample with just 200,000 read pairs belonging to Bacillus subtilis and Staphylococcus aureus that, due to its small size, is possible to run locally with relatively modest resources for most assemblers (Unicycler still requires a significant amount of resources with such small data).
Regarding the article:

The text mentions assemblers using OLC and dBg, but Table 1 shows only assemblers using deBruijn graphs were selected for evaluation. It is probably not necessary to mention OLC at all, since they have not been used for metagenome assemblers and the software selection reflects that.

We thank the reviewer for the comment. Indeed only dBg graph assemblers were ultimately included in LMAS. As such, and in accordance with the reviewer's

| | |
|---|---|
| | suggestion, the mention of OLC assemblers was removed from the background section of the manuscript (lines 60 to 63).<br><br>The definition of the PI-score (Equation 1) seems to be malformed: If E = 1 - Identity and assuming Identity is from 0-1 then E is always <60, so the first condition in Equation 1 needs to be changed.<br><br>We thank the reviewer for the comment. Indeed there was a mistake in Equation 1 that has now been corrected to the following:<br><br>"Assembly robustness" seems like a strange metric: Deterministic assemblers will always have the most robust results, but assemblers which use randomness are not necessarily worse; it is not clear to me why this is labeled as "poor results". In contrast to that, the LSA metric is an interesting addition to the set of metrics described in e.g. QUAST.<br><br>We thank the reviewer for the comment. We agree with the reviewer in regards to the fact that randomness does not translate directly into a worse result, therefore the assembly robustness alone should not be the metric of choice to exclude an assembler. Having said this, inconsistency of results from the same data may confuse more inexperienced users and cancreate difficulties if an assembler is to be included in a pipeline for accreditation. Having said this, we agree with the reviewer that non-deterministic assemblers are, by definition, non-reproducible. While not necessarily worse, they may be problematic for comparison with more deterministic assemblers in terms of the reproducibility of the comparison in different labs or at different times. We believe that the reproducibility of the assemblies generated is a piece of relevant information that should be included when assessing assembler performance. We have therefore changed “assembly robustness” to “assembler reproducibility” in Figure 3 (https://zenodo.org/record/7007317#.Yyn3Huxue3I).<br><br>The discovery that single k-mer dBg assemblers are typically outperformed by multi-k ones has been described before (e.g. IDBA-UD) and should be cited.<br><br>We thank the reviewer for the comment. Appropriate references have been added, namely (Sczyrba et al. 2017; Meyer et al. 2022; Xavier et al. 2014; Mahadik et al. 2019) (line 345).<br><br>Similarly, the dependence of assemblers to recover genomes with low abundance has been described before (e.g. CAMISIM).<br><br>We thank the reviewer for the comment. The appropriate reference has been added, namely (Sczyrba et al. 2017; Meyer et al. 2022; Fritz et al. 2019) (line 545).<br><br>Again in contrast to that, the influence of the individual species (particularly P. aeruginosa) on the assembly quality could have been elaborated on further.<br><br>Following the reviewer's suggestion, the following has been added to the manuscript (lines 470-471): However, in the case of the larger number of contigs of P. aeruginosa, no related species are present in the sample and these possibly reflect intrinsic properties of the replicon such as the high number of prophages integrated in the bacterial genome (Johnson et al, 2019). Unfortunately, existing mock communities do not exist allowing us to expand on the possible importance of replicon intrinsic properties in the quality of the assemblies produced. The availability of LMAS will allow more easily addressing this question by allowing researchers to focus on the experimental design of such communities.<br><br>For Figures 4 and Figure 7 many of the points cluster on top of each other, which makes manual inspection of these results hard to do. Generally the figures available to me were of quite low quality, they should either be vectorized or of higher quality.<br><br>We thank the reviewer for this comment. We apologise for the lack of readability regarding Figures 4 and 7, and have uploaded high-resolution PDF versions of each. Figure 5 was also uploaded as a PDF for the same reason.  Additionally, All figures are now available, in their original form, in Zenodo under DOI |

Powered by Editorial Manager® and ProduXion Manager® from Aries Systems Corporation

| | |
|---|---|
| | https://doi.org/10.5281/zenodo.6783042. The availability of data and materials has been updated to reflect this addition, now reading “Likewise, all figures in the current manuscript are available in their original format in the Zenodo repository, under https://doi.org/10.5281/zenodo.6783042.” (lines 660-662).<br><br>The conclusion that metagenome assemblers do not outperform genome assemblers is based on the - low complexity - data sets. This problem is touched upon in the conclusion but the header in the main text can be misleading. To come to the conclusion that metagenome assemblers do not outperform genome assemblers, LMAS would need to show this on at least one dataset with somewhat realistic complexity, e.g. the ones used for the mentioned CAMI challenge. Doing this could also show the advantage of LMAS against the mainly used QUAST in bundling the execution and evaluation together.<br><br>As stated throughout the manuscript, LMAS requires defined mock communities as a source of ground truth. We appreciate the reviewer’s comment regarding the misleading nature of the header of the manuscript. To emphasise this requirement, we’ve changed the title of the manuscript to “LMAS: Evaluating metagenomic short de novo assembly methods through defined communities”<br><br>Given the problems in obtaining the CAMI samples we have tried to find relevant mock communities, sequenced using short-read technologies,of greater complexity than the ZymoBIOMICS community. Keeping in mind the requirement of the inclusion of closely related strains in the dataset, we’ve introduced a new analysis in the manuscript of the BMock12 community (Sevim et al. 2019). The challenges of this dataset are the two Halomonas and the two Marinobacter strains, which come with an average ANIb of 0.98 and 0.85 (see Supplementary Table S25), respectively. Additionally, the NIBSC Gut DNA Reference Gut-Mix-RR and Gut-Mix-HiLo datasets were analysed, representing a more naturally occurring microbiome that includes 5 phyla, 13 families, 16 genera and 19 species that includes several abundant species and strains present in the human gut (Amos et al. 2020). With this, we wish to better showcase the usefulness of this tool using more difficult datasets. The reports for the ZymoBIOMICS Microbial Community Standard, BMock12 Community Standard and NIBSC Gut DNA Reference are available at https://doi.org/10.5281/zenodo.7088960, https://doi.org/10.5281/zenodo.7092431 and https://doi.org/10.5281/zenodo.7092693 respectively (lines 666-669 in the manuscript).<br>Following the analysis of these new datasets, the conclusion that the metagenomics assemblers do not outperform the genomic assemblers still holds true.<br><br>Reviewer 2 - Major Comments<br><br>This is a technical note that describes LMAS, a tool that launches multiple genome assemblers on the same metagenomic dataset and then generates a report with various assembly quality metrics. The metrics, including two newly introduced ones, make sense. The choice of assemblers also generally makes sense, although I do have some remarks below regarding a couple choices. The analysis is thorough and provides some interesting takeaway messages. The manuscript is well-written and the presentation is didactic.<br><br>My main doubt about this article is whether the LMAS tool would be of broad interest. Between existing large-scale benchmarking efforts like CAMI and popular evaluation tools that generate assembly reports like MetaQUAST, it is difficult to assess the precise need for LMAS. The suggested use case by the authors is that a user should run him/herself the assemblers on a particular dataset of interest. The advantage would therefore be to have an integrated solution to run all tools and evaluate the results automatically. While this is handy, a user might also simply run a 2-3 of the best assemblers as suggested by the article (or CAMI), considering that many of the LMAS produce suboptimal assemblies and they are not reconciled.<br><br>A redeeming aspect of the article is the newly made benchmark on low-complexity Zymo real & simulated data (8 bacterial species). This benchmark contains some |

interesting findings, such as the fact that genomic assemblers appear to perform as well a metagenomes ones. This claim should however be put into context: the metagenomic data is low-complexity (only 8 species), and the presence of similar strains in actual metagenomes may significantly change that message.

The authors may want to make a stronger case why a user would want to run LMAS instead of picking 2 (or 3) of the best-performing assemblers and run MetaQUAST on them. In an actual assembly scenario, i.e. without ground truth, are there situations where running more than 2 assemblers is beneficial? I am genuinely interested in the answer.

We thank the reviewer for this comment. The purpose of LMAS is, given a ground truth of a defined mock community, to enable the benchmarking of traditional and metagenomic prokaryotic de novo assembly software. Although we understand the reviewer's point that CAMI offers this guidance, CAMI challenges are spaced in time and assemblers are released at a much faster pace, so a user may be interested in evaluating a novel assembler without the "self-assessment" trap associated with the publication of novel tools before the next CAMI challenge. Moreover, our analyses show that most of the conclusions on assembler performance are not generalisable, therefore there is no absolute guarantee that the best performing assemblers for a given scenario will be the same as for a different one (namely considering the replicon of interest, its abundance in the sample and other replicons present in the sample). Therefore, we encourage users to run the assemblers of interest with mock communities as close as possible to their sample of interest. Admittedly, a user may want to concentrate on 2-3 assemblers, but the task of running these assemblers followed by MetaQUAST may still be daunting. LMAS, with its modular and streamlined design, lowers the barrier of performing such analyses. Moreover, in addition to the novel quality metrics, LMAS offers representations which are not offered by MetaQUAST, such as the positions of assembly gaps and of SNPs in the genomes of interest which can be explored in the future to further understand the strengths and weaknesses of particular assemblers.

To better reinforce some of these points, the following sections have been added to the text: "The portability and ease of use of LMAS are intended to provide users with a continuous benchmarking platform to easily evaluate the performance of assemblers, as they are developed, in mock communities mimicking as closely as possible their samples of interest." and "Even when considering communities with very similar replicons, the overall performance of metagenomic assemblers was not consistently better than that of genomic assemblers. The results also indicate that the recovery of assemblies allowing strain-level discrimination at the SNP level is highly unlikely based solely on the assembler generated contigs." (lines 580-583).

Were there multiple high-similarity strains in the Zymo data? If not, this should be noted in the presentation of the dataset. This is an important caveat that limits the significance of the reported results. In particular I would suggest to tone down the claim that genomic assemblers perform as well as metagenomic assemblers, given e.g. the many heuristics that MetaSpades implements to resolve close strains.

We thank the reviewer for this comment. The similarity of the strains in the ZymoBiomics is available as supplemental table S23, where pairwise comparisons of the microbial community standard reference replicons are available. As observed in this table, Escherichia coli and Salmonella enterica have the highest level of similarity, with an ANIb of 0.80.

Keeping in mind the need of analysing closely related species in the same mock community, we've introduced a new analysis in the manuscript of the BMock12 community (Sevim et al. 2019), composed of 12 strains including several closely related sets: two replicons of Halomonas sp. (ANIb=0.98), three replicons of the Micromonospora genus (average ANIb=0.85) and two replicons of Marinobacter sp (ANIb=0.78). We hope that, with the introduction of the analysis of this dataset, we have successfully addressed the scenario raised by the reviewer where multiple similar strains are present in the sample. Essentially, our initial assertion stands, with genomic assemblers not being systematically outperformed by metagenomic assemblers.

Keeping in mind the reviewer suggestions, the following has been added to the conclusion of the manuscript (lines 567-573): “Although the eight species ZymoBIOMICS Microbial Community Standards might not be representative of the metagenomic complexity of the samples of interest of most researchers, we hoped that its relative simplicity meant that the results shown would represent a best-case scenario, since as sample complexity increases so do the challenges to assembler performance. However, the results of the BMock12 and Gut-Mix community standards suggest that the actual genome of interest and community composition play an important part in the results of individual assemblers.”.

The abstract sentence: "Some assemblers still in use, such as ABySS, BCALM2, MetaHipmer2, minia and VelvetOptimiser" should be removed or revised as it is misleading for several reasons: BCALM2 is not an assembler, it constructs unitigs only. Minia is a part of GATBMiniaPipeline which offers better performance. And VelvetOptimiser is an odd choice as MetaVelvet also exists and was untested (although it might not scale). Therefore, I recommend VelvetOptimiser, BCALM2, and possibly minia to be removed from the benchmark, or be marked as odd choices for metagenome assembly.

We thank the reviewer for this comment. MetaVelvet has been last updated in 2015 (Supplemental Table S1), therefore it was not considered for the benchmark by not meeting our inclusion criteria. Due to the very fair point made regarding BCALM2, it has been removed from LMAS (https://github.com/B-UMMI/LMAS/pull/25), and the manuscript, figures and supplemental material adjusted accordingly. Regarding minia, a similar argument can be made for SPAdes and Unicycler assemblers. Therefore it has not been removed from LMAS and is still considered in the initial benchmark with the ZymoBIOMICS Microbial Community Standard, together with VelvetOptimiser. Given their very poor performance, their execution is now by default set to false in LMAS, alongside Abyss and MetaHipermer2, since given the results presented here these would not be recommended assemblers.

Of note, albeit VelvetOptimiser tries multiple k values, it is still considered to be a single-k assembler as it does not combine the results of multiple iterations. (It merely picks the best k and runs single-k.)

We thank the reviewer for this comment. Table 1 and supplemental Table S1 have been adjusted accordingly.

Some of the results are obvious, e.g. single-k assemblers perform more poorly than multiple-k assemblers; this is not quite a novel finding.

We thank the reviewer for this comment. Indeed the notion that multiple k-mer assemblers outperform single k-mer assemblers is not novel, but it is our opinion that there is still limited data supporting this assertion. Therefore, we found it worth mentioning and reinforcing this notion in our manuscript. As such, we have not changed the text. Instead, and in accordance with the comments of reviewer 1, the following references have been added to support this claim: (Sczyrba et al. 2017; Meyer et al. 2022; Xavier et al. 2014; Mahadik et al. 2019) (line 345 in the manuscript).

Figures 4 and 5, even when downloaded as PNG, were low resolution and hardly readable.

We thank the reviewer for this comment. We apologise for the lack of readability regarding Figures 4 and 5, and have uploaded a high-resolution PDF version of each. Additionally, All figures are now available, in their original form, in Zenodo under DOI https://doi.org/10.5281/zenodo.6783042 (lines 660-662 in the manuscript).

Reviewer 2 - Minor Comments

The term "replicon" is used ubiquitously in the paper, and while its definition is biologically sound, it could be worthwhile to recall what the authors precisely mean by it.

Powered by Editorial Manager® and ProduXion Manager® from Aries Systems Corporation

We thank the reviewer for the comment. In this manuscript, the term "replicon" is used to refer to DNA sequences replicating as a single unit,  containing a single origin of replication, and therefore excludes the genomes of eukaryotes which can initiate replication from several loci. We've altered the following sentence in the background section of the manuscript (lines 49 to 51) to better inform the reader: The de novo assembly process is key when analysing metagenomic data since it allows recovering contigs representing the replicons present in the sample, be it prokaryotic chromosomes, plasmids or viruses, from a pool of mixed raw reads.

While running LMAS on my cluster head node. Some issues occur. The pulling of singularity images did not work. The error message was:

Error executing process > 'preprocessing_wf:PROCESS_REFERENCE (1)'

Caused by:
  Failed to pull singularity image
  command: singularity pull  --name cimendes-lmas-python-base-0.1.img.pulling.1654889379926 docker://cimendes/lmas-python-base:0.1 > /dev/null
  status : 255
  message:
INFO:Converting OCI blobs to SIF format
FATAL:   While making image from oci registry: error fetching image to cache: while building SIF from layers: unable to create new build: while ensuring correct compression algorithm: while creating squashfs: create command failed: exit status 1: FATAL ERROR:Failed to create thread

However, running the command myself:

$ singularity pull  --name cimendes-lmas-python-base-0.1.img.pulling.1654889379926 docker://cimendes/lmas-python-base:0.1

Worked.
Then, after that, since I need to run LMAS on the head node for the first time (other nodes are not connected to internet so cannot pull containers), I get the following error message:

Error executing process > 'assembly_wf:SKESA (ERR2935805)'

Caused by:
Process requirement exceed available CPUs -- req: 8; avail: 4

In summary, it would be helpful to have instructions to pull all containers from the head node ahead of time to prepare for LMAS execution on a node not connected to Internet.

We thank the reviewer for this observation. Continuous integration with singularity has been added to LMAS under  https://github.com/B-UMMI/LMAS/pull/23. Indeed we observe that the pull of the images with singularity is sometimes inconsistent but restarting the workflow solves the issue most times. Additionally, in the LMAS documentation the requirement of an internet connection to download the required images was added as Nextflow automatically handles the pull of the required images. Alternatively, a script has been included to handle the pulling of the required images using shifter, docker or singularity (https://github.com/B-UMMI/LMAS/pull/31) before running LMAS for the first time to allow users to run LMAS without the problems described by the reviewer.
References

Amos, Gregory C. A., Alastair Logan, Saba Anwar, Martin Fritzsche, Ryan Mate, Thomas Bleazard, and Sjoerd Rijpkema. 2020. 'Developing Standards for the

Microbiome Field'. Microbiome 8 (1): 98. https://doi.org/10.1186/s40168-020-00856-3.
Fritz, Adrian, Peter Hofmann, Stephan Majda, Eik Dahms, Johannes Dröge, Jessika Fiedler, Till R. Lesker, et al. 2019. 'CAMISIM: Simulating Metagenomes and Microbial Communities'. Microbiome 7 (1): 17. https://doi.org/10.1186/s40168-019-0633-6.
Mahadik, Kanak, Christopher Wright, Milind Kulkarni, Saurabh Bagchi, and Somali Chaterji. 2019. 'Scalable Genome Assembly through Parallel de Bruijn Graph Construction for Multiple K-Mers'. Scientific Reports 9 (1): 14882. https://doi.org/10.1038/s41598-019-51284-9.
Meyer, Fernando, Adrian Fritz, Zhi-Luo Deng, David Koslicki, Till Robin Lesker, Alexey Gurevich, Gary Robertson, et al. 2022. 'Critical Assessment of Metagenome Interpretation: The Second Round of Challenges'. Nature Methods 19 (4): 429–40. https://doi.org/10.1038/s41592-022-01431-4.
Sczyrba, Alexander, Peter Hofmann, Peter Belmann, David Koslicki, Stefan Janssen, Johannes Dröge, Ivan Gregor, et al. 2017. 'Critical Assessment of Metagenome Interpretation—a Benchmark of Metagenomics Software'. Nature Methods 14 (11): 1063–71. https://doi.org/10.1038/nmeth.4458.
Sevim, Volkan, Juna Lee, Robert Egan, Alicia Clum, Hope Hundley, Janey Lee, R. Craig Everroad, et al. 2019. 'Shotgun Metagenome Data of a Defined Mock Community Using Oxford Nanopore, PacBio and Illumina Technologies'. Scientific Data 6 (1): 285. https://doi.org/10.1038/s41597-019-0287-z.
Xavier, Basil Britto, Julia Sabirova, Moons Pieter, Jean-Pierre Hernalsteens, Henri de Greve, Herman Goossens, and Surbhi Malhotra-Kumar. 2014. 'Employing Whole Genome Mapping for Optimal de Novo Assembly of Bacterial Genomes'. BMC Research Notes 7 (1): 484. https://doi.org/10.1186/1756-0500-7-484.

| Additional Information: | |
|---|---|
| **Question** | **Response** |
| Are you submitting this manuscript to a special series or article collection? | No |
| **Experimental design and statistics**<br><br>Full details of the experimental design and statistical methods used should be given in the Methods section, as detailed in our Minimum Standards Reporting Checklist. Information essential to interpreting the data presented should be made available in the figure legends.<br><br>Have you included all the information requested in your manuscript? | Yes |
| **Resources**<br><br>A description of all resources used, including antibodies, cell lines, animals and software tools, with enough information to allow them to be uniquely identified, should be included in the Methods section. Authors are strongly encouraged to cite Research Resource Identifiers (RRIDs) for antibodies, model organisms and tools, where possible. | Yes |

Powered by Editorial Manager® and ProduXion Manager® from Aries Systems Corporation

| | |
|---|---|
| Have you included the information requested as detailed in our Minimum Standards Reporting Checklist? | |
| **Availability of data and materials**<br><br>All datasets and code on which the conclusions of the paper rely must be either included in your submission or deposited in publicly available repositories (where available and ethically appropriate), referencing such data using a unique identifier in the references and in the "Availability of Data and Materials" section of your manuscript.<br><br>Have you have met the above requirement as detailed in our Minimum Standards Reporting Checklist? | Yes |

*Powered by Editorial Manager® and ProduXion Manager® from Aries Systems Corporation*

Manuscript

Click here to access/download;Manuscript;LMAS Manuscript_for_publication.docx

# LMAS: Evaluating metagenomic short *de novo* assembly methods through defined communities

**C I Mendes***, Instituto de Microbiologia, Instituto de Medicina Molecular, Faculdade de Medicina, Universidade de Lisboa, Lisboa, Portugal, cimendes@medicina.ulisboa.pt, https://orcid.org/0000-0002-3090-7426

P Vila-Cerqueira, Instituto de Microbiologia, Instituto de Medicina Molecular, Faculdade de Medicina, Universidade de Lisboa, Lisboa, Portugal, pedro.cerqueira@medicina.ulisboa.pt, https://orcid.org/0000-0002-6121-8906

Y Motro, Faculty of Health Sciences, Ben-Gurion University of the Negev, Beer-Sheva, Israel, motroy@post.bgu.ac.il, https://orcid.org/0000-0003-1289-6919

J Moran-Gilad, Faculty of Health Sciences, Ben-Gurion University of the Negev, Beer-Sheva, Israel, giladko@post.bgu.ac.il, https://orcid.org/0000-0001-9134-050X

J A Carriço, Instituto de Microbiologia, Instituto de Medicina Molecular, Faculdade de Medicina, Universidade de Lisboa, Lisboa, Portugal, jcarrico@medicina.ulisboa.pt, https://orcid.org/0000-0002-5274-2722

M Ramirez, Instituto de Microbiologia, Instituto de Medicina Molecular, Faculdade de Medicina, Universidade de Lisboa, Lisboa, Portugal, ramirez@medicina.ulisboa.pt, https://orcid.org/0000-0002-4084-6233

*** corresponding author**

# Abstract

**Background** The *de novo* assembly of raw sequence data is key in metagenomic analysis. It allows recovering draft genomes from a pool of mixed raw reads, yielding longer sequences that offer contextual information and provide a more complete picture of the microbial community.

**Findings** To better compare *de novo* assemblers for metagenomic analysis, LMAS was developed as a flexible platform allowing users to evaluate assembler performance given known standard communities. Overall, in our test datasets, k-mer De Bruijn graph assemblers outperformed the alternative approaches but came with a greater computational cost. Furthermore, assemblers branded as metagenomic specific did not consistently outperform other genomic assemblers in metagenomic samples. Some assemblers still in use, such as ABySS, BCALM2, MetaHipmer2, minia and VelvetOptimiser, perform relatively poorly and should be used with caution when assembling complex samples. Meaningful strain resolution at the SNP-level was not achieved, even by the best assemblers tested.

**Conclusions** The choice of a *de novo* assembler depends on the computational resources available, the replicon of interest, and the major goals of the analysis. No single assembler appeared an ideal choice for short-read metagenomic prokaryote replicon assembly, each showing specific strengths. The choice of metagenomic assembler should be guided by user requirements and characteristics of the sample of interest, and LMAS provides an interactive evaluation platform for this purpose. LMAS is open source and the workflow and its documentation are available at https://github.com/B-UMMI/LMAS and https://lmas.readthedocs.io/ respectively.



# Keywords

Shotgun Metagenomics, *de novo* assembly, benchmark, draft genome quality, simulation

# Background

Short-read shotgun metagenomics has the potential to offer comprehensive microbial detection and characterisation of complex clinical or environmental samples. Despite becoming an increasingly used approach, it comes at the cost of producing massive amounts of data that require expert handling and processing, as well as adequate computational resources. The *de novo* assembly process is key when analysing metagenomic data since it allows recovering contigs representing the replicons present in the sample, be it prokaryotic chromosomes, plasmids or viruses, from a pool of mixed raw reads. These contigs are longer sequences that offer better contextual information than reads alone and provide a more complete picture of the microbial community than the species composition. Despite efforts for the development, standardization and assessment of software for metagenomic analysis, both commercial and open-source [1–5], the *de novo* assembly process still represents a critical point in these analyses.

The assembly of draft genomes has become a central step when analysing pure bacterial cultures, for instance allowing genomic comparisons through single nucleotide polymorphisms (SNPs) or gene-by-gene methods, such as core-genome multilocus sequence typing (cgMLST). De Bruijn graphs (dBg) algorithms are currently the most widely used approaches in modern assembly software. dBg handles unresolvable repeats by essentially fragmenting the sequence, that is, forming multiple contigs for each of the possibly contiguous sequences present in the sample. Additionally, the inherent heterogeneity of complex samples, potentially containing a multitude of replicons, could make traditional genome assemblers, implementing optimizations based on the assumption of having a single genome in the sample, not suitable for metagenomics [6].

Several dedicated metagenomic assembly tools for short-read data are available [6]. These tools are generally assumed to perform better when dealing with complex samples having a combination of intragenomic and intergenomic repeats and uneven and low coverage

sequencing depths of some of the replicons [7]. Not using dedicated metagenomic assemblers was suggested to come with the cost of generating artificial variation and chimeric contigs, especially in samples that contain closely related species [8]. However, no formal comparison has been done looking at increased accuracy or gains in contiguity of assemblies obtained with metagenomic assemblers versus traditional assemblers.

With an ever-increasing range of both traditional and metagenomic assemblers becoming available, choosing the best performing tool can be an arduous and time-consuming task since the choice may vary depending on the purpose of the analysis, organism of interest, complexity of the sample and computational infrastructure available. Additionally, the evaluation of the resulting contigs is not straightforward since one metric is not sufficient to classify an assembly, particularly with complex samples [7,9]. Despite several *de novo* assembly validation methods relying on features of the created contigs themselves, such as QUAST [10], being useful in identifying inconsistencies indicative of potential assembly errors, the use of reference-based validation methods offer the possibility of a more complete evaluation of accuracy and are particularly important to benchmark attempts to reconstruct communities. MetaQUAST [11] , a modification of QUAST, extends the original software by performing assembly evaluation based on aligning contigs to a reference, which can be provided or inferred by the software, and reports, in addition to the standard metrics for single genomes reported by QUAST, the number of interspecies translocations and the number of possibly misassembled contigs.

The use of mock communities, with known composition, abundance and genomic information, provides a ground truth against which the success of the assembly of a complex sample can be evaluated. Furthermore, this can be done in circumstances in which the errors introduced by sample processing and the sequencing and associated methods used approximate, as much as possible, their effects in real samples. Such mock communities facilitate the identification of misassemblies, such as chimeric sequences generated from the improper combination of two distinct replicons, indels or single nucleotide variants improperly created

by the assembler. On the other hand, the comparison of the performance of two assemblers is only possible if the input data is the same and if the same evaluation metrics are applied [3].

To tackle these challenges, we developed LMAS (Last Metagenomic Assembler Standing), an automated workflow to enable the benchmarking of traditional and metagenomic prokaryotic *de novo* assembly software using defined mock communities. The results of LMAS are presented in an interactive HTML report where selected global and reference replicon specific performance metrics can be explored. The mock communities can be provided by the user to better reflect the samples of interest. New assemblers can be added with minimal changes to the pipeline so that LMAS can be expanded to include novel algorithms as they are developed. The portability and ease of use of LMAS are intended to allow provide users with a continuous benchmarking platform to easily evaluate the performance of assemblers, in mock communities mimicking as closely as possible their samples of interest.

# The LMAS Workflow

## Workflow overview

LMAS is a user-friendly automated workflow enabling the benchmarking of traditional and metagenomic prokaryotic *de novo* assembly software using defined mock communities. LMAS was implemented in Nextflow [12] to provide flexibility and ensure the transparency and reproducibility of the results. LMAS relies on the use of Docker [13] containers for each assembler, allowing versions to be tracked and changed easily.

Figure 1: **The LMAS workflow.** The input sequencing data is assembled in parallel, resources permitting, by the set of assemblers included in LMAS. The resulting contigs are processed and the global quality assessment is performed. After filtering for the user-defined minimum contig size, the remaining sequences are mapped against the provided reference and the resulting information is processed to evaluate assembly quality by replicon in the reference

file. All results, and optional text information describing the samples, are grouped in the LMAS report.

## Installation and Usage

LMAS can be installed through Bioconda [14] or Github [15], with detailed instructions available in the documentation [16]. LMAS requires as inputs the complete reference replicons (genomes, plasmids or any other replicons present) and short-read paired-end raw data. All complete references (linear replicons) should be provided in a single file. This raw data can be either obtained *in silico* by creating simulated reads from the reference replicons or sequencing mock communities of known composition. Optionally, information on the input samples in a markdown file can be provided to be presented in the report.

A step-by-step execution tutorial is available at [17]. Users can customize the workflow execution either by using command-line options or by modifying the simple plain-text configuration files. To make the execution of the workflow as simple as possible, a set of default parameters and directives is provided. A complete description of each parameter is available in Supplemental Material (see Supplemental Material, Workflow parameters), as well as in the documentation [18]. The results are presented in an interactive HTML report, stored in the "*report*" folder in the directory of LMAS' execution. The output files of all assemblers and quality assessment processing scripts in the workflow are stored in the "*results*" folder, in the same location.

## Supported Assemblers and selection criteria

A collection of *de novo* assembly tools was compiled, including OLC and dBg assembly algorithms, the latter including both single k-mer and multiple k-mer value approaches, and hybrid assemblers implementing both algorithms, including both genomic and metagenomic assemblers (Supplemental Table S1). Of these, 11 assemblers were selected based on the date of last update (at least 2015) and are implemented in LMAS: ABySS [19] (version 2.3.1),

GATB Minia Pipeline [20] (commit hash 9d56f42) , IDBA-UD [21] (version 1.1.3), MEGAHIT [22] (version 1.2.9), MetaHipMer2 [23] (version 2.0.0.65-gaad446d-dirty-AddGtest), metaSPAdes [24] (version 3.15.3), minia [25] (version 3.2.6), SKESA [26] (version 2.5.0), SPAdes [27] (version 3.15.3), Unicycler [28] (version 0.4.9) and VelvetOptimiser [29] (commit hash 092bdee) (Table 1). The execution commands for each assembler are available as Supplemental Material (see Supplemental Material, Short-read *de novo* assemblers) and in the documentation [30].

New assemblers can be added with minimal changes to the pipeline so that LMAS can be expanded as novel algorithms are developed. A template is available to facilitate their integration and a step-by-step guide is included in the documentation [31]. The only two requirements for the addition of a new assembler are the execution command for the assembler for paired-end short-read data and a Nextflow-compatible container with the assembler and any dependencies.

**Table 1: Prokaryotic *de novo* assemblers integrated into LMAS.**

| Assembler | Type | Algorithm |
|---|---|---|
| **GATBMiniaPipeline** | Metagenomic | Multiple k-mer De Bruijn graph |
| **IDBA-UD** | Metagenomic | Multiple k-mer De Bruijn graph |
| **MEGAHIT** | Metagenomic | Multiple k-mer De Bruijn graph |
| **MetaHipMer2** | Metagenomic | Multiple k-mer De Bruijn graph |
| **metaSPAdes** | Metagenomic | Multiple k-mer De Bruijn graph |
| **ABySS** | Genomic | Single k-mer De Bruijn graph |
| **MINIA** | Genomic | Single k-mer De Bruijn graph |
| **SKESA** | Genomic | Multiple k-mer De Bruijn graph |
| **SPAdes** | Genomic | Multiple k-mer De Bruijn graph |
| **Unicycler** | Genomic | Multiple k-mer De Bruijn graph |
| **VelvetOptimizer** | Genomic | Single k-mer De Bruijn graph |

## Assembly Quality Metrics

The success of an assembly is evaluated in two steps: globally (see Global Metrics) and relative to each of the replicons present in the sample (see Per Reference Metrics). In both, the tabular presentation in the reports allows the comparison of exact values between assemblers, and the interactive plots allow a more intuitive overview and easy exploration of results. In addition to the assembly success metrics, computational resource statistics are registered for each assembler (see Supplemental Material, LMAS Metrics, Computational Performance Metrics).

### Global Metrics

The computation of the global metrics is performed through statistics inherent to the complete set of contigs assembled per sample, independent of the species/sample of origin. The metrics are presented, in tabular form, for the complete set of contigs and those filtered for a minimum length, and also graphically for the contigs filtered for a minimum length. The statistics include information on contig number, size and ambiguous bases; and the proportion of reads mapping to the created contigs. Two statistics are a consolidation of per reference metrics: misassemblies (i.e. contigs that do not reflect the structural organization in the reference replicons); and the overall size of gaps in all reference replicons not covered by any contig. A more detailed description of all global metrics is available in Supplemental Material (see Supplemental Material, LMAS Metrics, Global Metrics).

### Per Reference Metrics

For the computation of the reference-based metrics, only the filtered set (FS) contigs are considered, for each reference replicon in the sample. These contigs are the ones exceeding the user-defined minimum sequence length, filtered using BBTools (version 38.44). After this initial step, the contigs are mapped to the reference replicons with minimap2 [32] (version 2.22). The metrics are computed through custom python code (see Supplemental Material,

Assembly filtering and mapping) for each replicon in the file provided as input. A detailed description of all reference-based metrics is available in Supplemental Material (see Supplemental Material, LMAS Metrics, Per Reference Metrics).

In addition to the statistics shared with the global metrics, LMAS also calculates the number of mismatches relative to each reference, the COMPASS [9] metrics and two new metrics we propose: LSA and Pls.

LSA represents the fraction of the longest single alignment between a contig and the reference, relative to the reference length. The Pls, or Phred-like score, is a scoring function based on the identity of each aligned contig to the reference replicon. Similarly to the Phred quality score [33], a measure of the quality of the identification of the bases by sequencing, the PIs measures the quality of the assembly of a contig. The formula of PIs is similar to the Phred score formula but uses as the error function the identity of the base in the contig to that of the reference replicon. The formula to obtain the PIs metric per contig is Equation 1.

$$Phred(E) = \begin{cases} -\log(E) \times 10 & \text{if } 0 < E \leq 1 \\ 60 & \text{if E} = 0 \end{cases}$$

where $E = 1 - Identity$

**Equation 1:** Formula for the PIs.

# The LMAS Report

The LMAS results are presented in an interactive HTML. The LMAS report is composed of two main panels: a top summary panel with information on input samples (provided by the user) and the resources used during LMAS' execution, and a bottom panel where selected global and reference specific assembly metrics can be explored for each sample. LMAS constructs the HTML file after workflow completion, storing it in the "reports" folder. The report data can be easily shared between users and requires only a browser for visualization.

**Figure 2: The LMAS report.** All results, and optional text information describing the samples, are grouped in the LMAS report, an interactive and responsive HTML file, for exploration in any browser. Links for LMAS source code and documentation are available in the top right corner of the report. 1) The summary panel of the LMAS report contains information on the input reference sequences and raw sequencing data samples (provided by the user), and the overall computational performance of the assemblers in LMAS. 2) The LMAS metric panel contains the explorable global and reference specific performance metrics per input raw sequencing data sample. The tabular presentation allows direct comparison of exact values between assemblies, and the interactive plots allow for an intuitive overview and easy exploration of results. 3) If an assembler fails to produce an assembly, or fails to assemble sequences that map to the reference replicon, it is marked in the table with a red warning sign. 4) The global or reference replicon specific metrics can be accessed for each sample in the dropdown menu.

## Summary Panel

The top panel of the report contains information on the input samples and overall performance of the assemblers in LMAS, divided into three tabs: Overview, Performance and About us. On the top right corner of the report, direct links to LMAS' source repository and documentation are provided.

- *Overview*: This tab contains information on the input data, including the name and number of reads of the raw sequencing data, and the name of the reference file. Additional information provided by the user about the community used as input is also presented here.
- *Performance*: This tab contains a table with information on the version, the containers used and computational performance metrics for each assembler in LMAS.
- *About us*: This tab contains information on the LMAS GitHub repositories and the LMAS development team

## Metrics Panel

The bottom portion of the report contains the explorable global and reference specific performance metrics per input raw sequencing data sample. Each sample has its own tab and the global or reference replicon specific metrics can be accessed in the dropdown menu.

### Global Metrics

A table displays the global assembly metrics computed for the complete and FS contigs. If an assembler fails to produce an assembly, it is marked on the table with a red warning sign. The global metric plots are interactive, allow zooming in on particular areas and provide extra information as hover text boxes. The plots can be saved as PNG in whatever view the user selects.

### Per Reference Metrics

Similarly to the global assembly metrics, a table displays the computed set of reference restricted metrics for the FS contigs. If an assembler fails to produce sequences that align to the reference, these are marked in the table with a red warning sign. Information on the expected reference replicon length and the GC content is calculated from the input files and reported above the table. The per-reference metric plots are also interactive, allowing the same type of operations as the global metric plots.

## Comparison with other assembly evaluation software programs

The assessment and evaluation of genome assemblies has been a relevant field ever since the emergence of the assembly process itself, and therefore many solutions have been proposed [3,7,9–11,34–36]. The Critical Assessment of Metagenome Interpretation (CAMI) proposed a set of recommendations and best practices for benchmarking in microbiome research [37]. These recommendations include the reporting of computational performance, which may condition the choice of software by the users, such as runtime, disk space and memory consumption, also reported by LMAS (see Supplementary Material, LMAS Metrics). As also suggested by CAMI, LMAS tracks the exact program version and command-line calls through its implementation in Nextflow. Moreover, using containerised assemblers and being easily installable through Bioconda, LMAS facilitates deployment in diverse user machines. Unlike the CAMI tutorial, in which users are asked to download and install the necessary tools,

in LMAS everything is provided in a one-stop reproducible workflow that effortlessly handles all pre-processing, assembly, post-processing, traceability and report production steps, freeing users to focus on providing relevant samples for analysis and interpreting the results in view of the intended applications.

Concerning software for assembly quality assessment currently available, the most widely adopted is QUAST [10], or when dealing with metagenomic data, its extension metaQUAST [11], which was also adopted by the CAMI challenges [3,5] and suggested in the CAMI Tutorial [37]. Although several features of these tools overlap with LMAS' quality assessment components, these differ from LMAS in the sense that they are not a single step workflow allowing a traceable and reproducible assembly of mock communities. Unlike QUAST and metaQUAST, whose purpose is to evaluate assemblies, the purpose of LMAS is to allow users to evaluate assembler performance for a given sample of interest. Supplementary Table S2 shows the comparison of the output and computed assembly quality metrics generated by LMAS, QUAST and metaQUAST.

# Results and Discussion

To illustrate the use of LMAS and evaluate the performance of the chosen assemblers we initially used the eight bacterial genomes and four plasmids of the ZymoBIOMICS Microbial Community Standards as reference. As input we used the raw sequence reads of mock communities with an even and logarithmic distribution of species, from real sequencing runs [38] and simulated read datasets, with and without error, matching the distribution of species in each sample [39]. Our dataset is composed of samples ENN (*in silico* generated evenly distributed without error), EMS (*in silico* generated evenly distributed with Illumina MiSeq error model), ERR2984773 (evenly distributed real Illumina MiSeq sample), LNN (*in silico* generated logarithmically distributed without error), LHS (*in silico* generated logarithmically distributed with Illumina HiSeq error model) and ERR2935805 (logarithmically distributed real Illumina

HiSeq sample) (see Supplemental Table S3). Detailed information about the generation of the input samples is available as Supplemental Material (see Supplemental Materials, ZymoBIOMICS microbial community standards, Supplemental Table S4). To evaluate the reproducibility of an assembler performance, the LMAS workflow was run three times for all samples using default parameters, and the resulting data was processed for each sample (see Supplemental Materials, Assessment of Assembly Success) Supplementary Table S5 to Table S10 present an overview of the average global performance per assembler for each sample in LMAS.

To test assembler performance with an even more complex dataset, we used the 12 strain BMock community standard (accession SRX4901583, real Illumina HiSeq 2500 sample) [40]. This sample includes a non-even distribution of species, with the most abundant replicon having 3093x coverage (*Muricauda* sp. ES.050) and the lowest only 0.1x coverage (*Micromonospora coxensis*) (Supplementary Table S24). For the sake of a less resource-intense evaluation we downsampled to have 20% of the reads available in the original sample and further processed only these. The main challenges of this data set are possibly to assemble the genomes of the three *Micromonospora spp.* and the two *Halomonas spp.* strains, which have an ANIb >0.84 and 0.98, respectively (Supplementary Table S25). The two Marinobacter *spp.* have an ANIb of 0.78.

To represent more realistic samples of a human microbiome study, the Gut-Mix-RR and Gut-Mix-HiLo standards, including 20 species known to be present in the human gut, were used as reference (accessions SRR11487941 and SRR11487935, respectively, both real Illumina MiSeq samples) [41]. For the Gut-Mix-RR, the abundance of each bacterial genome is relatively even (maximum of 66x, an average of 22.32x and a minimum of 5.99x; Supplementary Table S28). The Gut-Mix-HiLo has an uneven abundance of species (maximum of 115x, an average of 20.45x and a minimum of 0.34x; Supplementary Table S28). The genomes in these mock communities are fairly diverse (average ANIb=0.67, Supplementary Table S29), with the two subspecies of *Bifidobacterium longum* (ANIb=0.95)

possibly being the most challenging. It is worth noting that only draft genomes are available for eight of the strains, including one of the *Bifidobacterium longum* subspecies (Supplementary Table S28). The *Roseburia hominis* and *Roseburia intestinalis* are the closest related closed replicons (ANIb>0.77) in this sample.

## Some assemblers perform poorly

Of the 12 *de novo* prokaryotic assemblers included in LMAS, 4 stand out as having an overall poor performance in the ZymoBIOMICS Microbial Community Standards dataset: ABySS, MetaHipmer2, minia and VelvetOptimiser. Both ABySS and MetaHipmer2 performed inconsistently with differing resource requirements for the same sample in different runs, namely run time and memory allocation (see Supplemental Materials, Resource Requirements Differ Greatly, Supplemental Figure S2). Moreover, ABySS failed to produce an assembly for sample ERR2984773 for 1 of the runs (see Supplementary Table S7) and for sample LHS in any of the 3 runs in the time limit of 3 days (see Supplementary Table S9), and MetaHipmer2 failed to produce an assembly for samples LNN and LHS in all 3 runs (see Supplementary Tables S8-S9). VelvetOptimiser generated the highest number of inconsistent contigs across the 3 LMAS runs (Figure 3, Supplementary Table S11), with 1.69% of the total contigs created present in only 1 or 2 runs. Although not as extreme as VelvetOptimiser, ABySS (0.52%), minia (0.14%), GATBMiniaPipeline (0.32%), MetaHipMer2 (0.11%) and IDBA-UD (0.08%) also showed inconsistencies in contig size.

**Figure 3: Assembly reproducibility.** Inconsistent contigs produced per assembler over 3 LMAS runs. The distribution of contig sizes, in basepairs, consistently present in all three LMAS runs are indicated in the grey boxplots for each assembler. If an assembler produced a contig only present in two of the runs (as determined by its size), its size is indicated in teal. If a contig is present in a single run, it is represented in red.

Regarding the quality assessment of the assemblies produced (Figure 4, Supplementary Table S12), ABySS, and minia are the only single k-mer dBg assemblers in the collection and

were found to mostly underperform relative to their multiple k-mer dBg counterparts as reported previously [3,37,42,43], generally resulting in more fragmented assemblies, although there were significant differences in performance across samples. Among multiple k-mer assemblers, VelvetOptimiser frequently produced a very high number of contigs of very small size (over 99% of the contigs not surpassing the minimum length of 1,000 bp) and therefore a low N50 (an average of 29,768 bp versus a global average of 84,114 bp) (Supplementary tables S5-S10). Additionally, ABySS and VelvetOptimizer produced contigs with a very large number of Ns, with an average of 1,019 and 3,035 uncalled bases per assembly, respectively. MetaHipMer2, although having overall average metrics in the two evenly distributed mock samples (ENN and EMS, Supplementary Tables S5-S6) where it was able to run successfully, it severely underperformed in the real samples (ERR2984773 and ERR2935805, Supplementary Tables S7 and S10). Generally, the performance scores of the assemblers decreased considerably for the real samples in comparison with the simulated ones, either with or without error underscoring the importance of using mock samples instead of simulated reads to evaluate assembler performance. High utilization of the reads in the dataset is observed for most assemblers, with on average at least 90% of the reads mapping back to the assembly, except for ABySS, MetaHipMer2 and VelvetOptimiser whose values are in the range of 46-79%. Despite an overall good performance, SPAdes produced the highest number of misassembled contigs in the logarithmically distributed sample, with an average of 98 and a maximum of 572 (sample ERR2935805, Supplementary Table S10), in comparison to the global average of 11 misassembled contigs for all assemblers across all samples. However, this behaviour was not consistent across samples, with the evenly distributed sample showing similar misassembled contigs between SPAdes and other assemblers, similarly to the other mock samples tested (see below).

Due to their poor performance discussed above, the following assemblers have not been included in subsequent analyses: ABySS, MetaHipmer2, minia and VelvetOptimiser.

**Figure 4: Assembler performance for the ZymoBIOMICS Microbial Community Standards dataset.** For each sample in the dataset, the best score of each assembler in the 3 LMAS runs was selected. The results for each global assembly metric was normalised, with 1 representing the best result, and 0 the worst. For the original assembly, the following metrics are presented: number of contigs produced (in blue), number of basepairs produced (in teal), the size of the largest contig assembled (in green), N50 (in yellow), percentage of mapped reads to the assembly (in orange) and uncalled bases (in red). For the filtered assembly, the additional metrics are presented: number of misassembled contigs (in purple) and number of misassembly events (in brown).

## Metagenomic dedicated assemblers do not outperform genomic assemblers

After excluding the poorly performing assemblers, LMAS includes 3 genomic (SKESA, SPAdes and Unicycler) and 4 labelled as metagenomic specific (GATBMiniaPipeline, IDBA-UD, MEGAHIT and metaSPAdes) *de novo* prokaryotic assemblers, all implementing multiple k-mer dBg algorithms. As observed in Figure 5, Supplementary Table S13 and Supplemental Figure S3, there were very significant differences between the best and the worst performing assemblers of each type for the ZymoBIOMICS Microbial Community Standards dataset, with this difference being more pronounced for metagenomic assemblers. The best performing assemblers of each type behaved frequently quite similarly, and the differences between them tended to be attenuated after filtering for contigs <1 kbp. Still, for the linearly distributed samples (ENN, EMS and ERR2984773), the overall worst performers tended to be metagenomic assemblers. In contrast, for the logarithmically distributed samples (LNN, LHS and ERR2935805) the opposite was observed, with genomic assemblers tending to be the worst-performing (Figure 5). For the logarithmically distributed samples, the number of basepairs recovered is significantly lower than expected from their composition for both genomic and metagenomic assemblers, particularly after filtering (Supplementary Table S13), as contigs representing the less abundant species are not recovered by either type of assemblers (see Assembler performance is influenced by replicon abundance in the sample). For this dataset, the fact that an assembler is branded as genomic or metagenomic does not

translate into better or worse performance in dealing with these complex samples, but rather characteristics of the individual assemblers themselves determine their performance.

Figure 5: **Performance of genomic and metagenomic assemblers for the ZymoBIOMICS Microbial Community Standards dataset.** For each sample in the dataset and for the 3 runs, the best and worst scores for each assembler category were selected: genomic (in blue) and metagenomic (in red). The results for each global assembly metric were normalised, with 1 representing the best result, and 0 the worst. For the original assembly, the following metrics are presented: number of contigs produced, number of basepairs produced, the size of the largest contig assembled, N50, percentage of mapped reads to the assembly and uncalled bases. For the filtered assembly, the additional metrics are presented: number of misassembled contigs and number of misassembly events.

In the BMock standard, similarly to the ZymoBIOMICS standard, no significant difference was observed between the genomic and metagenomic assemblers, particularly after filtering for contigs <1 kbp (Supplemental Table S26). Both *Marinobacter* replicons (2615840697 and 2616644829; 448x and 135x coverage, respectively) were successfully recovered by all genomic and metagenomic assemblers with >0.87 breadth of coverage (Supplementary Table S27). The two most abundant *Micromonospora* replicons (2623620557 and 2623620567; 15x and 18x coverage, respectively) were also recovered to a breadth of coverage >0.95 by most assemblers, except the genomic assemblers SKESA and Unicycler (Supplementary Table S27). All assemblers, with the exception of SKESA and GATBMiniaPipeline, recovered both *Halomonas* replicons (2623620617 and 2623620618) with a breadth of coverage of >0.84 (Supplementary Table S27). When considering this set of closely related replicons metagenomic assemblers also did not perform consistently better than genomic assemblers in the number of misassembled contigs or SNPs relative to the reference genome. Among the metagenomic assemblers, GATBMiniaPipeline and IDBA-UD performed particularly well but with values close to those of the two best genomic assemblers (SPAdes and Unicycler). IDBA-UB performed significantly worse with the *Micromonospora* replicons, possibly because of their lower coverage. As could have been expected, the number of SNPs in the lower coverage *Halomonas* replicon was consistently higher than in the one with higher coverage (1.6-fold to 85.0-fold depending on the assembler), despite their relatively modest (12.6%) difference in

estimated coverage in the sample and very significant depth of coverage (>500x) (Supplementary Tables S24 and S27). However, when comparing the number of SNPs in the *Marinobacter* replicons, this relationship is reversed, with the replicon with higher coverage (448x) having more SNPs relative to the reference than the one with lower coverage (135x) (Supplementary Table S27). This indicates that other factors, such as characteristics of the replicon, the actual representation in the sample and the closeness to other replicons in the sample may influence the performance of assemblers. It is also interesting to see that the contigs generated by themselves do not allow the distinction of closely related strains, since the number of SNPs relative to the reference genomes (if the assembler is able to cover >0.79 of the genome), are in the ranges of 315-9,915 and 9,773-70,196 for the higher and lower depth of coverage *Halomonas* replicons, respectively.

For the Gut-Mix-RR and Gut-HiLo-RR mock communities, the same pattern was observed as with the other mock communities, with the differences between the metagenomic and genomic assemblers being attenuated after filtering for contigs <1 kbp (Supplemental Table S30 and S31). Particularly for the evenly distributed Gut-Mix-RR sample, when considering the subset of *Roseburia spp.*, the replicon with the lowest coverage (12x for *R. intestinalis* versus 18x for *R. hominis*) had a consistently higher number of SNPs, with the exception of the SKESA assemblies, where the opposite was observed. This is similar to what was observed for the *Halomonas* replicons in the BMock12 community standard.

## Success is not straightforward

Several factors contribute to suboptimal performance of the assembly process, from DNA isolation and library preparation protocol; sequencing technology, depth and read length; to possible contamination and inherent characteristics of the sample composition.

## Assembler performance is influenced by species

For the eight bacterial genomes present in the ZymoBIOMICS Microbial Community Standards dataset samples, even in those with an even distribution of the genomes (ENN, EMS and ERR2984773), variations in the assembly metrics were observed (Figure 6, Supplemental Figures S4-S6, Supplemental Tables S14-S16). For all samples in the dataset, the genomes are recovered almost completely, with all replicons being >90% represented in the resulting assemblies. *Lactobacillus fermentum* is the least represented genome (92.2%-94.9%). Most replicon sequences are recovered in <100 contigs, except for *Pseudomonas aeruginosa*, *Escherichia coli* and *Salmonella enterica*, and not considering IDBA-UB, which frequently produces a larger number of contigs when compared to other assemblers. However, in other mock samples these worse performance of IDBA-UD in terms of number of contigs is not so clear. The absolute values of other metrics of assembly quality, such as LSA, misassembly events or uncalled bases, are also different between bacterial genomes (Supplemental Tables S14-S16). The fact that *S. enterica* is a closely related species to *E. coli*, with high level of genetic similarity (ANIb >0.8, Supplemental Table S23), could have created difficulties for resolving the assemblies in a mixed sample and lead to the lower coverage observed, the higher number of contigs and the increased number of misassembled contigs identified in these species in some samples. However, in the case of the larger number of contigs of *P. aeruginosa*, no related species are present in the sample and these possibly reflect intrinsic properties of the replicon such as a high number of prophages integrated in the bacterial genome [44]. Similarly, replicon characteristics could be behind the lower breadth of coverage consistently observed in *L. fermentum* assemblies.

**Figure 6: Genome fragmentation for each reference replicon of the ZimoBIOMICS community standards dataset for the evenly distributed samples.** Genome fragmentation for the 3 LMAS runs is represented by the number of contigs and breadth of coverage of the reference per assembler for the evenly distributed samples: ENN (evenly distributed without error model, identified by a circle), EMS (evenly distributed with Illumina MiSeq error model, identified by a square) and ERR2984773 (real Illumina MiSeq sample, identified by a diamond). Each assembler is identified with the following colour scheme - dark blue: Unicycler, light blue: SPAdes, dark green: SKESA, light green: metaSPAdes, yellow: MEGAHIT, orange: IDBA-UD, red: GATBMiniaPipeline.

In the BMock12 and the Gut-Mix samples, which have pairs of much more similar replicons, it is true that the closely related replicons do have higher numbers of contigs. However, it is possible that the properties of the individual replicons also have an impact on the number of contigs generated by the assemblers (Supplemental Figure S8). Another potential confounder which was not explored is the length of the reads, with Miseq samples having 300bp reads and HiSeq samples having 150bp reads.

## Longer contigs have higher confidence

The PIs metric, which measures the error rate of a contig relative to the reference, shows that for every replicon, longer contigs have higher PIs (Figure 7). This could justify the option of filtering an assembly by length, even beyond the 1000 bp minimum contig size implemented by default in LMAS. Not only are we eliminating shorter, less informative contigs in terms of genetic context, but these are also the ones most likely to contain errors relative to the reference sequence.

**Figure 7: Phred-like score (PIs) per contig for each reference replicon of the ZimoBIOMICS community standards datasets.** The PIs score was calculated for each unique contig produced by each assembler in 3 LMAS runs and is represented in relation to its contig size. Each contig is coloured according to the assembler with the following colour scheme - dark blue: Unicycler, light blue: SPAdes, dark green: SKESA, light green: metaSPAdes, yellow: MEGAHIT, orange: IDBA-UD, red: GATBMiniaPipeline.

## Certain genomic regions are problematic for all assemblers

Some genomic regions in several replicons are consistently a challenge for all assemblers. As observed in Figure 8, all genomes of the ZymoBIOMICS Microbial Community Standards dataset present certain regions that fail to assemble for all tools in all runs, even those generating high-quality draft assemblies. Of all seven assemblers considered, only GATBMiniaPipeline, MEGAHIT and IDBA-UD showed inconsistency in the gaps produced over the 3 LMAS runs (Supplemental Table S17), as expected from producing variable sets of contigs. The regions consistently missing for all assemblers in all runs are rich in repetitive

elements, such as rRNA and tRNA coding sequences and mobile genetic elements (Supplemental Table S18), with larger gaps corresponding to tandem sets of these elements. This reflects an intrinsic limitation of short-read sequencing since the length of a read pair is not enough to bridge across the repetitive element, preventing the generation of contigs representing these regions. This is something that could be addressed by the use of long-read sequencing technologies. Despite this, some assemblers are able to produce contigs that represent some of these large tandem regions, such as MEGAHIT and SKESA for *E. faecalis,* and IDBA-UD, MEGAHIT and metaSPADES for *L. monocytogenes*, but such performance is not consistent for all reference replicons. For instance, SKESA fails to assemble two large regions of the *S. enterica* genome that all other assemblers successfully cover.

Figure 8: **Location of gaps in comparison to the reference sequence, per assembler, for each reference replicon of the ZimoBIOMICS community standards datasets.** The resulting plot contains the consistent gaps obtained from a three LMAS run for the evenly distributed dataset (ENN, EMS and ERR2984773) for GATBMiniaPipeline, IDBA-UD, MEGAHIT, metaSPAdes, SKESA, SPAdes and Unicycler assemblers.

For the BMock12 community standard, the same pattern of consistency of difficult regions across replicons can be observed for all replicons, with the exception of the lowest abundance replicons (see Supplemental Figure S9). Interestingly, the two closely related *Halomonas* replicons present a very dissimilar gap pattern, and with a high number of gaps (n=2789 and n=2702) distributed throughout the replicon sequence, which possibly reflects the difficulty of assembling closely related replicons in the same sample.

## Assembler performance is influenced by replicon abundance in the sample

The logarithmically distributed samples (LNN, LHS and ERR2935805) of the ZymoBIOMICS community standard dataset showed greater variation in the assembly success metrics than the evenly distributed samples (Supplementary Table S8-S10), reflecting the difficulty of recovering sequences of the lowest abundant replicons. For the three replicons with an

estimated depth of coverage >15x, a similar pattern is observed in logarithmically distributed samples as in evenly distributed samples, albeit with greater dispersion in the number of contigs generated and with a markedly decreased breath of coverage for some assemblers and samples in the logarithmically distributed samples (Figure 6 and Supplementary Figure S7). Almost no contigs >1000 bp were retrieved for replicons with an estimated depth of coverage of <2x resulting in a very low breadth of coverage (<1%) (Supplementary Table S4, Supplementary Table S22). This leads to a severe underrepresentation of the diversity of the community in the generated contigs, particularly of plasmid sequences due to their smaller length and abundance as was described previously [3,45,46]. This happens despite the greater sequencing depth of these samples versus those with an even distribution (>5-fold difference in the number of reads).

For the BMock12 community standard, the very low replicon abundance (*Micromonospora coxensis*, 2623620609, 0.02x coverage), fails to assemble in all tools (Supplemental Figure S9, Supplemental table S24). The *Micromonospora echinaurantiaca* (2623620557, 14.9x coverage) and *Micromonospora echinofusca* (2623620567, 18.2x coverage) fail to assemble with SKESA and Unicycler, and the *Propionibacteriaceae* replicon (2615840646, 31.9x coverage) fails to be assembled with SKESA. In the Gut-Mix-RR standards there are several replicons with <1x to 20x depth of coverage. Significant breath of coverage (>0.7) was obtained with the contigs created by most assemblers, including successful assemblies of some of the replicons by SKESA and Unicycler that had failed with higher coverage replicons in the BMock12 standard. Taking together these data and that of the ZymoBIOMICS standard, these suggest that it is hard to establish a universal breakpoint at which each of the assemblers is able to generate high breadth of coverage contigs, with the actual genome of interest and the composition of the sample possibly playing a role. Nevertheless, coverages >15x result in high breadth of coverage contigs, albeit in the lower range with many contigs and a significant number of SNPs.

# Conclusions

The purpose of LMAS is to empower users to test assembler performance in meaningful conditions for their experimental setup and objectives. Suitable mock communities, reproducing the users' samples of interest, can be used as a gold standard to evaluate assembler performance. To illustrate LMAS' functionalities we analysed three well-known samples used in several studies. Although the eight species ZymoBIOMICS Microbial Community Standards might not be representative of the metagenomic complexity of the samples of interest of most researchers, we hoped that its relative simplicity meant that the results shown would represent a best-case scenario, since as sample complexity increases so do the challenges to assembler performance. However, the results of the BMock12 and Gut-Mix community standards suggest that the actual genome of interest and community composition play an important part in the results of individual assemblers.

Our results showed significant differences in both global and reference-dependent assembly quality metrics generated by each *de novo* assembler. The performance of each assembler varied depending on the species of interest and its abundance in the sample, with less abundant species presenting a significant challenge for all assemblers. The fact that an assembler is branded as specific for metagenomics does not guarantee a better performance in metagenomic samples, with assemblers used for genomic assembly outperforming the worst metagenomic assembler tested. Even when considering communities with very similar replicons, the overall performance of metagenomic assemblers was not consistently better than that of genomic assemblers. The results also indicate that the recovery of assemblies allowing strain-level discrimination at the SNP level is highly unlikely based solely on the assembler generated contigs. The following assemblers showed significant performance problems and their usability may be limited, at least with the default parameters we used: ABySS, MetaHipmer2, minia and VelvetOptimiser.

The choice of de novo assembler depends greatly on the computational resources available, the species of interest, its representation in the sample, and, possibly, the composition of the community in the sample. In our testing with any of the mock communities, no assembler stood out as an undisputed all-purpose choice for short-read metagenomic prokaryotic genome assembly, with different assemblers showing specific strengths. Users would thus benefit from analysing the results of sequencing mock communities (ideally) or of artificially generated reads simulating their samples of interest to guide their choice of assembler. LMAS was developed to be an easy to use and flexible tool for this purpose. From the results that we obtained with various mock communities, the following assemblers performed consistently well (presented in alphabetical order): MEGAHIT, metaSPAdes, SKESA, SPAdes and Unicycler. From our assessment, we conclude that these assemblers are the most likely candidates to perform well in other complex samples.

LMAS was built with modularity and containerization as keystones, leveraging the parallelization of processes and guaranteeing reproducibility across platforms. The modular design allows for new assemblers to be easily added and existing assemblers to be easily updated, allowing LMAS to function as a continuous benchmarking platform and ensuring its future relevance as improvements in assembly software are proposed. LMAS will also support evaluating the gains of any cumulative improvements to existing assemblers using the same benchmark set adapted to a specific project or goal. Such reproducibility, capacity to easily add assemblers of interest not included in the current version and flexibility for future extensions are important principles in computational method benchmarking. Moreover,Additionally, by lowering the barriers to perform comparisons between assemblers, LMAS will encourage users mayto compare software performance against mock communities of special interest, depending on their operational focus.

The interactive report provides an intuitive platform for data exploration, allowing the user to easily sift through global and reference specific performance metrics for each sample, as well as providing information on the assemblers executed to allow traceability of the results.

Producing an extensive, metric rich report allows users interested in different aspects of assembler performance to make informed decisions, particularly when choosing among the top-performing assemblers, which show only minor differences.

LMAS applies several well-known assembly metrics and proposes two more: LSA, which represents the fraction of the longest single alignment between a contig and the reference, and Pls, a scoring function based on the identity of each aligned contig to the reference replicon. The entire set of assembly quality metrics used in LMAS allows not only the assessment of quality based on statistics inherent to a set of assembled contigs but also a comparison to a ground truth provided using samples of known composition and reference sequences. The LMAS report provides an interactive and intuitive platform for the exploration of these results, allowing users to easily test assemblers in mock samples with species composition and distribution relevant for their own studies.

Although computationally intensive due to the complex nature of the *de novo* assembly process, LMAS is the only software integrating assembly and its evaluation into a single pipeline, guaranteeing the same conditions are met for all tools. With LMAS, it is now possible to continuously evaluate which *de novo* assembler produces the most relevant results for a given community of interest. The LMAS workflow is open-source and its code and documentation are available at https://github.com/B-UMMI/LMAS and https://lmas.readthedocs.io/ respectively.

# Availability of supporting source code and requirements

**Project name:** LMAS

**Project home page:** https://github.com/B-UMMI/LMAS

**Operating system(s):** UNIX-like systems.

**Programming languages:** Nextflow, Python, Bash, Javascript

**Other requirements:** Java version 8 or highest. Docker/Singularity/Shifter

**License:** GNU GPL v3

**RRID:** SCR_022251

# List of abbreviations

Bp - Basepairs

cgMLST - core-genome multilocus sequence typing

dBg - de Bruijn graphs

FS - Filtered set

GB - gigabytes

HPCs - high-performance computing clusters

LSA - Longest single alignment

OLC - overlap-layout-consensus

Pls - Phred-like score

SNPs - single nucleotide polymorphisms

# Declarations

## Ethics approval and consent to participate

Not applicable.

## Consent for publication

Not applicable.

## Availability of data and material

The datasets analysed during the current study are available in the Zenodo repository, under [48]. All supplemental material is available in the Zenodo repository, under [49]. . Likewise, all figures in the current manuscript are available in their original format in the Zenodo repository, under [50]. Real sequencing data of the ZymoBIOMICS Microbial Community Standards is available under accessions ERR2984773 and ERR2935805 [38]. All data generated or analysed during this study are included in this published article, its supplementary information files and the data analysis repository located at [47]. Additionally, the reports for the ZymoBIOMICS Microbial Community Standard, BMock12 Community Standard and NIBSC Gut DNA Reference are available at [51], [52] and [53],respectively.

An archival copy of the github repository (https://github.com/B-UMMI/LMAS) is also available via the GigaScience database, GigaDB [54].

## Competing interests

MR received honoraria for serving on the speakers' bureau of Pfizer and Merck Sharp and Dohme and for participating in expert panels of GlaxoSmithKline and Merck Sharp and Dohme. The other authors declare that they have no competing interests.

## Funding

C.I.M. was supported by the Fundação para a Ciência e Tecnologia (grant SFRH/BD/129483/2017).

## Author's contributions

C.I.M., M.R. designed the workflow. C.I.M implemented and optimised the workflow, created the Docker containers, generated mock shotgun metagenomics data used to test and validate the workflow, contributed to the development of the HTML report and analysed the data. C.I.M. and M.R. wrote the manuscript. P.V.C. contributed to the development of the HTML report. M.R., J.A.C. Y.M, and J.M.G critically revised the manuscript. All authors read, commented on, and approved the final manuscript.

## Acknowledgements

The authors would like to thank Rafael Mamede for his contribution to the implementation and commentary on the several interactive plots implemented throughout the LMAS report. The authors would also like to thank Nabil Fareed-Alikan for his insightful commentary on the interpretation of the results reported in this manuscript, and Anthony Underwood and Robert A. Petit III for their assistance in building the LMAS Nextflow workflow. The author would also like to thank Samuel Nicholls, Joshua Quick, Shuiquan Tang and Nicholas Loman for publicly providing the sequencing data for the ZymoBIOMICS Microbial Community Standards. Likewise, the authors would like to thank Volkan Sevim, Juna Lee, Robert Egan, Alicia Clum, Hope Hundley, Janey Lee, R. Craig Everroad, Angela M. Detweiler, Brad M. Bebout, Jennifer Pett-Ridge, Markus Göker, Alison E. Murray, Stephen R. Lindemann, Hans-Peter Klenk, Ronan O'Malley, Matthew Zane, Jan-Fang Cheng, Alex Copeland, Christopher Daum, Esther Singer & Tanja Woyke for providing the sequencing data for the BMock12 Commynity Standard, and to Gregory C. A. Amos, Alastair Logan, Saba Anwar, Martin

Fritzsche, Ryan Mate, Thomas Bleazard & Sjoerd Rijpkema for providing the sequencing data of the NIBSC Gut DNA Reference Gut-Mix-RR and Gut-Mix-HiLo Community Standard.

# References


1. Angers-Loustau A, Petrillo M, Bengtsson-Palme J, Berendonk T, Blais B, Chan K-G, et al.. The challenges of designing a benchmark strategy for bioinformatics pipelines in the identification of antimicrobial resistance determinants using next generation sequencing technologies. *F1000Res*. 2018; doi: 10.12688/f1000research.14509.2.

2. Gruening B, Sallou O, Moreno P, da Veiga Leprevost F, Ménager H, Søndergaard D, et al.. Recommendations for the packaging and containerizing of bioinformatics software. *F1000Res*. 2019; doi: 10.12688/f1000research.15140.2.

3. Sczyrba A, Hofmann P, Belmann P, Koslicki D, Janssen S, Dröge J, et al.. Critical Assessment of Metagenome Interpretation—a benchmark of metagenomics software. *Nat Methods*. 2017; doi: 10.1038/nmeth.4458.

4. Couto N, Schuele L, Raangs EC, Machado MP, Mendes CI, Jesus TF, et al.. Critical steps in clinical shotgun metagenomics for the concomitant detection and typing of microbial pathogens. *Sci Rep*. 2018; doi: 10.1038/s41598-018-31873-w.

5. Meyer F, Fritz A, Deng Z-L, Koslicki D, Gurevich A, Robertson G, et al.. Critical Assessment of Metagenome Interpretation - the second round of challenges. Bioinformatics; 2021 Jul.

6. Ayling M, Clark MD, Leggett RM. New approaches for metagenome assembly with short reads. *Briefings in Bioinformatics*. 2020; doi: 10.1093/bib/bbz020.

7. Olson ND, Treangen TJ, Hill CM, Cepeda-Espinoza V, Ghurye J, Koren S, et al.. Metagenomic assembly through the lens of validation: recent advances in assessing and improving the quality of genomes assembled from metagenomes. *Briefings in Bioinformatics*. 2019; doi: 10.1093/bib/bbx098.

8. Teeling H, Glockner FO. Current opportunities and challenges in microbial metagenome analysis--a bioinformatic perspective. *Briefings in Bioinformatics*. 2012; doi: 10.1093/bib/bbs039.

9. Bradnam KR, Fass JN, Alexandrov A, Baranay P, Bechner M, Birol I, et al.. Assemblathon 2: evaluating de novo methods of genome assembly in three vertebrate species. *GigaSci*. 2013; doi: 10.1186/2047-217X-2-10.

10. Gurevich A, Saveliev V, Vyahhi N, Tesler G. QUAST: quality assessment tool for genome assemblies. *Bioinformatics*. 2013; doi: 10.1093/bioinformatics/btt086.

11. Mikheenko A, Saveliev V, Gurevich A. MetaQUAST: evaluation of metagenome assemblies. *Bioinformatics*. 2016; doi: 10.1093/bioinformatics/btv697.

12. Di Tommaso P, Chatzou M, Floden EW, Barja PP, Palumbo E, Notredame C. Nextflow enables reproducible computational workflows. *Nat Biotechnol*. 2017; doi: 10.1038/nbt.3820.

13. Merkel D. Docker: Lightweight Linux Containers for Consistent Development and Deployment. *Linux J*. Houston, TX: Belltown Media; 20142014;

14. : Lmas :: Anaconda.org. https://anaconda.org/bioconda/lmas Accessed 2022 Apr 6.

15. Mendes I, Vila-Cerqueira P, Ramirez M: LMAS: Last (Meta)genomic Assembler Standing. https://github.com/B-UMMI/LMAS (2021). Accessed 2022 Apr 4.

16. : LMAS's — LMAS 0.1 documentation. https://lmas.readthedocs.io/en/latest/index.html Accessed 2022 Apr 4.

17. : Basic Usage — LMAS 0.1 documentation. https://lmas.readthedocs.io/en/latest/user/basic_usage.html Accessed 2022 Apr 4.

18. : Parameters — LMAS 0.1 documentation. https://lmas.readthedocs.io/en/latest/user/parameters.html Accessed 2022 Apr 4.

19. Jackman SD, Vandervalk BP, Mohamadi H, Chu J, Yeo S, Hammond SA, et al.. ABySS 2.0: resource-efficient assembly of large genomes using a Bloom filter. *Genome Res*. 2017; doi: 10.1101/gr.214346.116.

20. : GATB/gatb-minia-pipeline. https://github.com/GATB/gatb-minia-pipeline (2022). Accessed 2022 Apr 4.

21. Peng Y, Leung HCM, Yiu SM, Chin FYL. IDBA-UD: a de novo assembler for single-cell and metagenomic sequencing data with highly uneven depth. *Bioinformatics*. 2012; doi: 10.1093/bioinformatics/bts174.

22. Li D, Liu C-M, Luo R, Sadakane K, Lam T-W. MEGAHIT: an ultra-fast single-node solution for large and complex metagenomics assembly via succinct de Bruijn graph. *Bioinformatics*. 2015; doi: 10.1093/bioinformatics/btv033.

23. Georganas E, Egan R, Hofmeyr S, Goltsman E, Arndt B, Tritt A, et al.. Extreme Scale De Novo Metagenome Assembly. *SC18: International Conference for High Performance Computing, Networking, Storage and Analysis*. Dallas, TX, USA: IEEE;

24. Nurk S, Meleshko D, Korobeynikov A, Pevzner PA. metaSPAdes: a new versatile metagenomic assembler. *Genome Res*. 2017; doi: 10.1101/gr.213959.116.

25. Chikhi R, Rizk G. Space-efficient and exact de Bruijn graph representation based on a Bloom filter. *Algorithms Mol Biol*. 2013; doi: 10.1186/1748-7188-8-22.

26. Souvorov A, Agarwala R, Lipman DJ. SKESA: strategic k-mer extension for scrupulous assemblies. *Genome Biol*. 2018; doi: 10.1186/s13059-018-1540-z.

27. Bankevich A, Nurk S, Antipov D, Gurevich AA, Dvorkin M, Kulikov AS, et al.. SPAdes: A New Genome Assembly Algorithm and Its Applications to Single-Cell Sequencing. *Journal of Computational Biology*. 2012; doi: 10.1089/cmb.2012.0021.

28. Wick RR, Judd LM, Gorrie CL, Holt KE. Unicycler: Resolving bacterial genome assemblies from short and long sequencing reads. Phillippy AM, editor. *PLoS Comput Biol*. 2017; doi: 10.1371/journal.pcbi.1005595.

29. Seemann T: VelvetOptimiser: automate your Velvet assemblies. https://github.com/tseemann/VelvetOptimiser (2021). Accessed 2022 Apr 4.

30. : Short-Read (Meta)Genomic Assemblers — LMAS 0.1 documentation. https://lmas.readthedocs.io/en/latest/user/assemblers.html Accessed 2022 Apr 4.

31. : Add Assembler Process — LMAS 0.1 documentation. https://lmas.readthedocs.io/en/latest/dev/add_process.html Accessed 2022 Apr 4.

32. Li H. Minimap2: pairwise alignment for nucleotide sequences. Birol I, editor. *Bioinformatics*. 2018; doi: 10.1093/bioinformatics/bty191.

33. Ewing B, Green P. Base-Calling of Automated Sequencer Traces Using Phred. II. Error Probabilities. *Genome Res*. 1998; doi: 10.1101/gr.8.3.186.

34. Manchanda N, Portwood JL, Woodhouse MR, Seetharam AS, Lawrence-Dill CJ, Andorf CM, et al.. GenomeQC: a quality assessment tool for genome assemblies and gene structure annotations. *BMC Genomics*. 2020; doi: 10.1186/s12864-020-6568-2.

35. Meader S, Hillier LW, Locke D, Ponting CP, Lunter G. Genome assembly quality: assessment and improvement using the neutral indel model. *Genome Res*. 2010; doi: 10.1101/gr.096966.109.

36. Challis R, Richards E, Rajan J, Cochrane G, Blaxter M. BlobToolKit – Interactive Quality Assessment of Genome Assemblies. *G3 Genes|Genomes|Genetics*. 2020; doi: 10.1534/g3.119.400908.

37. Meyer F, Lesker T-R, Koslicki D, Fritz A, Gurevich A, Darling AE, et al.. Tutorial: assessing metagenomics software with the CAMI benchmarking toolkit. *Nat Protoc*. Nature Publishing Group; 2021; doi: 10.1038/s41596-020-00480-3.

38. Nicholls SM, Quick JC, Tang S, Loman NJ. Ultra-deep, long-read nanopore sequencing of mock microbial community standards. *GigaScience*. 2019; doi: 10.1093/gigascience/giz043.

39. Gourlé H, Karlsson-Lindsjö O, Hayer J, Bongcam-Rudloff E. Simulating Illumina metagenomic data with InSilicoSeq. Hancock J, editor. *Bioinformatics*. 2019; doi: 10.1093/bioinformatics/bty630.

40. Sevim V, Lee J, Egan R, Clum A, Hundley H, Lee J, et al.. Shotgun metagenome data of a defined mock community using Oxford Nanopore, PacBio and Illumina technologies. *Sci Data*. Nature Publishing Group; 2019; doi: 10.1038/s41597-019-0287-z.

41. Amos GCA, Logan A, Anwar S, Fritzsche M, Mate R, Bleazard T, et al.. Developing standards for the microbiome field. *Microbiome*. 2020; doi: 10.1186/s40168-020-00856-3.

42. Xavier BB, Sabirova J, Pieter M, Hernalsteens J-P, de Greve H, Goossens H, et al.. Employing whole genome mapping for optimal de novo assembly of bacterial genomes. *BMC Research Notes*. 2014; doi: 10.1186/1756-0500-7-484.

43. Mahadik K, Wright C, Kulkarni M, Bagchi S, Chaterji S. Scalable Genome Assembly through Parallel de Bruijn Graph Construction for Multiple k-mers. *Sci Rep*. Nature Publishing Group; 2019; doi: 10.1038/s41598-019-51284-9.

44. Johnson G, Stark N, Wolfe AJ, Putonti C. Complete Genome Sequence of a Pseudomonas aeruginosa Isolate from a Kidney Stone. *Microbiology Resource Announcements*. American Society for Microbiology (ASM); 2019; doi: 10.1128/MRA.01073-19.

45. Meyer F, Fritz A, Deng Z-L, Koslicki D, Lesker TR, Gurevich A, et al.. Critical Assessment of Metagenome Interpretation: the second round of challenges. *Nat Methods*. 2022; doi: 10.1038/s41592-022-01431-4.

46. Fritz A, Hofmann P, Majda S, Dahms E, Dröge J, Fiedler J, et al.. CAMISIM: simulating metagenomes and microbial communities. *Microbiome*. 2019; doi: 10.1186/s40168-019-0633-6.

47. : LMAS Manuscript Analysis. https://github.com/B-UMMI/LMAS_Manuscript_Analysis (2022). Accessed 2022 Apr 4.

48. LMAS Test Dataset. Zenodo repository. https://doi.org/10.5281/zenodo.4588969

49. LMAS Supplemental Material Zenodo repository. https://doi.org/10.5281/zenodo.6623457

50. LMAS figures. Zenodo repository. https://doi.org/10.5281/zenodo.6783042

51. LMAS Test Dataset - ZymoBIOMICS Microbial Community Standards. Zenodo repository. https://doi.org/10.5281/zenodo.7088960

52. LMAS Test Dataset - BMock12 Community Standards. Zenodo repository. https://doi.org/10.5281/zenodo.7092431

53. LMAS Test Dataset - NIBSC Gut DNA Reference. . Zenodo repository.https://doi.org/10.5281/zenodo.7092693

54.
Mendes CI; Vila-Cerqueira P; Motro Y; Moran-Gilad J; Carriço JA; Ramirez M (2022): Supporting data for "LMAS: Evaluating metagenomic short de novo assembly methods through defined communities" GigaScience Database. 2022. http://dx.doi.org/10.5524/102335

Figure 1

Click here to access/download;Figure;Figure 1.pdf

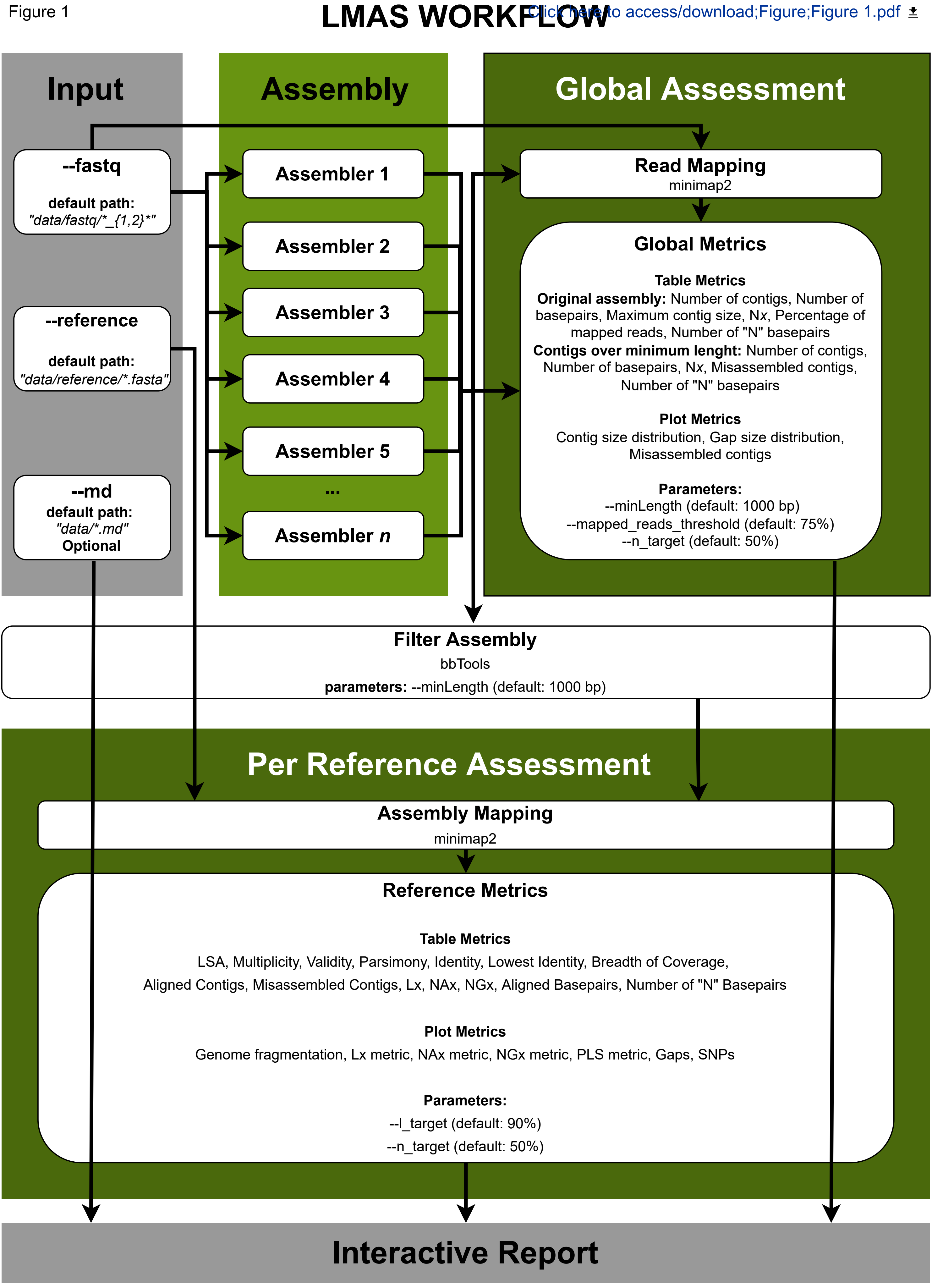

Figure 2

Click here to access/download;Figure;Figure 2.pdf

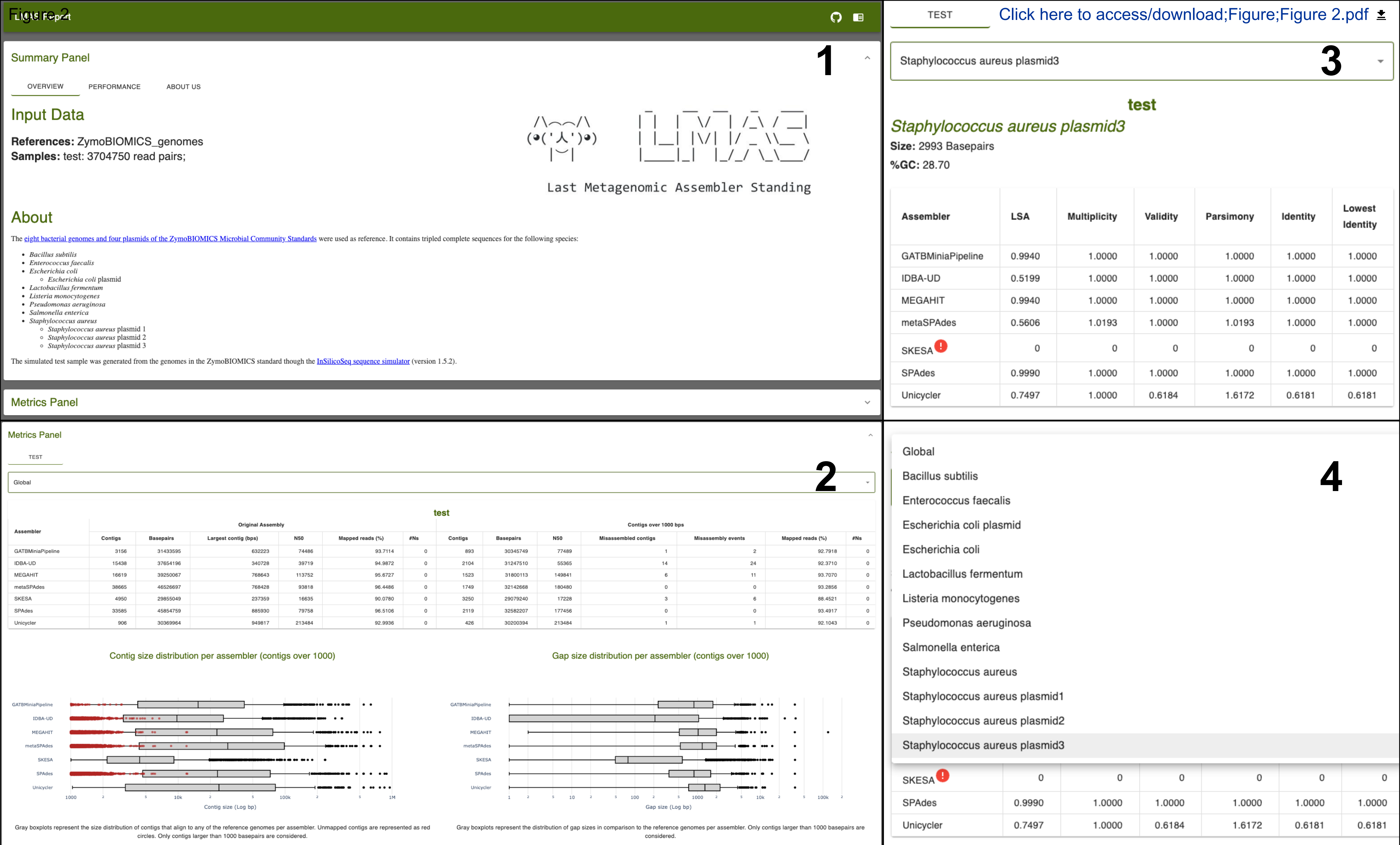

Figure 3 Click here to access/download;Figure;Figure 3.png

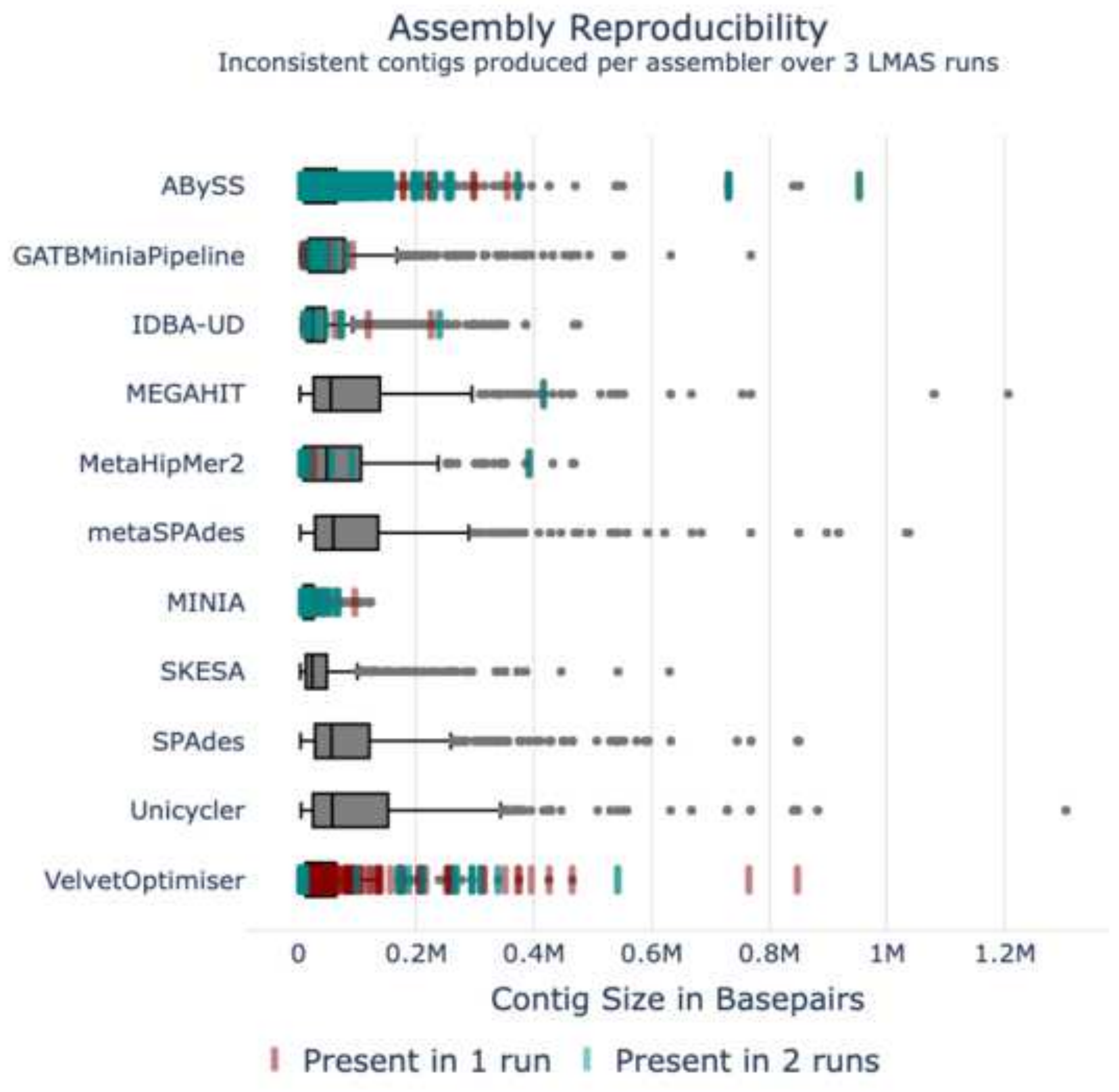

Figure 4

Click here to access/download;Figure;Figure 4.pdf

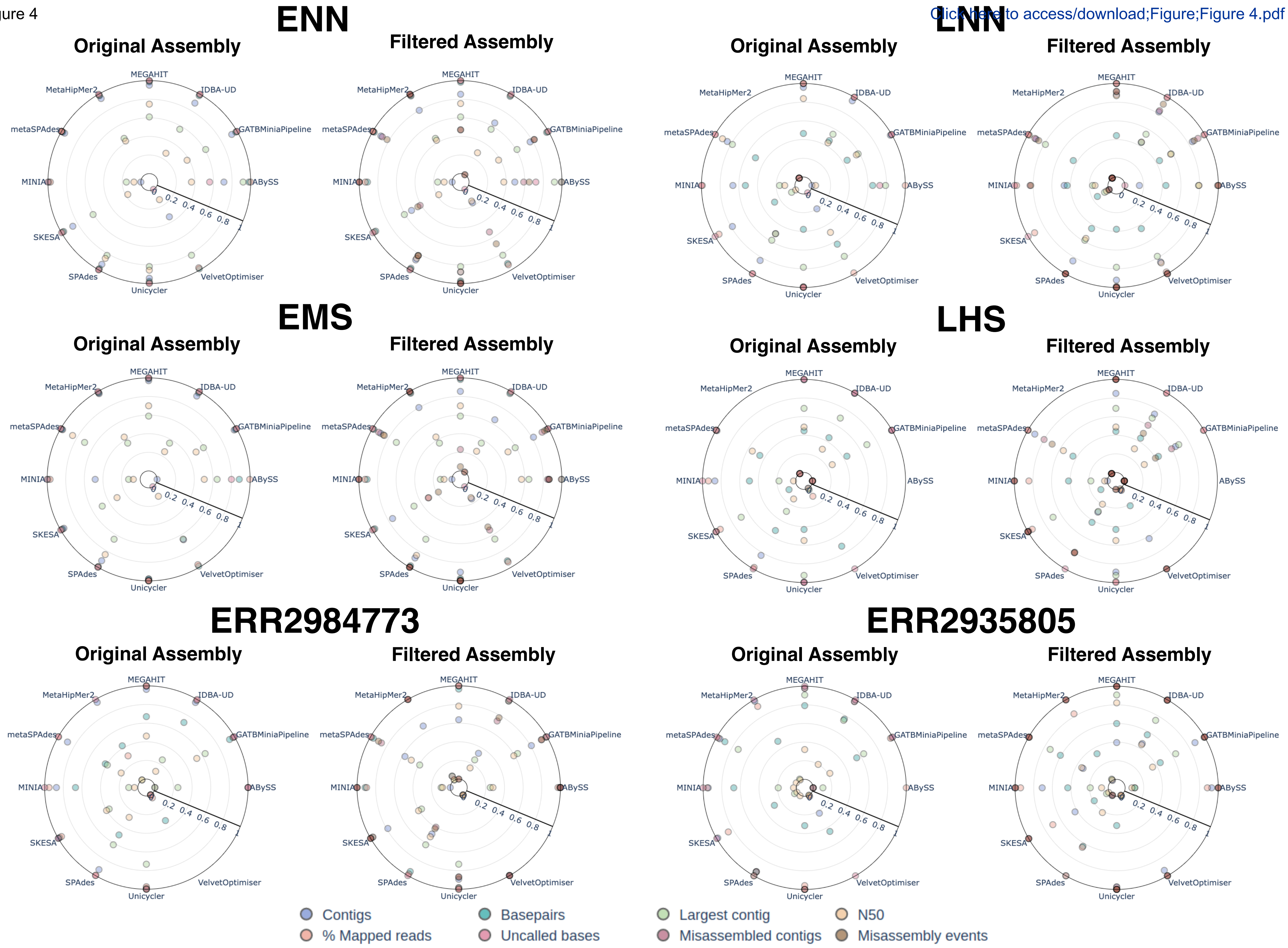

Figure 5

Click here to access/download;Figure;Figure 5.pdf

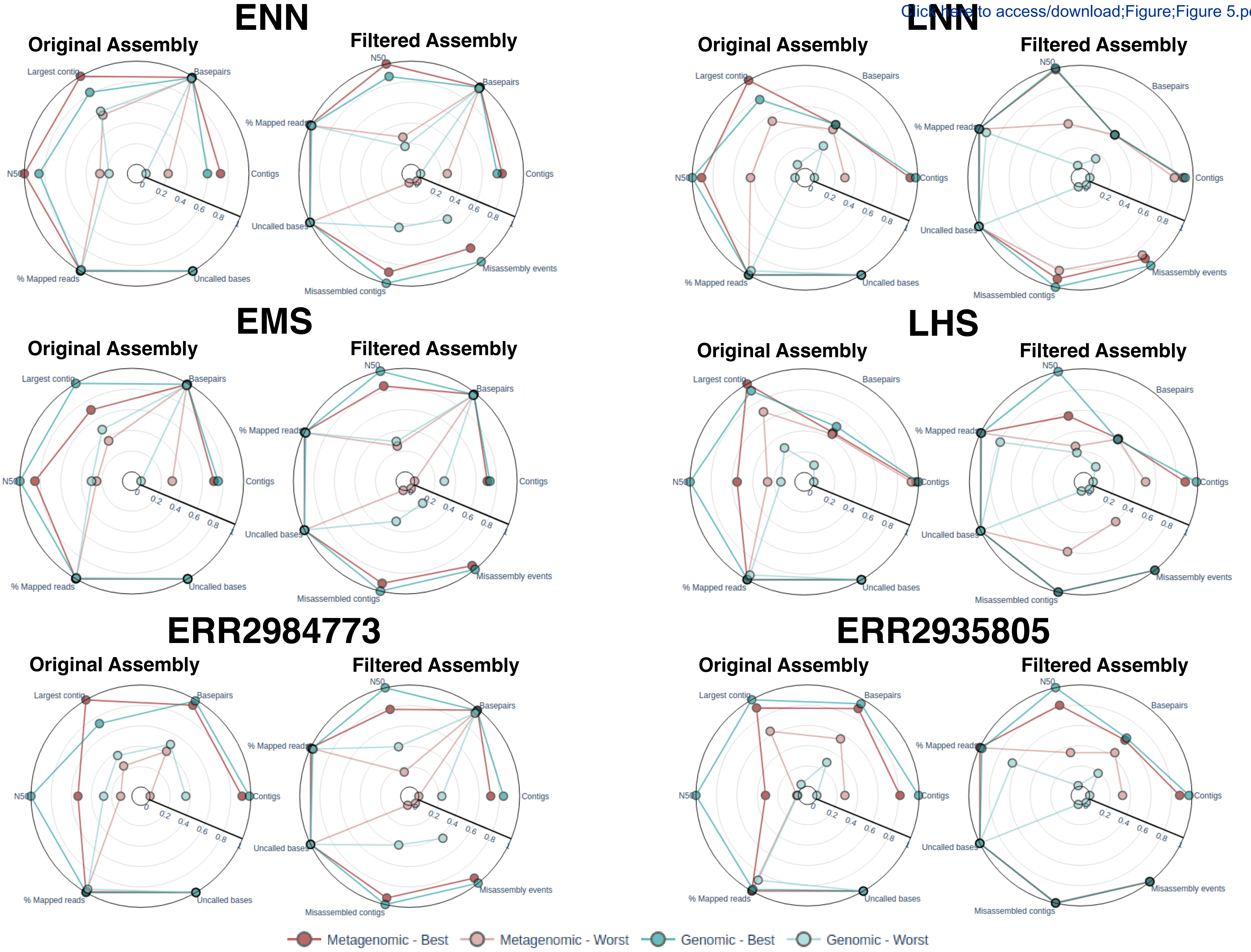

Figure 6

Click here to access/download;Figure;Figure 6.pdf

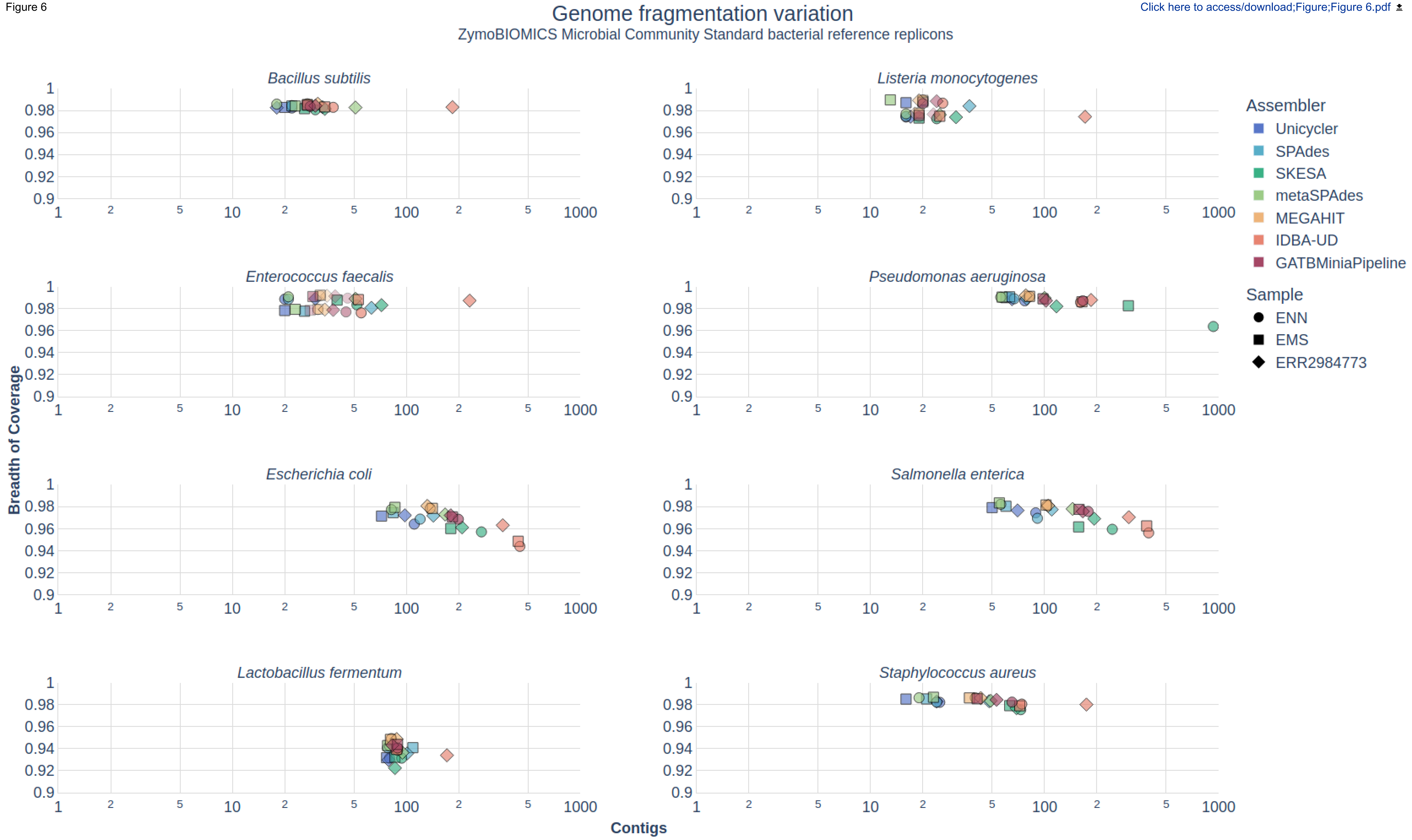

Figure 7

Click here to access/download;Figure;Figure 7.png

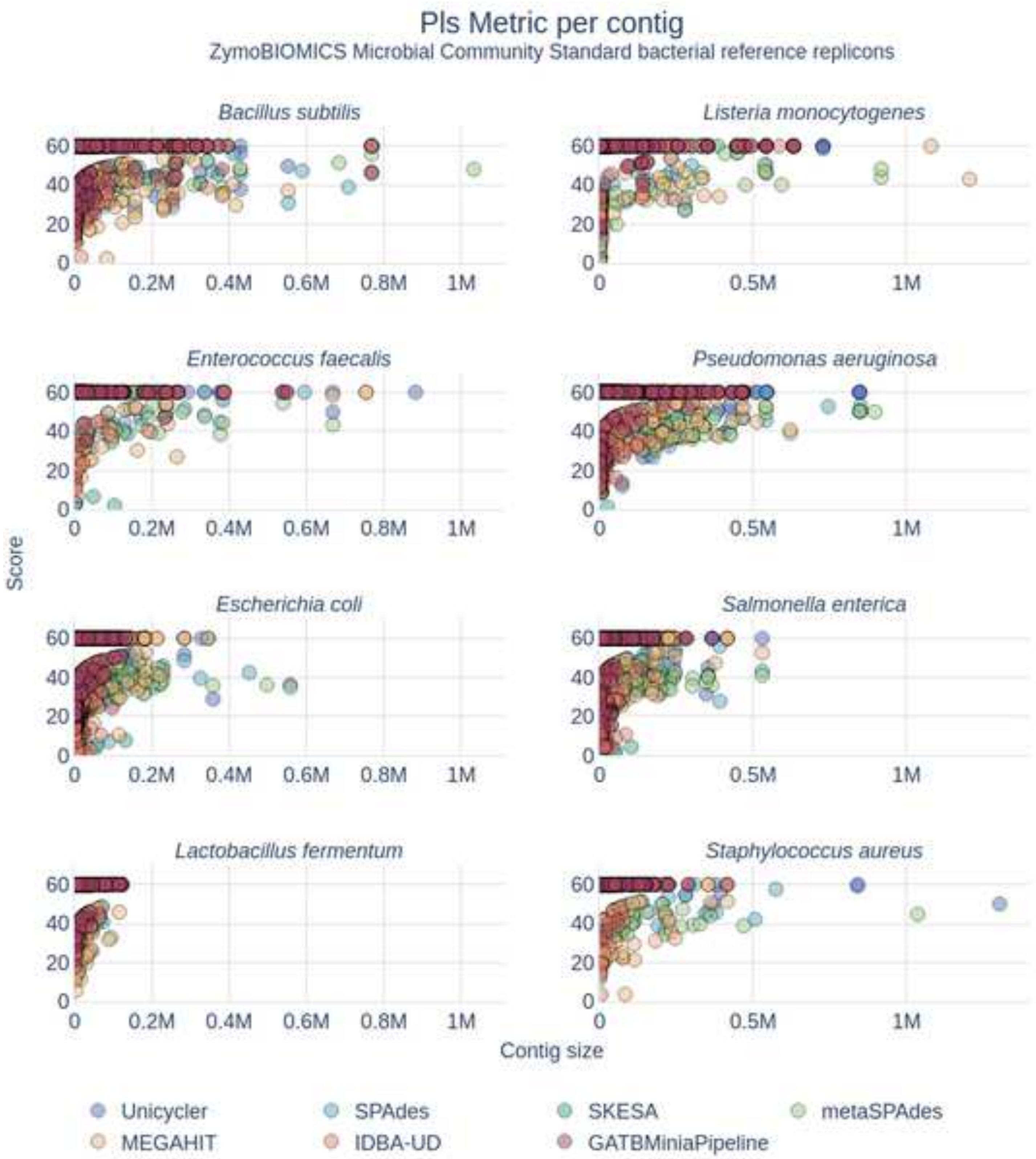

Figure 8

Click here to access/download;Figure;Figure 8.png

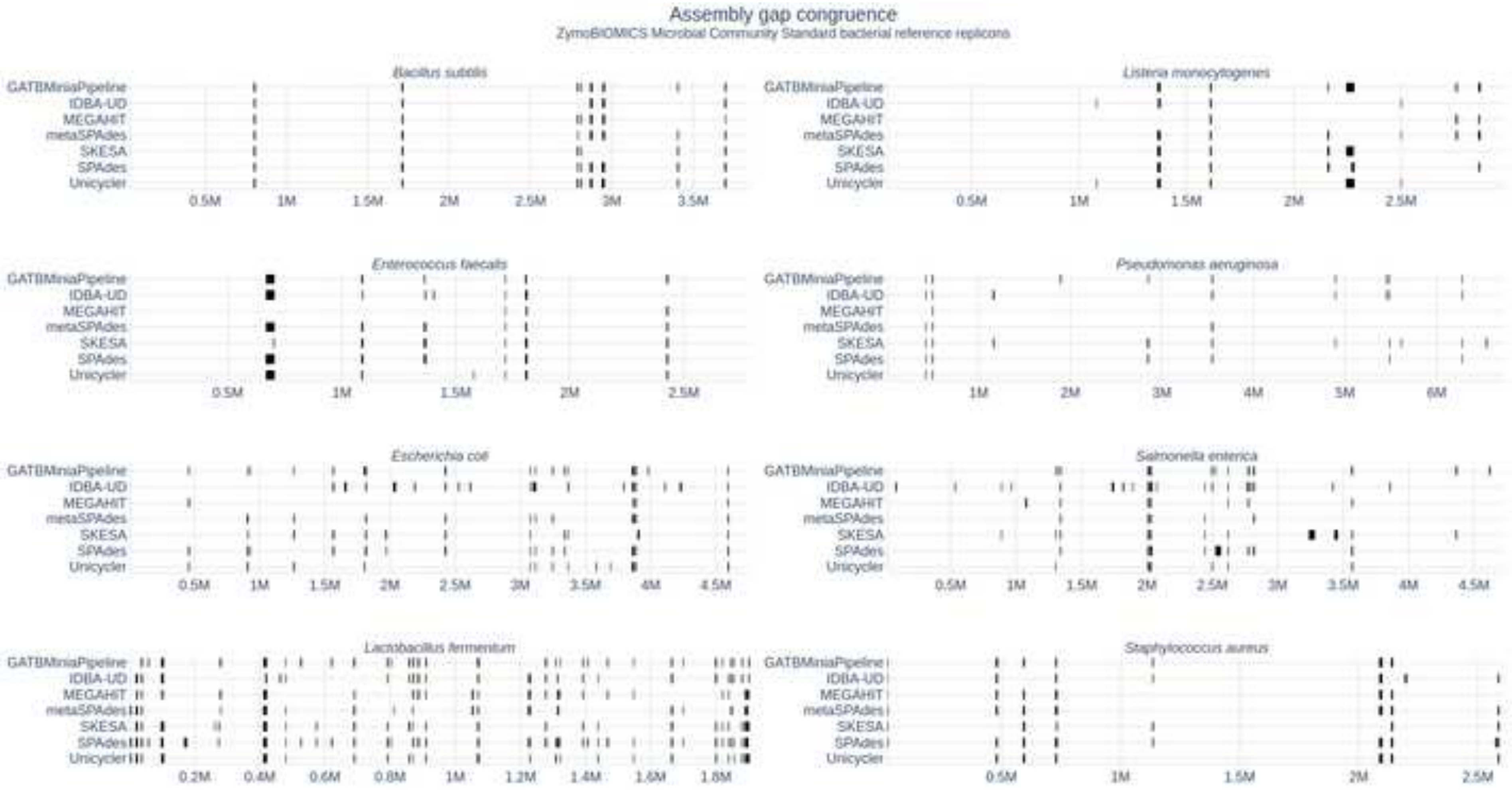

Click here to access/download
**Supplementary Material**
LMAS Supplemental Material - GigaScience - tracked changes.docx

Click here to access/download
**Supplementary Material**
LMAS Supplemental Material - Tables - revision.xlsx

Personal Cover Click here to access/download;Personal Cover;LMAS - Cover letter.docx

Catarina Inês Mendes
Instituto de Medicina Molecular João Lobo Antunes
Lisbon, Portugal
cimendes@medicina.ulisboa.pt

Dear Dr Scott Edmunds, editor-in-chief of *GigaScience*,

I ask that you consider our manuscript entitled "*LMAS: Last Metagenomic Assembler Standing*" for publication in *GigaScience*.

Short-read shotgun metagenomics can offer comprehensive microbial detection and characterisation of complex clinical samples. The *de novo* assembly of raw sequence data is key in metagenomic analysis, yielding longer sequences that offer contextual information and afford a more complete picture of the microbial community. The assembly process is the bedrock and may constitute a major bottleneck in obtaining trustworthy, reproducible results.

In this manuscript, we present LMAS, an automated workflow developed as a flexible platform to allow users to evaluate traditional and metagenomic dedicated prokaryotic *de novo* assembly software performance given known standard communities. Its implementation in Nextflow ensures the transparency and reproducibility of the results obtained and the use of Docker containers provides further flexibility. The results are presented in an interactive HTML report where global and reference specific performance metrics can be explored. Currently, twelve assemblers are still being maintained and were implemented in LMAS, with the possibility of expansion as novel algorithms are developed and new versions released.

LMAS is intended as a tool empowering users to perform their own analysis in conditions meaningful in their context, in contrast to more generic benchmarking efforts such as CAMI. To showcase LMAS we used the test dataset of eight bacterial genomes and four plasmids of the ZymoBIOMICS Microbial Community Standards with linear and logarithmic species distribution, and found that k-mer De Bruijn graph assemblers outperformed the alternative approaches but came with a greater computational cost. Furthermore, assemblers branded as metagenomic specific did not consistently outperform other genomic assemblers in metagenomic samples. Some assemblers still in use, such as ABySS, BCALM2, MetaHipmer2, minia and VelvetOptimiser, showed significant performance problems and their usability may be limited with default parameters, particularly when assembling complex samples.

The performance of each assembler varied depending on the species of interest and its abundance in the sample, with less abundant species presenting a significant challenge for all assemblers. No assembler stood out as an undisputed all-purpose choice for short-read metagenomic prokaryote genome assembly, highlighting that efforts are still needed to further improve metagenomic assembler performance. Our results also suggest that sample complexity and a particular interest in some sample components may affect assembler choice. The great diversity of samples of interest further highlights the usefulness of a one-stop tool assisting users in their decision of which assembler to choose. Using LMAS could help users in their selection of assembler for their specific purpose. As such, we believe that this manuscript is appropriate for publication in *GigaScience* as a Technical note.

This manuscript has not been published and is not under consideration for publication elsewhere. LMAS has been featured in several international conferences such as ABPHM 2021 (doi: 10.5281/zenodo.6025166), ECCMID 2021 (doi: 10.5281/zenodo.6025210) and ICCMg 6 (doi: 10.5281/zenodo.5578327), the latter receiving the best poster award.  All authors have approved the manuscript for submission and have no conflicts of interest to disclose.

Thank you for your consideration.
Sincerely, on behalf of all authors,

Catarina Inês Mendes, MSc
Email: cimendes@medicina.ulisboa.pt
Universidade de Lisboa, Instituto de Medicina Molecular, Instituto de Microbiologia

Reply to reviewers

Click here to access/download;Personal Cover;LMAS - Review reply to GigaScience.docx

September 26, 2022

Catarina Mendes
Instititutio de Microbiologia, Instituto de Medicina Molecular
Faculdade de Medicina, Universidade de Lisboa
Av. Prof. Egas Moniz
PT 1649-028 Lisboa
Portugal
Email: cimendes@medicina.ulisboa.pt

Dr Nicole Nogoy
Executive Editor, GigaScience

Dear Dr. Nogoy,

We are grateful for the editorial and referral critique of our paper entitled **"LMAS: Last Metagenomic Assembler Standing" (GIGA-D-22-00108)** that we submitted for publication in GigaScience. The reviewers' comments led to further analyses on our part which we believe enriched the paper. Moreover, it led us to highlight findings which we believe will be useful to the community, which were not immediately apparent previously, broadening the scope of the original paper. We have also carefully reviewed and modified the manuscript to further clarify in the text the points raised by the reviewers.

The CAMI datasets have remained popular after their publication (Sczyrba et al. 2017), Despite their popularity, no information is provided in the manuscript or in the supplemental material about the source of the genomes in each dataset. It is only mentioned that the community composition was designed according to specified criteria and the metagenome datasets simulated using https://github.com/CAMIchallenge/MetagenomeSimulationPipeline/releases/tag/0.1, which is no longer available. As such, despite the datasets being described as publicly available at https://data.cami-challenge.org/participate, only the "Gold Standard" assemblies are made available, and not the source references for the generation of the mock datasets. As the reviewer suggested, we tried to download the datasets and had difficulties obtaining the dataset following the information provided in the paper. We then contacted the authors and were informed that there were problems with the server where the data was hosted and that the data was unavailable (see attachment). Unfortunately there was no subsequent contact from the CAMI authors indicating the data had again become available.

As an alternative, and keeping in mind the requirement of the inclusion of closely related species in the dataset, we've introduced a new analysis in the manuscript of the **BMock12** community (Sevim et al. 2019), composed of 12 species including several closely related sets: two replicons of *Halomonas* sp. (ANIb=0.98), three replicons of the Micromonospora genus (average ANIb=0.85) and two replicons of *Marinobacter* sp (ANIb=0.78). Furthermore, and to represent mock community trying to reproduce and existing microbiome, the **NIBSC Gut DNA Reference Gut-Mix-RR and Gut-Mix-HiLo** community standard (Amos et al. 2020) was analysed, consisting of 20 common gut microbiome strains in an even and staggered composition respectively. These strains have an average ANIb of 0.67, with a maximum of 0.95 between the two *Bifidobacterium longum* subspecies. It's worth noting that no complete genome is available for eight of the strains in the sample, including one of the *Bifidobacterium*

*longum* subspecies, therefore taking full advantage of the potential information in this sample is challenging. With the addition of these two datasets, we aim to provide both a high similarity case study as well as the usefulness of this tool using a perhaps more realistic mock community.

LMAS requires defined mock communities as a ground truth. To emphasise this requirement, we've changed the title of the manuscript to "***LMAS: Evaluating metagenomic short de novo assembly methods through defined communities***". Below we will respond to all comments and corrections suggested by the reviewers.

We have submitted a new manuscript, with all changes highlighted in the text. The low quality figures have also been submitted separately to the submission platform in PDF format, as well as Zenodo (https://doi.org/10.5281/zenodo.6783042), including the original dynamic HTML files. Additionally, the reports for the ZymoBIOMICS Microbial Community Standard, BMock12 Community Standard and NIBSC Gut DNA Reference are available at https://doi.org/10.5281/zenodo.7088960, https://doi.org/10.5281/zenodo.7092431 and https://doi.org/10.5281/zenodo.7092693, respectively. We hope to have satisfactorily addressed all the issues raised by the reviewers and hope that you find the revised version of the manuscript acceptable for publication.

With our best regards,
On behalf of all authors,

Catarina Mendes

Catarina Inês Marques de Sousa Mendes

seg 20-06, 11:47

amc14@helmholtz-hzi.de; Mário Ramirez; João André Nogueira Custódio Carriço

Responder a todos

Greetings Dr. McHardy

My name is Inês Mendes and I'm a PhD student working on metagenomic data analysis, in particular the assessment of better standards for its applicability in the clinic. I'm currently working on a novel software benchmark approach including novel statistics and and since CAMI's datasets are the best reference for complex datasets and well studies they would be the best to be used in my analysis to contrast with the low complexity sets that I have. However, in CAMI's paper (https://doi.org/10.1038/nmeth.4458) I don't seem to find any place where I can download the "golden standard" reference genomes from where the datasets were originated

Is this data available? I can find the read data and the binning results here (https://edwards.flinders.edu.au/cami-challenge-datasets/ and https://data.cami-challenge.org/participate) but not the references that were used to generate the read data in the first place. Could you please assist me?

Thank you for your attention and your time,
Inês Mendes,
PhD Student
Faculdade de Medicina, Universidade de Lisboa

Fernando Meyer <fernando.meyer@helmholtz-hzi.de>

seg 15-08, 14:11

Dear Inês,
I'm not sure if your question from a while ago was answered, but I hope you already found the gold standards and reference genomes you needed. They are indeed available at https://data.cami-challenge.org/participate together with the complete datasets. We provide additional datasets in CAMI 2, described in our latest paper (https://doi.org/10.1038/s41592-022-01431-4). The paper contains a 'Data availability' section with links to the data.
Sorry if your question was unanswered until now. If you still have any questions, just let me know or write to support@cami-challenge.org, our official CAMI support email address.
Best,
Fernando

Catarina Inês Marques de Sousa Mendes
qua 17-08, 12:49
support@cami-challenge.org; fernando.meyer@helmholtz-hzi.de; +1

Responder a todos

1366_001.pdf
56 KB

Transferir

Dear Fernando,

Thank you for your reply. Indeed my question remained unanswered so I highly appreciate your help. Unfortunately I cannot access the full dataset as my email address is not recognised. I've attached to this email the data confidentiality agreement.

When you mention that the golden standards are available, are you referring to the .fasta.gz files available, for example for the low complexity dataset, at https://openstack.cebitec.uni-bielefeld.de:8080/swift/v1/CAMI_I_TOY_LOW? The file S_S001__genomes_30__insert_180_reads_anonymous.fq.gz contains the interleaved reads, but when trying to download it from https://openstack.cebitec.uni-bielefeld.de:8080/swift/v1/CAMI_I_TOY_LOW/S_S001__genomes_30__insert_180_reads_anonymous.fq.gz an "Access Denied" error is received. The "Golden standard assembly", available at https://openstack.cebitec.uni-bielefeld.de:8080/swift/v1/CAMI_I_TOY_LOW/S_S001__genomes_30__insert_180_gsa_anonymous.fasta.gz, contains 56168 contigs, with an N50 of just 18406, indicating a very fragmented assembly. Or are there additional files that I don't have current access to with the genomes used to originate the mock community?

Thank you very much for the time and clarifications you've provided.

Best wishes,
Inês Mendes

Fernando Meyer <fernando.meyer@helmholtz-hzi.de>
qui 18-08, 10:12
Catarina Inês Marques de Sousa Mendes; support@cami-challenge.org

Responder a todos

Hi Inês,

we are having some issues with our servers, sorry about that. My colleagues are working on a solution.

Regarding the fragmented assembly, CAMI 1 'low' is a relatively small and now somewhat old dataset. I would recommend you try the CAMI 2 datasets instead. Unfortunately, we currently have some download issues for those as well.

I will get back to you as soon as we have a solution.

Best,

Fernando

## Reviewer 1 - Major Comments

The authors describe "LMAS: Last Metagenome Assembler Standing", as a platform for evaluating different metagenome assemblers given known communities. Since the field of metagenome assemblers is a rapidly developing one, having good resources for evaluating and selecting methods available is an important topic. The paper itself is well-written and installation of the software via bioconda works out of the box. There are however some problems with the current form of the software and article which need to be addressed.

### Regarding the software:

1. **While starting the pipeline is easy enough, LMAS still requires a lot of resources since the assemblers themselves are run. Running the pipeline on the server available to me was not possible though (crashed with the error message "local avail memory attribute cannot zero. expression (avail memory 0). values avail memory = 0"). Using a lower max_cpus and max_memory option and running on a laptop (using the ENN dataset with even errors) was possible, but only a few assemblers were successful and the final report was not created.**

We thank the reviewer for the comment. The default CPU and memory values in LMAS are the ones we found were required to successfully assemble the ZymoBIOMICS dataset. Assembly is a resource-intensive process, as is the mapping done in the evaluation of the assemblies by LMAS. Unfortunately, this is a limitation that LMAS cannot circumvent. Although The CPU and memory values can be easily changed, and maximum CPU and memory values defined, we cannot guarantee the success of the assembly process for such data. LMAS is robust to assembler failures, with the possibility of assemblers being skipped from the workflow, as well as an indication in the final report that an assembly process has failed. The cause for a lack of a final report would warrant further investigation, but it could be due to a lack of resources for the mapping process required by LMAS. To address the reviewer's concerns continuous integration of the LMAS workflow was implemented, for both docker and singularity systems, to ensure that the system is working as intended (https://github.com/B-UMMI/LMAS/actions). Test data is available in LMAS' repository (https://github.com/B-UMMI/LMAS/tree/main/test/data) consisting of a small *in silico* sample with just 200,000 read pairs belonging to *Bacillus subtilis* and *Staphylococcus aureus* that, due to its small size, is possible to run locally with relatively modest resources for most assemblers (Unicycler still requires a significant amount of resources with such small data).

### Regarding the article:

2. **The text mentions assemblers using OLC and dBg, but Table 1 shows only assemblers using deBruijn graphs were selected for evaluation. It is probably not necessary to mention OLC at all, since they have not been used for metagenome assemblers and the software selection reflects that.**

We thank the reviewer for the comment. Indeed only dBg graph assemblers were ultimately included in LMAS. As such, and in accordance with the reviewer's suggestion, the mention of OLC assemblers was removed from the background section of the manuscript (lines 60 to 63).

3. **The definition of the PI-score (Equation 1) seems to be malformed: If E = 1 - Identity and assuming Identity is from 0-1 then E is always <60, so the first condition in Equation 1 needs to be changed.**

We thank the reviewer for the comment. Indeed there was a mistake in Equation 1 that has now been corrected to the following:

$$Phred(E) = \begin{cases} -log(E) \times 10 & \text{if } 0 < E \leq 1 \\ 60 & \text{if } E = 0 \end{cases}$$

$$\text{where } E = 1 - \text{Identity}$$

4. **"Assembly robustness" seems like a strange metric: Deterministic assemblers will always have the most robust results, but assemblers which use randomness are not necessarily worse; it is not clear to me why this is labeled as "poor results". In contrast to that, the LSA metric is an interesting addition to the set of metrics described in e.g. QUAST.**

We thank the reviewer for the comment. We agree with the reviewer in regards to the fact that randomness does not translate directly into a worse result, therefore the assembly robustness alone should not be the metric of choice to exclude an assembler. Having said this, inconsistency of results from the same data may confuse more inexperienced users and cancreate difficulties if an assembler is to be included in a pipeline for accreditation. Having said this, we agree with the reviewer that non-deterministic assemblers are, by definition, non-reproducible. While not necessarily worse, they may be problematic for comparison with more deterministic assemblers in terms of the reproducibility of the comparison in different labs or at different times. We believe that the reproducibility of the assemblies generated is a piece of relevant information that should be included when assessing assembler performance. We have therefore changed “assembly robustness” to “assembler reproducibility” in Figure 3 (https://zenodo.org/record/7007317#.Yyn3Huxue3I).

5. **The discovery that single k-mer dBg assemblers are typically outperformed by multi-k ones has been described before (e.g. IDBA-UD) and should be cited.**

We thank the reviewer for the comment. Appropriate references have been added, namely (Sczyrba et al. 2017; Meyer et al. 2022; Xavier et al. 2014; Mahadik et al. 2019) (line 345).

6. **Similarly, the dependence of assemblers to recover genomes with low abundance has been described before (e.g. CAMISIM).**

We thank the reviewer for the comment. The appropriate reference has been added, namely (Sczyrba et al. 2017; Meyer et al. 2022; Fritz et al. 2019) (line 545).

7. **Again in contrast to that, the influence of the individual species (particularly *P. aeruginosa*) on the assembly quality could have been elaborated on further.**

Following the reviewer's suggestion, the following has been added to the manuscript (lines 470-471): *However, in the case of the larger number of contigs of P. aeruginosa, no related species are present in the sample and these possibly reflect intrinsic properties of the replicon such as the high number of prophages integrated in the bacterial genome (Johnson et al, 2019).* Unfortunately, existing mock communities do not exist allowing us to expand on the possible importance of replicon intrinsic properties in the quality of the assemblies produced. The availability of LMAS will allow more easily addressing this question by allowing researchers to focus on the experimental design of such communities.

8. **For Figures 4 and Figure 7 many of the points cluster on top of each other, which makes manual inspection of these results hard to do. Generally the figures available to me were of quite low quality, they should either be vectorized or of higher quality.**

We thank the reviewer for this comment. We apologise for the lack of readability regarding Figures 4 and 7, and have uploaded high-resolution PDF versions of each. Figure 5 was also uploaded as a PDF for the same reason. Additionally, All figures are now available, in their original form, in Zenodo under DOI https://doi.org/10.5281/zenodo.6783042. The availability of data and materials has been updated to reflect this addition, now reading "*Likewise, all figures in the current manuscript are available in their original format in the Zenodo repository, under https://doi.org/10.5281/zenodo.6783042.*" (lines 660-662).

9. **The conclusion that metagenome assemblers do not outperform genome assemblers is based on the - low complexity - data sets. This problem is touched upon in the conclusion but the header in the main text can be misleading. To come to the conclusion that metagenome assemblers do not outperform genome assemblers, LMAS would need to show this on at least one dataset with somewhat realistic complexity, e.g. the ones used for the mentioned CAMI challenge. Doing this could also show the advantage of LMAS against the mainly used QUAST in bundling the execution and evaluation together.**

As stated throughout the manuscript, LMAS requires defined mock communities as a source of ground truth. We appreciate the reviewer's comment regarding the misleading nature of the header of the manuscript. To emphasise this requirement, we've changed the title of the manuscript to "***LMAS: Evaluating metagenomic short de novo assembly methods through defined communities***"

Given the problems in obtaining the CAMI samples we have tried to find relevant mock communities, sequenced using short-read technologies,of greater complexity than the ZymoBIOMICS community. Keeping in mind the requirement of the inclusion of closely related strains in the dataset, we've introduced a new analysis in the manuscript of the **BMock12** community (Sevim et al. 2019). The challenges of this dataset are the two *Halomonas* and the two *Marinobacter* strains, which come with an average ANIb of 0.98 and 0.85 (see Supplementary Table S25), respectively. Additionally, the **NIBSC Gut DNA Reference Gut-Mix-RR and Gut-Mix-HiLo** datasets were analysed, representing a more naturally occurring microbiome that includes 5 phyla, 13 families, 16 genera and 19 species that includes several abundant species and strains present in the human gut (Amos et al. 2020). With this, we wish

to better showcase the usefulness of this tool using more difficult datasets. The reports for the ZymoBIOMICS Microbial Community Standard, BMock12 Community Standard and NIBSC Gut DNA Reference are available at https://doi.org/10.5281/zenodo.7088960, https://doi.org/10.5281/zenodo.7092431 and https://doi.org/10.5281/zenodo.7092693 respectively (lines 666-669 in the manuscript).
Following the analysis of these new datasets, the conclusion that the metagenomics assemblers do not outperform the genomic assemblers still holds true.

## Reviewer 2 - Major Comments

This is a technical note that describes LMAS, a tool that launches multiple genome assemblers on the same metagenomic dataset and then generates a report with various assembly quality metrics. The metrics, including two newly introduced ones, make sense. The choice of assemblers also generally makes sense, although I do have some remarks below regarding a couple choices. The analysis is thorough and provides some interesting takeaway messages. The manuscript is well-written and the presentation is didactic.

My main doubt about this article is whether the LMAS tool would be of broad interest. Between existing large-scale benchmarking efforts like CAMI and popular evaluation tools that generate assembly reports like MetaQUAST, it is difficult to assess the precise need for LMAS. The suggested use case by the authors is that a user should run him/herself the assemblers on a particular dataset of interest. The advantage would therefore be to have an integrated solution to run all tools and evaluate the results automatically. While this is handy, a user might also simply run a 2-3 of the best assemblers as suggested by the article (or CAMI), considering that many of the LMAS produce suboptimal assemblies and they are not reconciled.

A redeeming aspect of the article is the newly made benchmark on low-complexity Zymo real & simulated data (8 bacterial species). This benchmark contains some interesting findings, such as the fact that genomic assemblers appear to perform as well a metagenomes ones. This claim should however be put into context: the metagenomic data is low-complexity (only 8 species), and the presence of similar strains in actual metagenomes may significantly change that message.

1. **The authors may want to make a stronger case why a user would want to run LMAS instead of picking 2 (or 3) of the best-performing assemblers and run MetaQUAST on them. In an actual assembly scenario, i.e. without ground truth, are there situations where running more than 2 assemblers is beneficial? I am genuinely interested in the answer.**

We thank the reviewer for this comment. The purpose of LMAS is, given a ground truth of a defined mock community, to enable the benchmarking of traditional and metagenomic prokaryotic *de novo* assembly software. Although we understand the reviewer's point that CAMI offers this guidance, CAMI challenges are spaced in time and assemblers are released at a much faster pace, so a user may be interested in evaluating a novel assembler without the "self-assessment" trap associated with the publication of novel tools before the next CAMI challenge. Moreover, our analyses show that most of the conclusions on assembler performance are not generalisable, therefore there is no absolute guarantee that the best performing assemblers for a given scenario will be the same as for a different one (namely

considering the replicon of interest, its abundance in the sample and other replicons present in the sample). Therefore, we encourage users to run the assemblers of interest with mock communities as close as possible to their sample of interest. Admittedly, a user may want to concentrate on 2-3 assemblers, but the task of running these assemblers followed by MetaQUAST may still be daunting. LMAS, with its modular and streamlined design, lowers the barrier of performing such analyses. Moreover, in addition to the novel quality metrics, LMAS offers representations which are not offered by MetaQUAST, such as the positions of assembly gaps and of SNPs in the genomes of interest which can be explored in the future to further understand the strengths and weaknesses of particular assemblers.

To better reinforce some of these points, the following sections have been added to the text: "The portability and ease of use of LMAS are intended to provide users with a continuous benchmarking platform to easily evaluate the performance of assemblers, as they are developed, in mock communities mimicking as closely as possible their samples of interest." and "*Even when considering communities with very similar replicons, the overall performance of metagenomic assemblers was not consistently better than that of genomic assemblers. The results also indicate that the recovery of assemblies allowing strain-level discrimination at the SNP level is highly unlikely based solely on the assembler generated contigs.*" (lines 580-583).

2. **Were there multiple high-similarity strains in the Zymo data? If not, this should be noted in the presentation of the dataset. This is an important caveat that limits the significance of the reported results. In particular I would suggest to tone down the claim that genomic assemblers perform as well as metagenomic assemblers, given e.g. the many heuristics that MetaSpades implements to resolve close strains.**

We thank the reviewer for this comment. The similarity of the strains in the ZymoBiomics is available as supplemental table S23, where pairwise comparisons of the microbial community standard reference replicons are available. As observed in this table, *Escherichia coli* and *Salmonella enterica* have the highest level of similarity, with an ANIb of 0.80.

Keeping in mind the need of analysing closely related species in the same mock community, we've introduced a new analysis in the manuscript of the **BMock12 community** (Sevim et al. 2019), composed of 12 strains including several closely related sets: two replicons of *Halomonas* sp. (ANIb=0.98), three replicons of the Micromonospora genus (average ANIb=0.85) and two replicons of *Marinobacter* sp (ANIb=0.78). We hope that, with the introduction of the analysis of this dataset, we have successfully addressed the scenario raised by the reviewer where multiple similar strains are present in the sample. Essentially, our initial assertion stands, with genomic assemblers not being systematically outperformed by metagenomic assemblers.

Keeping in mind the reviewer suggestions, the following has been added to the conclusion of the manuscript (lines 567-573): "*Although the eight species ZymoBIOMICS Microbial Community Standards might not be representative of the metagenomic complexity of the samples of interest of most researchers, we hoped that its relative simplicity meant that the results shown would represent a best-case scenario, since as sample complexity increases so do the challenges to assembler performance. However, the results of the BMock12 and*

*Gut-Mix community standards suggest that the actual genome of interest and community composition play an important part in the results of individual assemblers.*".

3. **The abstract sentence: "Some assemblers still in use, such as ABySS, BCALM2, MetaHipmer2, minia and VelvetOptimiser" should be removed or revised as it is misleading for several reasons: BCALM2 is not an assembler, it constructs unitigs only. Minia is a part of GATBMiniaPipeline which offers better performance. And VelvetOptimiser is an odd choice as MetaVelvet also exists and was untested (although it might not scale). Therefore, I recommend VelvetOptimiser, BCALM2, and possibly minia to be removed from the benchmark, or be marked as odd choices for metagenome assembly.**

We thank the reviewer for this comment. MetaVelvet has been last updated in 2015 (Supplemental Table S1), therefore it was not considered for the benchmark by not meeting our inclusion criteria. Due to the very fair point made regarding BCALM2, it has been removed from LMAS (https://github.com/B-UMMI/LMAS/pull/25), and the manuscript, figures and supplemental material adjusted accordingly. Regarding minia, a similar argument can be made for SPAdes and Unicycler assemblers. Therefore it has not been removed from LMAS and is still considered in the initial benchmark with the ZymoBIOMICS Microbial Community Standard, together with VelvetOptimiser. Given their very poor performance, their execution is now by default set to false in LMAS, alongside Abyss and MetaHipermer2, since given the results presented here these would not be recommended assemblers.

4. **Of note, albeit VelvetOptimiser tries multiple k values, it is still considered to be a single-k assembler as it does not combine the results of multiple iterations. (It merely picks the best k and runs single-k.)**

We thank the reviewer for this comment. Table 1 and supplemental Table S1 have been adjusted accordingly.

5. **Some of the results are obvious, e.g. single-k assemblers perform more poorly than multiple-k assemblers; this is not quite a novel finding.**

We thank the reviewer for this comment. Indeed the notion that multiple k-mer assemblers outperform single k-mer assemblers is not novel, but it is our opinion that there is still limited data supporting this assertion. Therefore, we found it worth mentioning and reinforcing this notion in our manuscript. As such, we have not changed the text. Instead, and in accordance with the comments of reviewer 1, the following references have been added to support this claim: (Sczyrba et al. 2017; Meyer et al. 2022; Xavier et al. 2014; Mahadik et al. 2019) (line 345 in the manuscript).

6. **Figures 4 and 5, even when downloaded as PNG, were low resolution and hardly readable.**

We thank the reviewer for this comment. We apologise for the lack of readability regarding Figures 4 and 5, and have uploaded a high-resolution PDF version of each. Additionally, All

figures are now available, in their original form, in Zenodo under DOI https://doi.org/10.5281/zenodo.6783042 (lines 660-662 in the manuscript).

## Reviewer 2 - Minor Comments

1. **The term "replicon" is used ubiquitously in the paper, and while its definition is biologically sound, it could be worthwhile to recall what the authors precisely mean by it.**

We thank the reviewer for the comment. In this manuscript, the term "replicon" is used to refer to DNA sequences replicating as a single unit, containing a single origin of replication, and therefore excludes the genomes of eukaryotes which can initiate replication from several loci. We've altered the following sentence in the background section of the manuscript (lines 49 to 51) to better inform the reader: *The de novo assembly process is key when analysing metagenomic data since it allows recovering contigs representing the replicons present in the sample, be it prokaryotic chromosomes, plasmids or viruses, from a pool of mixed raw reads.*

2. **While running LMAS on my cluster head node. Some issues occur. The pulling of singularity images did not work. The error message was:**

**Error executing process > 'preprocessing_wf:PROCESS_REFERENCE (1)'**

**Caused by:**
**Failed to pull singularity image**
**command: singularity pull --name cimendes-lmas-python-base-0.1.img.pulling.1654889379926 docker://cimendes/lmas-python-base:0.1 > /dev/null**
**status : 255**
**message:**
**INFO: Converting OCI blobs to SIF format**
**FATAL: While making image from oci registry: error fetching image to cache: while building SIF from layers: unable to create new build: while ensuring correct compression algorithm: while creating squashfs: create command failed: exit status 1: FATAL ERROR:Failed to create thread**

**However, running the command myself:**

**$ singularity pull --name cimendes-lmas-python-base-0.1.img.pulling.1654889379926 docker://cimendes/lmas-python-base:0.1**

**Worked.**
**Then, after that, since I need to run LMAS on the head node for the first time (other nodes are not connected to internet so cannot pull containers), I get the following error message:**

**Error executing process > 'assembly_wf:SKESA (ERR2935805)'**

**Caused by:**
**Process requirement exceed available CPUs -- req: 8; avail: 4**

**In summary, it would be helpful to have instructions to pull all containers from the head node ahead of time to prepare for LMAS execution on a node not connected to Internet.**

We thank the reviewer for this observation. Continuous integration with singularity has been added to LMAS under https://github.com/B-UMMI/LMAS/pull/23. Indeed we observe that the pull of the images with singularity is sometimes inconsistent but restarting the workflow solves the issue most times. Additionally, in the LMAS documentation the requirement of an internet connection to download the required images was added as Nextflow automatically handles the pull of the required images. Alternatively, a script has been included to handle the pulling of the required images using shifter, docker or singularity (https://github.com/B-UMMI/LMAS/pull/31) before running LMAS for the first time to allow users to run LMAS without the problems described by the reviewer.

## References


Amos, Gregory C. A., Alastair Logan, Saba Anwar, Martin Fritzsche, Ryan Mate, Thomas Bleazard, and Sjoerd Rijpkema. 2020. ‘Developing Standards for the Microbiome Field’. *Microbiome* 8 (1): 98. https://doi.org/10.1186/s40168-020-00856-3.

Fritz, Adrian, Peter Hofmann, Stephan Majda, Eik Dahms, Johannes Dröge, Jessika Fiedler, Till R. Lesker, et al. 2019. ‘CAMISIM: Simulating Metagenomes and Microbial Communities’. *Microbiome* 7 (1): 17. https://doi.org/10.1186/s40168-019-0633-6.

Mahadik, Kanak, Christopher Wright, Milind Kulkarni, Saurabh Bagchi, and Somali Chaterji. 2019. ‘Scalable Genome Assembly through Parallel de Bruijn Graph Construction for Multiple K-Mers’. *Scientific Reports* 9 (1): 14882. https://doi.org/10.1038/s41598-019-51284-9.

Meyer, Fernando, Adrian Fritz, Zhi-Luo Deng, David Koslicki, Till Robin Lesker, Alexey Gurevich, Gary Robertson, et al. 2022. ‘Critical Assessment of Metagenome Interpretation: The Second Round of Challenges’. *Nature Methods* 19 (4): 429–40. https://doi.org/10.1038/s41592-022-01431-4.

Sczyrba, Alexander, Peter Hofmann, Peter Belmann, David Koslicki, Stefan Janssen, Johannes Dröge, Ivan Gregor, et al. 2017. ‘Critical Assessment of Metagenome Interpretation—a Benchmark of Metagenomics Software’. *Nature Methods* 14 (11): 1063–71. https://doi.org/10.1038/nmeth.4458.

Sevim, Volkan, Juna Lee, Robert Egan, Alicia Clum, Hope Hundley, Janey Lee, R. Craig Everroad, et al. 2019. ‘Shotgun Metagenome Data of a Defined Mock Community Using Oxford Nanopore, PacBio and Illumina Technologies’. *Scientific Data* 6 (1): 285. https://doi.org/10.1038/s41597-019-0287-z.

Xavier, Basil Britto, Julia Sabirova, Moons Pieter, Jean-Pierre Hernalsteens, Henri de Greve, Herman Goossens, and Surbhi Malhotra-Kumar. 2014. ‘Employing Whole Genome Mapping for Optimal de Novo Assembly of Bacterial Genomes’. *BMC Research Notes* 7 (1): 484. https://doi.org/10.1186/1756-0500-7-484.
