# Supplementary material for: LMAS: evaluating metagenomic short de novo assembly methods through defined communities
Source: Gigascience. 2022 Dec 28;12:giac122. doi: 10.1093/gigascience/giac122 (PMC9795473; doi:10.1093/gigascience/giac122)
Supplement: giac122_Supplemental_Files [file giac122_supplemental_files.zip › LMAS Supplemental Material - GigaScience - tracked changes.docx]

Supplemental Materials

# Workflow parameters

In LMAS, a set of default parameters is provided but these can be altered, either by passing the new value when executing the workflow or by editing the “params.config” file in the “configs” folder. There are three main parameters in LMAS: “reference”, “fastq” and “md”. The short-read data is passed as input through the “--fastq” parameter, which by default is set to match all files in the “data/fastq” folder that match the pattern “*_R{1,2}*”. The reference sequences in a single file can be passed with the “--reference” parameter, matching by default fasta files (with the pattern “*.fasta”) in the “data/reference” folder. Although not mandatory, text information, in a markdown file, on input samples can be passed to LMAS to be presented in the report with the “--md” parameter. By default, this is matched to the “**.md*” pattern in the “*data*” folder.

Several options are available to alter the behaviour of the assemblers incorporated in LMAS, namely to alter the values of the k-mer for each assembly iteration, as detailed in the documentation [1]. By default, these values reflect the corresponding default settings of the assemblers. Additionally, each assembler can be skipped from the workflow, and the resources for the execution, such as CPUs, memory and time limit, can be altered for all assembly processes. For the assembly quality assessment performed by LMAS, the following parameters are provided and can be adjusted:

- “*--minLength*”: Value for minimum contig length, in basepairs. By default, this value is set to 1000 basepairs;
- *“--mapped_reads_threshold”:* Value for the minimum percentage of a read aligning to the contig to be considered as mapped. By default, this value is set to 75%;
- *“--n_target”*: Target value for the *N*, *NA* and *NG* metrics, ranging from 0 to 100%. By default, this value is set to 50%;
- *“--l_target”*: Target value for the *L* metric, ranging from 0 to 100%. By default, this value is set to 90%;

# Short-read *de novo* assemblers

We’ve compiled a collection of de novo assembly tools, including Overlap, Layout and Consensus (OLC) and De Bruijn graph (dBg) assembly algorithms, with both single k-mer and multiple k-mer value approaches, and hybrid assemblers (Supplemental Table S1). The collection includes both genomic and metagenomic assemblers, developed explicitly to handle metagenomic datasets. The dates of the last release correspond to the ones available in the preparation of this manuscript.

## Selection Criteria

Only open-source tools, with clear documentation describing the methodology implemented, were considered. The collection of tools was ordered by the date of the last update, and a Docker container [2] for the top 11 assemblers was created with the latest released version, with the version used as the tag. In the case of tools where a versioned release is not available, the container was created with the latest version in the default branch of the source repository, using the date of the last update as the tag. The PANDAseq [3] assembler was excluded due to execution errors.

## Assemblers in LMAS

Assemblers benchmarked in LMAS, in alphabetical order:

### ABySS

The ABySS assembler [4] is a de novo sequence assembler intended for short paired-end reads and genomes of all sizes. It follows the model of minia, wherein a probabilistic Bloom filter representation is used to encode the de single k-mer size Bruijn graph, reducing memory requirements for de novo assembly. The code is open-source and available at [5]. The following command is used: “abyss-pe name='$sample_id'k=$KmerSize B=$BloomSize in='$fastq”, where “$sample_id” contains the identifier of the sample, contains a list of the input read files, “$sample_id” the identifier of the sample, “$KmerSize” the length of the nodes of the graph (by default set to 96), “$BloomSize” the size, in Gb, of the bloom filter (by default set to 2 GB), and “$fastq” the forward and reverse fastq files.

### GATB-Minia Pipeline

GATB-Minia is an assembly pipeline, still unpublished, that consists of Bloocoo [6] for error correction, minia 3 [7] for contigs assembly, which is based on the BCALM2 assembler [8], and BESST [9] for scaffolding. It was developed to extend the minia assembler to use the dBg algorithm with multiple k-mer values and to explicitly handle metagenomic data. The code is open-source and available at [10]. The following command is used: “gatb -1 $fastq_pair[0] -2 $fastq_pair[1] --kmer-sizes $kmer_list -o $sample_id”, where $fastq_pair[0] contains the forward-facing reads, $fastq_pair[1] the reverse-facing reads, $kmer_list the list of values for length of the nodes of the dBg (by default set to 21,61,101,141,181), and “$sample_id” the identifier of the sample.

### IDBA-UD

IDBA-UD [11] is a dBg graph assembler for assembling reads from single-cell sequencing or metagenomic sequencing technologies with uneven sequencing depths. It employs multiple depth relative thresholds to remove erroneous k-mers in both low-depth and high-depth regions. The technique of local assembly with paired-end information is used to solve the branch problem of low-depth short repeat regions. To speed up the process, an error correction step is conducted to correct reads of high-depth regions that can be aligned to high confidence contigs. The code is open-source and available at [12]. The following command is used: “idba_ud -l $fasta_reads_single”, where $fasta_reads_single contains the combined sequence data converted to FASTA format reads with “reformat.sh” from BBtools [13].

### MEGAHIT

MEGAHIT [14] is a de novo assembler for large and complex metagenomics datasets. It makes use of the succinct dBg, with a multiple k-mer size strategy. In each iteration, MEGAHIT cleans potentially erroneous edges by removing tips, merging bubbles and removing low local coverage edges, especially useful for metagenomics which suffers from non-uniform sequencing depths. The code is open-source and available at [15]. The following command is used: “megahit -o megahit --k-list $kmers -1 $fastq_pair[0] -2 $fastq_pair[1]”, where $kmers contains the list of values for length of the nodes of the dBg (by default set to 21,29,39,59,79,99,119,141), $fastq_pair[0] contains the forward-facing reads, and $fastq_pair[1] the reverse-facing reads.

### MetaHipMer2

MetaHipMer2 [16] is a multiple k-mer size dBg de novo metagenome short-read assembler built to run efficiently on both single servers and on multi-node supercomputers, where it can scale up to coassemble terabase-sized metagenomes. The code is open-source and available at [17]. The following command is used: “mhm2.py -k $kmers -r $fasta_reads_single -s 0”, where $kmers contains the list of values for length of the nodes of the dBg (by default set to “21,33,55,77,99”), where $fasta_reads_single contains the combined sequence data converted to FASTA format reads with “reformat.sh” from BBtools [13]. The “-s 0” option skips the scaffolding step.

### metaSPAdes

SPAdes [18] started as a tool aiming to resolve uneven coverage in single-cell genome data, with metaSPAdes [19] later released building a specific metagenomic pipeline on top of SPAdes. It uses multiple k-mer sizes of dBg, starting with the lowest kmer size and adding hypothetical k-mers to connect the assembly graph. The code is open-source and available at [20]. The following command is used: “metaspades.py --only-assembler -k $kmers -1 $fastq_pair[0] -2 $fastq_pair[1]”, where $kmers contains the list of values for length of the nodes of the dBg (by default set to “auto”), $fastq_pair[0] contains the forward-facing reads, and $fastq_pair[1] the reverse-facing reads.

### minia

Minia [7] performs the assembly on a data structure based on unitigs produced by the BCALM [8] software and using graph simplifications that are heavily inspired by the SPAdes assembler [18]. Minia is a short-read traditional assembler based on dBg graph using a single k-mer length. The code is open-source and available at [21]. The following command is used: “minia -in $list_reads -out $sample_id”, where “$list_reads” contains a list of the input read files and “$sample_id” the identifier of the sample.

### SKESA

SKESA [22] is a de novo sequence read assembler that is based on dBg and uses conservative heuristics. It is designed to create breaks at repeat regions in the genome, creating shorter assemblies but with greater sequence quality. It tries to obtain good contiguity by using multiple k-mers longer than mate length and up to insert size. The code is open-source and available at <https://github.com/ncbi/SKESA>. The following command is used: “skesa --use_paired_ends --contigs_out $sample_id --fastq $fastq_pair[0] $fastq_pair[1]”, where “$sample_id” refers to the identifier of the sample, $fastq_pair[0] contains the forward-facing reads, and $fastq_pair[1] the reverse-facing reads.

### SPAdes

SPAdes [18] is an assembly tool aiming to resolve uneven coverage in single-cell genome data through multiple k-mer sizes of dBgs. It starts with the smallest k-mer size and adds hypothetical k-mers to connect the graph. The code is open-source and available at [20]. The following command is used: “spades.py --only-assembler -k $kmers -1 $fastq_pair[0] -2 $fastq_pair[1] ”, where $kmers contains the list of values for length of the nodes of the dBg (by default set to “auto”), $fastq_pair[0] contains the forward-facing reads, and $fastq_pair[1] the reverse-facing reads.

### UNICYCLER

Unicycler [23] is an assembly pipeline for bacterial genomes that can do long-read assembly, hybrid assembly and short-read assembly. When assembling Illumina-only read sets, it functions as a SPAdes-optimiser, using a dBg algorithm with multiple k-mer values. The code is open-source and available at [24]. The following command is used: “unicycler -o . --no_correct --no_pilon -1 $fastq_pair[0] -2 $fastq_pair[1]”, where $fastq_pair[0] contains the forward-facing reads, and $fastq_pair[1] the reverse-facing reads.

### VELVETOPTIMIZER

This optimizing pipeline of the Velvet assembler [25] is still unpublished but extends the original tool by performing several dBg assemblies with variable k-mer sizes. It searches a supplied hash value range for the optimum, estimates the expected coverage and then searches for the optimum coverage cutoff. It uses Velvet’s internal mechanism for estimating insert lengths for paired-end libraries. The code is open-source and available at [26]. The following command is used: “VelvetOptimiser.pl -v -s $velvetoptimizer_hashs -e $velvetoptimizer_hashe -f '-shortPaired -fastq.gz -separate $fastq_pair[0] $fastq_pair[1]'”, where $velvetoptimizer_hashs is the lower end of the hash value range that the optimiser will search for the optimum (default: 19), $velvetoptimizer_hashe is the upper end of the hash value range that the optimiser will search for the optimum (default: 31), $fastq_pair[0] contains the forward-facing reads, and $fastq_pair[1] the reverse-facing reads.

# Misassembly detection

For the detection of misassembly events in the assemblies, the assembled sequences are first filtered for a minimum sequence length with BBTools [13] (version 38.44), as defined in the parameters, using the following command: “reformat.sh in=$assembly out=filtered_$assembly minlength=$minLen”, where $assembly contains the file with the assembled sequences and $minLen the value of the minimum sequence length allowed.

The filtered assembled sequences are mapped against the tripled reference replicons, ensuring that the assembled contigs can fully align regardless of their starting position relative to that of the provided reference sequence. This is done with minimap2 [27] (version 2.22) with the following parameters: “minimap2 --cs -N 0 -t -r 10000 -g 10000 -x asm20 --eqx”.

# Assembly filtering and mapping

The assembled sequences are first filtered for a minimum sequence length with BBTools [13] (version 38.44), as defined in the parameters, using the following command: “reformat.sh in=$assembly out=filtered_$assembly minlength=$minLen”, where $assembly contains the file with the assembled sequences and $minLen the value of the minimum sequence length allowed.

The filtered assembled sequences are mapped against the tripled reference replicons, as explained above, with minimap2 [27] (version 2.22) with the following parameters: “minimap2 --cs -N 0 -t -r 10000 -g 10000 -x asm20 --eqx”.

# LMAS Metrics

The following metrics are computed by the LMAS workflow, globally for characteristics intrinsic to the assembled contigs, and relative to the replicons present in the sample.

## Global Metrics

### General contig information

The following metrics are computed and presented in tabular form:

- *Contigs*: The total number of contigs in the assembly;
- *Basepairs*: The total number of bases in the assembly;
- *Maximum sequence length:* The length of the largest contig in the assembly;
- *Number of ‘N’s*: Number of uncalled bases;
- *Mapped reads*: Percentage of mapped reads to the assembly;

For each plot, the following metrics are presented:

- *Contig size distribution per assembler:* For each assembler in LMAS, a boxplot is computed representing the size distribution of contigs that align to any of the reference replicons. The unmapped contigs, if present, are represented in a red scatterplot overlapping the boxplot.
- *Gap size distribution per assembler:* For each assembler in LMAS, a boxplot is computed representing the distribution of gap sizes. Gaps are calculated after aligning all contigs to the reference replicons. All gaps ≥1 basepair in length are considered.

### Contiguity

The following metrics are computed and presented in tabular form:

- *Nx (where 0 < x ⩽ 100)*: Length for which the collection of all contigs of that length or longer in an assembly covers at least a given percentage of the total length of the assembly

### Misassemblies

A misassembly event is defined as a continuously assembled contig being broken into multiple non-collinear blocks when mapping to the reference replicons, i.e. the contig produced by the assembler does not preserve the exact synteny observed in the reference replicon. This may reflect the addition or deletion of sequence stretches or the shuffling of sequence blocks relative to the reference replicons. For a large insertion or deletion to be considered it must be ≥50 basepairs in length [28]. This metric is computed for the filtered set of contigs, i.e. those of length above a user-specified minimum size and mapping to the reference replicons (see [Supplemental Material, Misassembly detection](https://docs.google.com/document/u/0/d/1dlPwQClqI69_HHGCmDUZ-RSc3yuq5RMIYmCbBiwxSwA/edit)). The misassemblies are processed with custom python code.

The following misassembly types are identified:

- *Chimera*: a contig has two or more sequence blocks mapping to different reference replicons;
- *Insertion:* a sequence block (≥50 basepairs) which is not present in any of the reference replicons has been introduced into the contig by the assembly process;
- *Deletion*: a sequence block (≥50 basepairs) of the reference replicon is missing from the contig created by the assembly process;
- *Inversion*: a contig has at least two sequence blocks mapping to the same replicon but reversed end to end, i.e. one of the blocks maps to the sense strand and the other to the antisense strand in the reference replicon while both are in the same strand in the contig, or vice-versa;
- *Rearrangement*: a contig has at least two sequence blocks mapping to the same replicon, in the same orientation, in a different order than in the reference sequence;
- *Translocation*: a contig has at least two sequence blocks abutting in the contig but mapping non-collinearly (over 1000 base pairs apart) in the reference replicon;
- *Duplication*: a sequence block of a contig maps at least twice to the reference replicon in different alignment blocks;
- *Inconsistency*: a contig has at least two sequence blocks abutting in the contig but fails to be classified in any of the previous categories.

[**Figure S1:**](#_5z23e8fa9s8d) **LMAS misassembly classification.** Misassembled contigs are classified into 6 main categories: chimera, insertion, deletion, inversion, rearrangement, translocation and duplication, according to the mapping orientation, the distance between blocks in the contig and the mapping coordinates in the reference replicon. If a contig is classified as being chimeric, no further classification is performed. The other categories are classified independently of each other, with combinations being possible, to better reflect the differences in comparison to the reference. If a contig is broken into multiple sequence blocks but fails to be classified in any of the previous categories, it is reported as being inconsistent.

[Figure S1](#_5z23e8fa9s8d) provides a visual description of the detected misassemblies. The following metric is computed and presented in tabular form:

- *Misassemblied contigs:* Number of contigs with misassembly events.
- *Misassembly events:* Total number of misassemblies in the contigs

In the plot, the metrics are presented for the filtered set of contigs:

- *Misassembled contigs:* Scatter plot for misassembled contigs per assembler, the size of the misassembled contigs, and the number of blocks created by the misassembly in the contig. The distribution of contig size for all misassembled contigs is represented in a boxplot. Information on the misassembly is presented as a hover text for each misassembly event.

## Per Reference Metrics

### General contig information

The following metrics are computed and presented in tabular form:

- *Contigs*: The total number of contigs in the assembly that align to the reference replicon;
- *Basepairs*: The total number of bases in the assembly that align to the reference replicon;
- *Number of ‘N’s*: Number of uncalled bases (N's) in the contigs that align to the reference replicon.

### COMPASS

A measure of the quality of a replicon assembly can be considered the proportion of the reference covered by the contigs, i.e. the breadth of coverage of the reference replicon. The COMPASS metrics [29] complement our view of the quality of the assembly with other metrics such as how much redundancy is there in the assembly or the parsimony of the contigs relative to the reference. COMPASS is composed of the following metrics, presented in tabular form:

- *Breadth of Coverage:* Ratio of covered sequence on the reference by aligned contigs;
- *Multiplicity:* Ratio of the length of the alignable assembled sequence to covered sequence on the reference;
- *Validity:* Ratio of the length of the alignable assembled sequence to total basepairs in the aligned contigs;
- *Parsimony:* Cost of the assembly (multiplicity over validity);

Additionally, the Breadth of Coverage metric is displayed graphically:

- *Genome Fragmentation:* Scatter plot representing the number of contigs per breadth of coverage of the reference, per assembler.

### Contiguity

To supplement the traditional NA and NG contiguity metrics implemented in QUAST [30], we define the LSA metric as the longest single alignment between the assembly and the reference replicon, relative to the reference replicon length, as proposed previously [31]. This provides a simpler picture of assembly quality as lower contiguity immediately suggests a higher fragmentation, missing sequences or more misassemblies. The following metrics are presented in tabular form:

- *LSA:* longest single alignment between the assembly and the reference, relative to the reference length;
- *NAx (where 0 < x ⩽ 100):* Length for which the collection of aligned contigs of that length or longer in an assembly covers at least a given percentage of the total length of the reference replicon;
- *NGx* *(where 0 < x ⩽ 100):* Length for which the collection of aligned contigs of that length or longer covers at least a given percentage of the sequence of the reference.
- *Lx* *(where 0 < x ⩽ 100):* Minimal number of contigs that cover x % of the sequence of the reference;

The *NAx*, *NGx* and *Lx* metrics are presented graphically in a line plot for each value of x, where *x* represents the percentage of the sequence of the reference, ranging from 0 to 100, per assembler*.*

### Identity

The identity is defined as the number of exact matches between the contigs and the reference replicon, relative to the reference replicon length. The following metrics are presented in tabular form:

- *Identity:* Ratio of identical basepairs in all aligned contigs to the reference;
- *Lowest identity:* Identity of the lowest scoring contig to the reference.

For each plot, the metrics are presented for the contigs filtered for a minimum length that align with the reference replicon.

- *Pls Metric:* Scatter plot for the Phred-like score per contig, per assembler.
- *Gaps*: Location of gaps in comparison to the reference sequence, per assembler, with the cumulative number of gaps per position in the reference. Gaps with 1 basepair or more in length are considered.
- *SNPs*: Location of substitutions in comparison to the reference sequence, per assembler, with the indication of the substitution type and coordinate in the reference. Additionally, the cumulative number of SNPs per position in the reference is presented.

### Misassembly

Similar to what is performed in Global Metrics, this metric is computed for the filtered set of contigs. An aligned contig is considered misassembled when broken into multiple blocks when mapping to the linear reference replicon. Chimeric contigs aligning to more than one reference replicon are counted in each reference individually.

- *Misassembled contigs*: Number of aligned contigs that contain a misassembly event;
- *Misassembly events:* Total number of misassemblies in the aligned contigs;

Additionally, the following information is shown graphically:

- *Misassemblies*: Location of the alignment blocks of misassembled contigs in comparison to the reference sequence, per assembler, with the cumulative number of basepairs in the alignment blocks per position in the reference.

## Computational Performance Metrics

Different software, implementing distinct de novo assembly algorithms, have distinct computational requirements. As such, computational statistics are registered for each assembler. The following metrics are presented in tabular form:

- Avg Time: Average run-time formatted as “hour:minute:second”;
- CPU/Hour: Average amount of time, in hours, of CPU usage by an assembler. CPU load obtained from the number of CPUs and their usage percentage;
- Max Memory (GB): Maximum peak memory usage by the assembler;
- Average Read (GB): Average data size read from disk by the assembler;
- Average Write (GB): Average data size written to disk by the assembler.

Additionally, for reproducibility and traceability purposes, the following information is also registered for each assembler in the table:

- Version: Version of the assembler captured from stdout;
- Container: Full tag of the container used to run the assembler, with a link to the container location in Docker Hub [2].

# LMAS Report

LMAS comes pre-packaged with the JS source code for the interactive report, available in the resources/ folder. The source code for the report is available in the LMAS.js repository [32]. It was built with the JavaScript frameworks React [33] (version 16.8.0) and Material-UI [34] (version 4.11.00). All interactive charts were rendered with the graph visualization library Plotly.js [35] (version 1.57.1) through its React component, react-plotly [36] (version 2.5.0).

# ZymoBIOMICS microbial community standards

The “get_data.sh” bash script file provided with LMAS downloads the ZymoBIOMICS Microbial Community Standard data and saves it in the “data” folder, in conformation with the default parameters. The simulated samples and all reference replicons saved in a singular multi-sequence fasta are publicly available in Zenodo under the DOI <https://doi.org/10.5281/zenodo.4588969>.

## Reference Sequences

The complete bacterial genomes and plasmid sequences for the Microbial Community Standards were obtained from ZymoBIOMICS’ Amazon Simple Storage Service, available at <https://s3.amazonaws.com/zymo-files/BioPool/ZymoBIOMICS.STD.refseq.v2.zip>.

For the analysis of LMAS results, the complete ZymoBIOMICS’ reference genomes were annotated with PROKKA [37] (version 1.14.5), using the species-specific database for each reference sequence when available. The number of tRNA, rRNA and mobile element coding genes is available in [Supplemental Table S19](https://docs.google.com/spreadsheets/u/0/d/1D0_QM8bycHmDTDm6LkZyr2qWszREmtBH8OsuNplnDpw/edit). Pairwise comparisons among the set of reference replicons were conducted by calculating the Average Nucleotide Identity (ANI) through BLASTn (version 2.12.0) [38] using pyani [39] (version 0.2.11) [40]. The results are available in [Supplemental Table S23](https://docs.google.com/spreadsheets/u/0/d/1D0_QM8bycHmDTDm6LkZyr2qWszREmtBH8OsuNplnDpw/edit).

## Real Sequencing Data

The real paired-end Illumina sequencing data for the ZymoBIOMICS Microbial Community Standards, both evenly and logarithmic distributed, was obtained from the PRJEB29504 study accession [41]. The evenly distributed community standard, containing 8.5 million read pairs, is available under the ERR2984773 accession, and the logarithmically distributed sample, containing 47.5 million read pairs, is available under the accession ERR2935805.

## Mock Sequencing Data

A set of simulated samples were generated from the genomes in the ZymoBIOMICS standard through the InSilicoSeq sequence simulator (version 1.5.2) [42], including both even and logarithmic distribution, with and without Illumina error model. The error model was obtained from each corresponding real sample depending on the distribution and used to generate the mock data with matching characteristics, including read number and abundance of species in the community ([Supplemental Table S4](https://docs.google.com/spreadsheets/u/0/d/1D0_QM8bycHmDTDm6LkZyr2qWszREmtBH8OsuNplnDpw/edit)).

## Taxonomic composition

The taxonomic composition of the ZymoBIOMICS standard samples, both real and mocks was determined through Kraken2 [43] using the Standard Database (<https://genome-idx.s3.amazonaws.com/kraken/k2_standard_20210517.tar.gz>). The following command was used: “kraken2 --output $sample.kraken --report $sample.kraken_report --memory-mapping --paired --gzip-compressed $fastq_pair[0] $fastq_pair[1]” where $sample is the sample name, $fastq_pair[0] contains the forward-facing reads, and $fastq_pair[1] the reverse-facing reads.

The processing of the kraken reports was performed through custom python code [44] where all the percentage of reads that matched for the species in the dataset were saved, as well as the percentage of unclassified reads. For the Lactobacillus fermentum, as in the Standard Kraken database no general Species level classification is available, the percentage of reads was calculated as the sum of all reads aligning to one of the *L. fermentum* subspecies. The rest of the reads that were classified as any other species were saved conjunctively as “Other”. [Supplemental Table S20](https://docs.google.com/spreadsheets/u/0/d/1D0_QM8bycHmDTDm6LkZyr2qWszREmtBH8OsuNplnDpw/edit) contains the percentage of classified reads for each of the species in the community, as well as “other” and unclassified reads.

## Assessment of Assembly Success

The complete set of results for 3 LMAS runs for the raw sequence reads of mock communities with an even and logarithmic distribution of species, from real sequencing runs [41] and simulated read datasets, with and without error, matching the intended distribution of species in each sample for the eight bacterial genomes and four plasmids of the ZymoBIOMICS Microbial Community Standards as reference is available in [Supplemental Table S21](https://docs.google.com/spreadsheets/u/0/d/1D0_QM8bycHmDTDm6LkZyr2qWszREmtBH8OsuNplnDpw/edit) and [S22](https://docs.google.com/spreadsheets/u/0/d/1D0_QM8bycHmDTDm6LkZyr2qWszREmtBH8OsuNplnDpw/edit). For the assessment of the assembly success for each sample, the different metrics for all LMAS runs were combined and descriptive statistics, such as the average value, standard deviation, minimum and maximum, were obtained through Python’s Pandas describe function [45,46]. Both global and reference based for each assembler, each reference replicon and each sample (ENN - evenly distributed without error model; EMS - evenly distributed with Illumina MiSeq error model; ERR2984773 - real evenly distributed Illumina MiSeq sample, LNN - logarithmically distributed without error model; LHS - logarithmically distributed with Illumina HiSeq error model; ERR2935805 - real logarithmically distributed Illumina HiSeq sample). For descriptive statistics on several assembler by each assembly type (genomic or metagenomic) and each assembler algorithm (single or multiple k-mer), the use of median was preferred due to its higher robustness against outliers and the high range of the distribution of the results. Plotly [35] was used to compute the graphs aggregating the results obtained. The jupyter notebooks [47] with the data processing and all resulting files are available at [44].

The top result of each assembler for each sample was selected, based on the following criteria:

- For the number of uncalled bases, number of misassembled contigs and number of misassembly events, the lower the value, the better, with the exception of 0 for the number of contigs;
- For the percentage of mapped reads and N50, the higher the value, the better;
- The number of basepairs, the best results was the one closest to the target value of the number of basepairs in the reference replicons.

For reference-specific metrics, in addition to the ones stated above when applicable (Number of contigs produced, number of uncalled bases, number of misassembled contigs and number of misassembly events), the following criteria were used:

- For the L90 metric, the lower value was better, with the exception of 0;
- For LSA, NA, NG, breadth of coverage, identity and lowest identity, the higher the value the better;
- For multiplicity, parsimony and validity, the closer to 1, the better.

To obtain the worst value in each metric, the opposite criteria were used. The normalised score for each metric was obtained from the best result for each assembler in each sample through [Equation 1](#kix.bcx1rgrx4uqg). For the assessment of assembler consistency, each contig for each assembler was considered the same as its size was exactly the same in each LMAS run.

$$\left\{ \begin{aligned} 1-\frac{x}{\min\left( X \right)} \text{if maximum value is best} \\ \frac{x}{min(X)} \text{if minimum value is best} \\ 1-\frac{\left| x-T \right|}{T} \text{if target value is best} \end{aligned} \right.$$

Where $x$ is the given value of a metric for an assembler, $X$ the list of values for that metric for all assemblers, and T the target value.

**Equa****tion 1:** Formula for the normalized score

[Figure S3:](#kix.asr5bpci2qnb) Performance per reference of genomic and metagenomic assemblers for the evenly distributed samples in the ZymoBIOMICS Microbial Community Standards dataset. For each sample in the dataset and for the 3 runs, the best and worst scores for each assembler category were selected: genomic (in blue) and metagenomic (in red). The results for each global assembly metric was normalised, with 1 representing the best result, and 0 the worst.

[**Figure S4:**](#_gv81jwtwaq28) **Assembler performance per reference for** **the ZymoBIOMICS Microbial Community Standards dataset for sample ENN.** The best score for each assembler was selected for 3 LMAS runs. The results for each global assembly metric was normalised, with 1 representing the best result, and 0 the worst. The following assemblers are represented: GATBMiniaPipeline: dark blue, IDBA-UD: light blue, MEGAHIT: dark green, metaSPAdes: light green, SKESA: yellow, SPAdes: orange, Unicycler: red.

[**Figure S5:**](#_7okvads4w8tb) **Assembler performance per reference for** **the ZymoBIOMICS Microbial Community Standards dataset for sample EMS.** The best score for each assembler was selected for 3 LMAS runs. The results for each global assembly metric was normalised, with 1 representing the best result, and 0 the worst. The following assemblers are represented: GATBMiniaPipeline: dark blue, IDBA-UD: light blue, MEGAHIT: dark green, metaSPAdes: light green, SKESA: yellow, SPAdes: orange, Unicycler: red.

[Figure S6:](#_j1rve1lon19p) Assembler performance per reference for the ZymoBIOMICS Microbial Community Standards dataset for sample ERR2984773. The best score for each assembler was selected for 3 LMAS runs. The results for each global assembly metric was normalised, with 1 representing the best result, and 0 the worst. The following assemblers are represented: GATBMiniaPipeline: dark blue, IDBA-UD: light blue, MEGAHIT: dark green, metaSPAdes: light green, SKESA: yellow, SPAdes: orange, Unicycler: red.

[Figure S7](#_d2m3aygsggfx): Genome fragmentation for each reference replicon of the ZimoBIOMICS community standards dataset for the logarithmically distributed samples. Genome fragmentation for the 3 LMAS runs is represented by the number of contigs and breadth of coverage of the reference per assembler for the logarithmically distributed samples: LNN (logarithmically distributed without error model, identified by a circle), LHS (logarithmically distributed with Illumina HiSeq error model, identified by a square) and ERR2935805 (real Illumina HiSeq sample, identified by a diamond). Each assembler is identified with the following colour scheme - dark blue: Unicycler, light blue: SPAdes, dark green: SKESA, light green: metaSPAdes, yellow: MEGAHIT, orange: IDBA-UD, red: GATBMiniaPipeline.

**Figure S8:** Genome fragmentation for each reference replicon of the BMock12 community standards dataset sample. Genome fragmentation is represented by the number of contigs and breadth of coverage of the reference per assembler. Each assembler is identified with the following colour scheme - dark blue: Unicycler, light blue: SPAdes, dark green: SKESA, light green: metaSPAdes, yellow: MEGAHIT, orange: IDBA-UD, red: GATBMiniaPipeline. Each reference replicon is identified by its IMG Taxon ID: 2615840527, Muricauda sp; 2615840533, Thioclava sp; 2615840601, Cohaesibacter sp; 2615840646, Propionibacteriaceae bacterium, 2615840697, Marinobacter sp LV10R510-8; 2616644829, Marinobacter sp LV10MA510-1; 2617270709, Psychrobacter sp; 2623620557, Micromonospora echinaurantiaca; 2623620567, Micromonospora echinofusca; 2623620609, Micromonospora coxensis; 2623620617, Halomonas sp. HL-4; and 2623620618, Halomonas sp. HL-93.

**Figure S9: Location of gaps in comparison to the reference sequence, per assembler, for each reference replicon of the BMock12 community standards datasets.** The resulting plot contains the gaps obtained for GATBMiniaPipeline, IDBA-UD, MEGAHIT, metaSPAdes, SKESA, SPAdes and Unicycler assemblers.

## Resource requirements differ greatly

Regarding computational resources, there is a disparity in usage for the evenly and logarithmically distributed samples ([Figure S2](https://docs.google.com/document/d/17RXbgvaQ9qDRmXza4KhkRsptQte53DYaz-3BMMSWrnw/edit#bookmark=kix.mfolvo56us4)), with the latter having more resource-intensive requirements possibly due mostly to a higher number of reads. The resource usage also varied greatly by assembler, with multiple k-mer dBG (SPAdes, metaSPAdes, MEGAHIT, SKESA, IDBA-UD, GATBMiniaPipeline and Unicycler) having overall higher resource usage. ABySS performance was inconsistent, having reached a maximum of 1412 CPU hours to produce an assembly (sample ERR2984773), resulting in a run time of 35.52 hours. MetaHipmer2 was the assembler with the highest memory usage, reaching a maximum of 68.7 GB.

[**Figure S2:**](#7hpfifk13621) **Computational resources used by each assembler for the evenly and logarithmically distributed samples.** Each plot describes the distribution of resource consumption for 3 LMAS runs for the ZymoBIOMICS microbial community standard dataset for the following metrics: A) CPU/hour, B) Maximum memory in GB; C) Data written to disk in GB; D) Data read from dist in GB; E) Run time in hours. The mean for all samples and all assemblers is indicated in red. The samples are indicated as follows: ENN: dark blue, EMS: teal, ERR2984773: green, LNN: light green, LHS: yellow, ERR2935805: light orange.

# References

1. : Parameters — LMAS 0.1 documentation. https://lmas.readthedocs.io/en/latest/user/parameters.html Accessed 2022 Apr 4.

2. : Docker Hub Container Image Library | App Containerization. https://hub.docker.com/ Accessed 2022 Apr 4.

3. Masella AP, Bartram AK, Truszkowski JM, Brown DG, Neufeld JD. PANDAseq: paired-end assembler for illumina sequences. *BMC Bioinformatics*. 2012; doi: 10.1186/1471-2105-13-31.

4. Jackman SD, Vandervalk BP, Mohamadi H, Chu J, Yeo S, Hammond SA, et al.. ABySS 2.0: resource-efficient assembly of large genomes using a Bloom filter. *Genome Res*. 2017; doi: 10.1101/gr.214346.116.

5. : ABySS. https://github.com/bcgsc/abyss (2022). Accessed 2022 Apr 4.

6. Benoit G, Lavenier D, Lemaitre C, Rizk G. Bloocoo, a memory efficient read corrector.

7. Chikhi R, Rizk G. Space-efficient and exact de Bruijn graph representation based on a Bloom filter. *Algorithms Mol Biol*. 2013; doi: 10.1186/1748-7188-8-22.

8. Chikhi R, Limasset A, Medvedev P. Compacting de Bruijn graphs from sequencing data quickly and in low memory. *Bioinformatics*. 2016; doi: 10.1093/bioinformatics/btw279.

9. Sahlin K, Vezzi F, Nystedt B, Lundeberg J, Arvestad L. BESST - Efficient scaffolding of large fragmented assemblies. *BMC Bioinformatics*. 2014; doi: 10.1186/1471-2105-15-281.

10. : GATB/gatb-minia-pipeline. https://github.com/GATB/gatb-minia-pipeline (2022). Accessed 2022 Apr 4.

11. Peng Y, Leung HCM, Yiu SM, Chin FYL. IDBA-UD: a de novo assembler for single-cell and metagenomic sequencing data with highly uneven depth. *Bioinformatics*. 2012; doi: 10.1093/bioinformatics/bts174.

12. Peng Y: loneknightpy/idba. https://github.com/loneknightpy/idba (2022). Accessed 2022 Apr 4.

13. Bushnell B, Rood J, Singer E. BBMerge – Accurate paired shotgun read merging via overlap. Biggs PJ, editor. *PLoS ONE*. 2017; doi: 10.1371/journal.pone.0185056.

14. Li D, Liu C-M, Luo R, Sadakane K, Lam T-W. MEGAHIT: an ultra-fast single-node solution for large and complex metagenomics assembly via succinct de Bruijn graph. *Bioinformatics*. 2015; doi: 10.1093/bioinformatics/btv033.

15. Li D: MEGAHIT. https://github.com/voutcn/megahit (2022). Accessed 2022 Apr 4.

16. Georganas E, Egan R, Hofmeyr S, Goltsman E, Arndt B, Tritt A, et al.. Extreme Scale De Novo Metagenome Assembly. *SC18: International Conference for High Performance Computing, Networking, Storage and Analysis*. Dallas, TX, USA: IEEE;

17. : berkeleylab / mhm2 — Bitbucket. https://bitbucket.org/berkeleylab/mhm2/src/master/ Accessed 2022 Apr 4.

18. Bankevich A, Nurk S, Antipov D, Gurevich AA, Dvorkin M, Kulikov AS, et al.. SPAdes: A New Genome Assembly Algorithm and Its Applications to Single-Cell Sequencing. *Journal of Computational Biology*. 2012; doi: 10.1089/cmb.2012.0021.

19. Nurk S, Meleshko D, Korobeynikov A, Pevzner PA. metaSPAdes: a new versatile metagenomic assembler. *Genome Res*. 2017; doi: 10.1101/gr.213959.116.

20. : About SPAdes. https://github.com/ablab/spades (2022). Accessed 2022 Apr 4.

21. : Minia. https://github.com/GATB/minia (2022). Accessed 2022 Apr 4.

22. Souvorov A, Agarwala R, Lipman DJ. SKESA: strategic k-mer extension for scrupulous assemblies. *Genome Biol*. 2018; doi: 10.1186/s13059-018-1540-z.

23. Wick RR, Judd LM, Gorrie CL, Holt KE. Unicycler: Resolving bacterial genome assemblies from short and long sequencing reads. Phillippy AM, editor. *PLoS Comput Biol*. 2017; doi: 10.1371/journal.pcbi.1005595.

24. Wick R: Unicycler. https://github.com/rrwick/Unicycler (2022). Accessed 2022 Apr 4.

25. Zerbino DR. Using the Velvet de novo assembler for short-read sequencing technologies. *Curr Protoc Bioinformatics*. 2010; doi: 10.1002/0471250953.bi1105s31.

26. Seemann T: VelvetOptimiser: automate your Velvet assemblies. https://github.com/tseemann/VelvetOptimiser (2021). Accessed 2022 Apr 4.

27. Li H. Minimap2: pairwise alignment for nucleotide sequences. Birol I, editor. *Bioinformatics*. 2018; doi: 10.1093/bioinformatics/bty191.

28. Kosugi S, Momozawa Y, Liu X, Terao C, Kubo M, Kamatani Y. Comprehensive evaluation of structural variation detection algorithms for whole genome sequencing. *Genome Biol*. 2019; doi: 10.1186/s13059-019-1720-5.

29. Bradnam KR, Fass JN, Alexandrov A, Baranay P, Bechner M, Birol I, et al.. Assemblathon 2: evaluating de novo methods of genome assembly in three vertebrate species. *GigaSci*. 2013; doi: 10.1186/2047-217X-2-10.

30. Gurevich A, Saveliev V, Vyahhi N, Tesler G. QUAST: quality assessment tool for genome assemblies. *Bioinformatics*. 2013; doi: 10.1093/bioinformatics/btt086.

31. Wick RR, Holt KE. Benchmarking of long-read assemblers for prokaryote whole genome sequencing. *F1000Res*. 2021; doi: 10.12688/f1000research.21782.4.

32. : LMAS Report. https://github.com/B-UMMI/LMAS.js (2021). Accessed 2022 Apr 4.

33. : React – A JavaScript library for building user interfaces. https://reactjs.org/ Accessed 2022 Apr 4.

34. : MUI: The React component library you always wanted. https://mui.com/pt/ Accessed 2022 Apr 4.

35. : Plotly. https://plotly.com/javascript/ Accessed 2022 Apr 4.

36. : React. https://plotly.com/javascript/react/ Accessed 2022 Apr 4.

37. Seemann T. Prokka: rapid prokaryotic genome annotation. *Bioinformatics*. 2014; doi: 10.1093/bioinformatics/btu153.

38. Camacho C, Coulouris G, Avagyan V, Ma N, Papadopoulos J, Bealer K, et al.. BLAST+: architecture and applications. *BMC Bioinformatics*. 2009; doi: 10.1186/1471-2105-10-421.

39. Pritchard L: pyani. https://github.com/widdowquinn/pyani (2022). Accessed 2022 Apr 4.

40. Pritchard L, Glover RH, Humphris S, Elphinstone JG, Toth IK. Genomics and taxonomy in diagnostics for food security: soft-rotting enterobacterial plant pathogens. *Anal Methods*. 2016; doi: 10.1039/C5AY02550H.

41. Nicholls SM, Quick JC, Tang S, Loman NJ. Ultra-deep, long-read nanopore sequencing of mock microbial community standards. *GigaScience*. 2019; doi: 10.1093/gigascience/giz043.

42. Gourlé H, Karlsson-Lindsjö O, Hayer J, Bongcam-Rudloff E. Simulating Illumina metagenomic data with InSilicoSeq. Hancock J, editor. *Bioinformatics*. 2019; doi: 10.1093/bioinformatics/bty630.

43. Wood DE, Lu J, Langmead B. Improved metagenomic analysis with Kraken 2. *Genome Biol*. 2019; doi: 10.1186/s13059-019-1891-0.

44. : LMAS Manuscript Analysis. https://github.com/B-UMMI/LMAS_Manuscript_Analysis (2022). Accessed 2022 Apr 4.

45. : pandas - Python Data Analysis Library. https://pandas.pydata.org/ Accessed 2022 Apr 4.

46. : pandas.DataFrame.describe — pandas 1.4.2 documentation. https://pandas.pydata.org/pandas-docs/stable/reference/api/pandas.DataFrame.describe.html Accessed 2022 Apr 4.

47. : Project Jupyter. https://jupyter.org Accessed 2022 Apr 4.

####

Figures
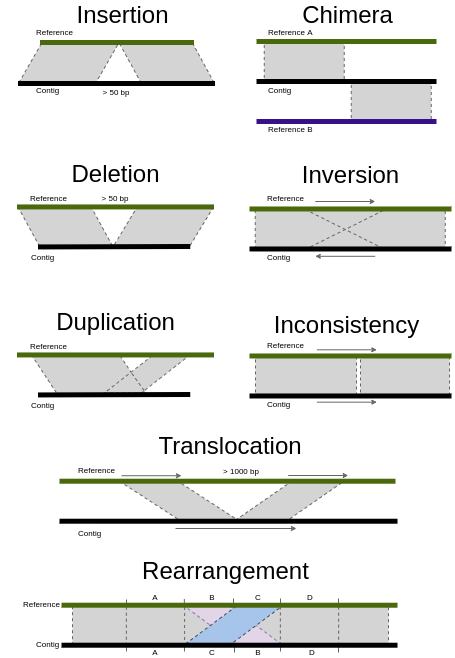


#### Figure S1

####
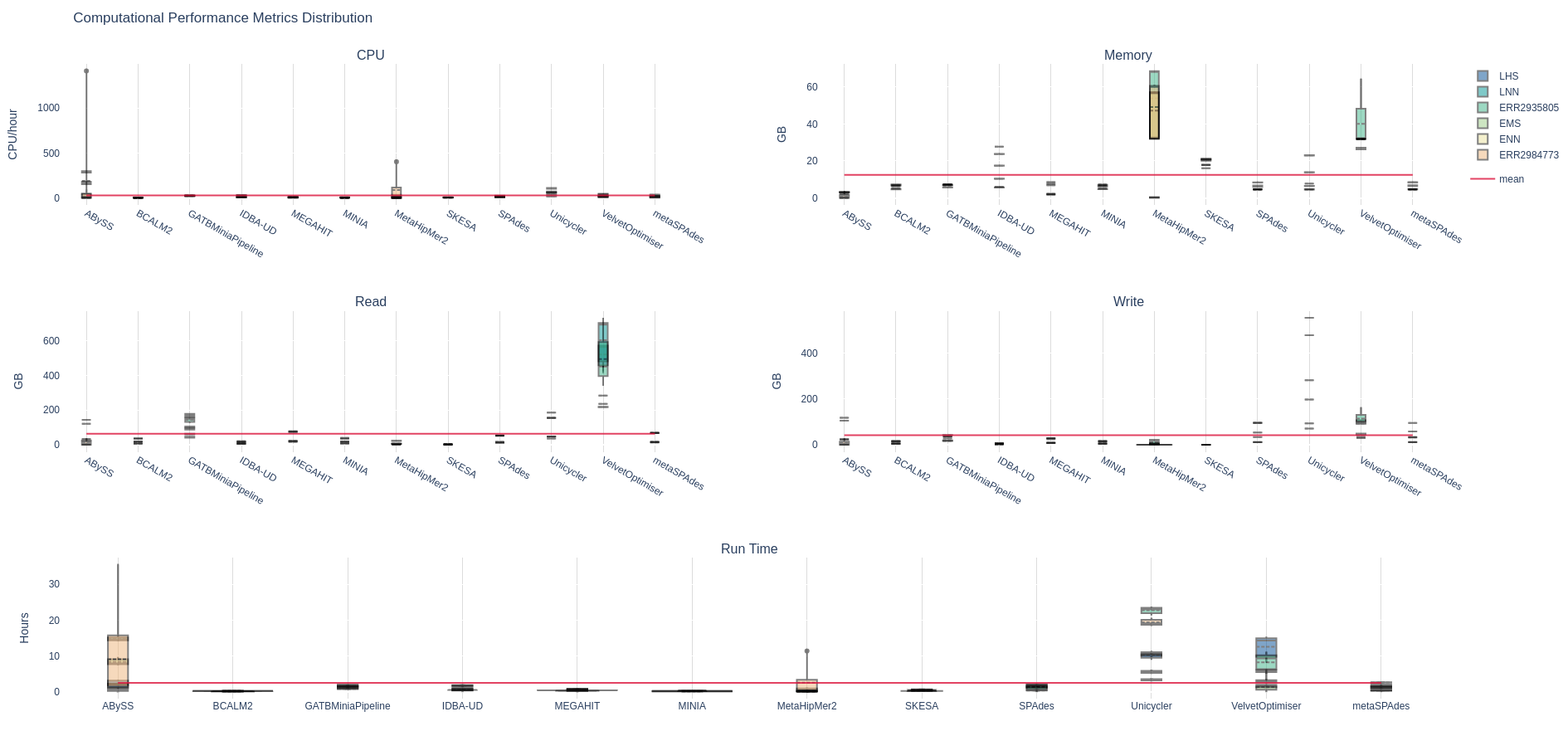
Figure S2

#
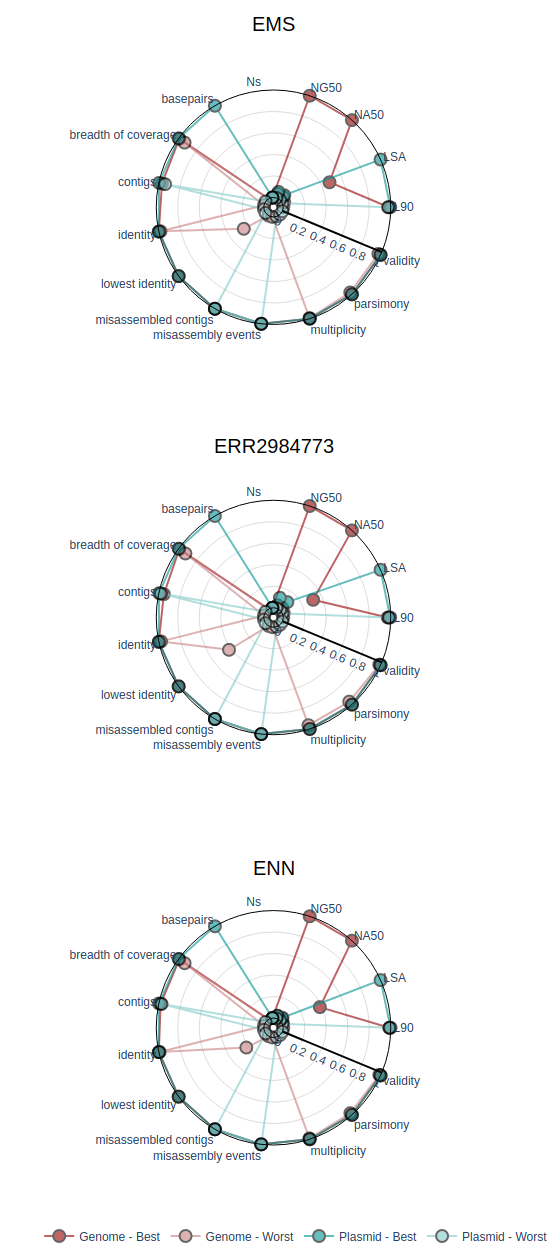


#### Figure S3

####

####
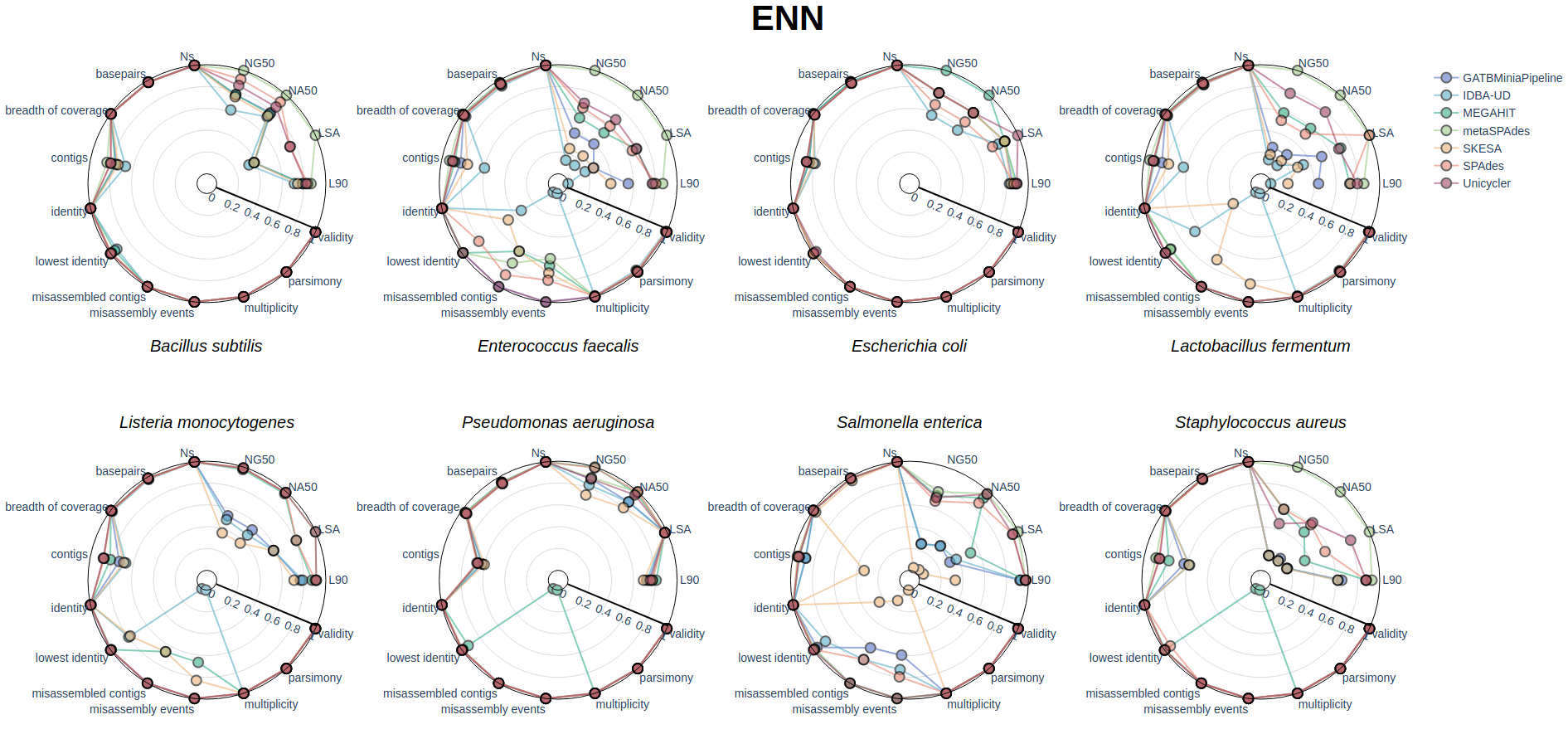


#### Figure S4


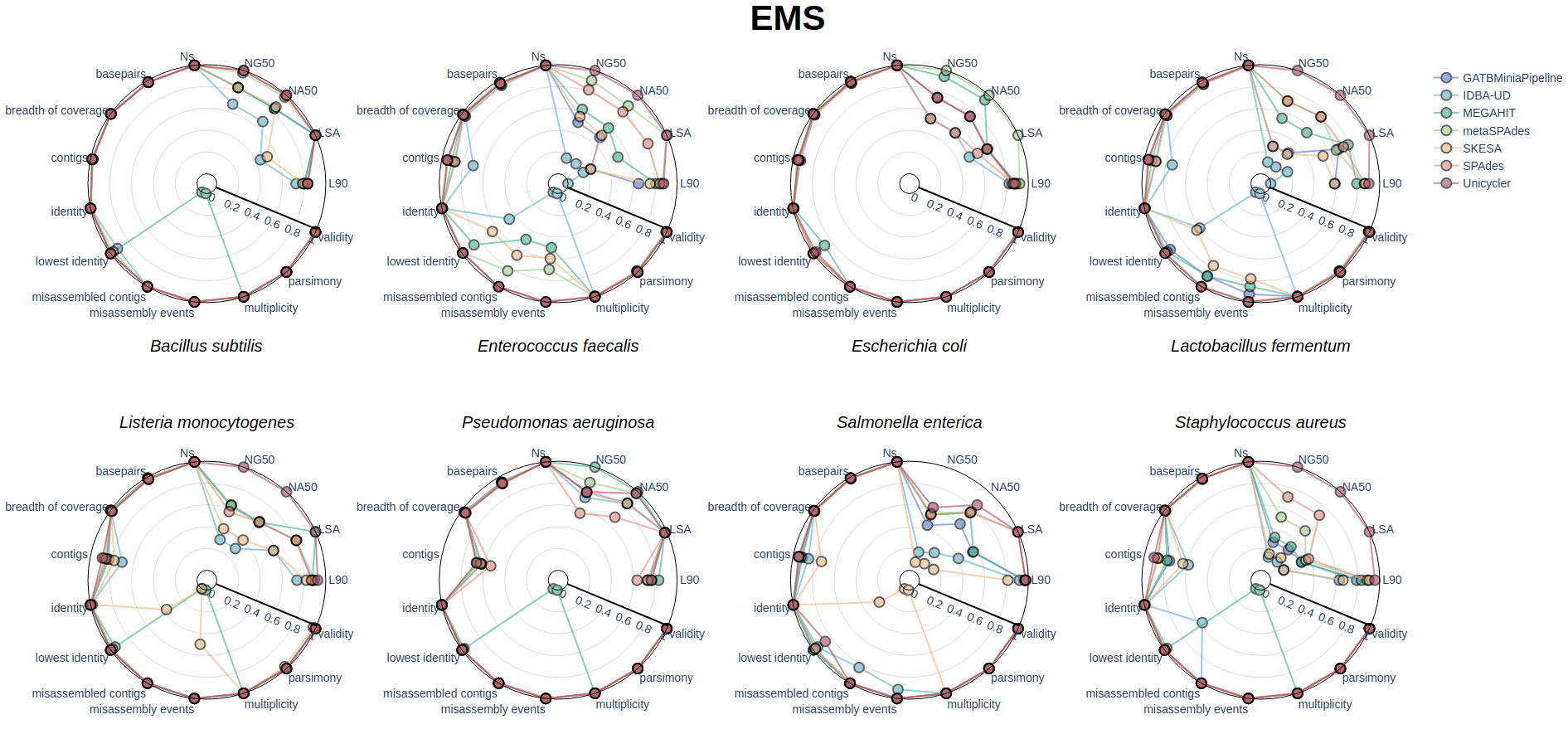


#### Figure S5


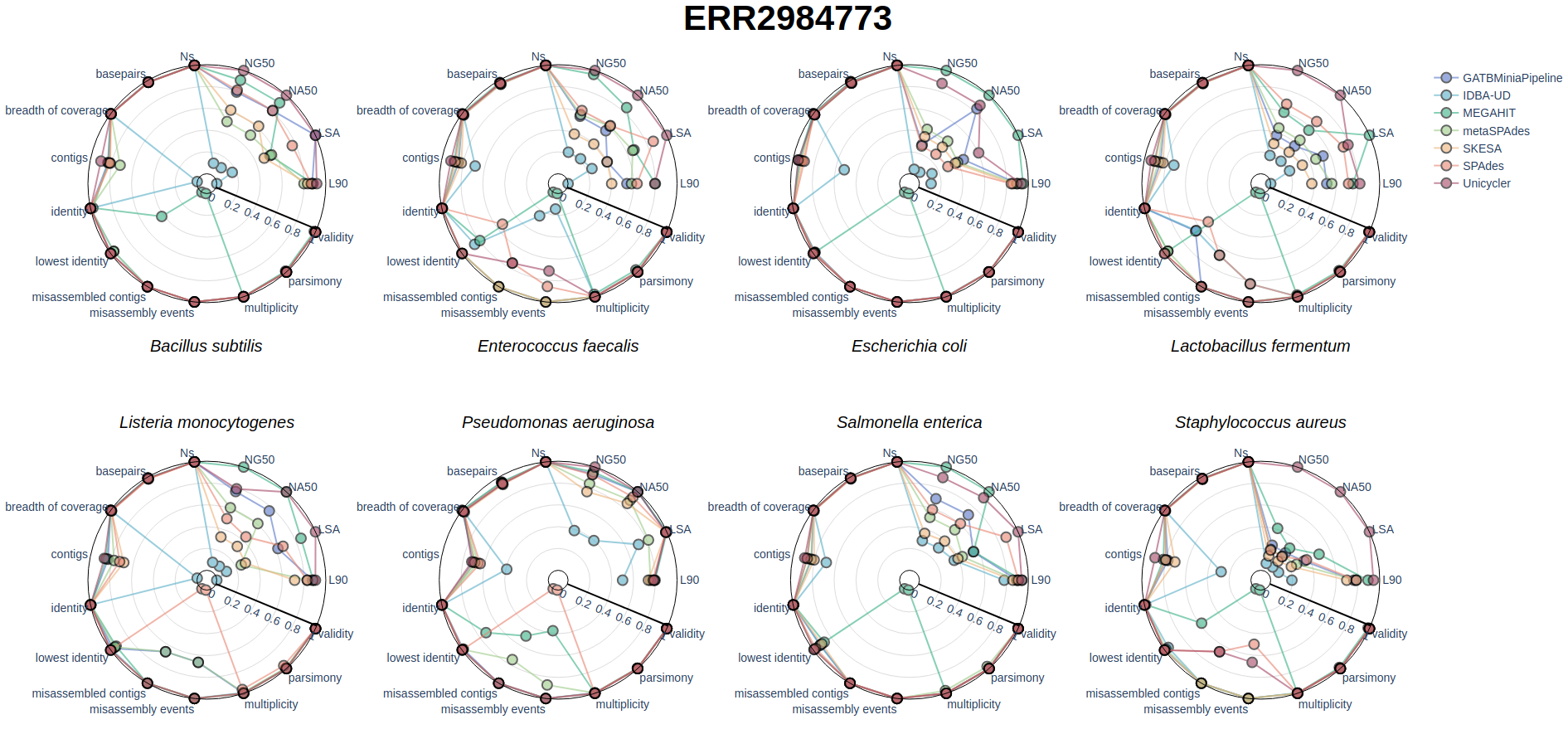


#### Figure S6


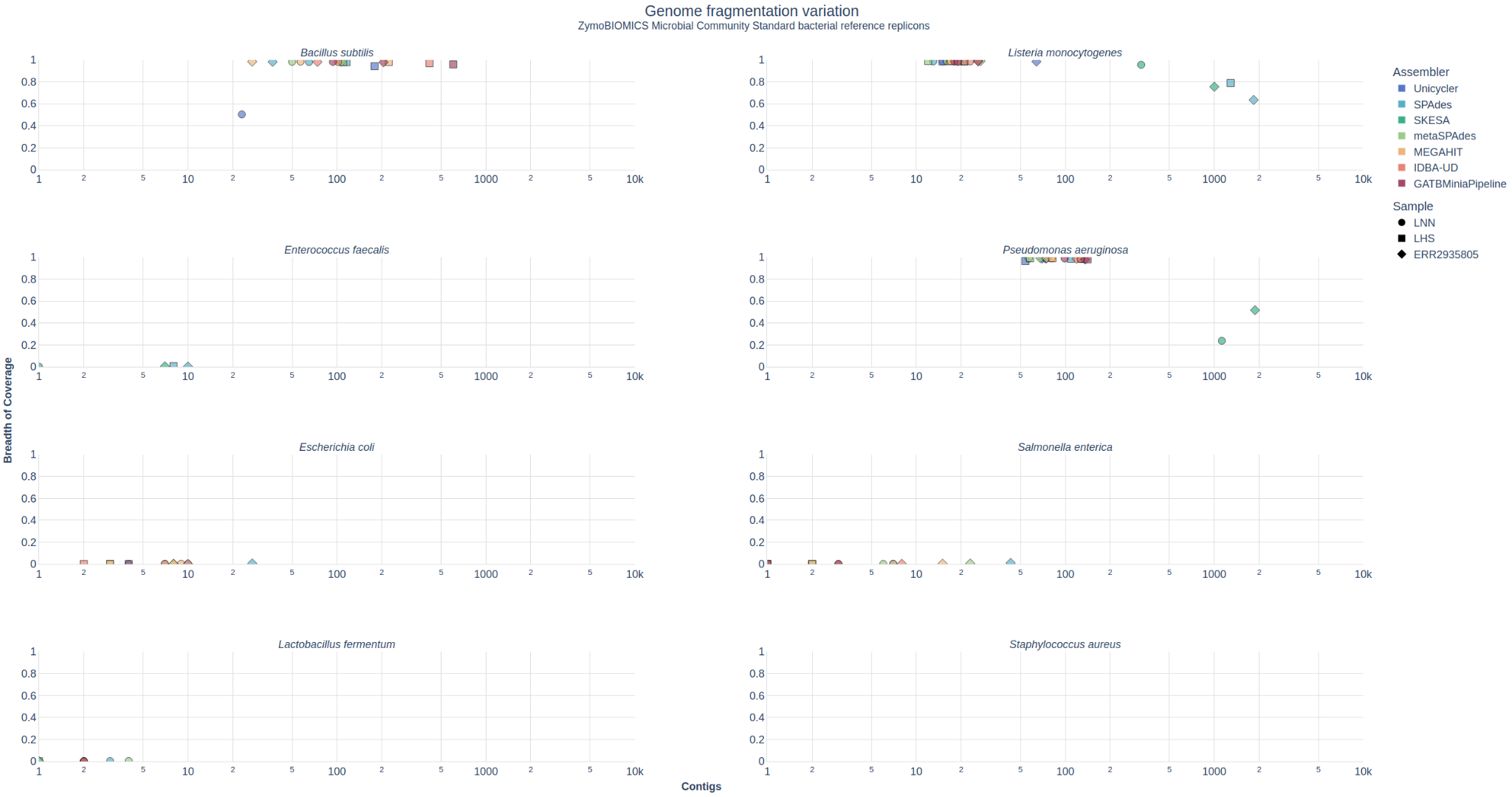


Figure S7


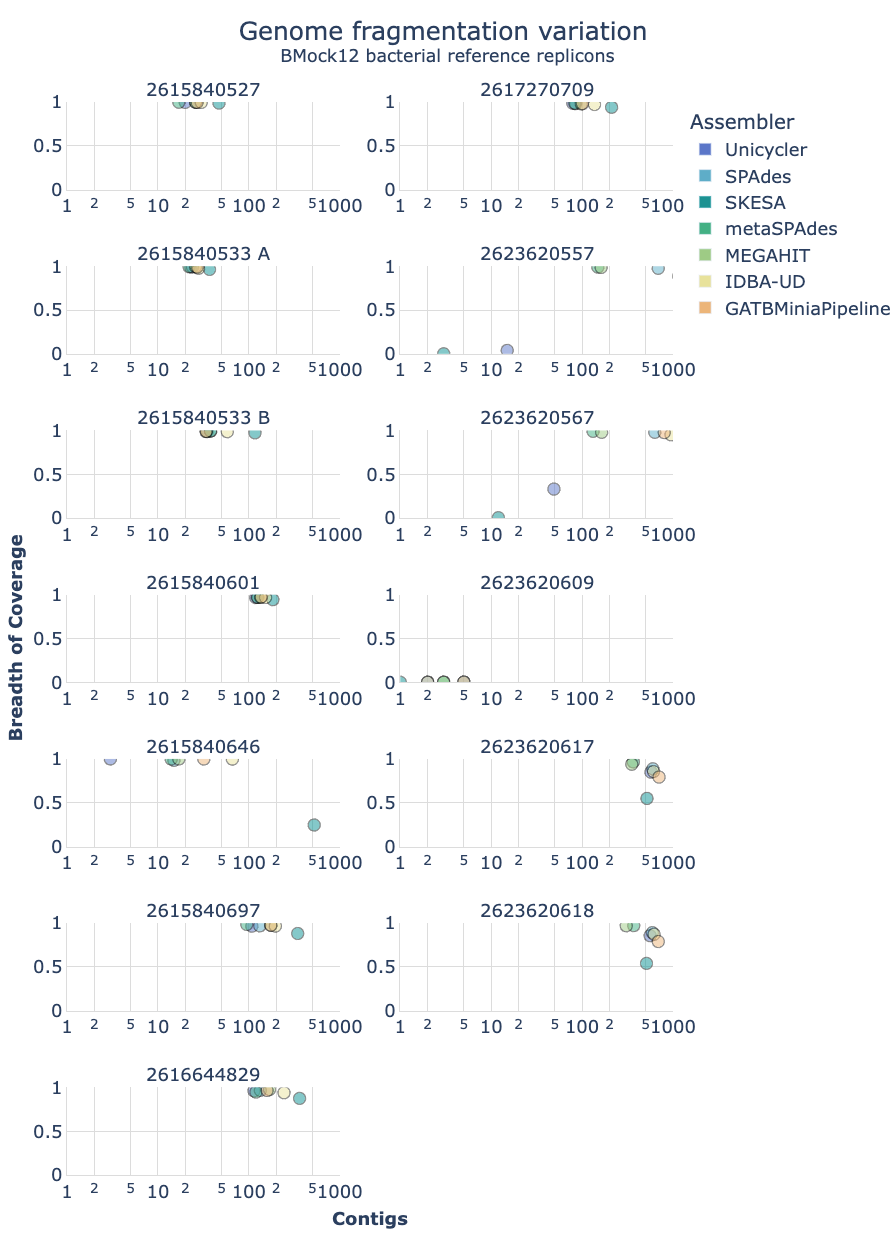


Figure S8


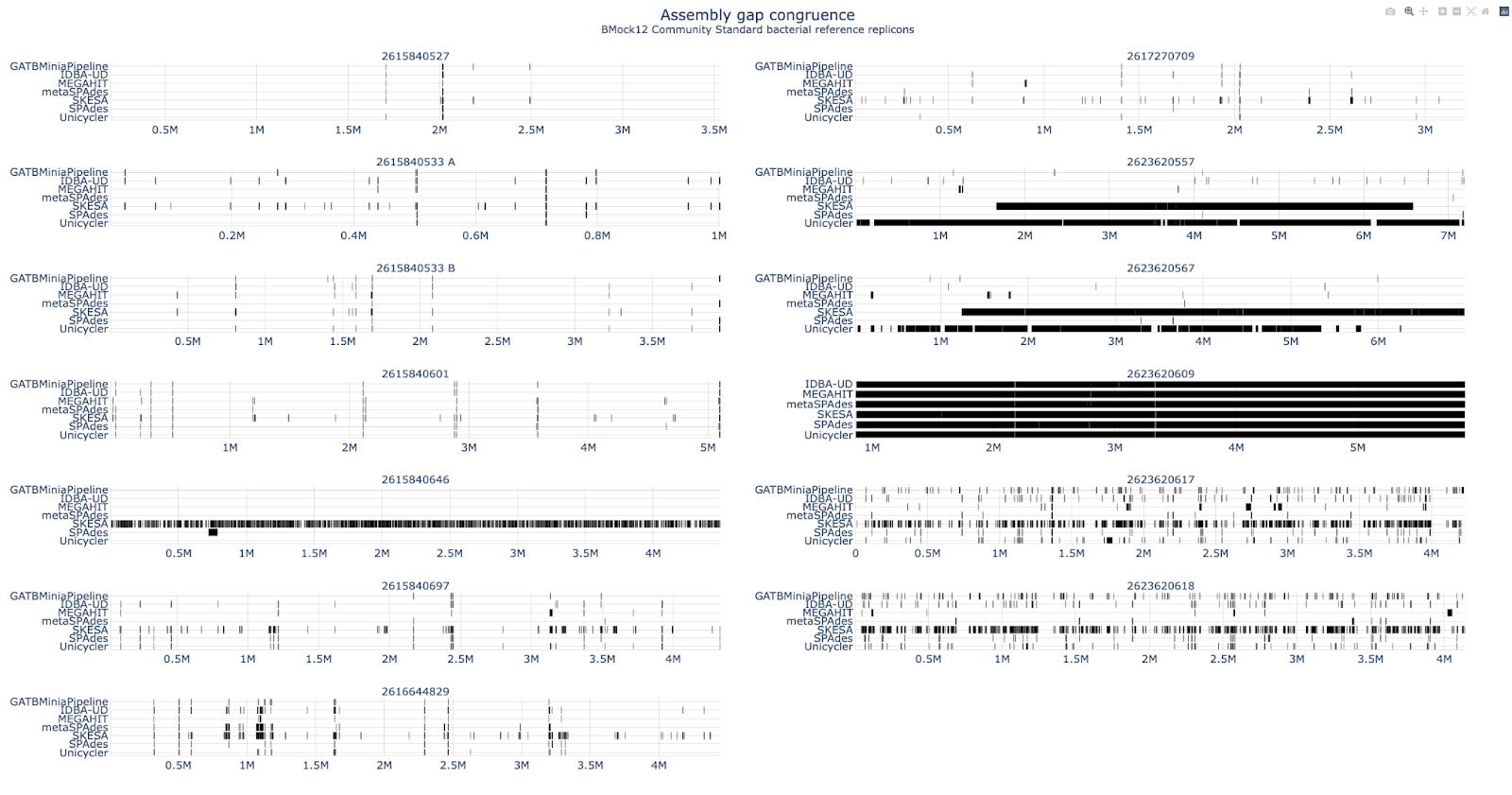


Figure S9

# Tables

#### [Table S1:](https://docs.google.com/spreadsheets/u/0/d/1D0_QM8bycHmDTDm6LkZyr2qWszREmtBH8OsuNplnDpw/edit) Tools available for the *de novo* assembly of prokaryotic genomes. For each tool, its publication is indicated, if available, as well as the assembly algorithm implemented if it was developed explicitly to handle metagenomic datasets. The tools are ordered by the date of the last update, with the source code indicated when available. The tools incorporated in LMAS are indicated as such.

#### [Table S2:](https://docs.google.com/spreadsheets/u/0/d/1D0_QM8bycHmDTDm6LkZyr2qWszREmtBH8OsuNplnDpw/edit) Comparison of metrics and features of LMAS with QUAST and MetaQUAST.

[**Table S3:**](https://docs.google.com/spreadsheets/u/0/d/1D0_QM8bycHmDTDm6LkZyr2qWszREmtBH8OsuNplnDpw/edit) **The ZymoBIOMICS Microbial Community Standard datasets.** Set of raw sequence reads used as input in LMAS of mock communities with an even and logarithmic distribution of species, from real sequencing runs and simulated read datasets, with and without error, matching the intended distribution of species in ZymoBIOMICS Microbial Community Standard.

#### [Table S4:](https://docs.google.com/spreadsheets/u/0/d/1D0_QM8bycHmDTDm6LkZyr2qWszREmtBH8OsuNplnDpw/edit) Microbial composition of the ZymoBIOMICS microbial community standard dataset with Even and Logarithmic distribution of species. Theoretical microbial composition of the standards, and the corresponding number of reads generated for each replicon.

#### [Table S5:](https://docs.google.com/spreadsheets/u/0/d/1D0_QM8bycHmDTDm6LkZyr2qWszREmtBH8OsuNplnDpw/edit) Global quality metrics variation in three LMAS runs for sample ENN per assembler. The average calculated for all samples in the dataset for the 3 independent LMAS runs, followed by the minimum and maximum values obtained, are presented for each metric for each assembler.

#### [Table S6:](https://docs.google.com/spreadsheets/u/0/d/1D0_QM8bycHmDTDm6LkZyr2qWszREmtBH8OsuNplnDpw/edit) Global quality metrics variation in three LMAS runs for sample EMS per assembler. The average calculated for all samples in the dataset for the 3 independent LMAS runs, followed by the minimum and maximum values obtained, are presented for each metric for each assembler.

#### [Table S7:](https://docs.google.com/spreadsheets/u/0/d/1D0_QM8bycHmDTDm6LkZyr2qWszREmtBH8OsuNplnDpw/edit) Global quality metrics variation in three LMAS runs for sample ERR2984773 per assembler. The average calculated for all samples in the dataset for the 3 independent LMAS runs, followed by the minimum and maximum values obtained, are presented for each metric for each assembler.

#### [Table S8:](https://docs.google.com/spreadsheets/u/0/d/1D0_QM8bycHmDTDm6LkZyr2qWszREmtBH8OsuNplnDpw/edit) Global quality metrics variation in three LMAS runs for sample LNN per assembler. The average calculated for all samples in the dataset for the 3 independent LMAS runs, followed by the minimum and maximum values obtained, are presented for each metric for each assembler.

#### [Table S9:](https://docs.google.com/spreadsheets/u/0/d/1D0_QM8bycHmDTDm6LkZyr2qWszREmtBH8OsuNplnDpw/edit) Global quality metrics variation in three LMAS runs for sample LHS per assembler. The average calculated for all samples in the dataset for the 3 independent LMAS runs, followed by the minimum and maximum values obtained, are presented for each metric for each assembler.

#### [Table S10:](https://docs.google.com/spreadsheets/u/0/d/1D0_QM8bycHmDTDm6LkZyr2qWszREmtBH8OsuNplnDpw/edit) Global quality metrics variation in three LMAS runs for sample ERR2935805 per assembler. The average calculated for all samples in the dataset for the 3 independent LMAS runs, followed by the minimum and maximum values obtained, are presented for each metric for each assembler.

#### [Table S11:](https://docs.google.com/spreadsheets/u/0/d/1D0_QM8bycHmDTDm6LkZyr2qWszREmtBH8OsuNplnDpw/edit) Inconsistent contigs produced by the assemblers in 3 LMAS runs. For each assembler, the total number of contigs produced over the 3 runs of the LMAS workflow is indicated, as well as the contigs present in only two and a single run.

#### [Table S12:](https://docs.google.com/spreadsheets/u/0/d/1D0_QM8bycHmDTDm6LkZyr2qWszREmtBH8OsuNplnDpw/edit) Global assembly metrics for single and multiple k-mer dBg assemblers. The median and the minimum and maximum values obtained are presented for each metric for all samples in 3 runs of LMAS. Single k-mer bBg assemblers: ABySS, and minia. Multiple k-mer bBg assembler: GATBMiniaPipeline, IDBA-UD, MEGAHIT, MetaHipMer2, metaSPAdes, SKESA, SPAdes, Unicycler and VelverOptimiser.

#### [Table S13:](https://docs.google.com/spreadsheets/u/0/d/1D0_QM8bycHmDTDm6LkZyr2qWszREmtBH8OsuNplnDpw/edit) Global assembly metrics for genomic and metagenomic multiple k-mer dBg assemblers. The median and the minimum and maximum values obtained are presented for each metric for all samples in 3 runs of LMAS. Genomic assemblers: SKESA, SPAdes and Unicycler. Metagenomic assemblers: GATBMiniaPipeline, IDBA-UD, MEGAHIT and metaSPAdes.

#### [Table S14:](https://docs.google.com/spreadsheets/u/0/d/1D0_QM8bycHmDTDm6LkZyr2qWszREmtBH8OsuNplnDpw/edit) Per reference quality metrics variation in three LMAS s for sample ENN per assembler of the ZymoBIOMICS microbial community standard dataset. The average calculated for all samples in the dataset for the 3 independent LMAS runs, followed by the minimum and maximum values obtained, are presented for each metric for each assembler.

####

#### [Table S15:](https://docs.google.com/spreadsheets/u/0/d/1D0_QM8bycHmDTDm6LkZyr2qWszREmtBH8OsuNplnDpw/edit) Per reference quality metrics variation in three LMAS s for sample EMS per assembler of the ZymoBIOMICS microbial community standard dataset. The average calculated for all samples in the dataset for the 3 independent LMAS runs, followed by the minimum and maximum values obtained, are presented for each metric for each assembler.

####

#### [Table S16:](https://docs.google.com/spreadsheets/u/0/d/1D0_QM8bycHmDTDm6LkZyr2qWszREmtBH8OsuNplnDpw/edit) Per reference quality metrics variation in three LMAS s for sample ERR2984773 per assembler of the ZymoBIOMICS microbial community standard dataset. The average calculated for all samples in the dataset for the 3 independent LMAS runs, followed by the minimum and maximum values obtained, are presented for each metric for each assembler.

#### [Table S17:](https://docs.google.com/spreadsheets/u/0/d/1D0_QM8bycHmDTDm6LkZyr2qWszREmtBH8OsuNplnDpw/edit) Inconsistent gaps produced by the assemblers in 3 LMAS runs. For each assembler, the total number of gaps consistently produced in relation to the reference replicons over the 3 runs of the LMAS workflow is indicated, as well as gaps present in only two and a single run.

####

#### [Table S18:](https://docs.google.com/spreadsheets/u/0/d/1D0_QM8bycHmDTDm6LkZyr2qWszREmtBH8OsuNplnDpw/edit) Annotation of consistent gaps produced by the assemblers in 3 LMAS runs.

####

#### [Table S19:](https://docs.google.com/spreadsheets/u/0/d/1D0_QM8bycHmDTDm6LkZyr2qWszREmtBH8OsuNplnDpw/edit) Number of tRNA and rRNA coding sequencing, and mobile elements in ZymoBIOMICS microbial community standard reference replicons. The average calculated for all samples in the dataset for the 3 independent LMAS runs, followed by the minimum and maximum values obtained, are presented for each metric for each assembler.

#### [Table S20:](https://docs.google.com/spreadsheets/u/0/d/1D0_QM8bycHmDTDm6LkZyr2qWszREmtBH8OsuNplnDpw/edit) Taxonomic classification of the ZymoBIOMICS microbial community standard dataset. The classification was performed with Kraken2, using the Standard Database. The results are presented as the percentage of classified reads for the 8 bacterial species in the community, as well as unclassified reads and the group of reads that are classified as species not contained in the community standard.

####

#### [Table S21:](https://docs.google.com/spreadsheets/u/0/d/1D0_QM8bycHmDTDm6LkZyr2qWszREmtBH8OsuNplnDpw/edit) Global assembly metrics for dBg assemblers with single and multiple k-mer algorithms.

####

#### [Table S22:](https://docs.google.com/spreadsheets/u/0/d/1D0_QM8bycHmDTDm6LkZyr2qWszREmtBH8OsuNplnDpw/edit) Reference based quality metrics in three LMAS runs for the ZimoBIOMICS community standards dataset.

#### [Table S23:](https://docs.google.com/spreadsheets/u/0/d/1D0_QM8bycHmDTDm6LkZyr2qWszREmtBH8OsuNplnDpw/edit) Pairwise comparisons of the ZymoBIOMICS microbial community standard reference replicons. All pairwise comparisons among the set of genomes were conducted using Average Nucleotide Identity through BLAST as a proxy for DNA-DNA hybridization.

####

#### 
