# Supplementary material for: LMAS: evaluating metagenomic short de novo assembly methods through defined communities
Source: Gigascience. 2022 Dec 28;12:giac122. doi: 10.1093/gigascience/giac122 (PMC9795473; doi:10.1093/gigascience/giac122)
Supplement: giac122_Response_to_Reviewer_Comments_Original_Submission [file giac122_response_to_reviewer_comments_original_submission.pdf]

Author's Response To Reviewer Comments

Close

September 26, 2022
Catarina Mendes
Institituto de Microbiologia, Instituto de Medicina Molecular
Faculdade de Medicina, Universidade de Lisboa
Av. Prof. Egas Moniz
PT 1649-028 Lisboa
Portugal
Email: cimendes@medicina.ulisboa.pt

Dr Nicole Nogoy
Executive Editor, GigaScience

Dear Dr. Nogoy,

We are grateful for the editorial and referral critique of our paper entitled "LMAS: Last Metagenomic Assembler Standing" (GIGA-D-22-00108) that we submitted for publication in GigaScience. The reviewers' comments led to further analyses on our part which we believe enriched the paper. Moreover, it led us to highlight findings which we believe will be useful to the community, which were not immediately apparent previously, broadening the scope of the original paper. We have also carefully reviewed and modified the manuscript to further clarify in the text the points raised by the reviewers.

The CAMI datasets have remained popular after their publication (Sczyrba et al. 2017), Despite their popularity, no information is provided in the manuscript or in the supplemental material about the source of the genomes in each dataset. It is only mentioned that the community composition was designed according to specified criteria and the metagenome datasets simulated using https://github.com/CAMIchallenge/MetagenomeSimulationPipeline/releases/tag/0.1, which is no longer available. As such, despite the datasets being described as publicly available at https://data.cami-challenge.org/participate, only the "Gold Standard" assemblies are made available, and not the source references for the generation of the mock datasets. As the reviewer suggested, we tried to download the datasets and had difficulties obtaining the dataset following the information provided in the paper. We then contacted the authors and were informed that there were problems with the server where the data was hosted and that the data was unavailable (see attachment). Unfortunately there was no subsequent contact from the CAMI authors indicating the data had again become available.

As an alternative, and keeping in mind the requirement of the inclusion of closely related species in the dataset, we've introduced a new analysis in the manuscript of the BMock12 community (Sevim et al. 2019), composed of 12 species including several closely related sets: two replicons of Halomonas sp. (ANIb=0.98), three replicons of the Micromonospora genus (average ANIb=0.85) and two replicons of Marinobacter sp (ANIb=0.78). Furthermore, and to represent mock community trying to reproduce and existing microbiome, the NIBSC Gut DNA Reference Gut-Mix-RR and Gut-Mix-HiLo community standard (Amos et al. 2020) was analysed, consisting of 20 common gut microbiome strains in an even and staggered composition respectively. These strains have an average ANIb of 0.67, with a maximum of 0.95 between the two Bifidobacterium longum subspecies. It's worth noting that no complete genome is available for eight of the strains in the sample, including one of the Bifidobacterium longum subspecies, therefore taking full advantage of the potential information in this sample is challenging. With the addition of these two datasets, we aim to provide both a high similarity case study as well as the usefulness of this tool using a perhaps more realistic mock community.

LMAS requires defined mock communities as a ground truth. To emphasise this requirement, we've

changed the title of the manuscript to “LMAS: Evaluating metagenomic short de novo assembly methods through defined communities”. Below we will respond to all comments and corrections suggested by the reviewers.

We have submitted a new manuscript, with all changes highlighted in the text. The low quality figures have also been submitted separately to the submission platform in PDF format, as well as Zenodo (https://doi.org/10.5281/zenodo.6783042), including the original dynamic HTML files. Additionally, the reports for the ZymoBIOMICS Microbial Community Standard, BMock12 Community Standard and NIBSC Gut DNA Reference are available at https://doi.org/10.5281/zenodo.7088960, https://doi.org/10.5281/zenodo.7092431 and https://doi.org/10.5281/zenodo.7092693, respectively. We hope to have satisfactorily addressed all the issues raised by the reviewers and hope that you find the revised version of the manuscript acceptable for publication.

With our best regards,
On behalf of all authors,

Catarina Mendes

Reviewer 1 - Major Comments
The authors describe "LMAS: Last Metagenome Assembler Standing", as a platform for evaluating different metagenome assemblers given known communities. Since the field of metagenome assemblers is a rapidly developing one, having good resources for evaluating and selecting methods available is an important topic. The paper itself is well-written and installation of the software via bioconda works out of the box. There are however some problems with the current form of the software and article which need to be addressed.

Regarding the software:

While starting the pipeline is easy enough, LMAS still requires a lot of resources since the assemblers themselves are run. Running the pipeline on the server available to me was not possible though (crashed with the error message "local avail memory attribute cannot zero. expression (avail memory 0). values avail memory = 0"). Using a lower max_cpus and max_memory option and running on a laptop (using the ENN dataset with even errors) was possible, but only a few assemblers were successful and the final report was not created.

We thank the reviewer for the comment. The default CPU and memory values in LMAS are the ones we found were required to successfully assemble the ZymoBIOMICS dataset. Assembly is a resource-intensive process, as is the mapping done in the evaluation of the assemblies by LMAS. Unfortunately, this is a limitation that LMAS cannot circumvent. Although The CPU and memory values can be easily changed, and maximum CPU and memory values defined, we cannot guarantee the success of the assembly process for such data. LMAS is robust to assembler failures, with the possibility of assemblers being skipped from the workflow, as well as an indication in the final report that an assembly process has failed. The cause for a lack of a final report would warrant further investigation, but it could be due to a lack of resources for the mapping process required by LMAS. To address the reviewer’s concerns continuous integration of the LMAS workflow was implemented, for both docker and singularity systems, to ensure that the system is working as intended (https://github.com/B-UMMI/LMAS/actions). Test data is available in LMAS’ repository (https://github.com/B-UMMI/LMAS/tree/main/test/data) consisting of a small in silico sample with just 200,000 read pairs belonging to Bacillus subtilis and Staphylococcus aureus that, due to its small size, is possible to run locally with relatively modest resources for most assemblers (Unicycler still requires a significant amount of resources with such small data).
Regarding the article:

The text mentions assemblers using OLC and dBg, but Table 1 shows only assemblers using deBruijn graphs were selected for evaluation. It is probably not necessary to mention OLC at all, since they have not been used for metagenome assemblers and the software selection reflects that.

We thank the reviewer for the comment. Indeed only dBg graph assemblers were ultimately included in LMAS. As such, and in accordance with the reviewer's suggestion, the mention of OLC assemblers was removed from the background section of the manuscript (lines 60 to 63).

The definition of the Pl-score (Equation 1) seems to be malformed: If E = 1 - Identity and assuming Identity is from 0-1 then E is always <60, so the first condition in Equation 1 needs to be changed.

We thank the reviewer for the comment. Indeed there was a mistake in Equation 1 that has now been corrected to the following:

"Assembly robustness" seems like a strange metric: Deterministic assemblers will always have the most robust results, but assemblers which use randomness are not necessarily worse; it is not clear to me why this is labeled as "poor results". In contrast to that, the LSA metric is an interesting addition to the set of metrics described in e.g. QUAST.

We thank the reviewer for the comment. We agree with the reviewer in regards to the fact that randomness does not translate directly into a worse result, therefore the assembly robustness alone should not be the metric of choice to exclude an assembler. Having said this, inconsistency of results from the same data may confuse more inexperienced users and cancreate difficulties if an assembler is to be included in a pipeline for accreditation. Having said this, we agree with the reviewer that non-deterministic assemblers are, by definition, non-reproducible. While not necessarily worse, they may be problematic for comparison with more deterministic assemblers in terms of the reproducibility of the comparison in different labs or at different times. We believe that the reproducibility of the assemblies generated is a piece of relevant information that should be included when assessing assembler performance. We have therefore changed "assembly robustness" to "assembler reproducibility" in Figure 3 (https://zenodo.org/record/7007317#.Yyn3Huxue3I).

The discovery that single k-mer dBg assemblers are typically outperformed by multi-k ones has been described before (e.g. IDBA-UD) and should be cited.

We thank the reviewer for the comment. Appropriate references have been added, namely (Sczyrba et al. 2017; Meyer et al. 2022; Xavier et al. 2014; Mahadik et al. 2019) (line 345).

Similarly, the dependence of assemblers to recover genomes with low abundance has been described before (e.g. CAMISIM).

We thank the reviewer for the comment. The appropriate reference has been added, namely (Sczyrba et al. 2017; Meyer et al. 2022; Fritz et al. 2019) (line 545).

Again in contrast to that, the influence of the individual species (particularly P. aeruginosa) on the assembly quality could have been elaborated on further.

Following the reviewer's suggestion, the following has been added to the manuscript (lines 470-471): However, in the case of the larger number of contigs of P. aeruginosa, no related species are present in the sample and these possibly reflect intrinsic properties of the replicon such as the high number of prophages integrated in the bacterial genome (Johnson et al, 2019). Unfortunately, existing mock communities do not exist allowing us to expand on the possible importance of replicon intrinsic properties in the quality of the assemblies produced. The availability of LMAS will allow more easily addressing this question by allowing researchers to focus on the experimental design of such communities.

For Figures 4 and Figure 7 many of the points cluster on top of each other, which makes manual inspection of these results hard to do. Generally the figures available to me were of quite low quality, they should

either be vectorized or of higher quality.

We thank the reviewer for this comment. We apologise for the lack of readability regarding Figures 4 and 7, and have uploaded high-resolution PDF versions of each. Figure 5 was also uploaded as a PDF for the same reason. Additionally, All figures are now available, in their original form, in Zenodo under DOI https://doi.org/10.5281/zenodo.6783042. The availability of data and materials has been updated to reflect this addition, now reading "Likewise, all figures in the current manuscript are available in their original format in the Zenodo repository, under https://doi.org/10.5281/zenodo.6783042." (lines 660-662).

The conclusion that metagenome assemblers do not outperform genome assemblers is based on the - low complexity - data sets. This problem is touched upon in the conclusion but the header in the main text can be misleading. To come to the conclusion that metagenome assemblers do not outperform genome assemblers, LMAS would need to show this on at least one dataset with somewhat realistic complexity, e.g. the ones used for the mentioned CAMI challenge. Doing this could also show the advantage of LMAS against the mainly used QUAST in bundling the execution and evaluation together.

As stated throughout the manuscript, LMAS requires defined mock communities as a source of ground truth. We appreciate the reviewer's comment regarding the misleading nature of the header of the manuscript. To emphasise this requirement, we've changed the title of the manuscript to "LMAS: Evaluating metagenomic short de novo assembly methods through defined communities"

Given the problems in obtaining the CAMI samples we have tried to find relevant mock communities, sequenced using short-read technologies,of greater complexity than the ZymoBIOMICS community. Keeping in mind the requirement of the inclusion of closely related strains in the dataset, we've introduced a new analysis in the manuscript of the BMock12 community (Sevim et al. 2019). The challenges of this dataset are the two Halomonas and the two Marinobacter strains, which come with an average ANIb of 0.98 and 0.85 (see Supplementary Table S25), respectively. Additionally, the NIBSC Gut DNA Reference Gut-Mix-RR and Gut-Mix-HiLo datasets were analysed, representing a more naturally occurring microbiome that includes 5 phyla, 13 families, 16 genera and 19 species that includes several abundant species and strains present in the human gut (Amos et al. 2020). With this, we wish to better showcase the usefulness of this tool using more difficult datasets. The reports for the ZymoBIOMICS Microbial Community Standard, BMock12 Community Standard and NIBSC Gut DNA Reference are available at https://doi.org/10.5281/zenodo.7088960, https://doi.org/10.5281/zenodo.7092431 and https://doi.org/10.5281/zenodo.7092693 respectively (lines 666-669 in the manuscript).
Following the analysis of these new datasets, the conclusion that the metagenomics assemblers do not outperform the genomic assemblers still holds true.

Reviewer 2 - Major Comments

This is a technical note that describes LMAS, a tool that launches multiple genome assemblers on the same metagenomic dataset and then generates a report with various assembly quality metrics. The metrics, including two newly introduced ones, make sense. The choice of assemblers also generally makes sense, although I do have some remarks below regarding a couple choices. The analysis is thorough and provides some interesting takeaway messages. The manuscript is well-written and the presentation is didactic.

My main doubt about this article is whether the LMAS tool would be of broad interest. Between existing large-scale benchmarking efforts like CAMI and popular evaluation tools that generate assembly reports like MetaQUAST, it is difficult to assess the precise need for LMAS. The suggested use case by the authors is that a user should run him/herself the assemblers on a particular dataset of interest. The advantage would therefore be to have an integrated solution to run all tools and evaluate the results automatically. While this is handy, a user might also simply run a 2-3 of the best assemblers as suggested by the article (or CAMI), considering that many of the LMAS produce suboptimal assemblies and they are not reconciled.

A redeeming aspect of the article is the newly made benchmark on low-complexity Zymo real & simulated data (8 bacterial species). This benchmark contains some interesting findings, such as the fact that genomic assemblers appear to perform as well a metagenomes ones. This claim should however be put into context: the metagenomic data is low-complexity (only 8 species), and the presence of similar strains in actual metagenomes may significantly change that message.

The authors may want to make a stronger case why a user would want to run LMAS instead of picking 2 (or 3) of the best-performing assemblers and run MetaQUAST on them. In an actual assembly scenario, i.e. without ground truth, are there situations where running more than 2 assemblers is beneficial? I am genuinely interested in the answer.

We thank the reviewer for this comment. The purpose of LMAS is, given a ground truth of a defined mock community, to enable the benchmarking of traditional and metagenomic prokaryotic de novo assembly software. Although we understand the reviewer's point that CAMI offers this guidance, CAMI challenges are spaced in time and assemblers are released at a much faster pace, so a user may be interested in evaluating a novel assembler without the "self-assessment" trap associated with the publication of novel tools before the next CAMI challenge. Moreover, our analyses show that most of the conclusions on assembler performance are not generalisable, therefore there is no absolute guarantee that the best performing assemblers for a given scenario will be the same as for a different one (namely considering the replicon of interest, its abundance in the sample and other replicons present in the sample). Therefore, we encourage users to run the assemblers of interest with mock communities as close as possible to their sample of interest. Admittedly, a user may want to concentrate on 2-3 assemblers, but the task of running these assemblers followed by MetaQUAST may still be daunting. LMAS, with its modular and streamlined design, lowers the barrier of performing such analyses. Moreover, in addition to the novel quality metrics, LMAS offers representations which are not offered by MetaQUAST, such as the positions of assembly gaps and of SNPs in the genomes of interest which can be explored in the future to further understand the strengths and weaknesses of particular assemblers.

To better reinforce some of these points, the following sections have been added to the text: "The portability and ease of use of LMAS are intended to provide users with a continuous benchmarking platform to easily evaluate the performance of assemblers, as they are developed, in mock communities mimicking as closely as possible their samples of interest." and "Even when considering communities with very similar replicons, the overall performance of metagenomic assemblers was not consistently better than that of genomic assemblers. The results also indicate that the recovery of assemblies allowing strain-level discrimination at the SNP level is highly unlikely based solely on the assembler generated contigs." (lines 580-583).

Were there multiple high-similarity strains in the Zymo data? If not, this should be noted in the presentation of the dataset. This is an important caveat that limits the significance of the reported results. In particular I would suggest to tone down the claim that genomic assemblers perform as well as metagenomic assemblers, given e.g. the many heuristics that MetaSpades implements to resolve close strains.

We thank the reviewer for this comment. The similarity of the strains in the ZymoBiomics is available as supplemental table S23, where pairwise comparisons of the microbial community standard reference replicons are available. As observed in this table, Escherichia coli and Salmonella enterica have the highest level of similarity, with an ANIb of 0.80.

Keeping in mind the need of analysing closely related species in the same mock community, we've introduced a new analysis in the manuscript of the BMock12 community (Sevim et al. 2019), composed of 12 strains including several closely related sets: two replicons of Halomonas sp. (ANIb=0.98), three replicons of the Micromonospora genus (average ANIb=0.85) and two replicons of Marinobacter sp (ANIb=0.78). We hope that, with the introduction of the analysis of this dataset, we have successfully addressed the scenario raised by the reviewer where multiple similar strains are present in the sample. Essentially, our initial assertion stands, with genomic assemblers not being systematically outperformed by metagenomic assemblers.

Keeping in mind the reviewer suggestions, the following has been added to the conclusion of the manuscript (lines 567-573): “Although the eight species ZymoBIOMICS Microbial Community Standards might not be representative of the metagenomic complexity of the samples of interest of most researchers, we hoped that its relative simplicity meant that the results shown would represent a best-case scenario, since as sample complexity increases so do the challenges to assembler performance. However, the results of the BMock12 and Gut-Mix community standards suggest that the actual genome of interest and community composition play an important part in the results of individual assemblers.”.

The abstract sentence: "Some assemblers still in use, such as ABySS, BCALM2, MetaHipmer2, minia and VelvetOptimiser" should be removed or revised as it is misleading for several reasons: BCALM2 is not an assembler, it constructs unitigs only. Minia is a part of GATBMiniaPipeline which offers better performance. And VelvetOptimiser is an odd choice as MetaVelvet also exists and was untested (although it might not scale). Therefore, I recommend VelvetOptimiser, BCALM2, and possibly minia to be removed from the benchmark, or be marked as odd choices for metagenome assembly.

We thank the reviewer for this comment. MetaVelvet has been last updated in 2015 (Supplemental Table S1), therefore it was not considered for the benchmark by not meeting our inclusion criteria. Due to the very fair point made regarding BCALM2, it has been removed from LMAS (https://github.com/B-UMMI/LMAS/pull/25), and the manuscript, figures and supplemental material adjusted accordingly. Regarding minia, a similar argument can be made for SPAdes and Unicycler assemblers. Therefore it has not been removed from LMAS and is still considered in the initial benchmark with the ZymoBIOMICS Microbial Community Standard, together with VelvetOptimiser. Given their very poor performance, their execution is now by default set to false in LMAS, alongside Abyss and MetaHipermer2, since given the results presented here these would not be recommended assemblers.

Of note, albeit VelvetOptimiser tries multiple k values, it is still considered to be a single-k assembler as it does not combine the results of multiple iterations. (It merely picks the best k and runs single-k.)

We thank the reviewer for this comment. Table 1 and supplemental Table S1 have been adjusted accordingly.

Some of the results are obvious, e.g. single-k assemblers perform more poorly than multiple-k assemblers; this is not quite a novel finding.

We thank the reviewer for this comment. Indeed the notion that multiple k-mer assemblers outperform single k-mer assemblers is not novel, but it is our opinion that there is still limited data supporting this assertion. Therefore, we found it worth mentioning and reinforcing this notion in our manuscript. As such, we have not changed the text. Instead, and in accordance with the comments of reviewer 1, the following references have been added to support this claim: (Sczyrba et al. 2017; Meyer et al. 2022; Xavier et al. 2014; Mahadik et al. 2019) (line 345 in the manuscript).

Figures 4 and 5, even when downloaded as PNG, were low resolution and hardly readable.

We thank the reviewer for this comment. We apologise for the lack of readability regarding Figures 4 and 5, and have uploaded a high-resolution PDF version of each. Additionally, All figures are now available, in their original form, in Zenodo under DOI https://doi.org/10.5281/zenodo.6783042 (lines 660-662 in the manuscript).

Reviewer 2 - Minor Comments

The term "replicon" is used ubiquitously in the paper, and while its definition is biologically sound, it could be worthwhile to recall what the authors precisely mean by it.

We thank the reviewer for the comment. In this manuscript, the term "replicon" is used to refer to DNA sequences replicating as a single unit, containing a single origin of replication, and therefore excludes the genomes of eukaryotes which can initiate replication from several loci. We’ve altered the following sentence

in the background section of the manuscript (lines 49 to 51) to better inform the reader: The de novo assembly process is key when analysing metagenomic data since it allows recovering contigs representing the replicons present in the sample, be it prokaryotic chromosomes, plasmids or viruses, from a pool of mixed raw reads.

While running LMAS on my cluster head node. Some issues occur. The pulling of singularity images did not work. The error message was:

Error executing process > 'preprocessing_wf:PROCESS_REFERENCE (1)'

Caused by:
Failed to pull singularity image
command: singularity pull --name cimendes-lmas-python-base-0.1.img.pulling.1654889379926 docker://cimendes/lmas-python-base:0.1 > /dev/null
status : 255
message:
INFO: Converting OCI blobs to SIF format
FATAL: While making image from oci registry: error fetching image to cache: while building SIF from layers: unable to create new build: while ensuring correct compression algorithm: while creating squashfs: create command failed: exit status 1: FATAL ERROR:Failed to create thread

However, running the command myself:

$ singularity pull --name cimendes-lmas-python-base-0.1.img.pulling.1654889379926 docker://cimendes/lmas-python-base:0.1

Worked.
Then, after that, since I need to run LMAS on the head node for the first time (other nodes are not connected to internet so cannot pull containers), I get the following error message:

Error executing process > 'assembly_wf:SKESA (ERR2935805)'

Caused by:
Process requirement exceed available CPUs -- req: 8; avail: 4

In summary, it would be helpful to have instructions to pull all containers from the head node ahead of time to prepare for LMAS execution on a node not connected to Internet.

We thank the reviewer for this observation. Continuous integration with singularity has been added to LMAS under https://github.com/B-UMMI/LMAS/pull/23. Indeed we observe that the pull of the images with singularity is sometimes inconsistent but restarting the workflow solves the issue most times. Additionally, in the LMAS documentation the requirement of an internet connection to download the required images was added as Nextflow automatically handles the pull of the required images. Alternatively, a script has been included to handle the pulling of the required images using shifter, docker or singularity (https://github.com/B-UMMI/LMAS/pull/31) before running LMAS for the first time to allow users to run LMAS without the problems described by the reviewer.
References

Amos, Gregory C. A., Alastair Logan, Saba Anwar, Martin Fritzsche, Ryan Mate, Thomas Bleazard, and Sjoerd Rijpkema. 2020. ‘Developing Standards for the Microbiome Field’. Microbiome 8 (1): 98. https://doi.org/10.1186/s40168-020-00856-3.
Fritz, Adrian, Peter Hofmann, Stephan Majda, Eik Dahms, Johannes Dröge, Jessika Fiedler, Till R. Lesker, et

al. 2019. ‘CAMISIM: Simulating Metagenomes and Microbial Communities’. Microbiome 7 (1): 17. https://doi.org/10.1186/s40168-019-0633-6.
Mahadik, Kanak, Christopher Wright, Milind Kulkarni, Saurabh Bagchi, and Somali Chaterji. 2019. ‘Scalable Genome Assembly through Parallel de Bruijn Graph Construction for Multiple K-Mers’. Scientific Reports 9 (1): 14882. https://doi.org/10.1038/s41598-019-51284-9.
Meyer, Fernando, Adrian Fritz, Zhi-Luo Deng, David Koslicki, Till Robin Lesker, Alexey Gurevich, Gary Robertson, et al. 2022. ‘Critical Assessment of Metagenome Interpretation: The Second Round of Challenges’. Nature Methods 19 (4): 429–40. https://doi.org/10.1038/s41592-022-01431-4.
Sczyrba, Alexander, Peter Hofmann, Peter Belmann, David Koslicki, Stefan Janssen, Johannes Dröge, Ivan Gregor, et al. 2017. ‘Critical Assessment of Metagenome Interpretation—a Benchmark of Metagenomics Software’. Nature Methods 14 (11): 1063–71. https://doi.org/10.1038/nmeth.4458.
Sevim, Volkan, Juna Lee, Robert Egan, Alicia Clum, Hope Hundley, Janey Lee, R. Craig Everroad, et al. 2019. ‘Shotgun Metagenome Data of a Defined Mock Community Using Oxford Nanopore, PacBio and Illumina Technologies’. Scientific Data 6 (1): 285. https://doi.org/10.1038/s41597-019-0287-z.
Xavier, Basil Britto, Julia Sabirova, Moons Pieter, Jean-Pierre Hernalsteens, Henri de Greve, Herman Goossens, and Surbhi Malhotra-Kumar. 2014. ‘Employing Whole Genome Mapping for Optimal de Novo Assembly of Bacterial Genomes’. BMC Research Notes 7 (1): 484. https://doi.org/10.1186/1756-0500-7-484.

Close
