# Supplementary material for: LMAS: evaluating metagenomic short de novo assembly methods through defined communities
Source: Gigascience. 2022 Dec 28;12:giac122. doi: 10.1093/gigascience/giac122 (PMC9795473; doi:10.1093/gigascience/giac122)
Supplement: giac122_Reviewer_1_Report_Original_Submission — Adrian Fritz -- 6/7/2022 Reviewed [file giac122_reviewer_1_report_original_submission.pdf]

Reviewer Report

Title: LMAS: Evaluating metagenomic short de novo assembly methods through defined communities

Version: Original Submission **Date:** 6/7/2022

Reviewer name: Adrian Fritz

Reviewer Comments to Author:

The authors describe "LMAS: Last Metagenome Assembler Standing", a platform for evaluating different metagenome assemblers given known communities. Since the field of metagenome assemblers is a rapidly developing one, having good resources for evaluating and selecting methods available is an important topic. The paper itself is well-written and installation of the software via bioconda works out of the box. There are however some problems with the current form of the software and article which need to be addressed.Regarding the software:While starting the pipeline is easy enough, LMAS still requires a lot of resources since the assemblers themselves are run. Running the pipeline on the server available to me was not possible though (crashed with the error message "local avail memory attribute cannot zero. expression (avail memory 0). values avail memory = 0"). Using a lower max_cpus and max_memory option and running on a laptop (using the ENN dataset with even errors) was possible, but only a few assemblers were succesful and the final report was not created.Regarding the article:The text mentions assemblers using OLC and dBg, but Table 1 shows only assemblers using deBruijn graphs were selected for evaluation. It is probably not necessary to mention OLC at all, since they have not been used for metagenome assemblers and the software selection reflects that.The definition of the Pl-score (Equation 1) seems to be malformed: If E = 1 - Identity and assuming Identity is from 0-1 then E is always <60, so the first condition in Equation 1 needs to be changed."Assembly robustness" seems like a strange metric: Deterministic assemblers will always have the most robust results, but assemblers which use randomness are not necessarily worse; it is not clear to me why this is labeled as "poor results". In contrast to that, the LSA metric is an interesting addition to the set of metrics described in e.g. QUAST.The discovery that single k-mer dBg assemblers are typically outperformed by multi-k ones has been described before (e.g. IDBA-UD) and should be cited.Similarily, the dependence of assemblers to recover genomes with low abundance has been described before (e.g. CAMISIM).Again in contrast to that, the influence of the individual species (particularly P. aeruginosa) on the assembly quality could have been elaborated on further.For Figures 4 and Figure 7 many of the points cluster on top of each other, which makes manual inspection of these results hard to do. Generally the figures available to me where of quite low quality, they should either be vectorized or of higher quality.The conclusion that metagenome assemblers do not outperform genome assemblers is based on the - low complexity - data sets. This problem is touched upon in the conclusion but the header in the main text can be misleading. To come to the conclusion that metagenome assemblers do not outperform genome assemblers, LMAS would need to show this on at least one dataset with somewhat realistic complexity, e.g. the ones used for the mentioned CAMI challenge. Doing this could also show the advantage of LMAS against the mainly used QUAST in bundling the execution and evaluation together.

Level of Interest

Please indicate how interesting you found the manuscript: Choose an item.

Quality of Written English

Please indicate the quality of language in the manuscript: Choose an item.

Declaration of Competing Interests

Please complete a declaration of competing interests, considering the following questions:

- Have you in the past five years received reimbursements, fees, funding, or salary from an organisation that may in any way gain or lose financially from the publication of this manuscript, either now or in the future?
- Do you hold any stocks or shares in an organisation that may in any way gain or lose financially from the publication of this manuscript, either now or in the future?
- Do you hold or are you currently applying for any patents relating to the content of the manuscript?
- Have you received reimbursements, fees, funding, or salary from an organization that holds or has applied for patents relating to the content of the manuscript?
- Do you have any other financial competing interests?
- Do you have any non-financial competing interests in relation to this paper?

If you can answer no to all of the above, write 'I declare that I have no competing interests' below. If your reply is yes to any, please give details below.

I declare that I have no competing interests

I agree to the open peer review policy of the journal. I understand that my name will be included on my report to the authors and, if the manuscript is accepted for publication, my named report including any attachments I upload will be posted on the website along with the authors' responses. I agree for my report to be made available under an Open Access Creative Commons CC-BY license (http://creativecommons.org/licenses/by/4.0/). I understand that any comments which I do not wish to be included in my named report can be included as confidential comments to the editors, which will not be published.

Choose an item.

To further support our reviewers, we have joined with Publons, where you can gain additional credit to further highlight your hard work (see: https://publons.com/journal/530/gigascience). On publication of this paper, your review will be automatically added to Publons, you can then choose whether or not to claim your Publons credit. I understand this statement.

Yes Choose an item.
