# Supplementary material for: LMAS: evaluating metagenomic short de novo assembly methods through defined communities
Source: Gigascience. 2022 Dec 28;12:giac122. doi: 10.1093/gigascience/giac122 (PMC9795473; doi:10.1093/gigascience/giac122)
Supplement: giac122_Reviewer_2_Report_Original_Submission — Rayan Chikhi -- 6/10/2022 Reviewed [file giac122_reviewer_2_report_original_submission.pdf]

Reviewer Report

Title: LMAS: Evaluating metagenomic short de novo assembly methods through defined communities

Version: Original Submission **Date:** 6/10/2022

Reviewer name: Rayan Chikhi

Reviewer Comments to Author:

This is a technical note that describes LMAS, a tool that launches multiple genome assemblers on the same metagenomic dataset and then generates a report with various assembly quality metrics. The metrics, including two newly introduced ones, make sense. The choice of assemblers also generally makes sense, although I do have some remarks below regarding a couple choices. The analysis is thorough and provides some interesting takeaway messages. The manuscript is well-written and the presentation is didactic.My main doubt about this article is whether the LMAS tool would be of broad interest. Between existing large-scale benchmarking efforts like CAMI and popular evaluation tools that generate assembly reports like MetaQUAST, it is difficult to assess the precise need for LMAS. The suggested use case by the authors is that a user should run him/herself the assemblers on a particular dataset of interest. The advantage would therefore be to have an integrated solution to run all tools and evaluate the results automatically. While this is handy, a user might also simply run a 2-3 of the best assemblers as suggested by the article (or CAMI), considering that many of the LMAS produce suboptimal assemblies and they are not reconciled.A redeeming aspect of the article is the newly made benchmark on low-complexity Zymo real & simulated data (8 bacterial species). This benchmark contains some interesting findings, such as the fact that genomic assemblers appear to perform as well a metagenomes ones. This claim should however be put into context: the metagenomic data is low-complexity (only 8 species), and the presence of similar strains in actual metagenomes may significantly change that message.Remarks:* The authors may want to make a stronger case why a user would want to run LMAS instead of picking 2 (or 3) of the best-performing assemblers and run MetaQUAST on them. In an actual assembly scenario, i.e. without ground truth, are there situations where running more than 2 assemblers is beneficial? I am genuinely interested in the answer.* Were there multiple high-similarity strains in the Zymo data? If not, this should be noted in the presentation of the dataset. This is an important caveat that limits the significance of the reported results. In particular I would suggest to tone down the claim that genomic assemblers perform as well as metagenomic assemblers, given e.g. the many heuristics that MetaSpades implements to resolve close strains.* The abstract sentence: "Some assemblers still in use, such as ABySS, BCALM2, MetaHipmer2, minia and VelvetOptimiser" should be removed or revised as it is misleading for several reasons: BCALM2 is not an assembler, it constructs unitigs only. Minia is a part of GATBMiniaPipeline which offers better performance. And VelvetOptimiser is an odd choice as MetaVelvet also exists and was untested (although it might not scale). Therefore, I recommend VelvetOptimiser, BCALM2, and possibly minia to be removed from the benchmark, or be marked as odd choices for metagenome assembly.* Of note, albeit VelvetOptimiser tries multiple k values, it is still considered to be a single-k assembler as it does not combine the results of multiple iterations. (It merely picks the best k and runs single-k.)* Some of the results are obvious, e.g. single-k

assemblers perform more poorly than multiple-k assemblers; this is not quite a novel finding.* Figures 4 and 5, even when downloaded as PNG, were low resolution and hardly readable.Minor:* The term "replicon" is used ubiquitously in the paper, and while its definition is biologically sound, it could be worthwhile to recall what the authors precisely mean by it.* While running LMAS on my cluster head node. Some issues occur. The pulling of singularity images did not work. The error message was:Error executing process > 'preprocessing_wf:PROCESS_REFERENCE (1)'Caused by:Failed to pull singularity imagecommand: singularity pull --name cimendes-lmas-python-base-0.1.img.pulling.1654889379926 docker://cimendes/lmas-python-base:0.1 > /dev/nullstatus : 255message:INFO: Converting OCI blobs to SIF formatFATAL: While making image from oci registry: error fetching image to cache: while building SIF from layers: unable to create new build: while ensuring correct compression algorithm: while creating squashfs: create command failed: exit status 1: FATAL ERROR:Failed to create threadHowever, running the command myself:$ singularity pull --name cimendes-lmas-python-base-0.1.img.pulling.1654889379926 docker://cimendes/lmas-python-base:0.1Worked.Then, after that, since I need to run LMAS on the head node for the first time (other nodes are not connected to internet so cannot pull containers), I get the following error message:Error executing process > 'assembly_wf:SKESA (ERR2935805)'Caused by:Process requirement exceed available CPUs -- req: 8; avail: 4In summary, it would be helpful to have instructions to pull all containers from the head node ahead of time to prepare for LMAS execution on a node not connected to Internet.

Level of Interest

Please indicate how interesting you found the manuscript: Choose an item.

Quality of Written English

Please indicate the quality of language in the manuscript: Choose an item.

Declaration of Competing Interests

Please complete a declaration of competing interests, considering the following questions:

- Have you in the past five years received reimbursements, fees, funding, or salary from an organisation that may in any way gain or lose financially from the publication of this manuscript, either now or in the future?
- Do you hold any stocks or shares in an organisation that may in any way gain or lose financially from the publication of this manuscript, either now or in the future?
- Do you hold or are you currently applying for any patents relating to the content of the manuscript?
- Have you received reimbursements, fees, funding, or salary from an organization that holds or has applied for patents relating to the content of the manuscript?
- Do you have any other financial competing interests?
- Do you have any non-financial competing interests in relation to this paper?

If you can answer no to all of the above, write 'I declare that I have no competing interests' below. If your reply is yes to any, please give details below.

I declare that I have no competing interests.

I agree to the open peer review policy of the journal. I understand that my name will be included on my report to the authors and, if the manuscript is accepted for publication, my named report including any attachments I upload will be posted on the website along with the authors' responses. I agree for my report to be made available under an Open Access Creative Commons CC-BY license (http://creativecommons.org/licenses/by/4.0/). I understand that any comments which I do not wish to be included in my named report can be included as confidential comments to the editors, which will not be published.

Choose an item.

To further support our reviewers, we have joined with Publons, where you can gain additional credit to further highlight your hard work (see: https://publons.com/journal/530/gigascience). On publication of this paper, your review will be automatically added to Publons, you can then choose whether or not to claim your Publons credit. I understand this statement.

Yes Choose an item.
